# Extreme genome scrambling in marine planktonic *Oikopleura dioica* cryptic species

**DOI:** 10.1101/gr.278295.123

**Published:** 2024-03

**Authors:** Charles Plessy, Michael J. Mansfield, Aleksandra Bliznina, Aki Masunaga, Charlotte West, Yongkai Tan, Andrew W. Liu, Jan Grašič, María Sara del Río Pisula, Gaspar Sánchez-Serna, Marc Fabrega-Torrus, Alfonso Ferrández-Roldán, Vittoria Roncalli, Pavla Navratilova, Eric M. Thompson, Takeshi Onuma, Hiroki Nishida, Cristian Cañestro, Nicholas M. Luscombe

**Affiliations:** 1 Genomics and Regulatory Systems Unit, Okinawa Institute of Science and Technology Graduate University (OIST), Onna-son, Okinawa 904-0495, Japan;; 2 Departament de Genètica, Microbiologia i Estadística, Facultat de Biologia, Universitat de Barcelona (UB), Barcelona 08028, Spain;; 3 Institut de Recerca de la Biodiversitat (IRBio), Universitat de Barcelona (UB), Barcelona 08028, Spain;; 4 Centre of Plant Structural and Functional Genomics, Institute of Experimental Botany, 779 00 Olomouc, Czech Republic;; 5 Sars International Centre, University of Bergen, Bergen N-5008, Norway;; 6 Department of Biological Sciences, University of Bergen, Bergen N-5020, Norway;; 7 Faculty of Science, Kagoshima University, Kagoshima 890-0065, Japan;; 8 Department of Biological Sciences, Graduate School of Science, Osaka University, Toyonaka, Osaka 560-0043, Japan

## Abstract

Genome structural variations within species are rare. How selective constraints preserve gene order and chromosome structure is a central question in evolutionary biology that remains unsolved. Our sequencing of several genomes of the appendicularian tunicate *Oikopleura dioica* around the globe reveals extreme genome scrambling caused by thousands of chromosomal rearrangements, although showing no obvious morphological differences between these animals. The breakpoint accumulation rate is an order of magnitude higher than in ascidian tunicates, nematodes, *Drosophila,* or mammals. Chromosome arms and sex-specific regions appear to be the primary unit of macrosynteny conservation. At the microsyntenic level, scrambling did not preserve operon structures, suggesting an absence of selective pressure to maintain them. The uncoupling of the genome scrambling with morphological conservation in *O. dioica* suggests the presence of previously unnoticed cryptic species and provides a new biological system that challenges our previous vision of speciation in which similar animals always share similar genome structures.

The concept of “reference genome” for each species comes from the notion that genomic structural variations and chromosomal rearrangements within species are rare, which is a fundamental aspect sustaining projects such as the Earth Biogenome Project (EBP) (Damas et al. 2021). It is widely accepted that, in each species, the distribution and order of genes on chromosomes are not random, as changes in gene order are likely to affect the regulation of gene expression, and in humans, it has been intimately associated with a variety of diseases, including cancer (Li et al. 2020). How evolution acts on the preservation or variation of gene order within species, or even between closely related organisms, remains poorly understood.

Comparisons of distantly related groups of metazoans have revealed gene linkages within chromosomes that have been preserved for more than half a billion years (Simakov et al. 2022). The conservation of gene linkage is a feature referred to as “conserved synteny,” from the Greek meaning “same ribbon,” which describes homologous genes that colocate, independently of order, within a single chromosome (Passarge et al. 1999). Differences in the scale and extent of synteny conservation have led to the concepts of micro- and macrosynteny. Microsynteny (also known as “collinearity” in genomics) refers to the conservation of gene content and order within sets of tightly linked orthologous genes. Generally, closely related species tend to possess greater conservation of microsynteny, and for this reason, it can even be used to clarify phylogenies (Drillon et al. 2020; Pereira-Santana et al. 2020). Although microsynteny is generally weakly conserved in distantly related species, the remnants of ancient linkage karyotype groups can be detected at the chromosome scale; the conservation of genes on chromosomes that can be traced back to an ancestral karyotype is reflected in the concept of macrosynteny, examples of which include the chromosomal conservation that can be traced back to the last common ancestor of metazoans (Simakov et al. 2022). The most famous example of conserved microsynteny in animals is the *Hox* cluster, which contains genes that regulate axial patterning during embryogenesis and whose ancestry can be traced back to the origin of bilaterian animals hundreds of millions of years ago (for a recent review, see Wanninger 2024). There are many other examples of highly conserved microsyntenies across metazoans, in many cases related to the functional constraints imposed by *cis*-regulatory elements on the coordinated transcription of nearby genes. This includes genomic regulatory blocks (GRBs), within which the action of conserved noncoding elements allows the coordinated expression of genes in a local genomic neighborhood (Hurst et al. 2004; Engström et al. 2007; Irimia et al. 2012; Rowley and Corces 2018). Thus, evaluating the conservation and loss of synteny can provide important information for generating testable hypotheses related to gene regulation, genome biology, and evolution.

The loss of synteny can be provoked by genome rearrangements, such as chromosome translocations related to unequal recombination, or by chromosome fragment mobilization owing to transposon activity. Both of these processes can result in changes of gene order and the reallocation of genes to different genomic neighborhoods. The accumulation over time of many rearrangements results in genome “scrambling,” a concept that in linguistics refers to language syntaxes that permit changes in word order without altering the meaning of a sentence. Scrambling has been used to describe the patterns of synteny loss in genomic comparisons of distantly related species, such as fugu and humans, whose genome organization has significantly diverged over hundreds of millions of years (Aparicio et al. 2002). However, fundamental questions remain, such as how evolutionary forces act to constrain or accelerate the rate of rearrangement or how phenotypic differences could be related to rearrangements. Addressing these problems is difficult at large time scales and genetic distances.

Chromosomes and chromosomal rearrangements have been a classic topic of discussion in evolutionary biology, in support of Darwin's theory of natural selection and the origin of species (Darwin 1859; Dobzhansky 1937; Goldschmidt 1940), for the enormous potential that these chromosomal changes can become raw material for evolution, enabling populations to quickly isolate from each other and facilitating the rapid evolution of adaptations to sudden environmental variations. The description of chromosomal rearrangements associated with adaptive phenotypes (Joron et al. 2011; Lamichhaney et al. 2016) has brought renewed attention to this area, especially given the advent of sequence technologies that enable high-quality, telomere-to-telomere, chromosome-scale genome assemblies across the tree of life, such as those produced under the umbrella of the EBP (for review, see Damas et al. 2021). Lepidopterans provide one of the most enigmatic examples of how genome rearrangements are responsible for the speciation and adaptations such as mimicry (Joron et al. 2011; Hill et al. 2019; de Vos et al. 2020), mostly owing to reciprocal translocation and repeated events of fusion and fission among a highly dynamic number of chromosomes between species.

To better understand the phenomenon of genome scrambling, we study the zooplanktonic appendicularian tunicate *Oikopleura dioica. O. dioica* has the smallest nonparasitic animal genome reported to date (Seo et al. 2004; Denoeud et al. 2010; Wang et al. 2020a; Bliznina et al. 2021). This genome reduction appears to be the result of a drastic process of compaction involving a reduction in repeat content (∼15%) (Henriet et al. 2015), as well as numerous gene losses (for a review, see Ferrández-Roldán et al. 2021). *O. dioica*’s karyotype comprises three chromosome pairs (Körner 1952; Liu et al. 2020): two acrocentric autosomes and an acrocentric X and Y sex chromosome containing a long pseudoautosomal region (PAR) connected to sex-specific regions by a ribosomal DNA locus (Denoeud et al. 2010; Bliznina et al. 2021). The Y-specific region is repeat-rich and gene-poor and differs from all other genomic regions. Chromosome contact analysis of *O. dioica* suggests that there is relatively little interaction between the arms of individual chromosomes or sex-specific regions, which corresponds with the “type-I” genome architecture reported by Hoencamp et al. (2021). In *O. dioica*, a significant fraction of genes is densely packed in a head-to-tail configuration and transcribed in polycistronic mRNAs, forming operons, which are processed by the addition of a *trans*-spliced leader RNA (Ganot et al. 2004), similar to the operons seen in other eukaryotic taxa (Van der Ploeg 1986; Stover and Steele 2001; Blumenthal and Gleason 2003; Zayas et al. 2005; Zhang et al. 2007; Zeller 2010). In contrast to bacterial operons, in which cotranscribed genes tend to be functionally related, in *O. dioica* the functions of genes in operons are more loosely related, with a trend toward housekeeping, cell cycle, translation, and germline functions (Zeller 2010; Danks et al. 2015; Wang et al. 2015). How operons might relate to genome scrambling is not known. At the same time, genome compaction in *O. dioica* also appears to have been accompanied by a drastic loss of conserved microsynteny compared with other chordate genomes, including the disintegration of the paradigmatic *Hox* cluster (Seo et al. 2004).

Our recent study of cross-fertility and molecular markers revealed that *O. dioica* sampled from the Japanese Seto inland sea (Osaka University laboratory strain), from the subtropical island of Okinawa, Japan (OIST laboratory strain), and from the Mediterranean Catalan coast (University of Barcelona laboratory strain) (Fig. 1A) were reproductively isolated and showed high genetic distance. Despite this, we were unable to identify reliable morphological characteristics that could be used in the field to distinguish these samples without the need for crossing experiments or DNA sequencing, suggesting that these are cryptic species (Masunaga et al. 2022). A collegial discussion on a possible taxonomy update is currently taking place in the tunicate scientific community, and to facilitate a consensual conclusion, as well as for the sake of simplicity in this paper, we will refer to these populations as “lineages” named after the location in which specimens have been collected or from which laboratory cultures have originated. The telomere-to-telomere genome assembly of *O. dioica* from Okinawa (Bliznina et al. 2021) further implied the existence of differences in gene organization compared with genome sequences obtained from individuals sampled in Osaka (Wang et al. 2020a) and Bergen (Norway) (Denoeud et al. 2010), but this possibility could not be investigated properly without chromosome-scale assemblies for all lineages. Worse, the apparent level of synteny compared with chromosomes from the Okinawa lineage was variable between contigs. Here, using chromosome-scale genome assemblies, we report a substantial degree of genomic rearrangement between *O. dioica* lineages, describing the genomic features that underlie this genomic scrambling and laying the foundations toward making *O. dioica* an attractive system to study the loss of conserved synteny in the absence of obvious phenotypic differences.

**Figure 1.**
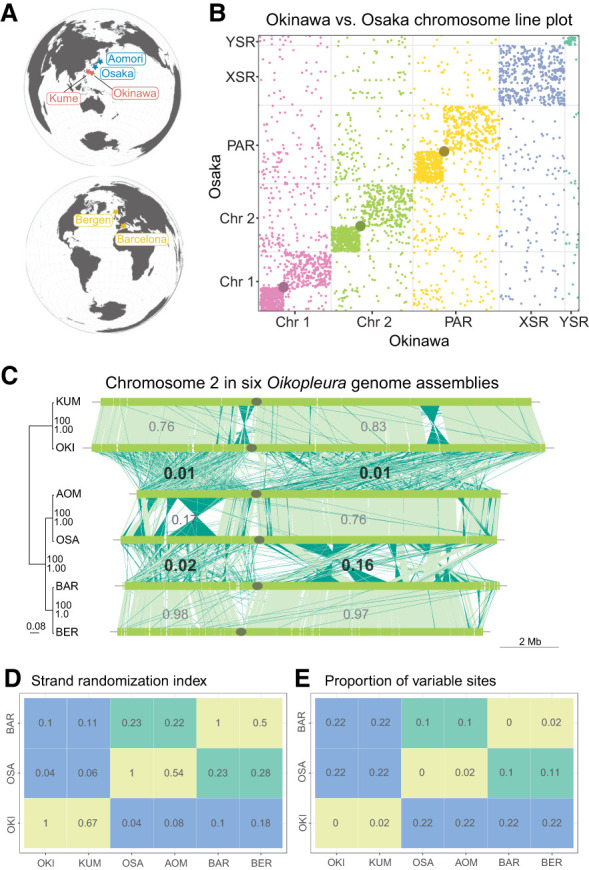
Extensive genomic rearrangement in *Oikopleura dioica* lineages. (*A*) Geographical map locating the origin of the lineages and assemblies. (*B*) Line plot representation of the whole-genome alignment between the Okinawa and Osaka genomes. Each chromosome is plotted in a different color that will identify them in the following figures. A gray dot is overlaid at the position of centromeres. (*C*, *left*) Maximum likelihood phylogenetic tree of 5162 single-copy orthologous genes common to all *O. dioica* genomes. The tree is midpoint-rooted with clade support values indicating bootstrap values from RAxML and Bayesian posterior probability from MrBayes. Branch lengths are proportional to the estimated number of substitutions per nucleotide site. (*Right*) Pairwise comparisons of Chromosome 2 between *Oikopleura* genomes (names abridged with their first three letters). Dark green indicates plus/minus-strand alignments; the gray ellipse, the centromere. The numbers indicate the scrambling index computed for a given arm pair. (*D*,*E*) Scrambling index (*D*) and proportion of variable sites (*E*) across all single-copy orthologous nucleotide sequences for same-lineage (green), Osaka–Barcelona (yellow), and Okinawa–other (red) pairs of genomes.

## Results

### Pan-oceanic genome assemblies of *O. dioica*

We generated chromosome-scale genome assemblies of *O. dioica* specimens from Barcelona (BAR) and Osaka (OSA), which were added to our published assembly from an individual from Okinawa (OKI) (Table 1). We validated each reference assembly using an additional contig-level assembly chosen or generated according to sequence similarity (respectively, Bergen [BER], Aomori [AOM], and Kume [KUM]) (Table 1; Fig. 1A). The animals from Kume were cross-fertilized with the Okinawa laboratory strain, and all three pairs displayed similarity scores (see below) in the same order of magnitude. Together with geographical proximity and the results presented below, we refer to each pair of cross-validating assemblies as belonging to the same lineage or clade. We assembled the Barcelona genome using a similar procedure to the Okinawan genome, including the use of chromosome conformation information (Hi-C libraries) to aid scaffolding. A Hi-C contact map (Supplemental Fig. S1) showed that the chromosome arms and the sex-specific regions had few interactions with each other, and the assembly graph connected the sex-specific regions to the PAR's long arm through ribosomal DNA repeats. Moreover, we have constructed a new Osaka genome assembly by scaffolding the OSKA2016 assembly (Wang et al. 2020a) with long Nanopore reads that we sequenced from single individuals from the same laboratory strain. To ensure consistency (Weisman et al. 2022), we generated updated annotations for all genomes using a common automated pipeline, including repeat masking and gene prediction steps, which provide a robust set of annotations that facilitate inter-species comparisons (Table 1).

**Table 1.** Details on each of the assemblies used in this study

Name	Version	Region	Lineage name	Breeding	Length	Scaffold N50	AT-richness	Genes	BUSCO score	Technology	Level	BioSample	Reference
Okinawa	OKI2018_I69_1.0	Okinawa, Ryūkyū archipelago	Okinawa	Laboratory	64,281,565	17,092,476	59%	17,291	64%	Nanopore + Illumina + HiC	Chromosomal	SAMEA7282646	Bliznina et al. 2021
Kume	KUM-M3-7f	Kume, Ryūkyū archipelago	Okinawa	Wild	64,653,574	2,969,719	59%	16,852	66%	Nanopore	Scaffold	SAMEA111279290	This paper
Osaka	OSKA2016v1.9	Honshū, Japan inland sea	Osaka	Laboratory	56,625,162	15,521,227	58%	15,720	63%	PacBio + Illumina	Chromosomal	SAMD00227923	Wang et al. 2020a; this paper
Aomori	AOM-5-5f	Honshū, Japan, northeast Pacific coast	Osaka	Wild	56,753,784	6,419,763	59%	15,224	65%	Nanopore	Scaffold	SAMEA111279288	This paper
Barcelona	Bar2_p4	Spain, Mediterranean sea coast	Barcelona	Laboratory	55,793,437	13,545,857	60%	14,272	64%	Nanopore + Illumina + HiC	Chromosomal	SAMEA111279286	This paper
Bergen	OdB3	Norway, north Atlantic coast	Barcelona	Laboratory	70,471,451	395,387	57%	17,113	60%	Sanger	Scaffold	SAMEA2272014	Denoeud et al. 2010

(Name) Name of the genome reflecting provenance; (version) character string uniquely identifying the used version of the assembly file; (region) where the animal or the founders of its laboratory line was isolated; (breeding) wild isolate or laboratory culture line; (length) assembly sequence length in base pairs; (scaffold N50) half of the genome is covered by scaffolds of at least this length; (AT richness) a percentage of A's and T's in the sequence; (genes) number of genes in the annotation used in this study; (BUSCO score) detected percentage of the metazoan set of Benchmark Unique Single-Copy Orthologs; (technology) sequencing and assembly technologies used; (level) chromosomal or scaffold; (BioSample) identifier in the DDBJ, EBI, and NCBI databases; and (reference) peer-reviewed manuscripts describing the already published genome sequences.

### The scrambled genomes of *O. dioica*

To investigate the evolution of the chromosomes in *O. dioica*, we developed a reproducible, standardized pipeline to compute the optimal set of one-to-one local alignments in a pair of genomes using the LAST software (Frith and Kawaguchi 2015; Mitsuhashi et al. 2020) and the Nextflow workflow system (Di Tommaso et al. 2017). The all-by-all pairwise genome alignments revealed an unexpected level of genomic rearrangement, and the most extreme case of scrambling was observed in the OKI–OSA (Fig. 1B) and OKI–BAR (Supplemental Fig. S2) comparisons. The line plot comparing the whole-genome sequences of *O. dioica* from OSA and OKI revealed a striking pattern, with little to no conservation of collinear DNA segments on any chromosome (Fig. 1B). Multichromosome line plots comparing *O. dioica* from all three lineages further revealed that the genome scrambling phenomenon was common among all compared genomes (Fig. 1C). In general, the extent of genome scrambling was proportional to and increased with genetic distance (Fig. 1C). Within-lineage comparisons showed little scrambling, with large, intact collinear segments of DNA visible (Fig. 1C; Supplemental Fig. S2). These observations suggested that genome scrambling was therefore a common evolutionary characteristic in *O. dioica* genomes.

To quantify the degree of scrambling between any pair of genomes and to determine how scrambling might relate to other measures of genetic distance, we created a “scrambling index,” which measures the degree of strand randomization and, thus, the loss of collinearity between aligned regions. A scrambling index value approaching one indicates that most aligned bases have the same orientation (i.e., plus-to-plus or minus-to-minus); scrambling index values approaching zero indicate that either alignment orientation is equally frequent (i.e., plus-to-minus and vice-versa) (Fig. 1C). Computation of the scrambling index for each genome pair (Fig. 1D) yielded high values for within-lineage comparisons, allowing us to rule out technological biases introduced by different sequencing technologies (Table 1). The smallest scrambling indices were obtained for comparisons of the Okinawa lineage to other lineages (Fig. 1D). Comparisons between the Osaka and Barcelona lineages also yielded intermediate scrambling index values (near 0.2), which was congruent with the intermediate degree of scrambling observed in line plot comparisons (Fig. 1C). Each pair's scrambling index value was proportional to the proportion of variable sites among the single-copy ortholog nucleotide sequences (Fig. 1E).

### Impact of genome scrambling on macrosynteny conservation in *O. dioica*

**Figure 2.**
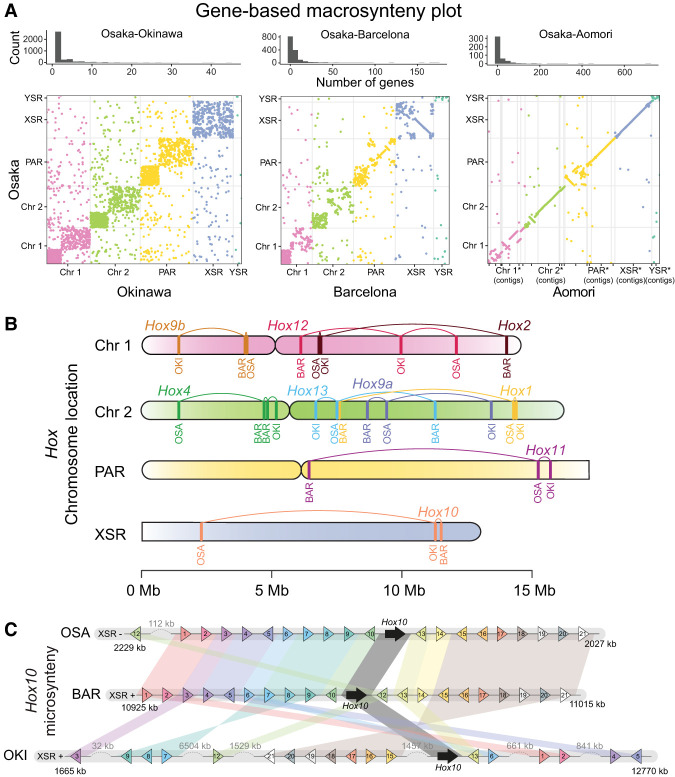
The preservation of orthologous synteny blocks gradually decreases with increasing evolutionary distance in *O. dioica*. (*A*, *top*) Histogram of the number of orthologous genes per syntenic region in pairs of genomes. (*Bottom*) Dot plots indicating the coordinates of genes belonging to the same orthogroup in pairs of genomes. (*B*) Comparative chromosome mapping of the *Hox* genes in the genomes of *O. dioica* from Osaka (OSA), Barcelona (BAR), and Okinawa (OKI). (*C*) Comparative microsynteny conservation of the block of the next 10 genes at each side of the *Hox10* genes in the same three genomes.

Line plots between Osaka and Okinawa showed that ∼94% of all rearrangements were intra-chromosomal, whereas inter-chromosomal rearrangements were rare (Fig. 1B). Within each chromosome, rearrangements tended to occur within arms or the sex-specific regions (∼99%), which for the sake of simplicity we will also refer to as “arms.” To investigate the impact of genome scrambling on the evolution of synteny blocks, we compared gene-order conservation across lineages. We computed 5162 groups of single-copy orthologs present in the six genomes, and visualized them with strand-independent macrosynteny dot plots, which showed the positions of the same gene in a pair of genomes (Fig. 2A; Supplemental Fig. S3). This confirmed that gene-order rearrangements were mostly restricted to homologous arms (Fig. 2A), and confirmed that some inter-chromosomal translocations observed at the whole-genome level involved whole-gene translocations. The number of orthologs per synteny block decreased with increasing genetic distance, with a maximum of 44 for Osaka versus Okinawa, a maximum of 174 for Osaka versus Barcelona, and a maximum of 714 for Osaka versus Aomori (Fig. 2A; Supplemental Fig. S3).

As case studies, we next mapped the chromosomal locations of several genes associated with characteristic gene clusters (*Hox*, *Fgf*, and *Myosins*) (Fig. 2B; Supplemental Figs. S4, S5). Microsynteny conservation of the *Hox* cluster has been shown to be essential for embryonic development and axial patterning in vertebrates, but the disintegration of the *Hox* cluster in *O. dioica* from Bergen suggested that it may not be essential in *O. dioica* (Seo et al. 2004). We mapped all *Hox* genes of *O. dioica* in all our assembled genomes, corroborating that the full catalog of *Hox* genes in *O. dioica* is reduced to six genes from which all central *Hox* genes (*Hox3* to *Hox*7) have been lost. Comparison of the position of *Hox* orthologs revealed that multiple changes in gene order must have occurred, although all have been maintained within the same chromosome arm. Mapping of all six Fgf genes previously reported in *O. dioica* (Oulion et al. 2012) revealed a similar pattern of gene movement (Supplemental Fig. S4). On the other hand, chromosome mapping of the eight myosin heavy chain class II genes presented a different pattern, whereby orthologs occasionally seemed to move more freely, including inter-arm and inter-chromosomal translocations (Supplemental Fig. S4). We also inspected patterns of microsynteny in these gene families by examining their 10 nearest neighboring genes up- and downstream (Fig. 2C; Supplemental Fig. S5). In general, gene families in Barcelona and Osaka showed far greater conservation of microsynteny with each other than either does with Okinawa. These examples revealed different degrees of microsynteny conservation, ranging from near-complete conservation of entire blocks (e.g., *MyhF*, *MyhG*, *Fgf11/12/13/14a*, *Fgf11/12/13/14b*, and *Hox1*) to situations in which a block has seemingly fragmented into many small pieces (e.g., *Fgf9*/*16*/*20a* and *Hox10* or *Hox12*). Based on our examination of chromosome mapping for different conserved gene families in *O. dioica*, the position of a gene in one lineage had little predictive power for the position or orientation of that gene in other lineages.

### Genome scrambling moves short functional regions

**Figure 3.**
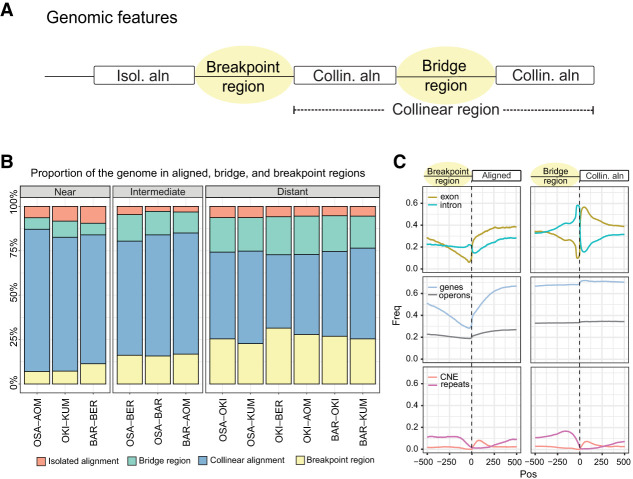
Properties of genomic alignments. (*A*) We divided the aligned and unaligned regions of the genome into four categories according to their participation in collinear regions. Collinear regions are defined as an uninterrupted succession of alignments that are on the same chromosome strand and in the same order in both genomes. (*B*) Proportion of the four categories in different alignment pairs, grouped by evolutionary distance. (*C*) Enrichment of genomic features at the boundary between breakpoint or bridge regions and aligned regions in the Okinawa–Osaka comparison (for other pairs, see Supplemental Fig. S7). (CNE) Conserved noncoding elements.

We next identified “breakpoint regions” to search for the molecular breakpoints responsible for scrambling synteny blocks. First, we identified collinear alignments, defining them as adjacent alignments in the same orientation in both genomes. We termed the regions flanked by these collinear alignments “bridge regions.” We then defined “collinear regions” as successions of collinear alignments and bridge regions. The “breakpoint regions” were therefore the remaining unaligned regions, for which there was no one-to-one correspondence in a pair of genomes, and always correspond to an interruption of collinearity. Lastly, we termed aligned regions that were not collinear to anything as “isolated alignments” (Fig. 3A). Although breakpoint regions tended to be short (0.32 ± 5.1 kbp, *n* = 8821 for the Okinawa–Osaka comparison), they covered a considerable fraction of the genome (∼23.5%) (Fig. 3B). Three reasons may explain the lack of alignability in breakpoint regions: (1) so many mutations accrued in these regions that they exceed the limits of detectable sequence similarity; (2) repeats were the target or the cause of the breaks; or (3) the mechanism involved the loss of DNA.

To determine how the phenomenon of scrambling related to functional genomic regions, we studied the frequencies of coding and conserved noncoding elements (a proxy for potential regulatory regions) (Tan et al. 2019) and repeats at the boundaries of the four nonoverlapping classes of genome segments (Fig. 3C). The alignments’ boundaries with breakpoint regions tended to coincide with exon start positions, as well as with intron stop positions to a lesser extent (Fig. 3C). Isolated alignments were less frequently part of operons. In terms of noncoding elements, repeats were depleted in isolated alignments, whereas conserved noncoding elements were enriched, with a peak downstream from the alignment start position, consistent with previously reported patterns of erosion (Royo et al. 2011). Breakpoint regions were the least likely to be found within genes. Bridge regions occurred mainly in genic regions, with strong enrichment for introns, which is consistent with the high intron turnover reported earlier (Edvardsen et al. 2004; Denoeud et al. 2010), and repeats (which may be intronic) upstream of collinear alignments; bridge regions were also most frequently associated with operons. Altogether, the most marked changes in the frequency of genomic elements between classes were related to the frequency of protein-coding features, with the exception of operons, which showed modest changes in frequency at the edges of aligned and breakpoint or bridge regions.

### Genome scrambling does not preserve operon structure

**Figure 4.**
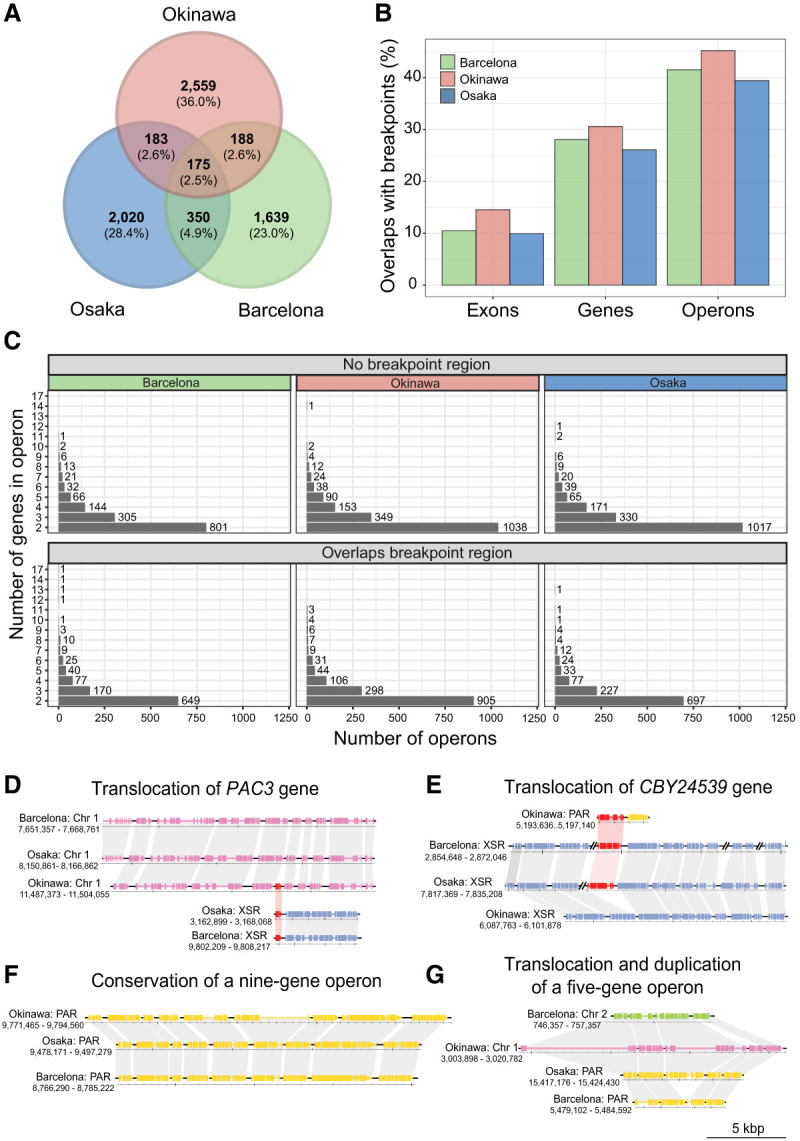
Conservation of operons in *O. dioica* lineages using the chromosome-level genomes as representatives. (*A*) Number of shared and unique operons across the chromosome assemblies representing each lineage. (*B*) The proportion of protein-coding genetic elements that overlap a breakpoint region for each genome. (*C*) Size distribution of operons that overlap or do not overlap a breakpoint region. (*D*,*E*) Translocation of the genes *PAC3* (*D*) and *CBY24539* (*E*; putative activin type I receptor), belonging to different operons in Okinawa lineages and the other lineages. (*F*) The nine-gene operon reported by Ganot et al. (2004) is conserved in Osaka, Barcelona, and Okinawa. (*G*) An example of an operon that has been translocated to different chromosomes in each species and duplicated in the Barcelona genome.

We next assessed the conservation of operons within the chromosome-scale assemblies of *O. dioica*. Operons may impose some limitations to rearrangements in synteny blocks. For example, a single-gene inversion in the middle of a three-gene operon could result in expression defects by decoupling that gene from its primary regulatory elements. The number of operons per chromosome-scale assembly ranged between 2379 and 3124, representing between 6653 and 9543 operonic genes (Fig. 4A). Only a small number of operons preserved homologous genes across Okinawa, Osaka, and Barcelona (Fig. 4A), and this finding remained true when considering alternative criteria for operon equivalence (Supplemental Fig. S6). Among protein-coding genetic elements—operons, genes, and exons—operons were the most likely to overlap breakpoint regions. In the Okinawa–Osaka genome pair, 616 out of 1281 operons overlapped a breakpoint (48%), whereas 5294 out of 17,291 genes (30%) and 16,787 out of 106,811 exons (15%) overlapped one (Fig. 4B). Further, large and small operons were both affected by scrambling (Fig. 4C). Detailed comparison of operon microsynteny revealed examples of operons with complete conservation located on the same chromosome for Okinawa, Osaka, and Barcelona (Fig. 4F). Other examples showed the conservation of an operon following a translocation of some operonic genes to a new location (Fig. 4D–E). In some cases, an operon rearrangement involved duplication and translocation of a large portion of an operon into a new chromosome (Fig. 4G). Although operons were rarely conserved between lineages in general, operonic genes from one genome were significantly more likely to be operonic in a second genome across all within-lineage pairs (*P* ≪ 0.001, chi-squared ≫ 4420.2, d.f. = 3). Overall, our data revealed an absence of strong selective constraints to strictly maintain operon structure between lineages, suggesting operons are prone to be impacted by genome scrambling.

### Genome scrambling and chromosomal evolution

**Figure 5.**
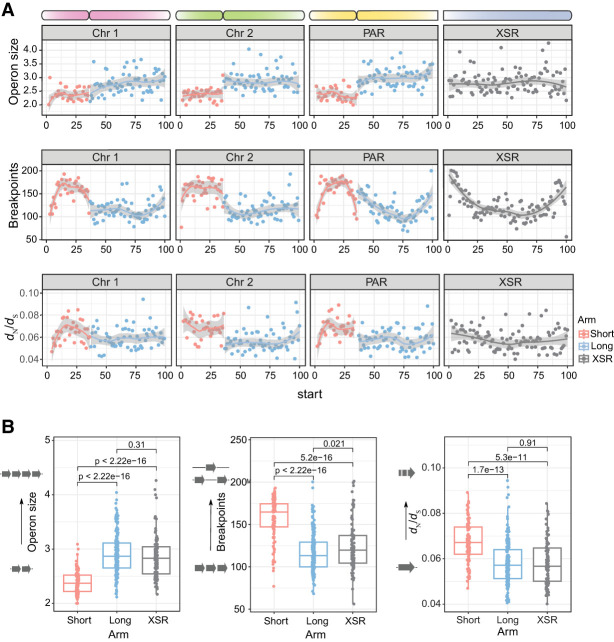
Genome-wide patterns of genomic feature density. (*A*) The mean values for various genomic features (*y*-axis) versus chromosomal location by the percentage of each chromosome's length (*x*-axis). Each bin is the average of the three chromosomal assemblies representing each lineage. Two regions of the chromosomes show characteristic differences in feature distribution: The first difference can be seen between short and long chromosome arms, and the second difference is between the centers and edges of chromosome arms. (*B*) Short and long arms show significant differences in operon size, the number of breakpoint regions, and *d*_N_/*d*_S_ ratios (Wilcoxon rank-sum test).

Given that our data pointed to chromosome arms as the primary scale of macrosynteny conservation, to better understand chromosomal evolution in *O. dioica*, we investigated the distribution of breakpoint regions, operon sizes, and mutation rate at the chromosome level (Fig. 5A–D). Our analysis revealed that short chromosome arms consistently showed four different qualities compared with long chromosome arms: (1) short arms showed a higher relative frequency of breakpoints; (2) short arms contained shorter genes and shorter operons; (3) genes on short arms overlapped breakpoint regions at a higher rate (∼50% vs. ∼20%; *P* ≪ 0.001, chi-squared ≫ 109.6, d.f. = 2); and (4) genes on short arms showed elevated *d*_N_/*d*_S_ values. In all cases, the XSR showed patterns comparable to long arms. Our analysis also revealed that these features also consistently varied across chromosome arms, differing between the centers of chromosome arms and subtelomeric or pericentromeric regions. As reported for the Okinawa genome (Bliznina et al. 2021), repeat density increased whereas gene and operon density decreased in subtelomeric and pericentromeric regions for the Osaka and Barcelona genomes (Supplemental Fig. S8). The co-occurrence in short arms of an increase in repeat content, a greater frequency of breakpoint regions, and elevated *d*_N_/*d*_S_ values together implied that repeat-related rearrangements could play a role in generating or maintaining structural variations that yielded nonrecombining loci, leading to more rapid accumulation of point mutations and substitutions.

### Evolutionary framework of the unprecedented genome scrambling in *O. dioica*

**Figure 6.**
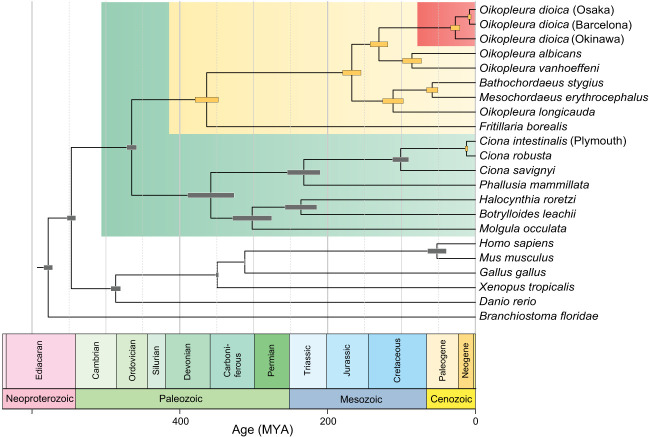
Time-scaled phylogenetic tree including several appendicularians, tunicates, and vertebrates, including *O. dioica*, based on 177 single-copy orthologous protein sequences. The different clades of *O. dioica* lineages were estimated to have shared a common ancestor ∼25 Mya (95% HPD: 12–41 Mya). The Osaka and Barcelona lineages were estimated to have diverged more recently, ∼7 Mya (95% HPD: 5–10 Mya).

To relate the rate of scrambling to evolutionary distance, we estimated a species tree and divergence times for *O. dioica* using orthologs common to chordates (Fig. 6; Supplemental Table S1). We used relatively unconstrained priors for the nodes within the appendicularians owing to a lack of supporting fossil evidence. As well, because *O. dioica* is among the fastest-evolving animals known (Berná et al. 2012), we attempted to reduce the effects of heterotachy through careful ortholog selection, curation, alignment trimming, and comparison of multiple replicates (Supplemental Table S1). The resulting phylogenetic tree supported the existence of at least three independent lineages of *O. dioica*, which were estimated to have shared a last common ancestor about 25 million years ago (Mya). This split represented the divergence between the Okinawa lineage and other lineages, and a more recent divergence time of ∼7.3 Mya was estimated for the split between the Osaka and Barcelona lineages.

**Figure 7.**
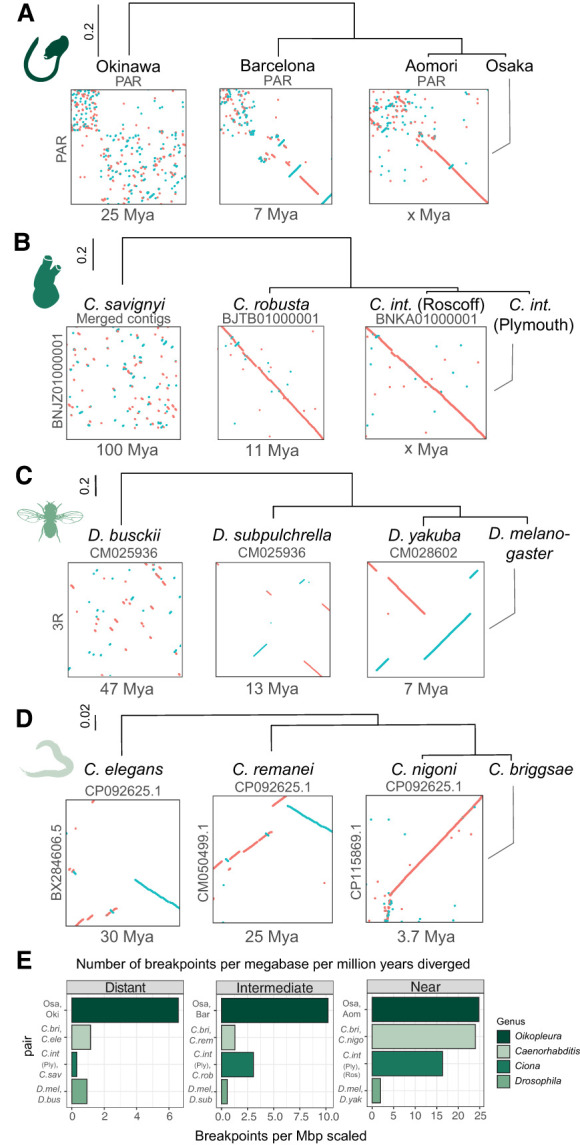
Genome scrambling in 10 × 10-Mbp regions for pairs of genomes in *O. dioica* (*A*), *Ciona* (*B*), *Drosophila* (*C*), and *Caenorhabditis* (*D*). Scaffold names are indicated in gray. (*E*) The number of breakpoint regions per megabase aligned per million years diverged for different animal clades.

Using these divergence time estimates, we calculated that the breakpoint accumulation for *O. dioica* lies between six and 25 breakpoint regions per megabase pair per million years (Fig. 7A,E). To better contextualize this result, we estimated the same value for comparisons of ascidian tunicates using two isolates of *Ciona intestinalis*, *Ciona robusta* (differing only by pigmentation) (Caputi et al. 2007) and *Ciona savignyi* (a known example of scrambling on a long divergence time of ∼100 Mya) (Fig. 7B; Satou et al. 2019), and found that breakpoint accumulation in *O. dioica* is up to an order of magnitude higher (Fig. 7E; Supplemental Fig. S9). This is also several orders of magnitude greater than the reported rate for comparisons of mammals (Damas et al. 2022); using our pipeline, the number of breakpoints between *Pan troglodytes* and *Bos taurus* yielded approximately 0.7 breakpoint regions per megabase pair per million years, based on a conservative divergence estimate of 62 Mya (Delsuc et al. 2018). A similar figure (about 0.6) was found comparing the karyotype-derived muntjac deer *Muntiacus muntjak* to its close relative *Muntiacus reevesi* (∼5 Mya divergence) (Supplemental Fig. S1, line plot; Mudd et al. 2020). Further, to relate our results to other invertebrates with short generation times, we also computed these values for near, intermediate, and distantly related species of *Drosophila*, in which scrambling was reported earlier (Fig. 7C; Suvorov et al. 2022), and *Caenorhabditis*, which also contain *trans*-spliced operons (Fig. 7D). Importantly, between the effects of heterotachy, the potential for ortholog misidentification, and misalignment, the divergence time estimates for the splits between the *O. dioica* lineages were more likely to be overestimated than underestimated, in which case the rate of chromosomal rearrangements would be even greater than we have computed. In conclusion, based on these metrics, all *O. dioica* lineages showed a distinctly greater rate of scrambling than any other group of animals (Fig. 7E).

## Discussion

### Genome scrambling and speciation

Our study design, combined with the recent divergence times estimated for *O. dioica*, allowed us to study genome scrambling on a finer timescale than was reached by previous studies. Despite the relatively small evolutionary distances between the *O. dioica* lineages used here, we discovered thousands of breakpoints, which may be an order of magnitude higher than other ascidian tunicates or flies with similar divergence times and may be more comparable to species pairs that diverged hundreds of millions of years ago (Fig. 7; *Drosophila* 12 Genomes Consortium 2007; Hane et al. 2011; Albertin et al. 2022; Damas et al. 2022). The phylogeny we estimated suggests that the three lineages (KUM + OKI, AOM + OSA, BAR + BER) may correspond to three distinct cryptic species, which was corroborated by our analyses of marker genes (Masunaga et al. 2022), but we ask the reader to refrain from using their names as species names to let the taxonomical discussion proceed to an optimal solution. Our molecular clock analysis suggests that the Osaka and Barcelona lineages shared a common ancestor ∼7 Mya, which diverged from the Okinawa lineage >20 Mya. The genetic and environmental factors that might have contributed to cryptic speciation in this clade are unknown. It is tempting to speculate that the extreme rate of rearrangement in *O. dioica* could accelerate sympatric speciation through the formation of reproductively incompatible subpopulations within an area, even in marine environments lacking physical geographic boundaries. Further extensive sequencing of *O. dioica* genomes around the globe and surveys of intrapopulation genetic variation are needed to validate this hypothesis. Sampling other appendicularian species will be necessary to explore if genome scrambling is present beyond *O. dioica*, as it could be a hallmark of the evolution of appendicularian genomes, perhaps related to the high rate of gene loss in the clade (Ferrández-Roldán et al. 2021).

The case of *O. dioica* has two qualities that run contrary to typical biological intuitions: The similarity of morphology would not have predicted significant differences in genome structure, and the significant differences of genome structure would not have predicted similarity of morphology. As such, it shows that genetic distances and even taxonomic ranks may be insufficient to predict the amount of information that could be gained by sequencing a given organism's genome, which is particularly relevant for large-scale genome sequencing projects. Conversely, it provides a clear example of a case in which substantial differences in genome structure do not result in easily determined distinguishing characteristics (synapomorphies) that could be useful for taxonomic purposes. Although *O. dioica* may represent a unique challenge (and opportunity) for taxonomists and evolutionary biologists, we believe that difficulties in understanding the relationship between genome conservation and what defines a biological species will become increasingly common in the postgenomic era, as clades across the tree of life continue to be sequenced. Thus, our results serve as a reminder that translating results between different scientific fields cannot solely rely on raw data but requires interdisciplinary cooperation and expertise.

### Mechanisms of genome scrambling and impact on gene regulation

Between the high divergence times between the *O. dioica* lineages, the elevated rate of evolution in *O. dioica*, and the complex nature of the identified rearrangements, we were unable to identify precise molecular breakpoints that could be explained by a simple and specific genetic mechanism. Future comparisons between less distant *O. dioica* lineages, or even within populations, might help us to better understand the mechanisms responsible for this massive genome scrambling. It is tempting, though, to speculate that the loss of the canonical nonhomologous end-joining (NHEJ) DNA repair pathway in *O. dioica* might have created synergies that act to promote scrambling. For instance, the alternative microhomology-based pathway (MMEJ), which was shown to be active in experimentally induced lesions in *O. dioica* (Deng et al. 2018), is slower than other repair mechanisms (Fu et al. 2021), which might allow for greater chromatin movement to occur before the repair of a double-stranded break. Cut-and-paste transposons that use the MMEJ pathway may also act as a source of microhomologies that could facilitate repair by MMEJ. The low repeat content of *O. dioica* genomes might therefore be a reflection of genomic instability that also causes scrambling. Although *O. dioica* genomes seem to be repeat-sparse, a relatively small number of interspersed repeats is sufficient to facilitate rearrangements through repair mechanisms such as homologous recombination. Scrambling in *O. dioica* seems to correlate with phylogenetic distance and divergence time. Parsimoniously, the mechanisms underlying scrambling are more likely to involve the gradual and ongoing accumulation of rearrangements rather than the result of one or more dramatic lineage-specific rearrangement events.

The genome of *O. dioica* is not as well annotated as those of humans or mice, and the significant genomic rearrangements between different lineages of *O. dioica* complicate the comparison of epigenomic or transcriptomic data across these lineages. Consequently, our knowledge of elements like enhancers, promoters, or topologically associating domains (TADs) and their similarities across lineages is too limited for practical use in current research. This issue is being addressed in ongoing projects that are generating data from various laboratory strains concurrently. Despite these challenges, operons can still be inferred through the proximity of predicted coding sequences, allowing their use in this study.

The operon structures that control the transcription of neighboring genes in *O. dioica* are rarely identically conserved between lineages. Two properties of *O. dioica* operons could be related to this observation: (1) the expression levels of operonic genes are not strongly correlated (Danks et al. 2015), and (2) the functional categories of operonic genes are not necessarily correlated. Together, these observations suggest that the operon structure in *O. dioica* need not solely or primarily be related to the regulation of transcription. On the contrary, the presence of the operon transcriptional system could act to decrease the necessity for genes to retain their own promoters, by allowing them to freely insert into other operons with their own transcriptional machinery. Indeed, we identified several lines of evidence suggesting that operon-switching can occur. As such, an operon system such as exists in *O. dioica* might in fact help to maintain gene expression in the context of genome scrambling. Although operons may facilitate genome scrambling, they may not directly cause scrambling; operons are found in the short-lived nematode *Caenorhabditis elegans* (Blumenthal and Gleason 2003) without a marked difference in the rate of scrambling compared with *Drosophila*. The fact that *O. dioica* operons scramble underlines the profound difference between bacterial operons and eukaryotic operons that use *trans*-splicing and calls for further investigations in *O. dioica* to better understand how genome scrambling can affect the regulation and evolutionary dynamics of operons in eukaryotes.

In conclusion, our results reveal an unprecedented degree of genome scrambling among what was considered a single cosmopolitan *O. dioica* species but which, according to our findings, may represent multiple cryptic species around the globe. In contrast to lepidopterans, in which speciation is intimately linked to highly dynamic evolution in the number of chromosomes (Joron et al. 2011; Hill et al. 2019; de Vos et al. 2020), the karyotype of *O. dioica* remains constant between lineages. Despite massive genome scrambling that drastically changes gene order and disintegrates microsynteny, the lineages that we studied do not show obvious morphological differences (Masunaga et al. 2022); they share similar ecological niches throughout the world; and laboratory cultures seeded by local samples are used as an animal model internationally without previously noticing physiological differences (Bouquet et al. 2009; Martí-Solans et al. 2015; Masunaga et al. 2020). This apparent uncoupling of conservation of morphology from conservation of genome structure—perhaps the first such example of this phenomenon among animals, and certainly among the chordates—has important implications for investigating genotype–phenotype relationships in other species.

## Methods

### Sampling, genome sequencing, genome assembly, and scaffolding

We extracted high-molecular-weight DNA from one individual (“Bar2”) from the Barcelona laboratory strain (Martí-Solans et al. 2015) using a modified salting-out protocol (Masunaga et al. 2022), sequenced it on MinION sequencer Mk1B (Oxford Nanopore Technologies [ONT]) using a SQK-LSK109 kit (ONT) following the manufacturer's instructions and base-called it with the Guppy software (ONT) version 4.4.2 using the Rerio model res_dna_r941_min_crf_v031 (https://github.com/nanoporetech/rerio). The shortest reads were discarded until remaining data reached 7 × 10^9^ nt, using filtlong software (https://github.com/rrwick/Filtlong), resulting in a read N50 >30,000 nt. We then assembled the genome using Flye software (Kolmogorov et al. 2019) version 2.8.2-b1689 with the ‐‐min-overlap 10,000 parameter using a custom Nextflow pipeline (see Data access). To ensure one-to-one correspondence between assemblies, we removed alternative haplotype sequences using the purge_dups tool (Guan et al. 2020). However, as a single removal step was not efficient enough, we used an iterative approach in which haplotigs were flagged with purge_dups, reads were aligned uniquely to contigs with LAST and last-split, and then reads aligning to purged haplotigs were removed before restarting the whole assembly process. Iterations were stopped after purge_dups stopped discovering haplotigs, and an assembly was selected that provided the best tradeoff between contiguity (which typically increased during the first iterations) and a low number of duplicated single-copy orthologs (Supplemental Table S2). The contigs were then polished with Pilon 1.22 (Walker et al. 2014) using short-read sequences from the same individual (available from the NCBI BioProject [https://www.ncbi.nlm.nih.gov/bioproject/] under accession number PRJEB55052) and scaffolded using Hi-C data from the Bergen line at tailbud stage (available from the NCBI Sequence Read Archive [SRA; https://www.ncbi.nlm.nih.gov/sra] under accession number SRR14470734) using Juicer (Durand et al. 2016) and 3D-DNA (Dudchenko et al. 2017), as in the work of Bliznina et al. (2021). The correctness of the scaffolding was later assessed using the same tools with Hi-C data from the Barcelona line itself (Supplemental Fig. S1).

We sequenced the Kume and Aomori genomes using single animals isolated from wild populations (Masunaga et al. 2020) with the same method except that we base-called with Guppy version 5.0.11 and the Guppy model dna_r9.4.1_450 bps_sup and used Flye version 2.8.3-b1763 with the parameters ‐‐min-overlap 10,000 ‐‐extra-params assemble_ovlp_divergence = 0.04,repeat_graph_ovlp_divergence = 0.04,read_align_ovlp_divergence = 0.04,max_bubble_length = 800,000,use_minimizers = 1,minimizer_window = 5, and no scaffolding no polishing was performed.

We rescaffolded the OSKA2016 genome (Wang et al. 2020a) by merging scaffolds that were overlapped by long contigs from independent single individual genome draft Nanopore long-read assemblies from the same laboratory strain (SRA: SAMEA6864573 and BioProject: PRJEB55052). As a last resort, we arbitrarily merged some contigs to a chromosome arm based on synteny information. The resulting OSKA2016v1.9 assembly is described in more detail at GitHub (https://github.com/oist/LuscombeU_OSKA2016_rescaffolding).

We sequenced genomes exclusively from male animals because they simplify the assembly of the sex-specific regions, which are single copy in males.

For all genomes, we counted metazoan near-universal single-copy genes using the benchmarking universal single-copy orthologs (BUSCO) (Manni et al. 2021) tool version 5.2.1 and an AUGUSTUS model trained for annotating the OKI2018_I69_1.0 assembly (Hoff and Stanke 2019; Bliznina et al. 2021). Although this version of BUSCO appears to have a lower detection baseline compared with the v3 series that we used for the OKI2018_I69 genome assembly (64% vs. 73%) (Bliznina et al. 2021), the completeness of our new assemblies is consistent with the score of the OKI2018_I69 genome assembly for which we have previously shown high completeness (Bliznina et al. 2021). Finally, we removed unplaced scaffolds from all chromosomal assemblies.

### Pairwise genome alignment and comparison

We aligned pairs of genomes using the same approach as previously described (Bliznina et al. 2021). In brief, we used the LAST software (Kiełbasa et al. 2011) to align a “query” genome to a “target” genome indexed with the YASS seed (Noé and Kucherov 2005) for long and weak similarities with parameters and a scoring matrix determined by LAST-TRAIN software (Hamada et al. 2017); filtered the resulting many-to-many set of alignment pairs with the last-split tool (Frith and Kawaguchi 2015), which searches for an optimal set of one-to-one local alignments; and finally removed alignments that include a significant amount of masked sequences with the last-postmask tool (Frith 2011). Our one-to-one alignments share some features with the “chains” of Kent et al. (2003) but do not allow local inversions.

We parallelized this process in a Nextflow (Di Tommaso et al. 2017) workflow available at GitHub (https://github.com/oist/plessy_pairwiseGenomeComparison/tree/v5.1.0). To load the alignment coordinates in the R environment for statistical computing (R Core Team 2023), we wrote a package called GenomicBreaks (https://oist.github.io/GenomicBreaks/) using core Bioconductor libraries (Lawrence et al. 2013). In this package, the strand_randomisation_index function computes for each chromosome the absolute difference of the total length of opposite-strand alignments to the total length of same-strand alignments and divides the result by the total length of the aligned regions so that a value of one indicates that all alignments are same-strand, and a value of zero indicates that, overall, the orientations appear to be random. The average of the values obtained on each chromosome is then computed and weighted by the length of the chromosomes. For the computation of the breakpoint and bridge regions, we used the strictest definition of collinearity, in which it is interrupted by inversions (changes of alignment strand) and translocations (presence of one extra aligned region in one genome only) of any length. A copy of the software and the alignment files is archived (see Data access). A rendering of the R vignettes that we used to produce these visualizations is available at GitHub (https://oist.github.io/LuscombeU_OikScrambling/) and Zenodo (doi:10.5281/zenodo.10677221) and compiled as an interactive notebook of vignettes in Supplemental Data.

Pairwise comparison between the *O. dioica* genomes produced in this work and the *Oikopleura vanhoeffeni*, *Oikopleura longicauda*, and *Oikopleura albicans* genomes (Naville et al. 2019) were loaded in the CNEr package (Tan et al. 2019) to define conserved noncoding elements with a window size of 50 and an identity threshold of 48 (see Data access).

### Repeat masking and gene annotation

For each genome, a custom library of repeats was created by merging outputs of three different software—RepeatModeler (Flynn et al. 2020) version 2.0.1, MITE-Hunter (Han and Wessler 2010) version 11–2011, and SINE_Finder (Wenke et al. 2011)—that were used as input for RepeatMasker (Smit et al. 2013) version 4.1.0. The repeats identified by homology searches were soft-masked in each assembly.

Gene models were predicted using AUGUSTUS (Stanke et al. 2006) v3.3.3 using the species model trained for OKI2018_I69 *O. dioica* (Bliznina et al. 2021). To produce more accurate annotations, transcripts aligned to genomes with BLAT (Kent 2002) version 36 were used as “hints.” In cases in which an assembled transcriptome was not available, data from related individuals were used. In particular, the transcriptome assembly generated by Wang et al. (2015) was used for predicting genes in both OSKA2016v1.9 and AOM-5-5f genomes, whereas the transcriptome assembly generated by Bliznina et al. (2021) was used for reannotation of the OKI2018_I69 genome and annotation of the KUM-M3-7f genome. A Barcelona transcriptome assembly was used for gene prediction in the Bar2_p4 genome. The parameter “‐‐allow_hinted_splicesites” was used with AUGUSTUS to allow the prediction of noncanonical splice sites (GAAG, GCAG, GGAG, GTCG, GTAA).

Operons were annotated for each species, defining an operon as a set of genes that follow each other on the same strand and are separated by an intergenic distance of at most 500 bp, as this definition produces distributions of operon lengths comparable to the one reported by Denoeud et al. (2010). For the operon conservation analysis in Figure 4, two kinds of gene equivalence were considered: genes equivalent by assignment to the same hierarchical orthogroup (“HOG”) (see Supplemental Fig. S6A–C) or genes equivalent by assignment to the same orthogroup (OG) (Supplemental Fig. S6D–F) using OrthoFinder. Because HOGs can include one-to-one orthologs as well as paralogs, it is relatively permissive to regard genes in HOGs as equivalent; however, assignment to the same OG is even more permissive, as OGs often contain many HOGs and may represent entire gene families. Operon equivalence was also assessed in two ways: Operons considered equivalent when all genes of an operon from species 1 were equivalent to all genes of an operon from species 2 (exact) (Supplemental Fig. S6A,B,D–E). A second type of operon equivalence allowed for up to one gene to differ in operons of length three or greater (inexact) (Supplemental Fig. S6C,F). This means an operon consisting of genes ABC in species 1 would match to any/all operons containing the genes ABC, XBC, AXC, or ABX in species 2. Overall, the conclusion that operons are not conserved between species of *O. dioica* is unaffected by the intergenic distance used to define operons (Supplemental Fig. 6, cf. A and B, cf. D and E), gene equivalence criteria (A vs. D, B vs. E), or operon equivalence criteria (Supplemental Fig. 6, cf. B and C, cf. E and F).

### Ortholog identification

Gene orthology was reconstructed using OrthoFinder (Emms and Kelly 2015, 2019) version 2.5.4 based on 26 proteomes spanning three subphylums of chordates. To improve orthology assignment within *O. dioica*, multiple tunicate species were included as recommended in the OrthoFinder tutorials (https://davidemms.github.io/). Gene predictions for six appendicularian genomes from Naville et al. (2019) and two geographically distinct *C. intestinalis* genomes (Plymouth and Roscoff) (Satou et al. 2021) were computed using a similar approach to *O. dioica*, including repeat-masking followed by gene prediction with AUGUSTUS version 3.3.3. Gene prediction used either the *O. dioica* or *Ciona* model, as other species lack publicly available gene annotations. The proteomes of other species were downloaded from UniProt: *Branchiostoma floridae* (UP000001554), *C. intestinalis* type “A” (*robusta*, UP000008144), *C. savignyi* (UP000007875), *Danio rerio* (UP000000437), *Xenopus tropicalis* (UP000008143), *Gallus gallus* (UP000000539), *Mus musculus* (UP000000589), and *Homo sapiens* (UP000005640). Four more tunicate species were included from the Aniseed database: *Botrylloides leachii*, *Halocynthia roretzi*, *Molgula oculata*, and *Phallusia mammillata*. To remove redundancy in the data set, protein sequences were clustered at 100% identity using CD-HIT (Li and Godzik 2006) version 4.8.1. Alternative haplotypes were removed from the Bergen *O. dioica* proteome, and only the longest isoforms per gene were used for the analysis. OrthoFinder was run with the parameters -M msa -T raxml-ng with the following fixed species tree to ensure that *O. dioica* sequences fall within the oikopleurid branch:

(((*Danio*_*rerio*,(*Xenopus*_*tropicalis*,((*Mus*_*musculus*,*Homo*_*sapiens*),*Gallus*_*gallus*))),(((*Molgula*_*oculata*,(*Halocynthia*_*roretzi*,*Botrylloides*_*leachii*)),((*Ciona*_*savignyi*,((*C*_*intesinalis*_*P*,*C*_*intestinalis*_*R*),*Ciona*_*robusta*)),*Phallusia*_*mammillata*)),(*Fritillaria*_*borealis*,((*Oikopleura*_*longicauda*,(*Mesochordaeus*_*erythrocephalus*,*Bathochordaeus*_sp)),((*Oikopleura*_*vanhoeffeni*,*Oikopleura*_*albicans*),((KUM-M3-7f,OKI2018_I69),((Bar2_p4,OdB3),(AOM-5-5f,OSKA2016v1.9)))))))),*Branchiostoma*_*floridae*).

The Hox protein sequences of the Bergen genome were used as reference Hox sequences for *O. dioica* (Seo et al. 2004). In general, Hox genes were assigned appropriate orthogroups by OrthoFinder, although Hox11 could not be identified within the Barcelona proteome and the *Hox9* model for Osaka had not been spliced appropriately. Regardless, the identities of these genes were confirmed by alignment with the Bergen sequence as well as multiple sequence alignment with all orthologous family members followed by tree estimation with IQ-TREE (Nguyen et al. 2015) version 1.6.12.

### Phylogenomics and divergence time estimation

A species tree for *O. dioica* was estimated using a concatenated alignment of 5162 single-copy orthologous nucleotide sequences common to all six *O. dioica* genomes. A maximum likelihood tree was estimated with RAxML (Stamatakis 2014) version 8.2.4 using the GTRCAT substitution model and the autoMRE bootstopping criterion. The same data were used to estimate a Bayesian tree using MrBayes 3.2.7 (Ronquist et al. 2012) with six gamma-distributed rate categories, the 4-×4-nt substitution model. The MCMC chain was computed with three runs, a maximum of 100,000,000 generations, and 25% burn-in, with automatic stoppage after the average standard deviation of the split frequencies was lower than 0.01 and a minimum split frequency of 0.10. For both maximum likelihood and Bayesian analyses, each ortholog was assigned a separate independent partition.

To estimate the divergence times of *O. dioica* lineages, we created a stringent and conservative set of single-copy orthologous protein sequences in accordance with recommended practices in phylogenomics (Philippe et al. 2017; Simion et al. 2020), acknowledging that heterotachy is particularly problematic in the case of *O. dioica* (Berná et al. 2012) and the difficulty of retrieving accurate ortholog sequences from larvacean genomes of variable completeness and contiguity. A set of single-copy orthologous protein sequences was extracted from the results of OrthoFinder, selecting proteins that were shared by 10 or more of the 26 species, yielding 555 ortholog candidates. Each candidate ortholog was aligned using PRANK (Löytynoja 2014) v.170427 and then trimmed with HmmCleaner (Di Franco et al. 2019), and a gene tree was estimated with RAxML (Stamatakis 2014) version 8.2.4, with 100 rapid bootstraps and a gamma model of rate heterogeneity with automatic model selection using PROTGAMMAAUTO. Each gene tree was compared with the later species tree with the ete3 toolkit and evaluated for congruence (Huerta-Cepas et al. 2016). A supermatrix (concatenated alignment) was constructed, and gene information content was assessed with MARE (https://bonn.leibniz-lib.de/en/research/research-centres-and-groups/mare) v0.1.2-rc, which reduced the number of orthologs to 177. The alignment supermatrix generated from these 177 genes (containing 60,630 aligned amino acid sites, including gaps) was used to estimate a species tree with RAxML using 100 rapid bootstrap replicates, the gamma model of rate heterogeneity, and automatic model selection for each gene as separate partitions. To estimate divergence times, BEAST1 (Suchard et al. 2018) v1.10.4 was used with the BEAGLE library (Ayres et al. 2012) with the following parameters: the birth–death tree density model (Gernhard 2008), a linked random local clock model (Drummond and Suchard 2010), an unlinked gamma-distributed rate heterogeneity with four categories for each partitioned gene, and the CTMC scale reference prior model (Ferreira and Suchard 2008). To estimate only divergence times, the tree topology was fixed to the species tree estimated by RAxML. Where possible, the divergence time estimates published by Delsuc et al. (2018) using their LN CAT-GTR + Γ_4_ model were used as normally distributed priors on our tree with matching mean and standard deviation. Each node that did not correspond between the two studies, including the appendicularian proteomes that we annotated, uses uniformly distributed priors with a maximum age as the age of the tunicates, owing to a lack of suitable fossils to calibrate these nodes. The only exception was a normally distributed prior for the split between *C. intestinalis* and *C. robusta*, which used the value reported by Bouchemousse et al. (2016). To ensure the models had converged, Tracer (Rambaut et al. 2018) was used (v1.7.2), and further, three replicate analyses were performed using these parameters, taking the last 100 million steps after convergence for calculating statistics. The final resampled, combined metrics are reported in Supplemental Table S1. The maximum clade credibility tree with node heights summarized to the median is depicted in Figure 5, using the replicate with the best marginal likelihood estimated by generalized stepping-stone sampling. The R libraries ggtree (Yu 2020) version 3.2.1, treeio (Wang et al. 2020b) version 1.18.1, and deeptime (Hoffmann et al. 2022) version 0.2.2 were used for tree visualization.

### *d*_N_/*d*_S_ estimation

To generate *d*_N_/*d*_S_ estimates for *O. dioica* genes, single-copy orthologous proteins common to all six *O. dioica* proteomes were assessed. Each orthologous protein was aligned using PRANK, and protein alignments were converted to codon alignments using PAL2NAL (Suyama et al. 2006) v14.1. Then, a global estimate for *d*_N_/*d*_S_ was calculated using the CODEML program of the PAML package (Yang 1997, 2007) version 4.9j using the species tree estimated from all single-copy orthologs as the tree input file, as well as the FMutSel mutation-selection model (codonfreq = 7). Estimating a single *d*_N_/*d*_S_ value for a gene family, irrespective of differences between sites or branches, is almost certain to underestimate *d*_N_/*d*_S_; although this is less powerful for identifying cases of positive selection, it is nonetheless suitable for roughly characterizing substitution patterns across genome as used in Figure 4. To support the estimates produced from global comparisons, maximum likelihood and Bayesian estimates for *d*_S_, *d*_N_, and *d*_N_/*d*_S_ were also calculated for all pairs (using runmode = −2 and runmode = −3) and are depicted in Supplemental Figure S10, providing support for the relatively low *d*_N_/*d*_S_ values reported by global estimates.

## Data access

Raw Nanopore reads generated in this study have been submitted to the NCBI BioProject database (https://www.ncbi.nlm.nih.gov/bioproject/) under accession number PRJEB55052. Software, alignments, and intermediate data are available at Zenodo (https://doi.org/10.5281/zenodo.10241527) and as Supplemental Code and Supplemental Data.

## Supplementary Material

Supplement 1

Supplement 2

Supplement 3

Supplement 4

Supplement 5
