# Supplementary material for: Extreme genome scrambling in marine planktonic Oikopleura dioica cryptic species
Source: Genome Res. 2024 Mar;34(3):426–40. doi: 10.1101/gr.278295.123 (PMC11067885; doi:10.1101/gr.278295.123)

Supplemental Figure S1: Contact map of a Hi-C library made from the Barcelona laboratory strain, aligned on the Barcelona chromosomal assembly.

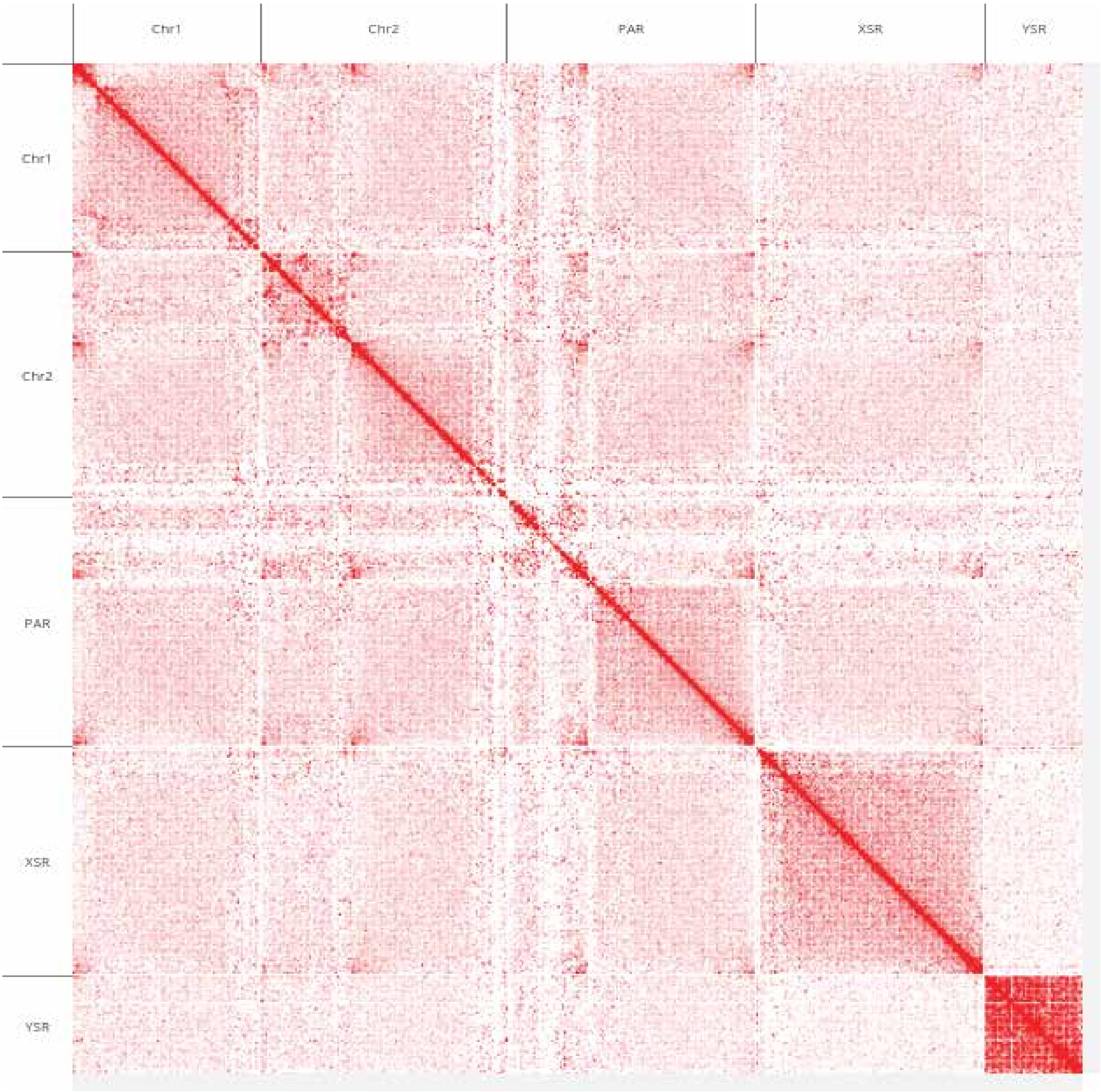

Supplemental Figure S2: Line plot representation of pairwise whole-genome alignments.
Red: +/+ alignment. Blue: +/- alignment.

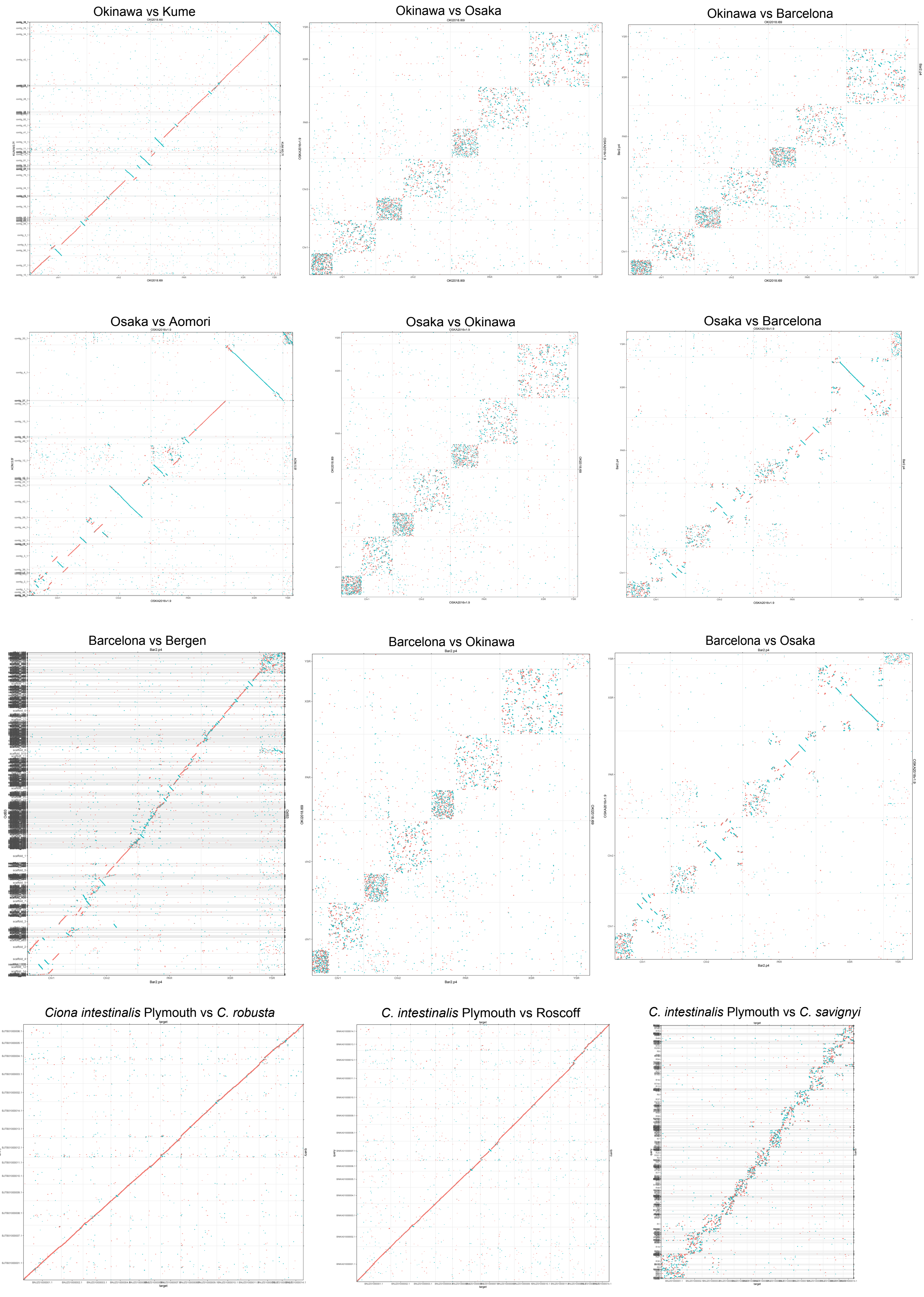

Supplemental Figure S2 (continued)

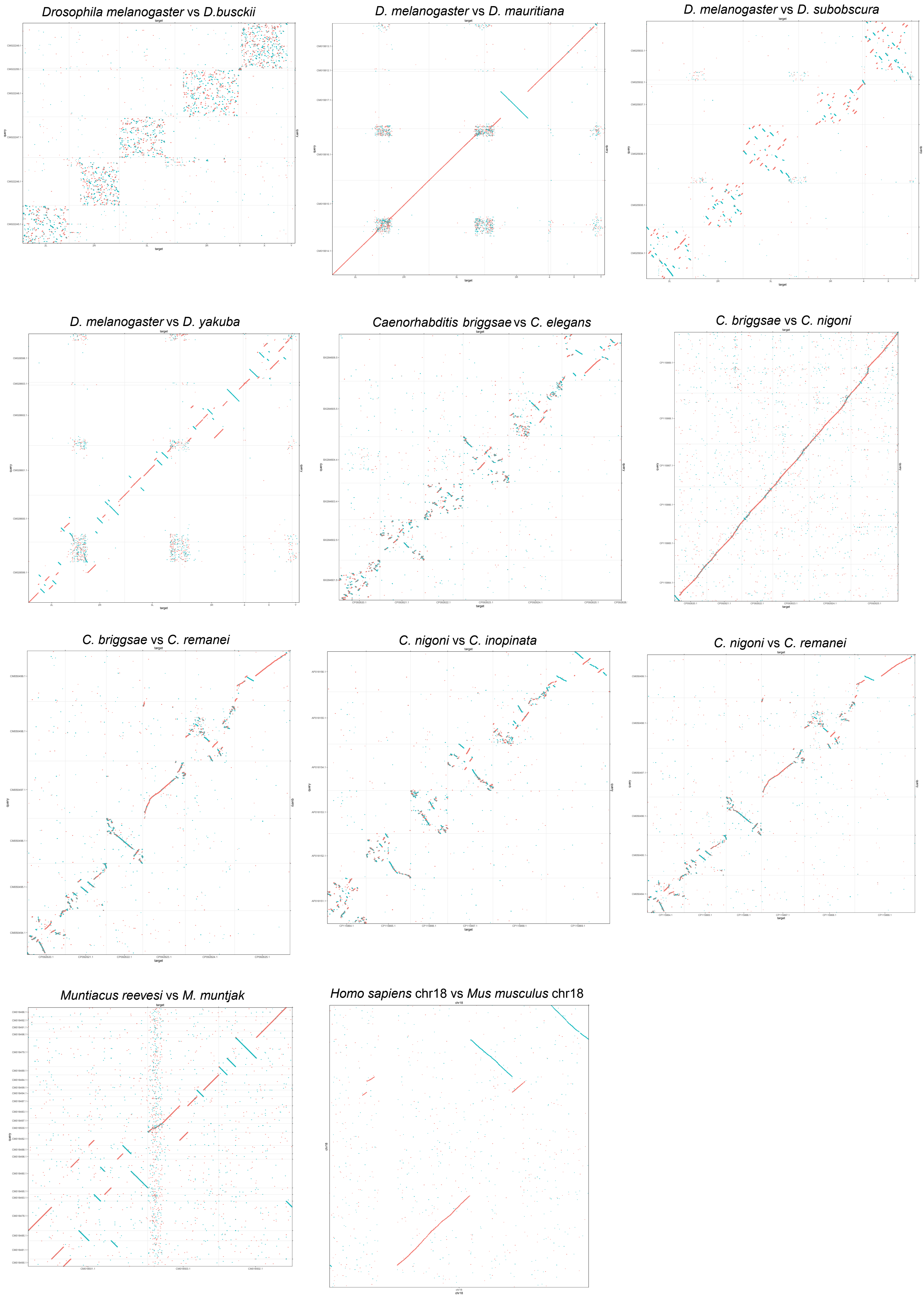

Supplemental Figure S3: Histogram of the number of orthologous genes per syntenic region in pairs of genomes (horizontal axis in natural or log scale), followed by dot-plot plots of homologous genes in pairs of genomes not displayed in the main Figure 2.

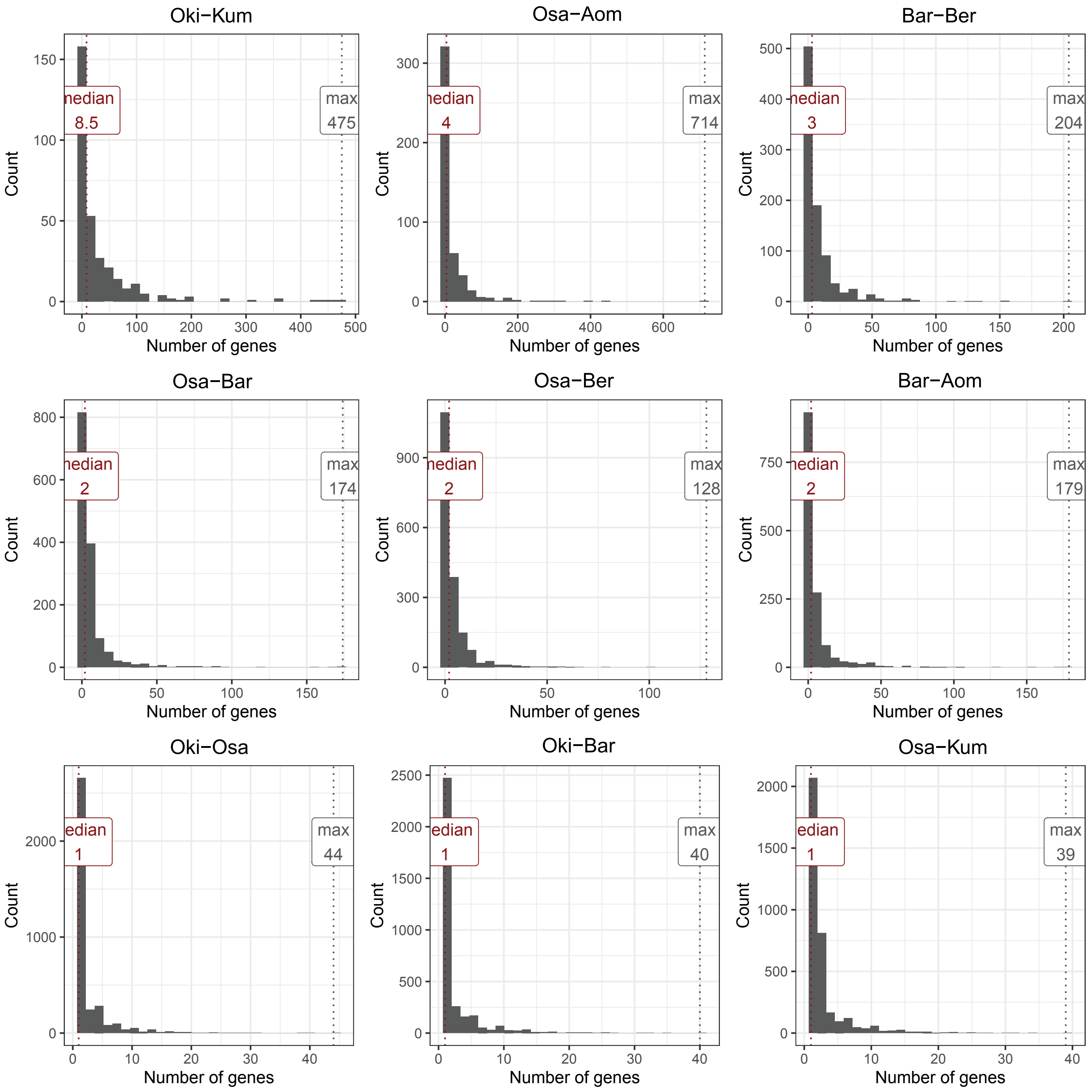

Supplemental Figure S3 (continued)

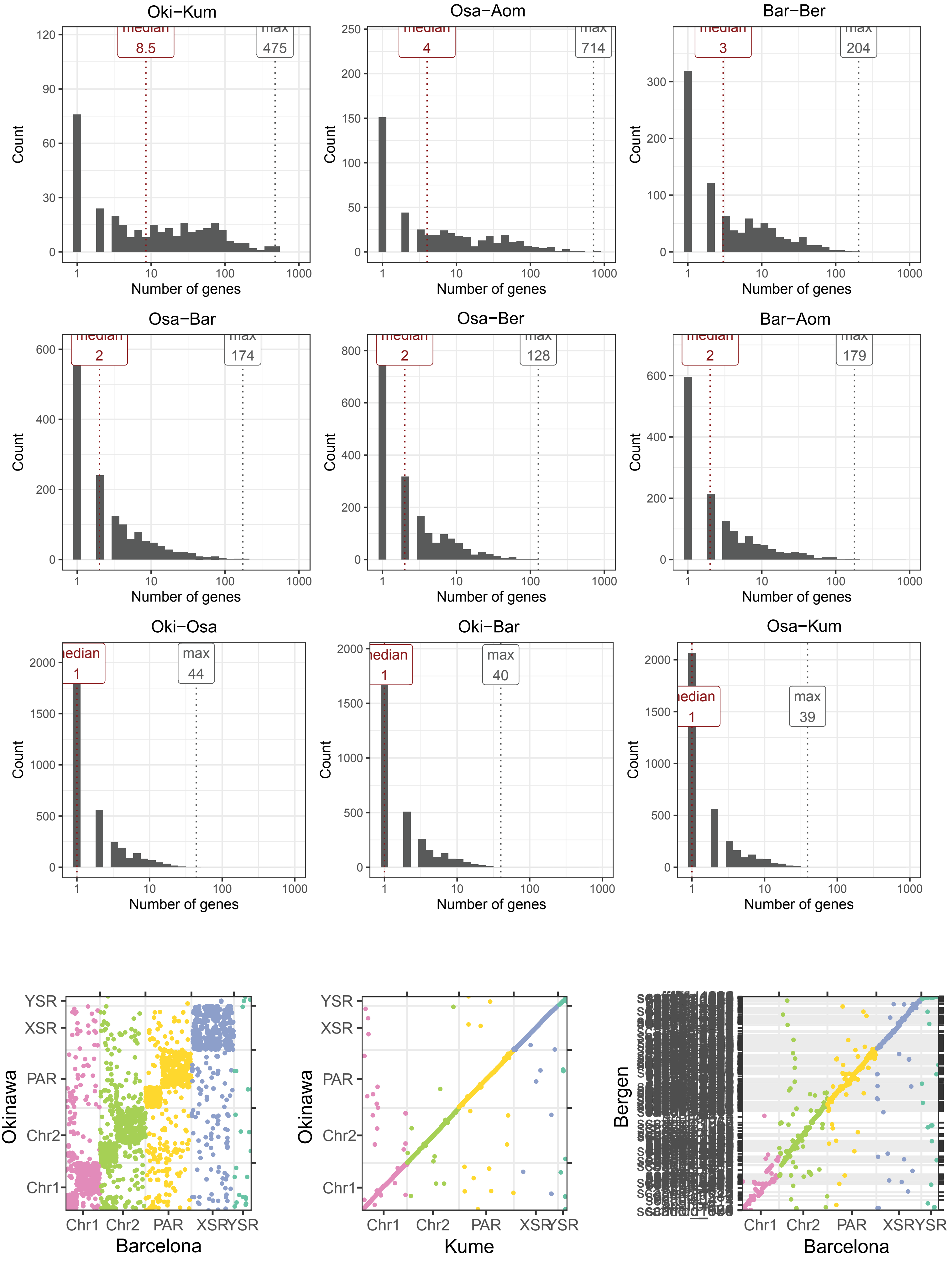

Supplemental Figure S4: Comparative chromosome mapping of the Fgf (A) and Myh genes in the genomes of *O. dioica* from Osaka (OSA), Barcelona (BAR) and Okinawa (OKI).

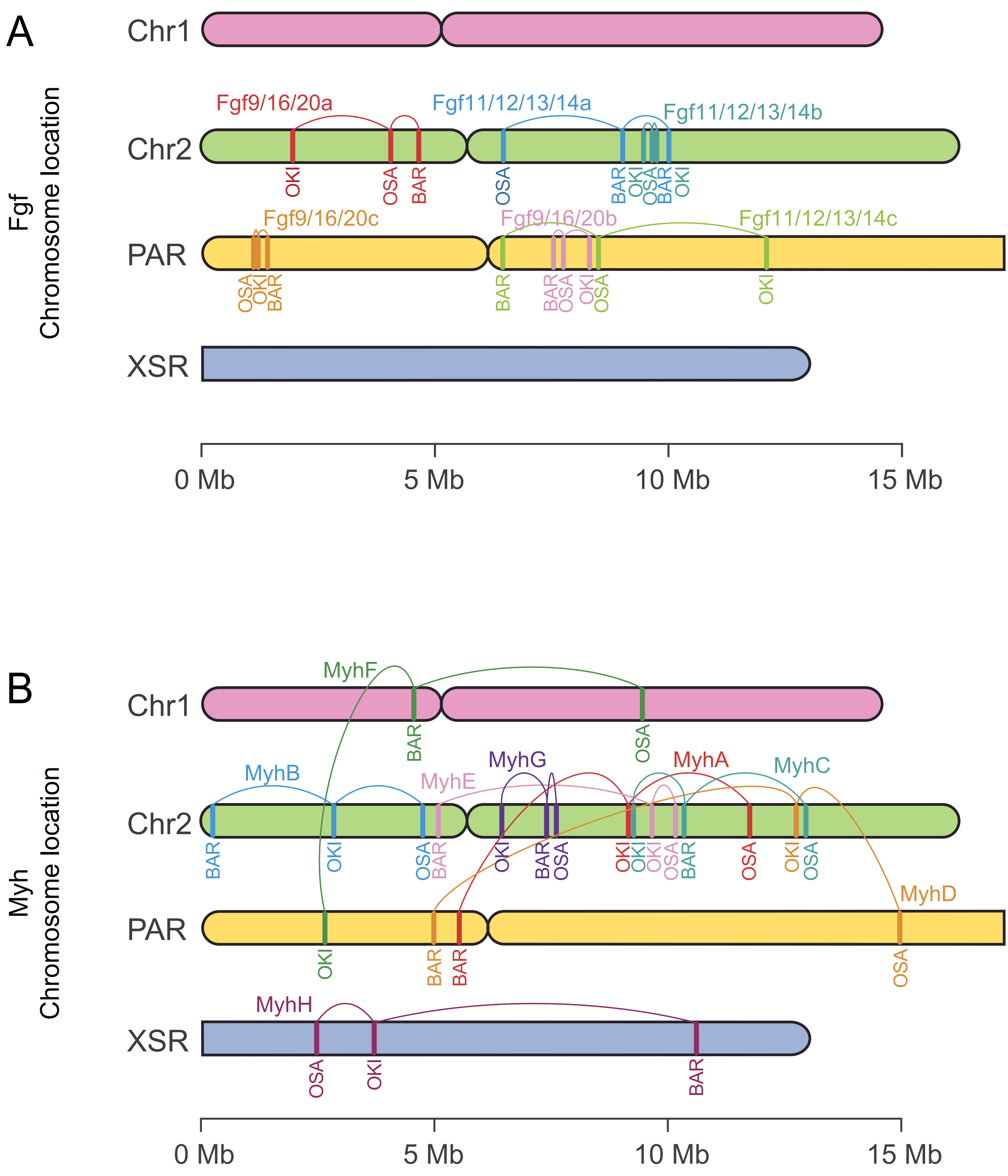

Supplemental Figure S5: Comparative microsynteny analysis of loci surrounding Fgf and Myosin gene family members in *O. dioica*. A: Fgf11/12/13/14a; B: Fgf11/12/13/14b; C: Fgf9/16/20) and Myh (D: MyhG; E: MyhA; F: MyhF). Species names are shortened as follows: Osaka (OSA), Barcelona (BAR) and Okinawa (OKI).

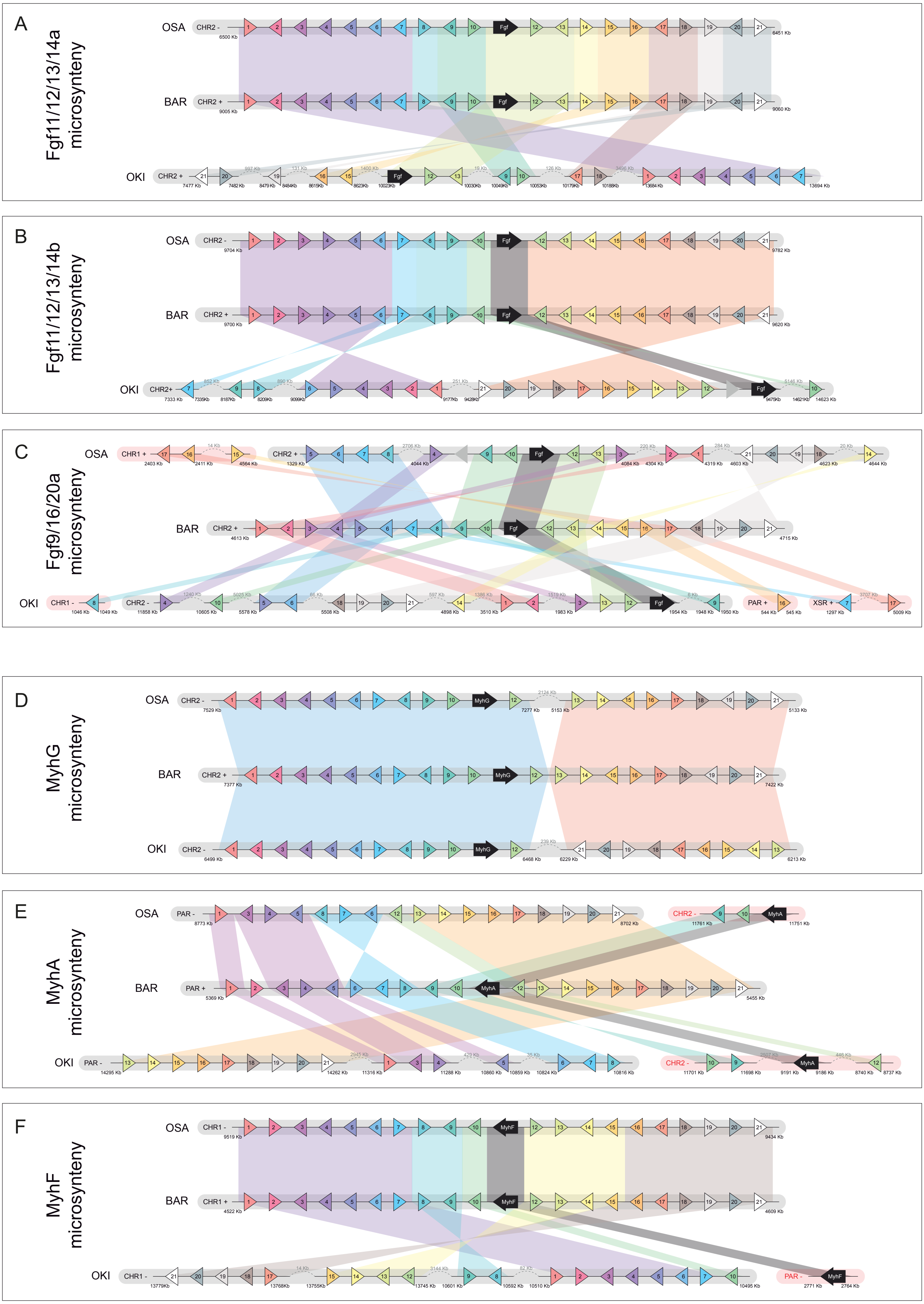

Supplemental Figure S5 (continued)

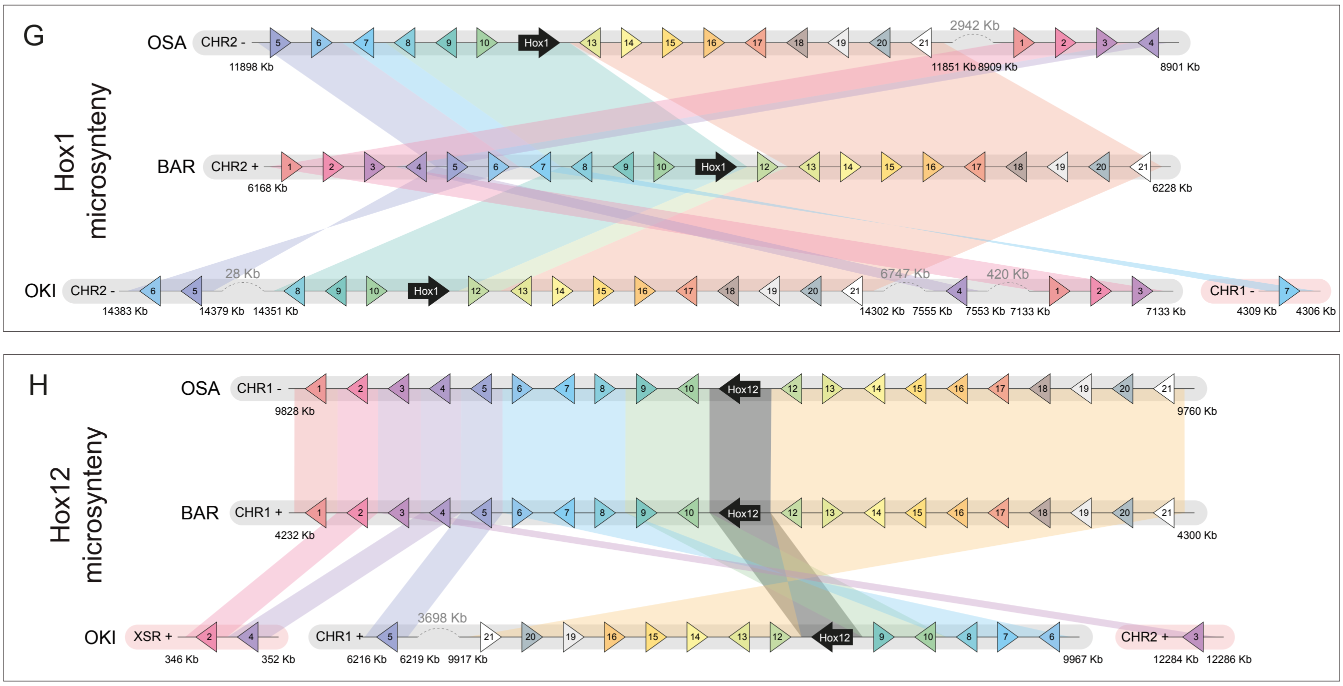

Supplemental Figure S6: Conservation of operons as defined by different matching criteria. We assessed operon conservation using sets of operons defined by different intergenic distances, gene equivalence criteria, and operon equivalence criteria.

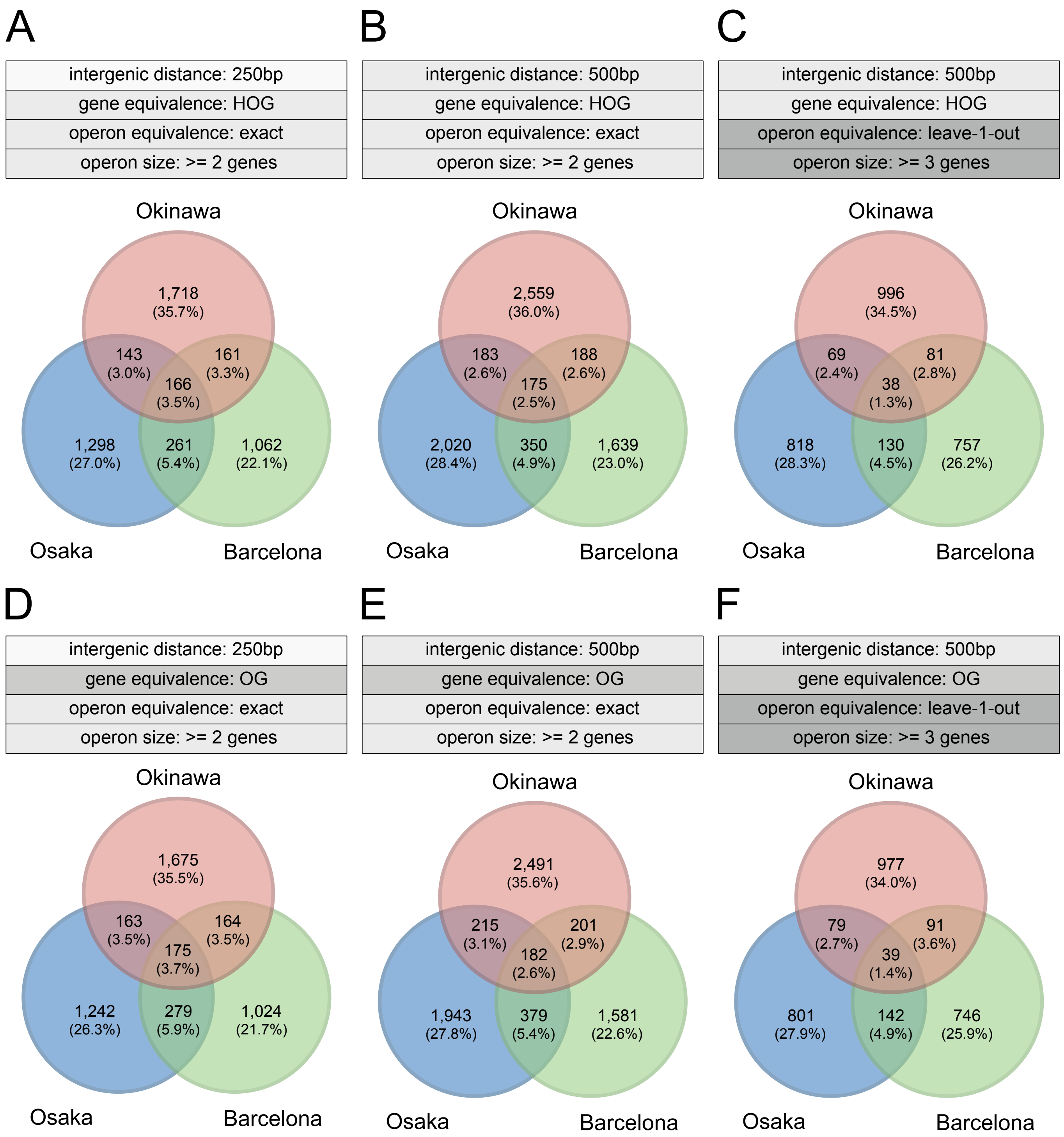

Supplemental Figure S7: Enrichment of genomic features at the boundary between breakpoint or bridge regions and aligned regions in various pairwise comparison (complement to Figure 3C). CNE: conserved non-coding elements. Reps: repeat elements.

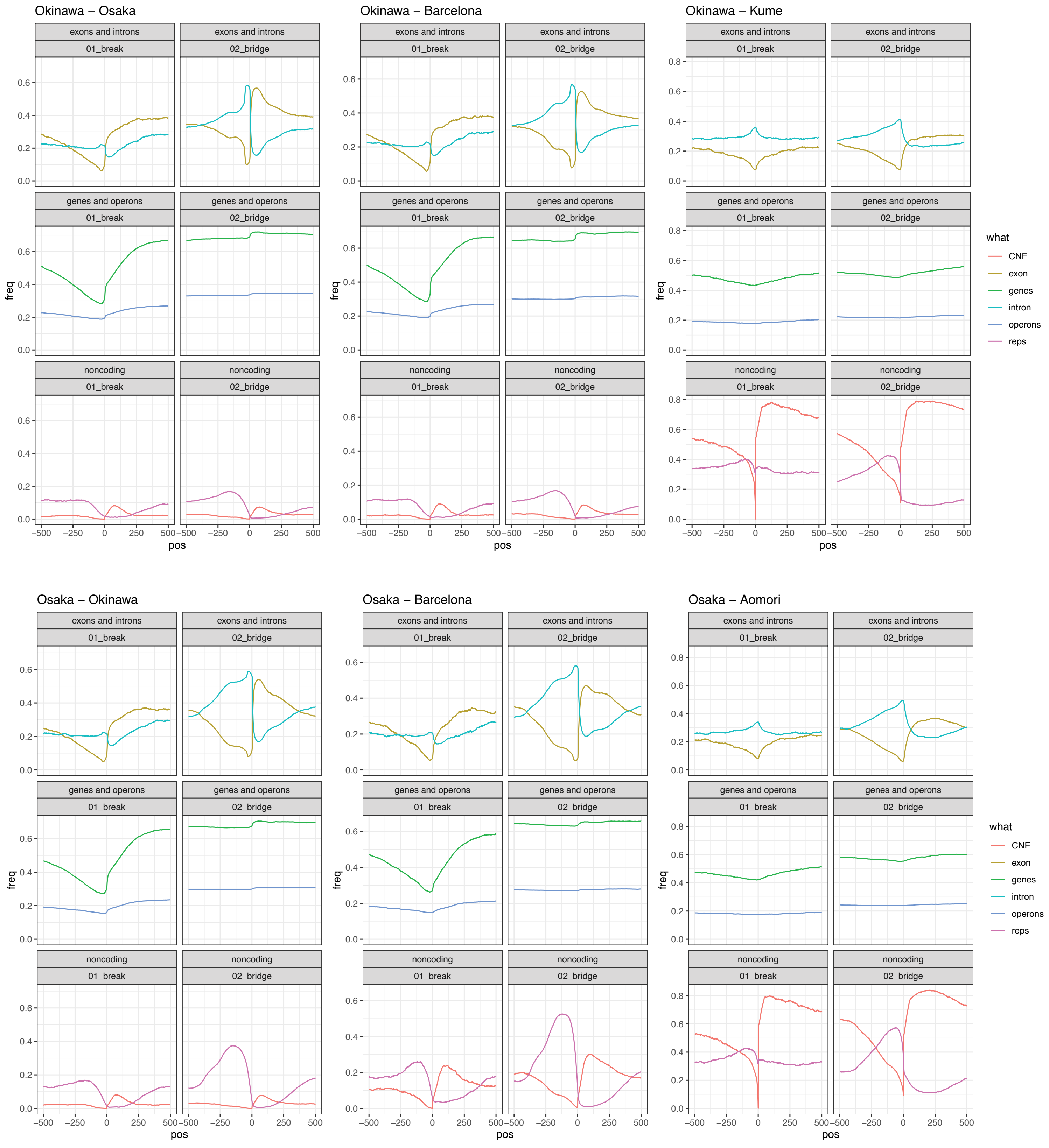

Supplemental Figure S8: Repeat density in *Oikopleura* genomes. The result for the Okinawan genome was originally reported in Bliznina et al. (2021), but is plotted here to facilitate comparisons with *O. dioica* from Osaka and Barcelona.

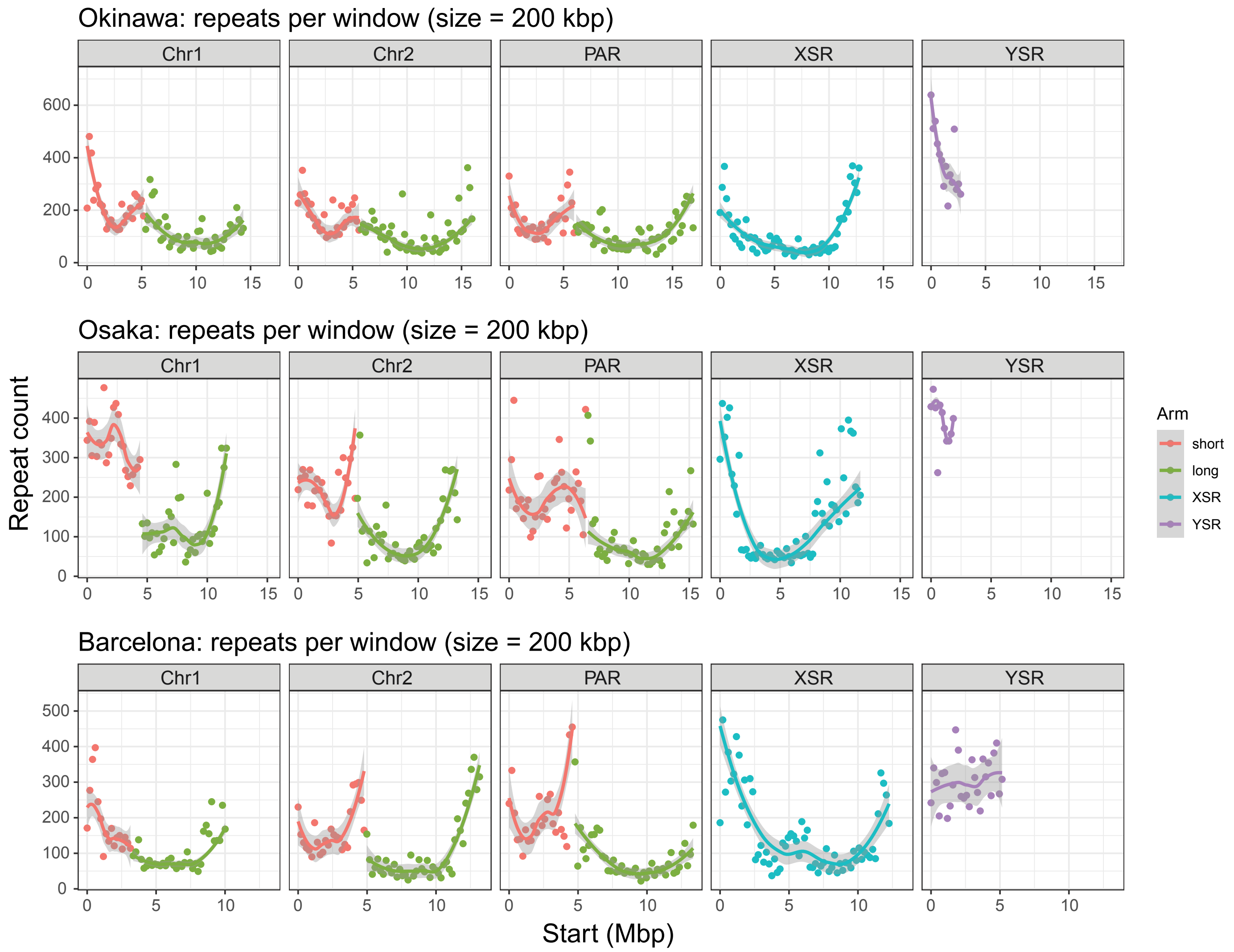

Supplemental Figure S9: Breakpoint accumulation in *Oikopleura*, *Ciona*, *Drosophila* and *Caenorhabditis* at close (near), mid (intermediary) and far (distant) evolutionary distance. The purpose of this figure is to provide extra context to the main text Figure 7 panel E and help the readers assess our choice for the normalisation. A) Number of breakpoints. B) Number of breakoints normalised by genome size, in megabases. C) Number of breakpoints normalised by genome size, excluding the regions that were not aligned (to take into account for instance that some *Drosophila* genomes assemblies contain very large centromeric regions). D) Number of breakpoints normalised by evolutionary distance, in million years. The evolutionary distance was estimated by a molecular clock (*Oikopleura*, main text Figure 5), or taken from the literature. E) Number of breakpoint normalised by genome size and evolutionary distance. This panel displays the same data as Figure 7E and is the direct output of R scripts (colors and bar orders were then edited in Figure 7E to match the other panels of the figure). F) Number of breakpoints normalised by alignment length (see C.) and evolutionary distance.

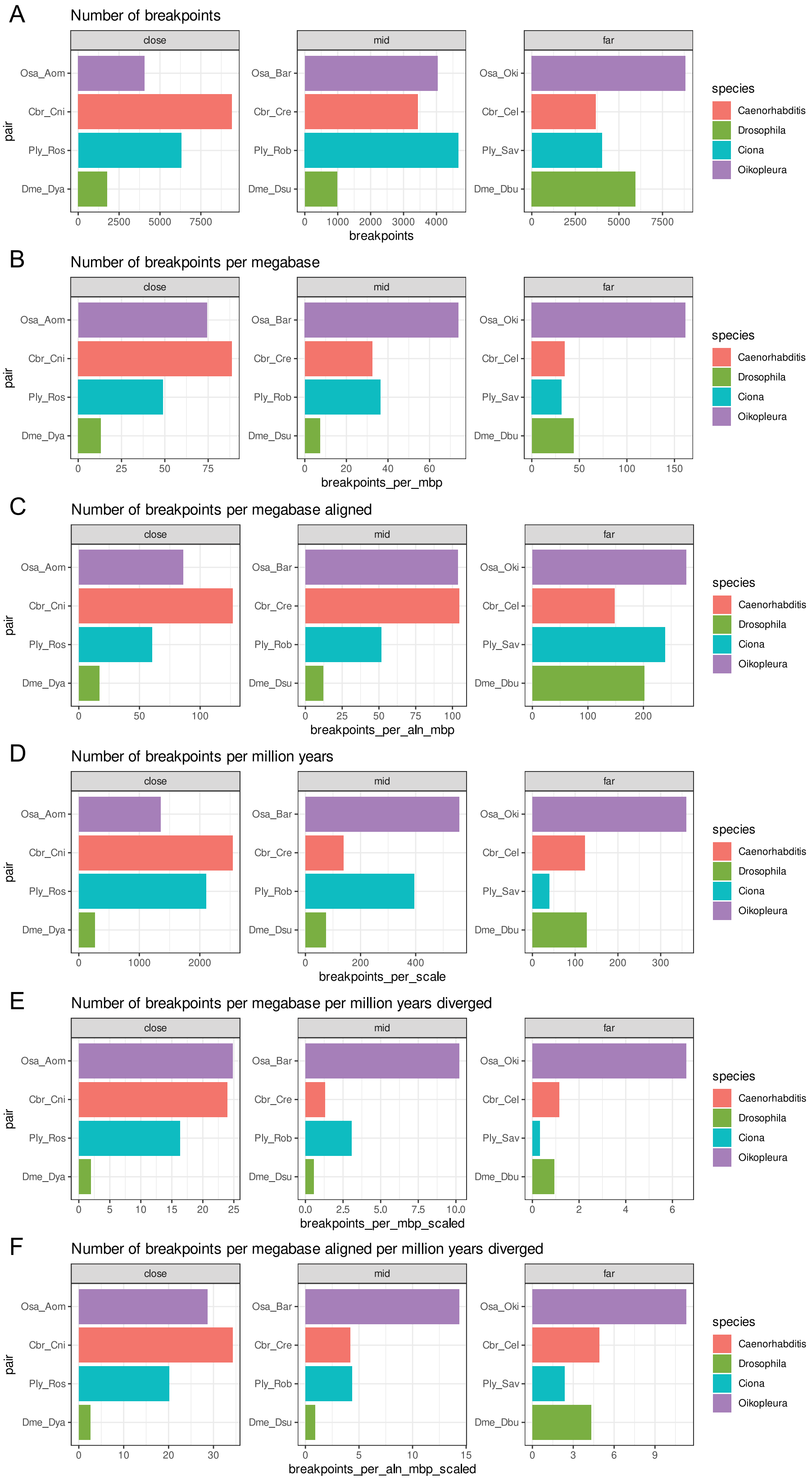

Supplemental Figure S10: Pairwise estimations for dN, dS, and dN/dS values for every genome pair. A) Maximum likelihood estimates and B) Bayesian estimates (calculated using the runmode = -2 and runmode = -3 settings in PAML).

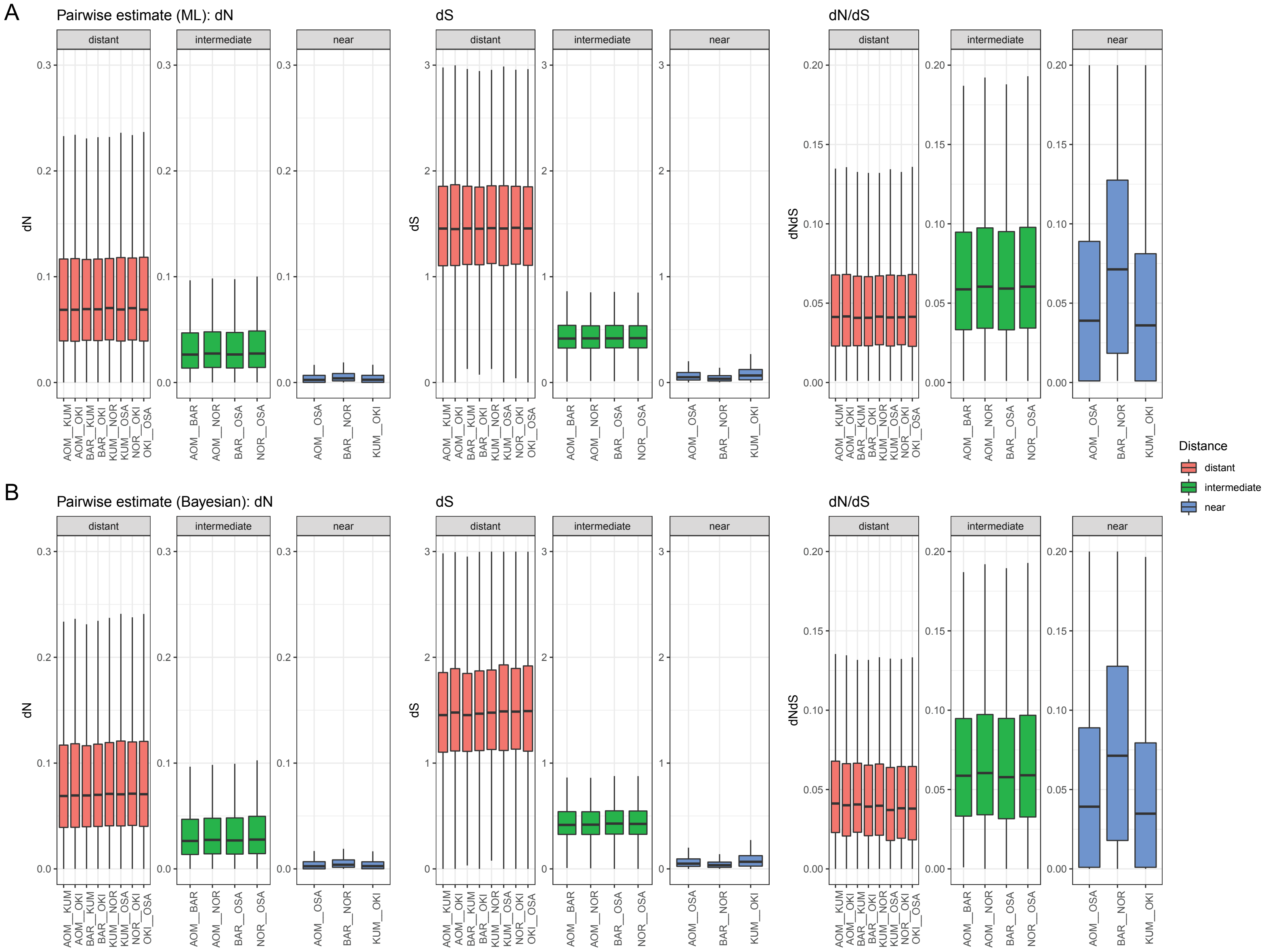

Supplement: Supplement 5 [file Supplemental_Figures.pdf]
