# Supplementary material for: Extreme genome scrambling in marine planktonic Oikopleura dioica cryptic species
Source: Genome Res. 2024 Mar;34(3):426–40. doi: 10.1101/gr.278295.123 (PMC11067885; doi:10.1101/gr.278295.123)
Supplement: Supplement 4 [file Supplemental_Data.zip › Supplemental_Data.tar.xz!/Supplemental_Data/articles/OxfordPlots_files/figure-html/Oki_Kum-1.pdf]

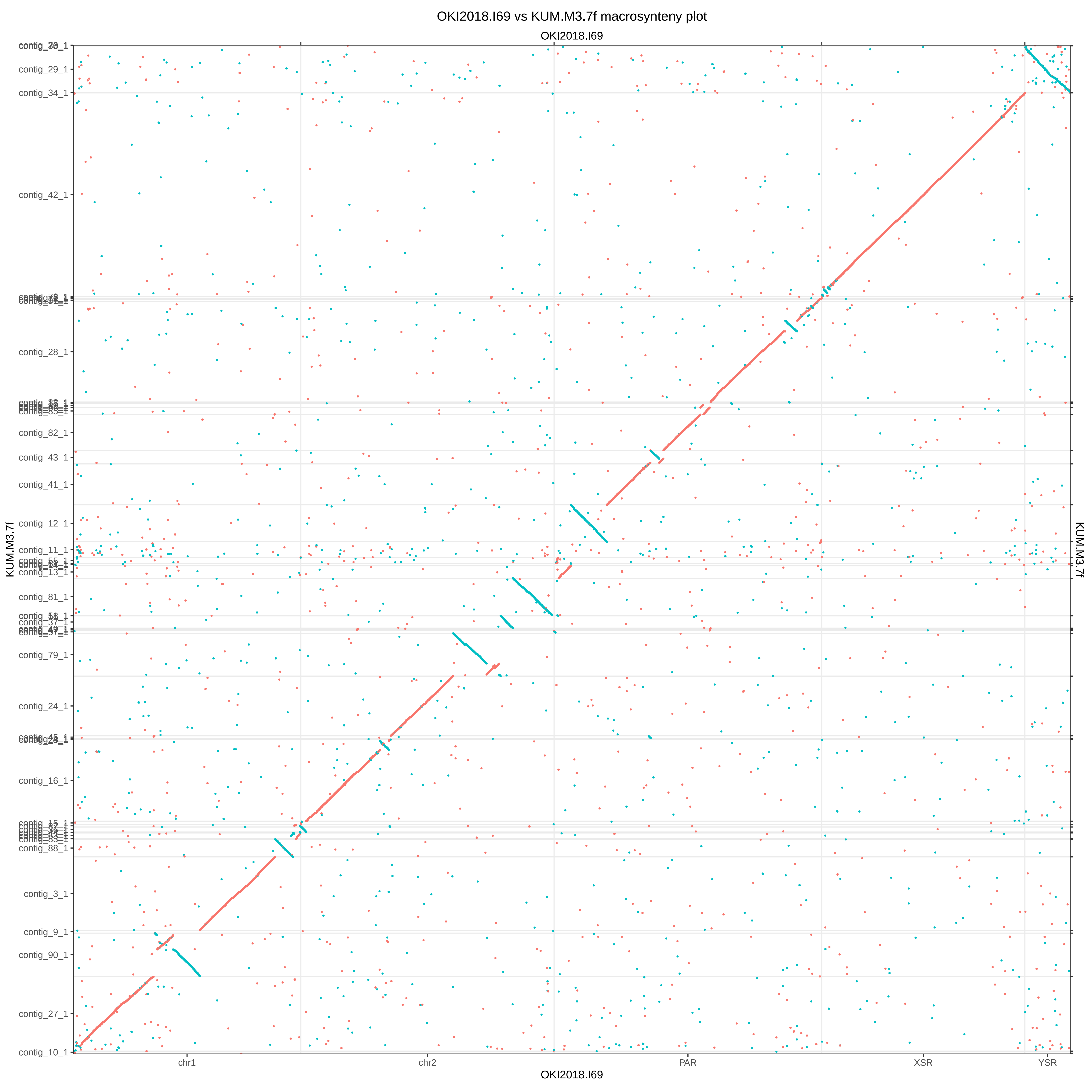
OKI2018.I69 vs KUM.M3.7f macrosynteny plot
OKI2018.I69
contig_29_1
contig_34_1
contig_42_1
contig_28_1
contig_83_1
contig_82_1
contig_43_1
contig_41_1
contig_12_1
contig_11_1
contig_55_1
contig_13_1
contig_81_1
contig_37_1
contig_79_1
contig_24_1
contig_45_1
contig_16_1
contig_15_1
contig_88_1
contig_3_1
contig_9_1
contig_90_1
contig_27_1
contig_10_1
KUM.M3.7f
chr1
chr2
PAR
XSR
YSR
OKI2018.I69
KUM.M3.7f
