# Supplementary material for: Extreme genome scrambling in marine planktonic Oikopleura dioica cryptic species
Source: Genome Res. 2024 Mar;34(3):426–40. doi: 10.1101/gr.278295.123 (PMC11067885; doi:10.1101/gr.278295.123)
Supplement: Supplement 4 [file Supplemental_Data.zip › Supplemental_Data.tar.xz!/Supplemental_Data/articles/OxfordPlots_files/figure-html/Ply_Ros-1.pdf]

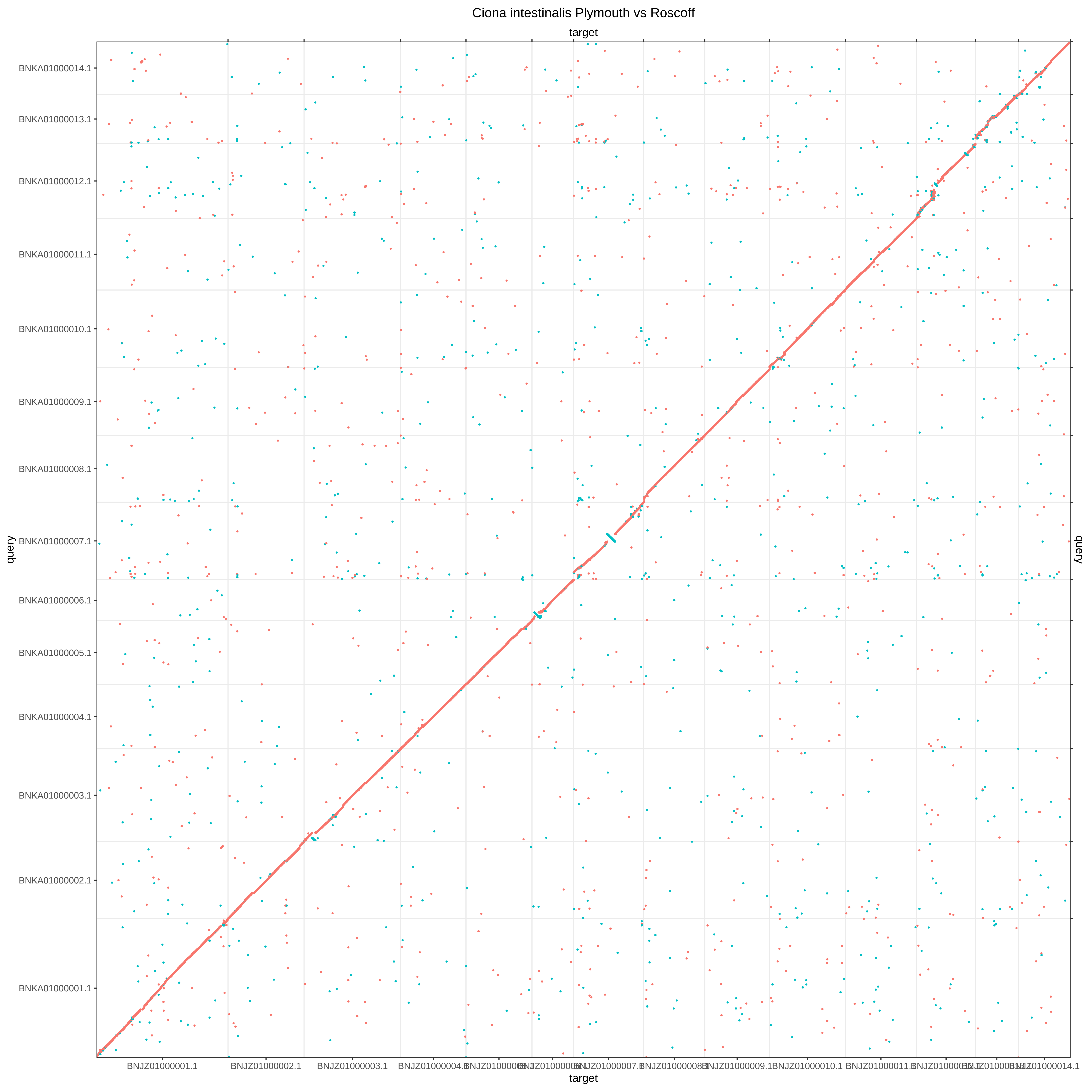

Ciona intestinalis Plymouth vs Roscoff
target
query
BNKA01000014.1
BNKA01000013.1
BNKA01000012.1
BNKA01000011.1
BNKA01000010.1
BNKA01000009.1
BNKA01000008.1
BNKA01000007.1
BNKA01000006.1
BNKA01000005.1
BNKA01000004.1
BNKA01000003.1
BNKA01000002.1
BNKA01000001.1
BNJZ01000001.1
BNJZ01000002.1
BNJZ01000003.1
BNJZ01000010.1
target
query
