# Supplementary material for: Extreme genome scrambling in marine planktonic Oikopleura dioica cryptic species
Source: Genome Res. 2024 Mar;34(3):426–40. doi: 10.1101/gr.278295.123 (PMC11067885; doi:10.1101/gr.278295.123)
Supplement: Supplement 4 [file Supplemental_Data.zip › Supplemental_Data.tar.xz!/Supplemental_Data/articles/OxfordPlots_files/figure-html/Cbr_Cre-1.pdf]

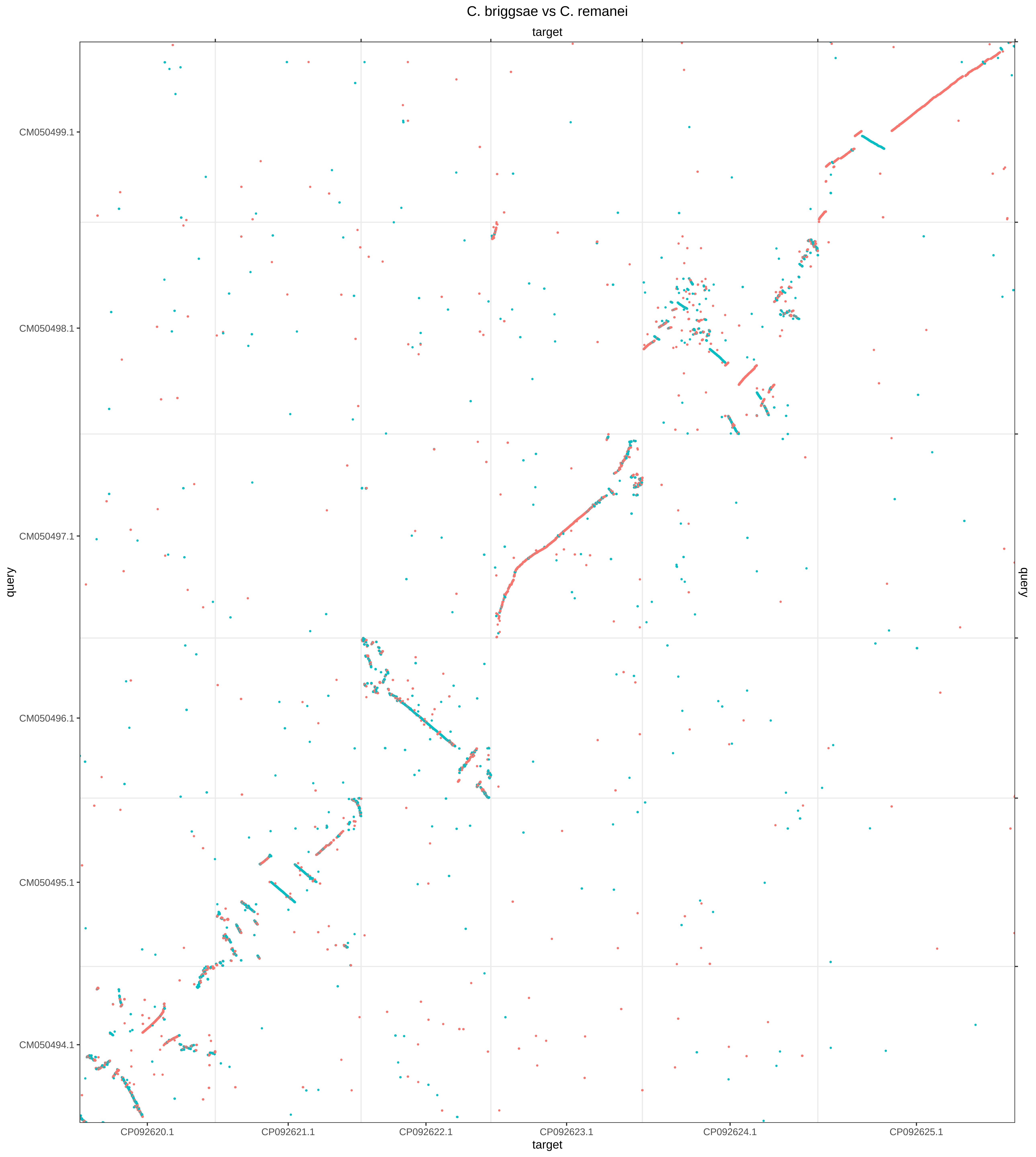

C. briggsae vs C. remanei
target
query
CM050499.1
CM050498.1
CM050497.1
CM050496.1
CM050495.1
CM050494.1
CP092620.1
CP092621.1
CP092622.1
CP092623.1
CP092624.1
CP092625.1
