# Supplementary material for: Extreme genome scrambling in marine planktonic Oikopleura dioica cryptic species
Source: Genome Res. 2024 Mar;34(3):426–40. doi: 10.1101/gr.278295.123 (PMC11067885; doi:10.1101/gr.278295.123)
Supplement: Supplement 4 [file Supplemental_Data.zip › Supplemental_Data.tar.xz!/Supplemental_Data/articles/OxfordPlots_files/figure-html/Bar_Nor-1.pdf]

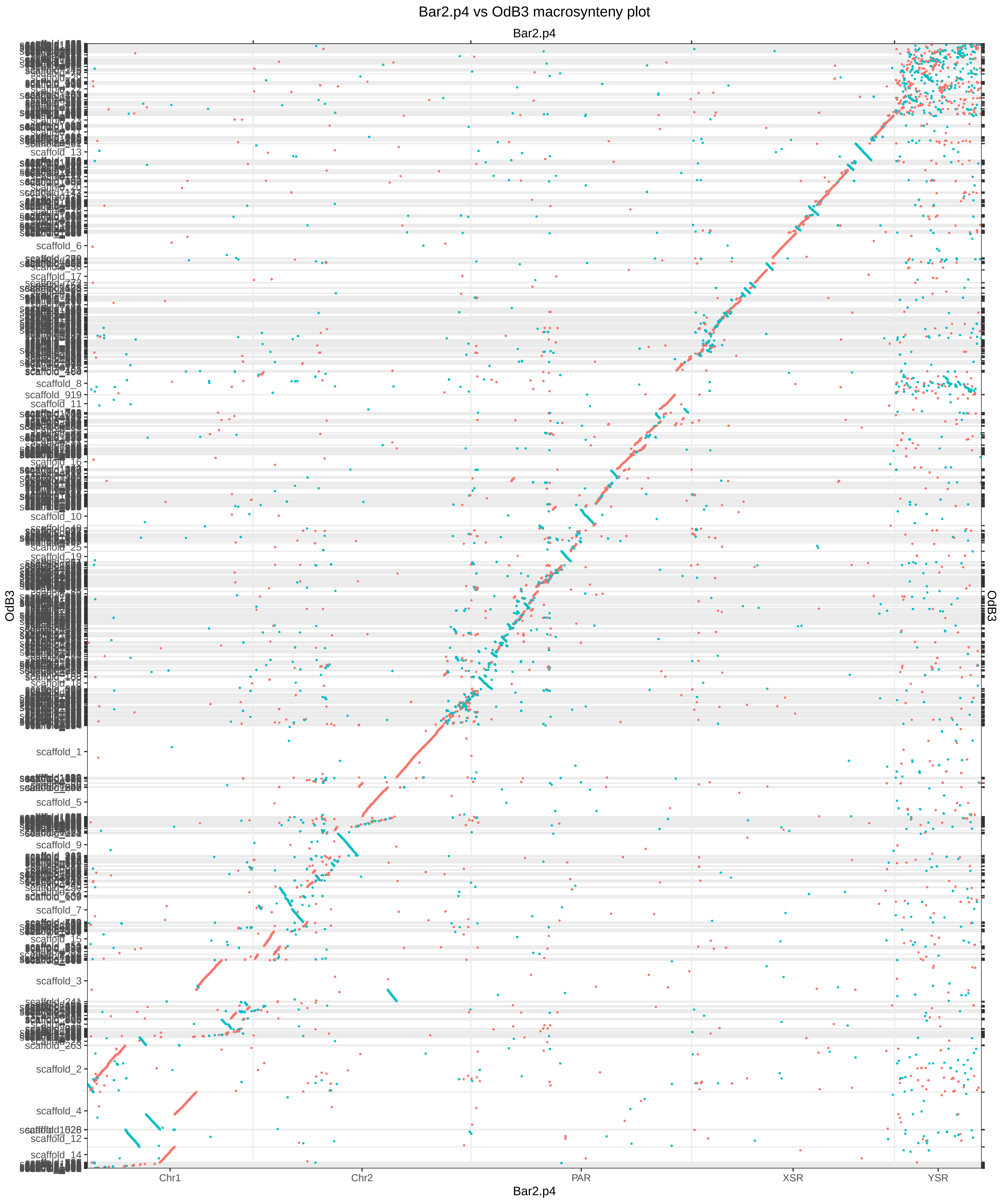

Bar2.p4 vs OdB3 macrosynteny plot
Bar2.p4
OdB3
scaffold_13
scaffold_6
scaffold_17
scaffold_8
scaffold_919
scaffold_11
scaffold_16
scaffold_10
scaffold_25
scaffold_19
scaffold_18
scaffold_1
scaffold_5
scaffold_9
scaffold_7
scaffold_15
scaffold_3
scaffold_263
scaffold_2
scaffold_4
scaffold_12
scaffold_14
Chr1
Chr2
PAR
XSR
YSR
Bar2.p4
