# Supplementary material for: Extreme genome scrambling in marine planktonic Oikopleura dioica cryptic species
Source: Genome Res. 2024 Mar;34(3):426–40. doi: 10.1101/gr.278295.123 (PMC11067885; doi:10.1101/gr.278295.123)
Supplement: Supplement 4 [file Supplemental_Data.zip › Supplemental_Data.tar.xz!/Supplemental_Data/articles/OxfordPlots_files/figure-html/Oki_Bar_all-1.pdf]

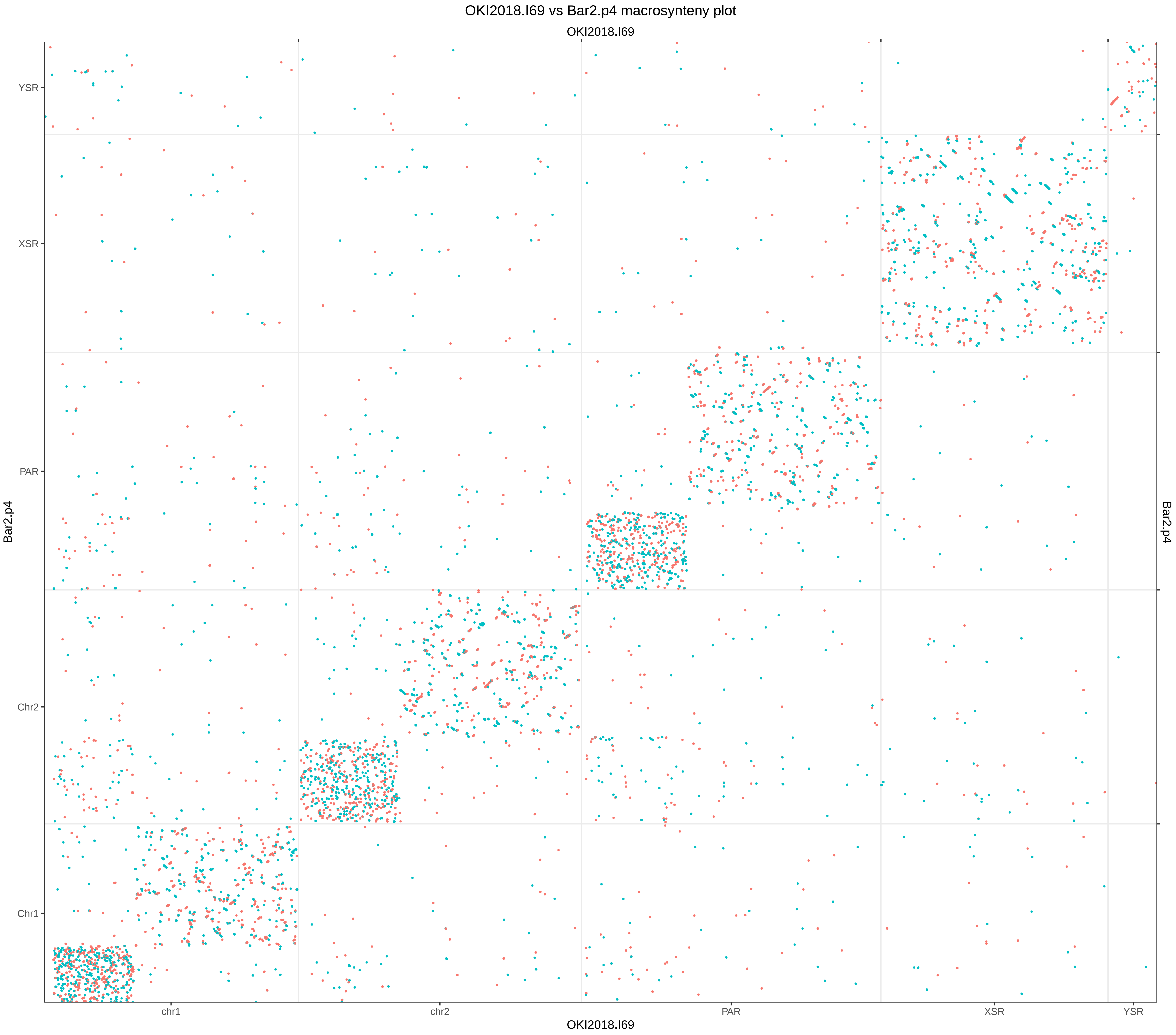

OKI2018.I69 vs Bar2.p4 macrosynteny plot
OKI2018.I69
YSR
XSR
PAR
Chr2
Chr1
Bar2.p4
Bar2.p4
chr1
chr2
PAR
XSR
YSR
OKI2018.I69
