# Supplementary material for: Extreme genome scrambling in marine planktonic Oikopleura dioica cryptic species
Source: Genome Res. 2024 Mar;34(3):426–40. doi: 10.1101/gr.278295.123 (PMC11067885; doi:10.1101/gr.278295.123)
Supplement: Supplement 4 [file Supplemental_Data.zip › Supplemental_Data.tar.xz!/Supplemental_Data/articles/OxfordPlots_files/figure-html/Cni_Cin-1.pdf]

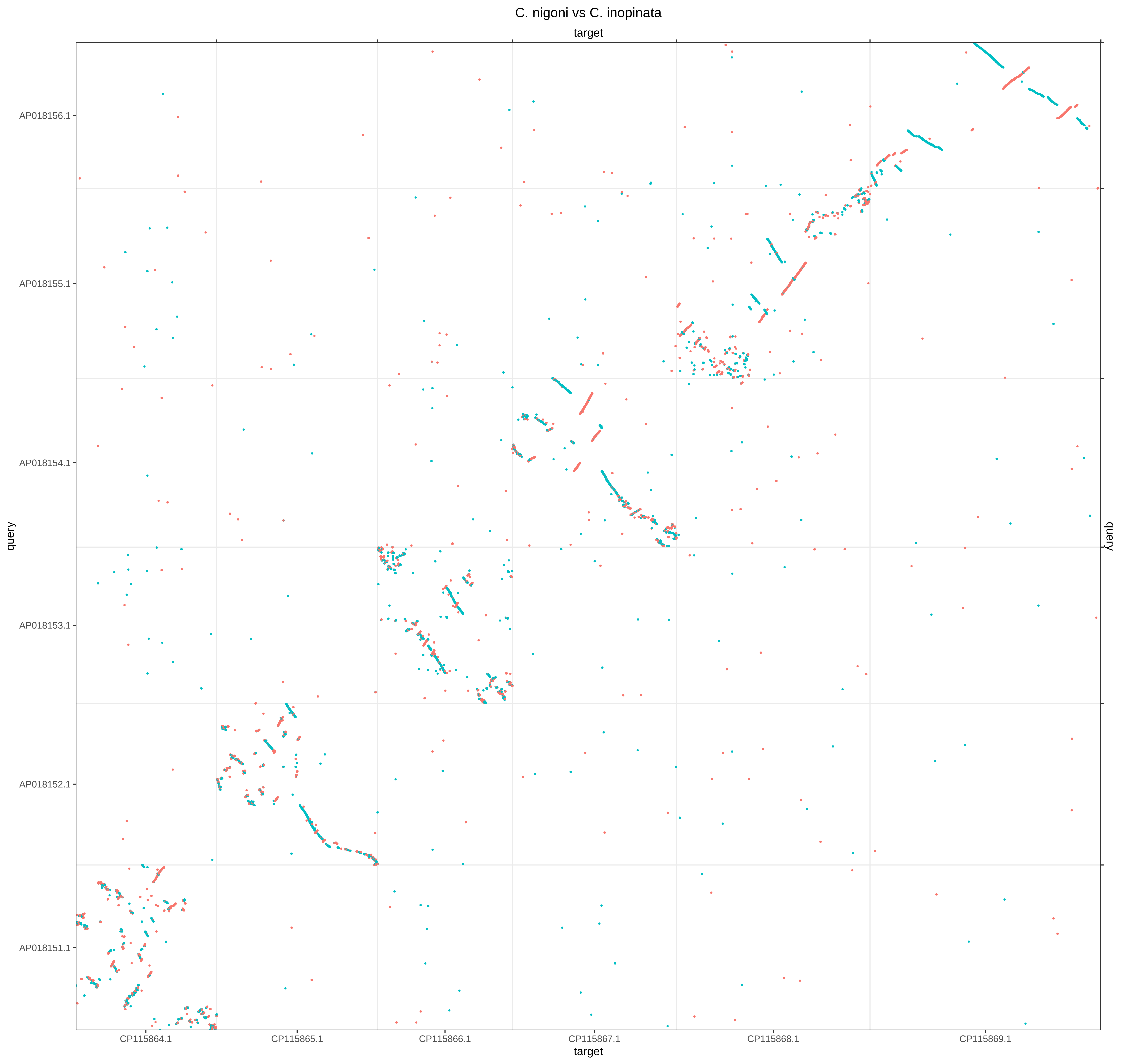
C. nigoni vs C. inopinata
target
query
AP018156.1
AP018155.1
AP018154.1
AP018153.1
AP018152.1
AP018151.1
CP115864.1
CP115865.1
CP115866.1
CP115867.1
CP115868.1
CP115869.1
