# Supplementary material for: Extreme genome scrambling in marine planktonic Oikopleura dioica cryptic species
Source: Genome Res. 2024 Mar;34(3):426–40. doi: 10.1101/gr.278295.123 (PMC11067885; doi:10.1101/gr.278295.123)
Supplement: Supplement 4 [file Supplemental_Data.zip › Supplemental_Data.tar.xz!/Supplemental_Data/articles/OxfordPlots_files/figure-html/Dmel_Dbus-1.pdf]

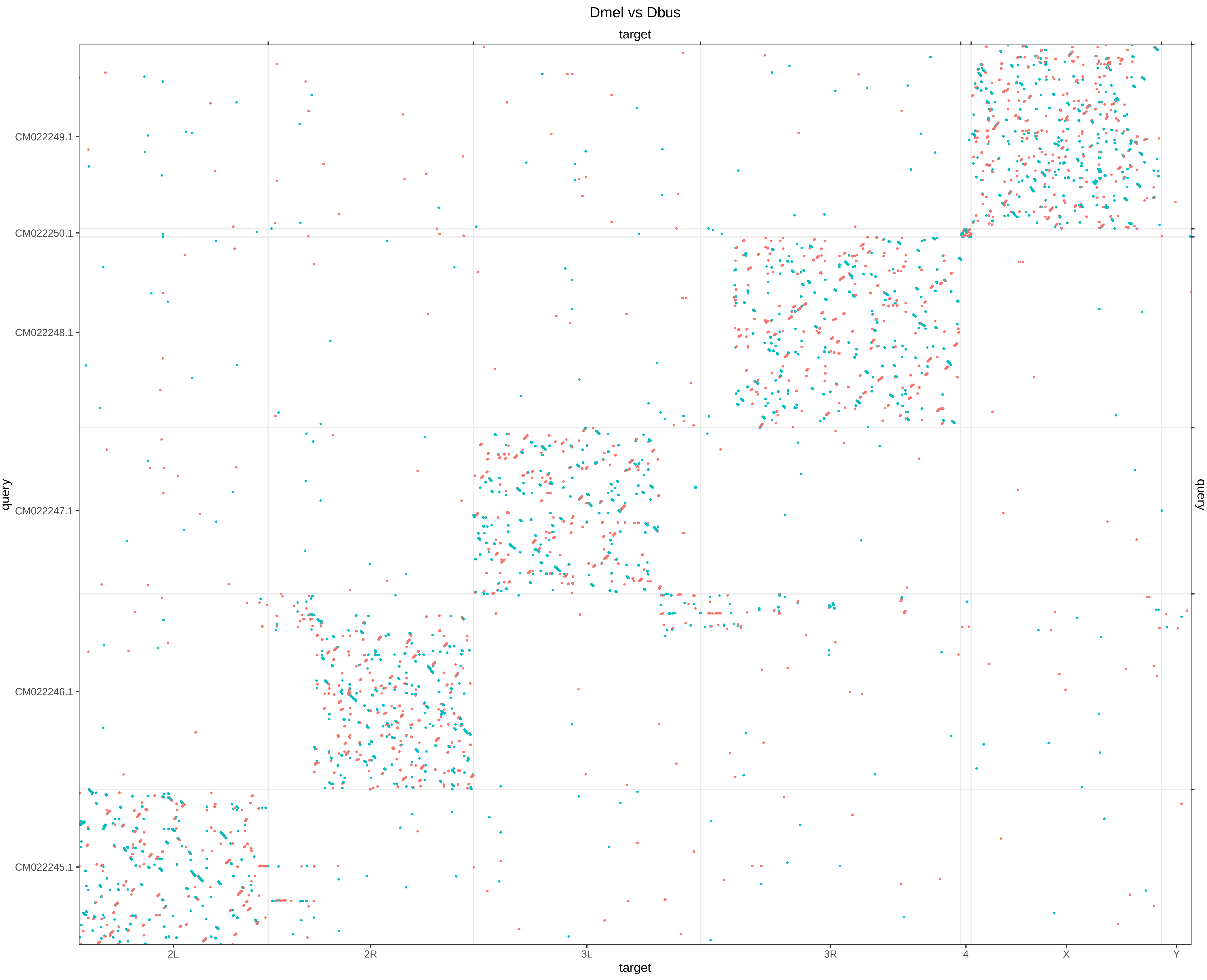

Dmel vs Dbus
target
CM022249.1
CM022250.1
CM022248.1
CM022247.1
CM022246.1
CM022245.1
query
query
2L
2R
3L
3R
4
X
Y
target
