# Supplementary material for: Extreme genome scrambling in marine planktonic Oikopleura dioica cryptic species
Source: Genome Res. 2024 Mar;34(3):426–40. doi: 10.1101/gr.278295.123 (PMC11067885; doi:10.1101/gr.278295.123)
Supplement: Supplement 4 [file Supplemental_Data.zip › Supplemental_Data.tar.xz!/Supplemental_Data/articles/OxfordPlots_files/figure-html/Dmel_Dyak-1.pdf]

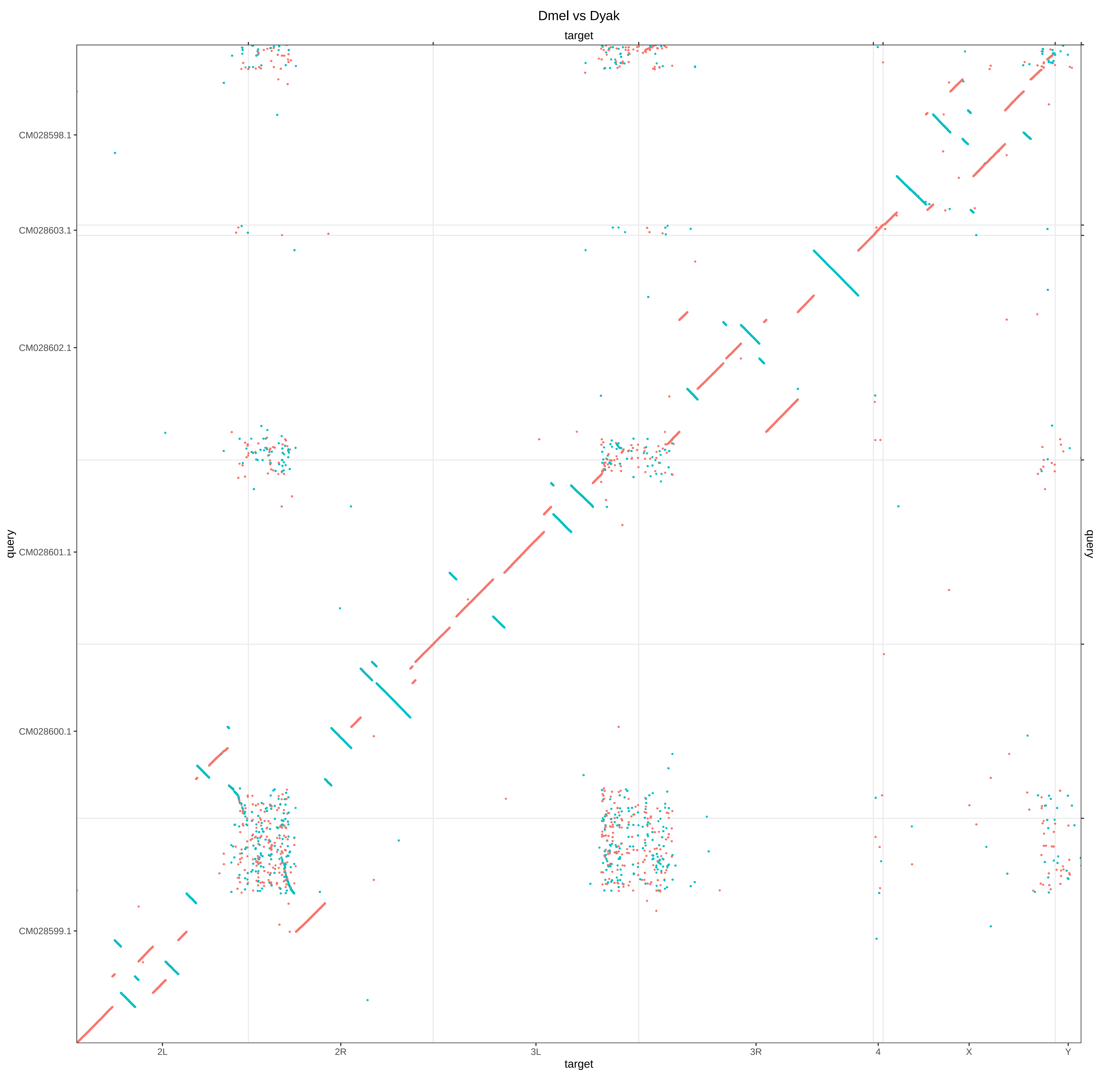
Dmel vs Dyak
target
query
CM028598.1
CM028603.1
CM028602.1
CM028601.1
CM028600.1
CM028599.1
2L
2R
3L
3R
4
X
Y
target
query
