# Supplementary material for: Extreme genome scrambling in marine planktonic Oikopleura dioica cryptic species
Source: Genome Res. 2024 Mar;34(3):426–40. doi: 10.1101/gr.278295.123 (PMC11067885; doi:10.1101/gr.278295.123)
Supplement: Supplement 4 [file Supplemental_Data.zip › Supplemental_Data.tar.xz!/Supplemental_Data/articles/DistanceMeasures.html]

Distance Measures • OikScrambling
 


  


 


Toggle navigation


OikScrambling
5.1.0

- Get started
- Data load
- Supplemental material
  - Figure 1B and S1 DNA line plots
  - Figure 1C chromosome parallel plots
  - Figure 1D scrambling index
  - Figure 2A and S1 orthogroup dot plots and synteny block histograms
  - Figure 3C breakpoint feature density plots
  - Figure 4B and 4C operon barplots
  - Figure 5 genome-wide feature patterns
  - Figure 7A–D DNA line plots
  - Figure 7E breakpoint number barplots
- Articles
  - Scaffolding of the AOM-5 genome
  - Scaffolding of the OdB3 genome
  - Conserved non-coding elements
  - Chromosome-scale feature density plots
  - Modifying coalescent cutoffs
  - Regions interrupting colinearity
  - Distance Measures
  - Genomic features of the breakpoints
  - Genomic features of the breakpoints (heatmaps)
  - Inversions
  - Scaffolding of the KUM-M3 genome
  - Load genomic break data
  - Operon conservation analysis
  - Operon detection and counting
  - Orthogroup syntenies
  - Alignment line plots, whole genomes
  - Region of interest: PAC3
  - Motif search and analysis
  - Whole-chromosome parallel plots
  - Width of the aligned and mapped regions.
  - Regions of interest
  - Repeat regions
  - Scaling breakpoints
  - Segment movement distances
  - Alignment line plots, 10×10 Mbp windows
  - Transcription factor binding sites
  - Tandem site duplications
  - Translocations
- Changelog
- Reference

# Distance Measures

#### Charles Plessy

#### 20 November, 2023

Source: `vignettes/DistanceMeasures.Rmd`

`DistanceMeasures.Rmd`

## Purpose

We want to put numbers on visual impressions such as:

- There is a lot of scrambling.
- Aligned regions stay on their chromosome arm.
- Aligned regions are more “off the diagonal” on short arms than on
  long arms.
- …

\*\* Figure 1D’s *scrambling index* table is computed here under
the name *strand randomisation indexes*.\*\*

## Load R pacakges and data

```
library('OikScrambling') |> suppressPackageStartupMessages()
load("BreakPoints.Rdata")
```

See
`vignette("LoadGenomicBreaks", package = "OikScrambling")`
for how the different GBreaks objects are prepared.

## Declare service functions

```
pairs2table6x3 <- function (x) {
  x <- as.list(x)
  cbind(
    Oki = c(Oki = NA,        Osa = x$Osa_Oki, Bar = x$Bar_Oki),
    Kum = c(Oki = x$Oki_Kum, Osa = x$Osa_Kum, Bar = x$Bar_Kum),
    Osa = c(Oki = x$Oki_Osa, Osa = NA,        Bar = x$Bar_Osa),
    Aom = c(Oki = x$Oki_Aom, Osa = x$Osa_Aom, Bar = x$Bar_Aom),
    Bar = c(Oki = x$Oki_Bar, Osa = x$Osa_Bar, Bar = NA       ),
    Nor = c(Oki = x$Oki_Nor, Osa = x$Osa_Nor, Bar = x$Bar_Nor)
  )
}

pairs2Kable6x3 <- function (df) df |> pairs2table6x3() |> round(2) |> knitr::kable()
```

## Strand randomisation index

See `?GenomicBreaks::strand_randomisation_index` for
details.

```
strand_randomisation_index
```

```
## function (gb) 
## {
##     if (length(gb) == 0) 
##         return(numeric(0))
##     if (length(gb) == 1) 
##         return(1)
##     gbl <- split(gb, seqnames(gb), drop = TRUE)
##     idx <- sapply(gbl, function(x) {
##         onPlus <- sum(width(x[strand(x) == "+"]))
##         onMinus <- sum(width(x[strand(x) == "-"]))
##         abs((onPlus - onMinus)/(onPlus + onMinus))
##     })
##     weighted.mean(idx, sum(width(gbl)))
## }
## <bytecode: 0x5631f449d200>
## <environment: namespace:GenomicBreaks>
```

The computation of strand randomisation indices is not symmetric: the
results differ a bit when the roles of *target* and
*query* genomes are swapped.

```
SRIs         <- BiocParallel::bplapply(gbs,                 strand_randomisation_index  )
SRIS_swapped <- BiocParallel::bplapply(gbs, \(g) swap(g) |> strand_randomisation_index())
```

### Table 1

We averaged the numbers computed with and without swapping
*target* and *query* genomes.

```
as.list(1 / 2 * (unlist(SRIs) + unlist(SRIS_swapped))) |> pairs2Kable6x3()
```

|  | Oki | Kum | Osa | Aom | Bar | Nor |
| --- | --- | --- | --- | --- | --- | --- |
| Oki | NA | 0.67 | 0.04 | 0.08 | 0.10 | 0.18 |
| Osa | 0.04 | 0.06 | NA | 0.54 | 0.23 | 0.28 |
| Bar | 0.10 | 0.11 | 0.23 | 0.22 | NA | 0.50 |

### Unidirectional computation

The raw data is shown here to prove that the differences do not
affect general conclusions.

```
SRIs         |> pairs2Kable6x3()
```

|  | Oki | Kum | Osa | Aom | Bar | Nor |
| --- | --- | --- | --- | --- | --- | --- |
| Oki | NA | 0.52 | 0.04 | 0.06 | 0.10 | 0.05 |
| Osa | 0.05 | 0.06 | NA | 0.43 | 0.23 | 0.10 |
| Bar | 0.10 | 0.09 | 0.23 | 0.19 | NA | 0.18 |

```
SRIS_swapped |> pairs2Kable6x3()
```

|  | Oki | Kum | Osa | Aom | Bar | Nor |
| --- | --- | --- | --- | --- | --- | --- |
| Oki | NA | 0.83 | 0.05 | 0.10 | 0.10 | 0.31 |
| Osa | 0.04 | 0.06 | NA | 0.66 | 0.23 | 0.47 |
| Bar | 0.10 | 0.14 | 0.23 | 0.25 | NA | 0.82 |

## Synteny index

Ad-hoc index measuring to what extent a scaffold of the
*target* genome is mostly aligned to a single scaffold in the
*query* genome. See `?GenomicBreaks::synteny_index`
for details. One limitation to the use of this index is that it requires
that at least the *query* genome is a complete chromosome
assembly.

```
synteny_index
```

```
## function (gb) 
## {
##     if (length(gb) == 0) 
##         return(numeric(0))
##     if (length(gb) == 1) 
##         return(1)
##     gbl <- split(gb, seqnames(gb), drop = TRUE)
##     synIdx <- sapply(gbl, function(x) {
##         alnOnMainHit <- tail(sort(sum(width(split(x, seqnames(x$query))))), 
##             1)
##         alnOnMainHit/sum(width(x))
##     })
##     weighted.mean(synIdx, sum(width(gbl)))
## }
## <bytecode: 0x5631ef2a6748>
## <environment: namespace:GenomicBreaks>
```

```
BiocParallel::bplapply(gbs,                 synteny_index  ) |> pairs2Kable6x3()
```

|  | Oki | Kum | Osa | Aom | Bar | Nor |
| --- | --- | --- | --- | --- | --- | --- |
| Oki | NA | 0.51 | 0.94 | 0.65 | 0.94 | 0.17 |
| Osa | 0.94 | 0.52 | NA | 0.61 | 0.97 | 0.17 |
| Bar | 0.94 | 0.53 | 0.97 | 0.66 | NA | 0.16 |

```
BiocParallel::bplapply(gbs, \(g) swap(g) |> synteny_index()) |> pairs2Kable6x3()
```

|  | Oki | Kum | Osa | Aom | Bar | Nor |
| --- | --- | --- | --- | --- | --- | --- |
| Oki | NA | 0.97 | 0.94 | 0.94 | 0.94 | 0.93 |
| Osa | 0.94 | 0.94 | NA | 0.97 | 0.97 | 0.96 |
| Bar | 0.94 | 0.94 | 0.97 | 0.97 | NA | 0.95 |

## Correlation

Ad-hoc index measuring the correlation of the coordinates of the
syntenic alignments in scaffolds of a *target* genome and their
best match in the *query* genome. See
`?GenomicBreaks::correlation_index` for details. It is
intended to be more robust to the presence of uncollapsed haplotypes in
the *query* genome but probably needs further testing.

```
correlation_index
```

```
## function (gb) 
## {
##     if (length(gb) == 0) 
##         return(numeric(0))
##     if (length(gb) == 1) 
##         return(1)
##     gbl <- split(gb, seqnames(gb), drop = TRUE)
##     corIdx <- sapply(gbl, function(x) {
##         bestQuery <- names(tail(sort(table(seqnames(x$query))), 
##             1))
##         x <- x[seqnames(x$query) == bestQuery]
##         if (length(x) == 1) 
##             return(1)
##         weights <- width(x)
##         x <- resize(x, fix = "center", width = 1)
##         x$query <- resize(x$query, fix = "center", width = 1)
##         weights::wtd.cor(start(x), start(x$query), weights)[1]
##     })
##     weighted.mean(corIdx, sum(width(gbl)))
## }
## <bytecode: 0x5631de11af38>
## <environment: namespace:GenomicBreaks>
```

```
BiocParallel::bplapply(gbs,                 correlation_index  ) |> pairs2Kable6x3()
```

|  | Oki | Kum | Osa | Aom | Bar | Nor |
| --- | --- | --- | --- | --- | --- | --- |
| Oki | NA | 0.89 | 0.48 | -0.03 | 0.44 | -0.19 |
| Osa | 0.48 | -0.06 | NA | -0.43 | 0.37 | 0.30 |
| Bar | 0.44 | -0.01 | 0.36 | 0.07 | NA | 0.36 |

```
BiocParallel::bplapply(gbs, \(g) swap(g) |> correlation_index()) |> pairs2Kable6x3()
```

|  | Oki | Kum | Osa | Aom | Bar | Nor |
| --- | --- | --- | --- | --- | --- | --- |
| Oki | NA | 0.51 | 0.48 | -0.01 | 0.44 | 0.01 |
| Osa | 0.48 | -0.01 | NA | -0.22 | 0.36 | 0.01 |
| Bar | 0.44 | 0.03 | 0.36 | 0.11 | NA | 0.20 |

## Gene Order Conservation

See `?GenomicBreaks::GOC` for details.

```
GOC
```

```
## function (gb, vicinity = 4, debug = FALSE) 
## {
##     if (length(gb) == 0) 
##         return(numeric(0))
##     if (length(gb) == 1) 
##         return(1)
##     gb <- sort(gb, ignore.strand = TRUE)
##     gb <- flagColinearAlignments(gb, details = TRUE)
##     gb$GOCi <- abs(gb$qfoll) <= vicinity
##     if (debug) 
##         return(gb)
##     gb[is.na(gb$tfoll)] <- NULL
##     sum(gb$GOCi %in% TRUE)/length(gb)
## }
## <bytecode: 0x5632001b2478>
## <environment: namespace:GenomicBreaks>
```

```
BiocParallel::bplapply(gbs,                 GOC  ) |> pairs2Kable6x3()
```

|  | Oki | Kum | Osa | Aom | Bar | Nor |
| --- | --- | --- | --- | --- | --- | --- |
| Oki | NA | 0.76 | 0.84 | 0.85 | 0.85 | 0.82 |
| Osa | 0.84 | 0.84 | NA | 0.78 | 0.89 | 0.85 |
| Bar | 0.85 | 0.85 | 0.89 | 0.89 | NA | 0.70 |

```
BiocParallel::bplapply(gbs, \(g) swap(g) |> GOC()) |> pairs2Kable6x3()
```

|  | Oki | Kum | Osa | Aom | Bar | Nor |
| --- | --- | --- | --- | --- | --- | --- |
| Oki | NA | 0.76 | 0.84 | 0.84 | 0.84 | 0.84 |
| Osa | 0.84 | 0.84 | NA | 0.77 | 0.89 | 0.88 |
| Bar | 0.85 | 0.85 | 0.89 | 0.89 | NA | 0.73 |

## Strand proportion index

Attempts to use pure proportions lead to scores drifting towards 0.5
when comparing fragmented assemblies, as the fragments are not
oriented.

```
strand_proportion_index <- function(gb) {
  gbl <- split(gb, droplevels(seqnames(gb)))
  # Calculate an index for each sequence feature
  idx <- sapply(gbl, \(x) {
    onPlus  <- sum(width(x[strand(x) == '+']))
    onMinus <- sum(width(x[strand(x) == '-']))
    onPlus / (onPlus + onMinus)
  })
  # Average by the sum of all widths
  weighted.mean(idx, sum(width(gbl)))
}
```

```
BiocParallel::bplapply(gbs,                 strand_proportion_index  ) |> pairs2Kable6x3()
```

|  | Oki | Kum | Osa | Aom | Bar | Nor |
| --- | --- | --- | --- | --- | --- | --- |
| Oki | NA | 0.73 | 0.49 | 0.47 | 0.47 | 0.49 |
| Osa | 0.49 | 0.50 | NA | 0.41 | 0.38 | 0.46 |
| Bar | 0.47 | 0.46 | 0.38 | 0.54 | NA | 0.59 |

```
BiocParallel::bplapply(gbs, \(g) swap(g) |> strand_proportion_index()) |> pairs2Kable6x3()
```

|  | Oki | Kum | Osa | Aom | Bar | Nor |
| --- | --- | --- | --- | --- | --- | --- |
| Oki | NA | 0.73 | 0.49 | 0.47 | 0.47 | 0.49 |
| Osa | 0.49 | 0.50 | NA | 0.41 | 0.38 | 0.46 |
| Bar | 0.47 | 0.46 | 0.38 | 0.54 | NA | 0.59 |

## Strand proportion majority index

One possible solution to the problem above could be to report the
highest proportion, so that the value returned is not sensitive to
orientation. But it scales the index between 0.5 and 1, which may be
counter-intuitive.

```
strand_proportion_majority_index <- function(gb) {
  gbl <- split(gb, droplevels(seqnames(gb)))
  # Calculate an index for each sequence feature
  idx <- sapply(gbl, \(x) {
    onPlus  <- sum(width(x[strand(x) == '+']))
    onMinus <- sum(width(x[strand(x) == '-']))
    p <- onPlus / (onPlus + onMinus)
    if(p < 0.5) p <- 1 - p
    p
  })
  # Average by the sum of all widths
  weighted.mean(idx, sum(width(gbl)))
}
```

```
BiocParallel::bplapply(gbs,                 strand_proportion_majority_index  ) |> pairs2Kable6x3()
```

|  | Oki | Kum | Osa | Aom | Bar | Nor |
| --- | --- | --- | --- | --- | --- | --- |
| Oki | NA | 0.76 | 0.52 | 0.53 | 0.55 | 0.53 |
| Osa | 0.52 | 0.53 | NA | 0.72 | 0.62 | 0.55 |
| Bar | 0.55 | 0.54 | 0.62 | 0.59 | NA | 0.59 |

```
BiocParallel::bplapply(gbs, \(g) swap(g) |> strand_proportion_majority_index()) |> pairs2Kable6x3()
```

|  | Oki | Kum | Osa | Aom | Bar | Nor |
| --- | --- | --- | --- | --- | --- | --- |
| Oki | NA | 0.91 | 0.52 | 0.55 | 0.55 | 0.66 |
| Osa | 0.52 | 0.53 | NA | 0.83 | 0.62 | 0.74 |
| Bar | 0.55 | 0.57 | 0.62 | 0.63 | NA | 0.91 |

## Contents

Developed by Aleksandra Bliznina, Michael Mansfield, Charles Plessy.

Site built with pkgdown 2.0.7.
