# Supplementary material for: Extreme genome scrambling in marine planktonic Oikopleura dioica cryptic species
Source: Genome Res. 2024 Mar;34(3):426–40. doi: 10.1101/gr.278295.123 (PMC11067885; doi:10.1101/gr.278295.123)
Supplement: Supplement 4 [file Supplemental_Data.zip › Supplemental_Data.tar.xz!/Supplemental_Data/articles/SquareWindowPlots_files/figure-html/Cbr_Cni_win-1.pdf]

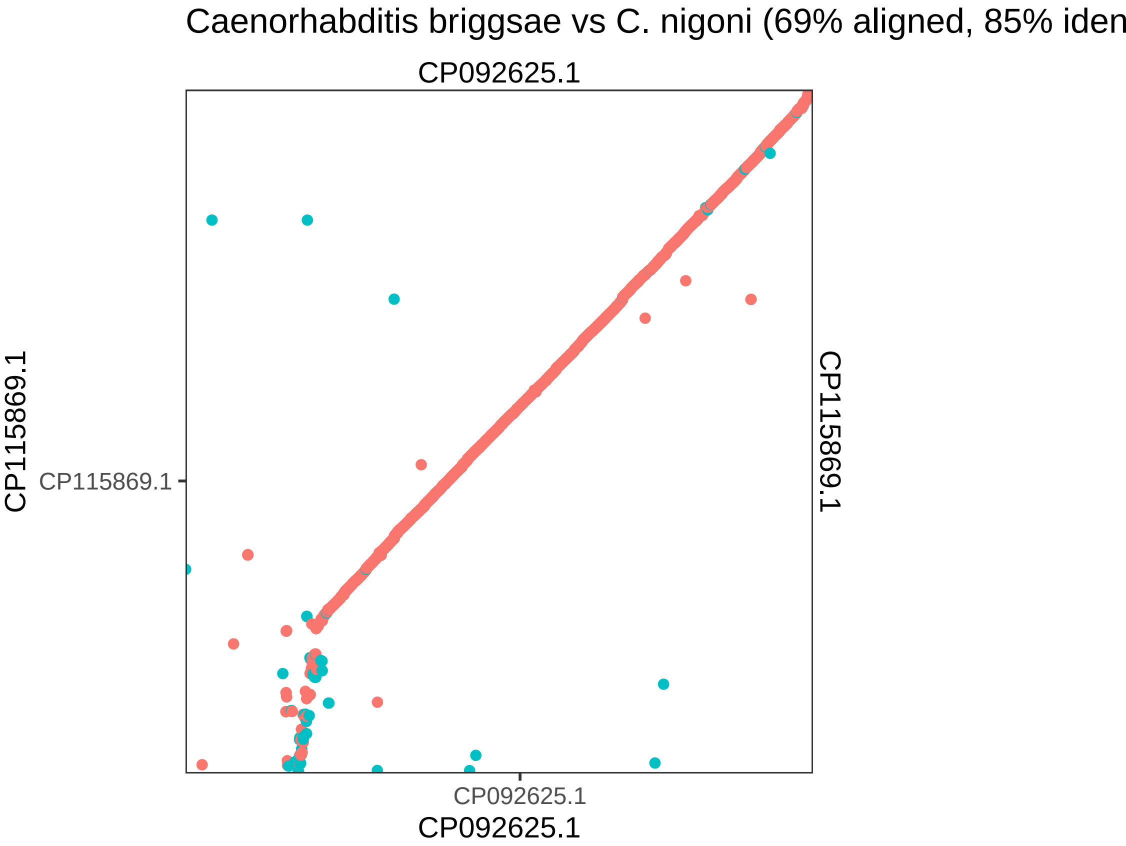

Caenorhabditis briggsae vs C. nigoni (69% aligned, 85% iden
CP092625.1
CP115869.1
CP115869.1
CP115869.1
CP092625.1
CP092625.1
