# Supplementary material for: Extreme genome scrambling in marine planktonic Oikopleura dioica cryptic species
Source: Genome Res. 2024 Mar;34(3):426–40. doi: 10.1101/gr.278295.123 (PMC11067885; doi:10.1101/gr.278295.123)
Supplement: Supplement 4 [file Supplemental_Data.zip › Supplemental_Data.tar.xz!/Supplemental_Data/articles/OperonDetection_files/figure-html/operon_summary_stats_plots-3.pdf]

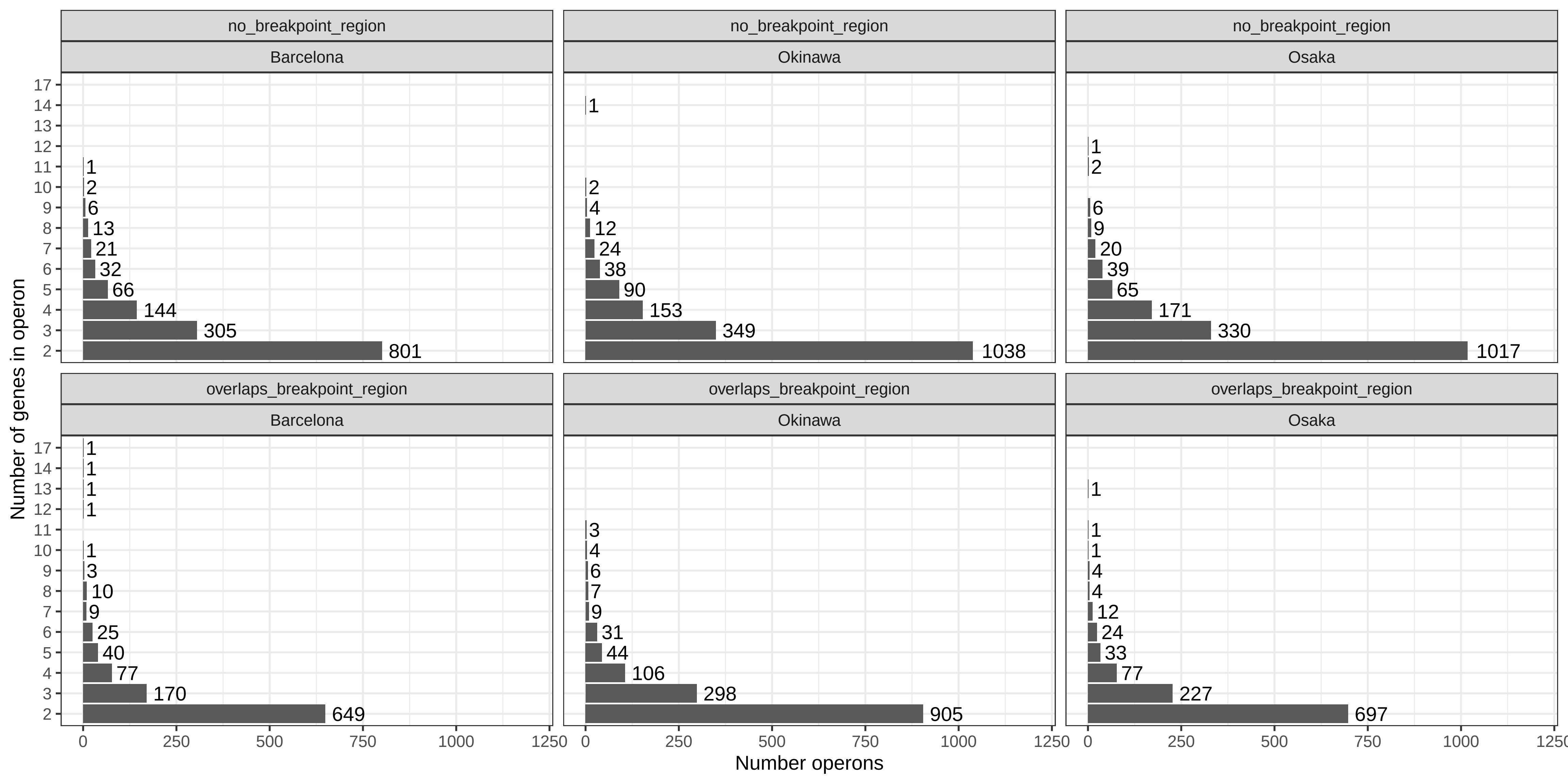

no_breakpoint_region
Barcelona
Okinawa
Osaka
overlaps_breakpoint_region
Number of genes in operon
Number operons
