# Supplementary material for: Extreme genome scrambling in marine planktonic Oikopleura dioica cryptic species
Source: Genome Res. 2024 Mar;34(3):426–40. doi: 10.1101/gr.278295.123 (PMC11067885; doi:10.1101/gr.278295.123)
Supplement: Supplement 4 [file Supplemental_Data.zip › Supplemental_Data.tar.xz!/Supplemental_Data/articles/OperonDetection_files/figure-html/operon_overlap_stats_plot-1.pdf]

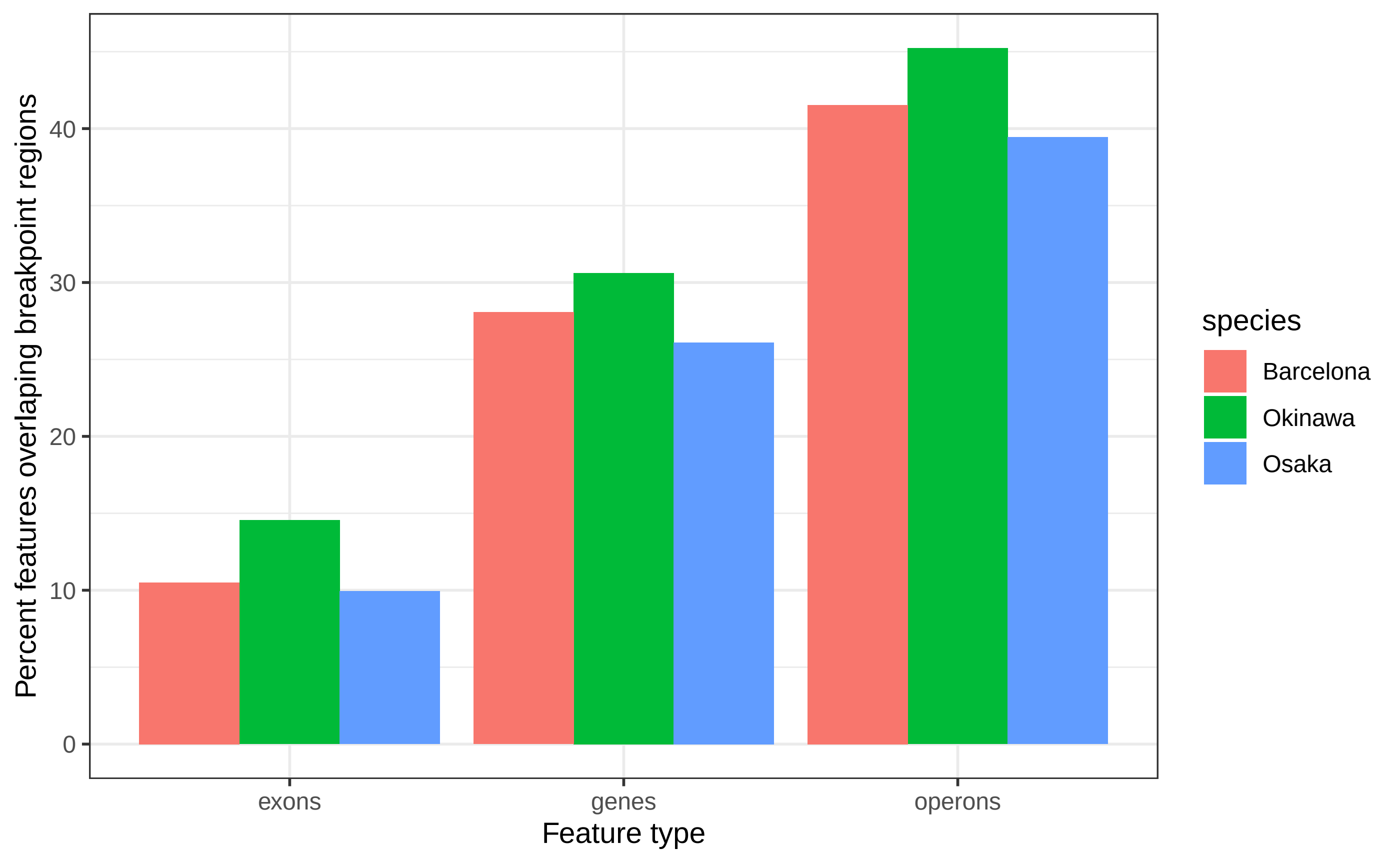
Percent features overlaping breakpoint regions
0
10
20
30
40
exons
genes
operons
Feature type
species
Barcelona
Okinawa
Osaka
