# Supplementary material for: Extreme genome scrambling in marine planktonic Oikopleura dioica cryptic species
Source: Genome Res. 2024 Mar;34(3):426–40. doi: 10.1101/gr.278295.123 (PMC11067885; doi:10.1101/gr.278295.123)
Supplement: Supplement 4 [file Supplemental_Data.zip › Supplemental_Data.tar.xz!/Supplemental_Data/articles/OperonDetection_files/figure-html/operon_summary_stats_plots-5.pdf]

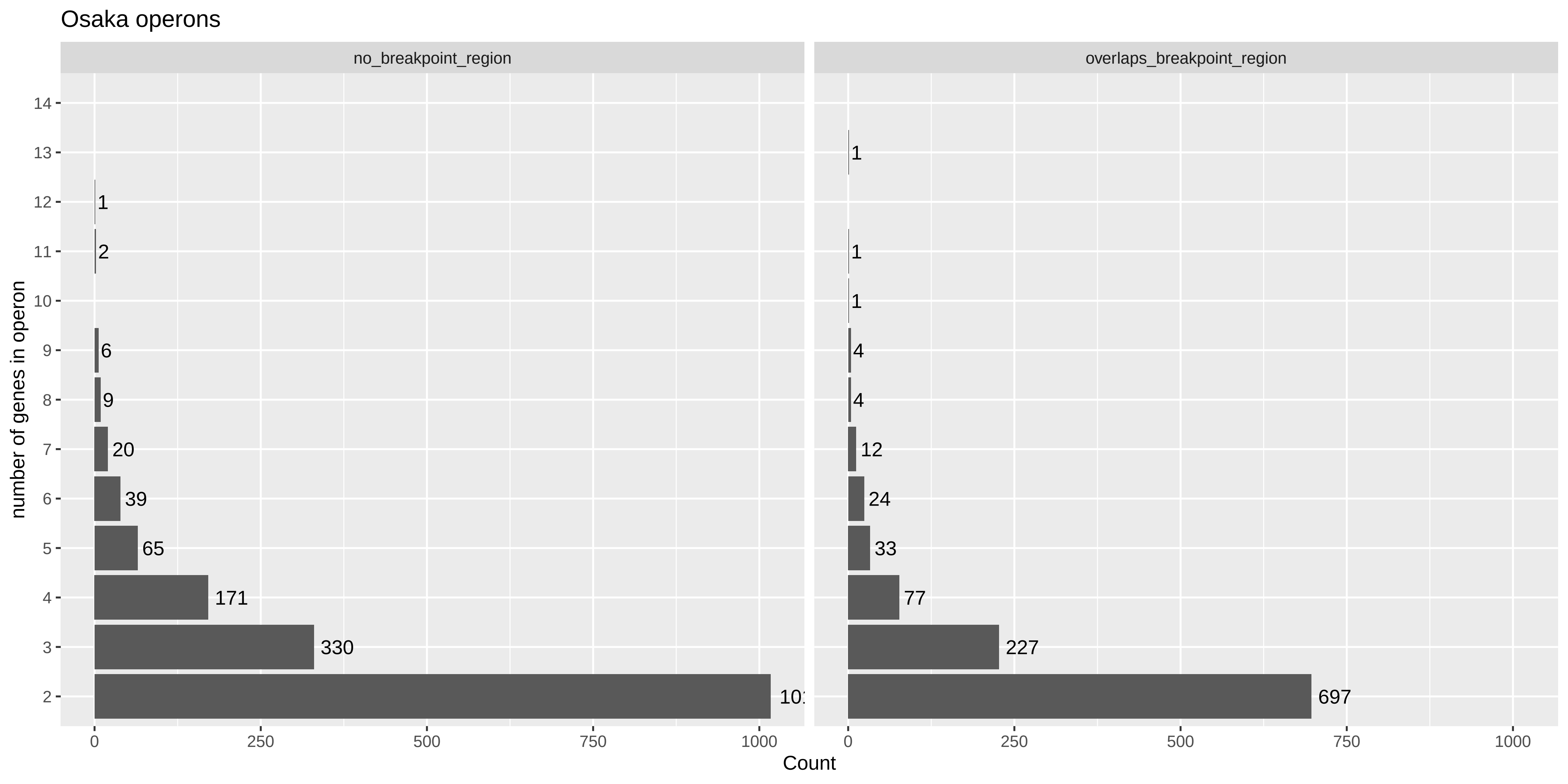

Osaka operons
no_breakpoint_region
overlaps_breakpoint_region
number of genes in operon
Count
14
13
12
11
10
9
8
7
6
5
4
3
2
0
250
500
750
1000
1
2
6
9
20
39
65
171
330
1
1
1
4
4
12
24
33
77
227
697
