# Supplementary material for: Extreme genome scrambling in marine planktonic Oikopleura dioica cryptic species
Source: Genome Res. 2024 Mar;34(3):426–40. doi: 10.1101/gr.278295.123 (PMC11067885; doi:10.1101/gr.278295.123)
Supplement: Supplement 4 [file Supplemental_Data.zip › Supplemental_Data.tar.xz!/Supplemental_Data/articles/OperonDetection_files/figure-html/operon_summary_stats_plots-4.pdf]

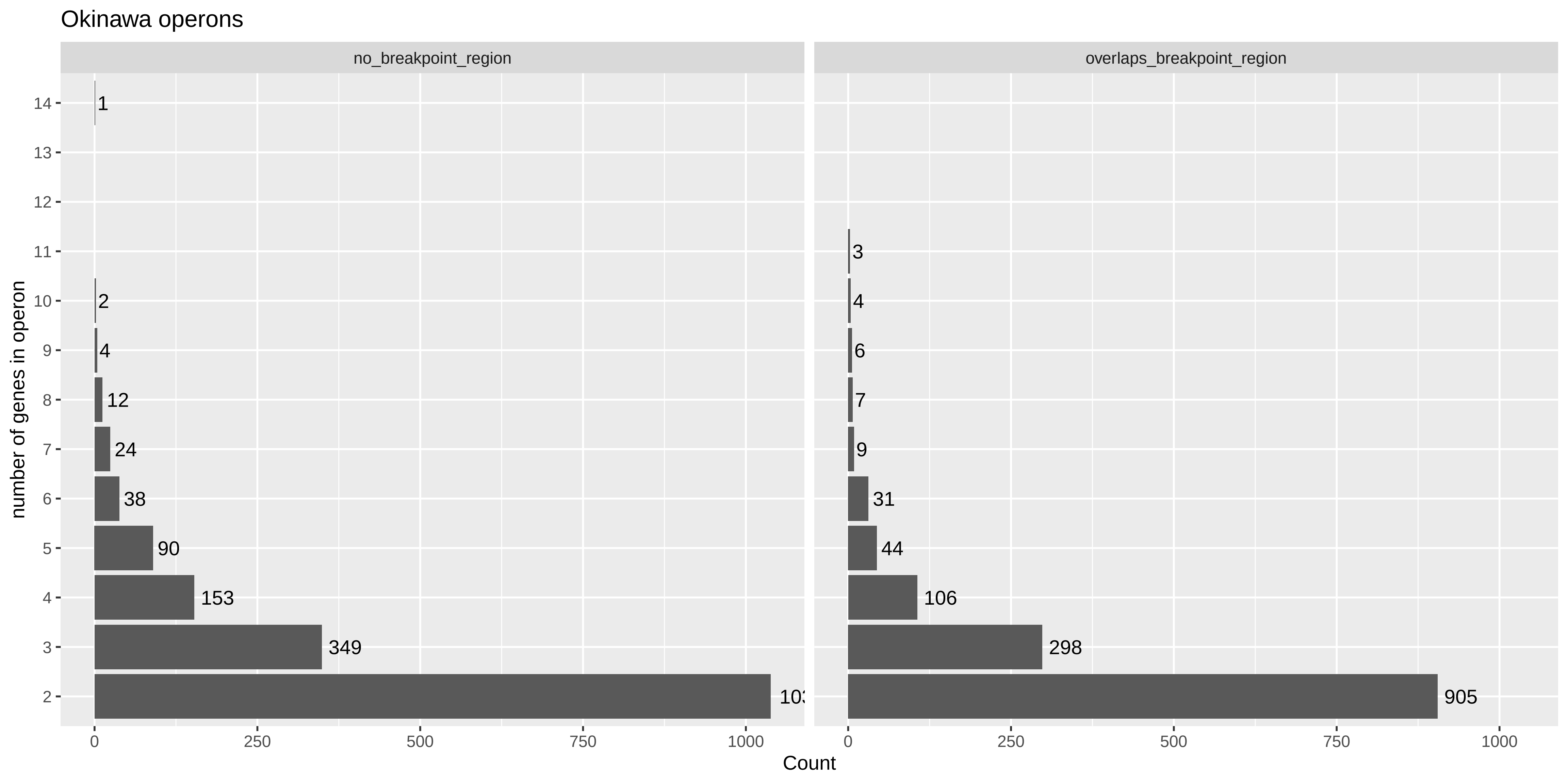

Okinawa operons
no_breakpoint_region
overlaps_breakpoint_region
number of genes in operon
Count
14
13
12
11
10
9
8
7
6
5
4
3
2
0
250
500
750
1000
1
2
4
12
24
38
90
153
349
3
4
6
7
9
31
44
106
298
905
