# Supplementary material for: Extreme genome scrambling in marine planktonic Oikopleura dioica cryptic species
Source: Genome Res. 2024 Mar;34(3):426–40. doi: 10.1101/gr.278295.123 (PMC11067885; doi:10.1101/gr.278295.123)
Supplement: Supplement 4 [file Supplemental_Data.zip › Supplemental_Data.tar.xz!/Supplemental_Data/articles/OperonDetection_files/figure-html/operon_summary_stats_plots-7.pdf]

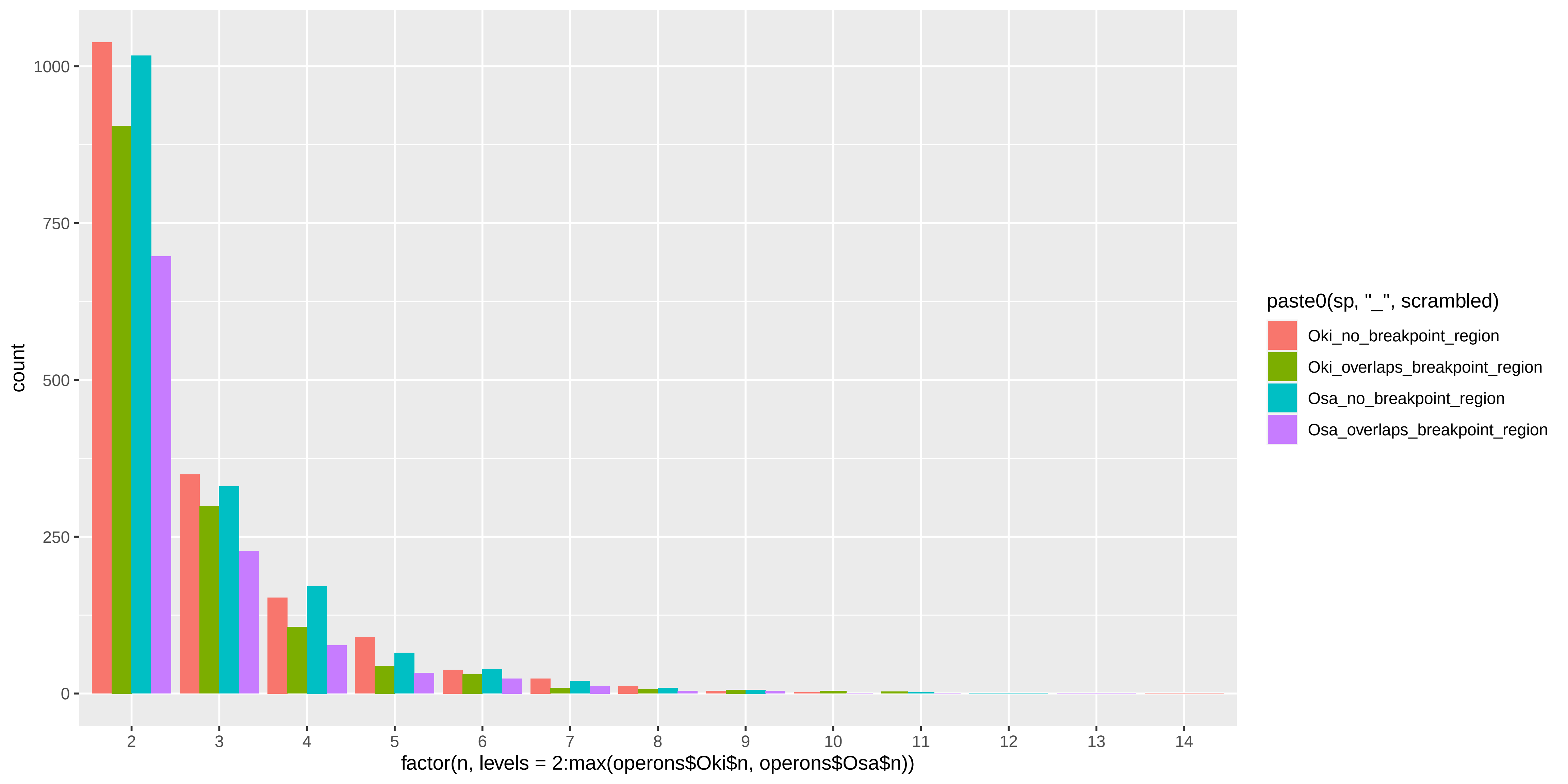

1000
750
500
250
0
count
2
3
4
5
6
7
8
9
10
11
12
13
14
factor(n, levels = 2:max(operonsOkin, operonsOsan))
paste0(sp, "_", scrambled)
Oki_no_breakpoint_region
Oki_overlaps_breakpoint_region
Osa_no_breakpoint_region
Osa_overlaps_breakpoint_region
