# Supplementary material for: Extreme genome scrambling in marine planktonic Oikopleura dioica cryptic species
Source: Genome Res. 2024 Mar;34(3):426–40. doi: 10.1101/gr.278295.123 (PMC11067885; doi:10.1101/gr.278295.123)
Supplement: Supplement 4 [file Supplemental_Data.zip › Supplemental_Data.tar.xz!/Supplemental_Data/articles/ChromosomePlots.html]

Chromosome-scale feature density plots • OikScrambling
 


  


 


Toggle navigation


OikScrambling
5.0.0

- Get started
- Data load
- Supplemental material
  - Figure 1A DNA line plots
  - Figure 1B chromosome parallel plots
  - Table 1 scrambling index
  - Figure 5 genome-wide feature patterns
  - Figure 7 DNA line plots
  - Figure 7 breakpoint number barplots
- Articles
  - Conserved non-coding elements
  - Chromosome-scale feature density plots
  - Modifying coalescent cutoffs
  - Regions interrupting colinearity
  - Distance Measures
  - Genomic features of the breakpoints
  - Genomic features of the breakpoints (heatmaps)
  - Inversions
  - Load genomic break data
  - Operon conservation analysis
  - Orthogroup syntenies
  - Alignment line plots, whole genomes
  - Region of interest: PAC3
  - Motif search and analysis
  - Whole-chromosome parallel plots
  - Width of the aligned and mapped regions.
  - Regions of interest
  - Repeat regions
  - Scaling breakpoints
  - Segment movement distances
  - Alignment line plots, 10×10 Mbp windows
  - Transcription factor binding sites
  - Tandem site duplications
  - Translocations
- Changelog
- Reference

# Chromosome-scale feature density plots

#### Michael Mansfield

#### Charles Plessy

#### 13 October, 2023

Source: `vignettes/ChromosomePlots.Rmd`

`ChromosomePlots.Rmd`

```
knitr::opts_chunk$set(cache = TRUE)
```

## Introduction

This vignette produced panels used in Figures 5 and S5, see its table
of contents to jump directly where they were computed.

In this vignette, we develop some of the materials needed to produce
plots that summarize genomic data at chromosome-scale.

The core functions used here are maintained in our
*GenomicBreaks* R package, which is fully documented at: https://oist.github.io/GenomicBreaks.

### Load packages

#### Load R packages and data

```
library('OikScrambling')      |> suppressPackageStartupMessages()
library('GenomicFeatures')    |> suppressPackageStartupMessages()
library('viridis')            |> suppressPackageStartupMessages()
library('dplyr')              |> suppressPackageStartupMessages()
library('plyranges')          |> suppressPackageStartupMessages()
library("patchwork")          |> suppressPackageStartupMessages()

genomes <- OikScrambling:::loadAllGenomes()
```

```
## Warning in runHook(".onLoad", env, package.lib, package): input string
## 'Génoscope' cannot be translated from 'ANSI_X3.4-1968' to UTF-8, but is valid
## UTF-8

## Warning in runHook(".onLoad", env, package.lib, package): input string
## 'Génoscope' cannot be translated from 'ANSI_X3.4-1968' to UTF-8, but is valid
## UTF-8
```

```
transcripts <- OikScrambling:::loadAllTranscriptsGR()
reps <- OikScrambling:::loadAllRepeats()
annots      <- OikScrambling:::loadAllAnnotations()   |> suppressWarnings()
load("BreakPoints.Rdata")
```

#### Creating chromosome-scale plots

In this vignette, we create the plumbing needed to generate summary
plots that show windows of genomic features on a chromosome scale.

##### Summarizing genomic windows of annotated `GRanges` objects

Below, I create some of the functions needed to produce these
plots.

```
# Make a BSgenome into a GRanges object, one range per chromosome
BSgenomeToGR <- function(genome) {
  as(seqinfo(genome), "GRanges")
}
(tmp <- genomes$Oki |> BSgenomeToGR())
```

```
## GRanges object with 19 ranges and 0 metadata columns:
##            seqnames     ranges strand
##               <Rle>  <IRanges>  <Rle>
##       chr1     chr1 1-14533022      *
##       chr2     chr2 1-16158756      *
##    chrUn_1  chrUn_1    1-11000      *
##    chrUn_2  chrUn_2    1-57957      *
##    chrUn_3  chrUn_3   1-131584      *
##        ...      ...        ...    ...
##   chrUn_13 chrUn_13     1-5739      *
##   chrUn_14 chrUn_14     1-2887      *
##        PAR      PAR 1-17092476      *
##        XSR      XSR 1-12959145      *
##        YSR      YSR  1-2916375      *
##   -------
##   seqinfo: 19 sequences from OKI2018.I69 genome
```

```
# Make a genome into a bunch of windows of length N
genomeGRToWindows <- function(gr, windowSize=10000) {
  tile(gr, w=windowSize) |> unlist() |> unname()
}
(tmp <- genomes$Oki |> BSgenomeToGR() |> genomeGRToWindows())
```

```
## GRanges object with 6437 ranges and 0 metadata columns:
##          seqnames          ranges strand
##             <Rle>       <IRanges>  <Rle>
##      [1]     chr1          1-9995      *
##      [2]     chr1      9996-19990      *
##      [3]     chr1     19991-29985      *
##      [4]     chr1     29986-39980      *
##      [5]     chr1     39981-49976      *
##      ...      ...             ...    ...
##   [6433]      YSR 2866438-2876424      *
##   [6434]      YSR 2876425-2886412      *
##   [6435]      YSR 2886413-2896399      *
##   [6436]      YSR 2896400-2906387      *
##   [6437]      YSR 2906388-2916375      *
##   -------
##   seqinfo: 19 sequences from an unspecified genome; no seqlengths
```

```
# Match ranges to a single bin by reducing each range to its center.
# Returns the index of the matched bins
matchToOneBin <- function(ranges, bins) {
  # Ensure that a range overlaps with only one bin
  safeRanges <- ranges |> resize(1, fix="center")
  o <- findOverlaps(safeRanges, bins, type = 'within')
  subjectHits(o)
}
(matchToOneBin(transcripts$Oki, tmp)) |> head()
```

```
## [1] 19 22 23 24 26 27
```

```
# Apply one function to data grouped by bins, and array it according to the
# original bins (that is, insert NAs for bins that contained no data).
# This function only works as intended if the bins fully tile the genome.
binApply <- function(f, bins, data, index, ...) {
  res <- rep(NA, length(bins))
  vals <-tapply(data, index, f, ...)
  res[as.numeric(names(vals))] <- unname(vals)
  res
}
(binApply(mean, tmp, transcripts$Oki$dNdS_GUIDANCE2, matchToOneBin(transcripts$Oki, tmp), na.rm = TRUE)) |> head(100)
```

```
##   [1]       NA       NA       NA       NA       NA       NA       NA       NA
##   [9]       NA       NA       NA       NA       NA       NA      NaN       NA
##  [17]       NA       NA      NaN      NaN       NA      NaN      NaN      NaN
##  [25]      NaN      NaN      NaN      NaN      NaN      NaN      NaN      NaN
##  [33]      NaN      NaN      NaN      NaN      NaN      NaN      NaN      NaN
##  [41]      NaN      NaN       NA      NaN      NaN      NaN       NA      NaN
##  [49]      NaN      NaN      NaN      NaN      NaN      NaN      NaN      NaN
##  [57]      NaN 0.012050      NaN      NaN      NaN       NA       NA      NaN
##  [65]      NaN 0.033770      NaN      NaN      NaN      NaN 0.005020 0.023600
##  [73]      NaN 0.035260 0.021470 0.023170 0.064320 0.034810      NaN      NaN
##  [81] 0.064100      NaN 0.035005      NaN      NaN 0.009240       NA      NaN
##  [89] 0.018220 0.034370      NaN      NaN 0.039270      NaN      NaN      NaN
##  [97] 0.055030 0.010370      NaN 0.072860
```

```
# Intersect each genome window with some other GRange (meta, as in metadata)
metaPerWindow <- function(genomeWindows, meta, meta_prefix=NULL) {
  idx <- matchToOneBin(meta, genomeWindows)
  for (name in colnames(mcols(meta))) {
    data <- mcols(meta)[[name]]
    if(is.numeric(data)) {
      mcols(genomeWindows)[[paste0(meta_prefix, name, ".count")]]       <- binApply(length, genomeWindows, data, idx)
      mcols(genomeWindows)[[paste0(meta_prefix, name, ".median")]]      <- binApply(median, genomeWindows, data, idx, na.rm = TRUE)
      mcols(genomeWindows)[[paste0(meta_prefix, name, ".mean")]]        <- binApply(mean,   genomeWindows, data, idx, na.rm = TRUE)
      mcols(genomeWindows)[[paste0(meta_prefix, name, ".sd")]]          <- binApply(sd,     genomeWindows, data, idx, na.rm = TRUE)
    } else if(is.character(data) ) {
      mcols(genomeWindows)[[paste0(meta_prefix, name, ".count")]]       <- binApply(length, genomeWindows, data, idx)
    } else if(is.factor(data)) {
      mcols(genomeWindows)[[paste0(meta_prefix, name, ".count.total")]] <- binApply(length, genomeWindows, data, idx)
      if(length(levels(data)) > 1) {
        binned_list_of_tables <- binApply(table,  genomeWindows, data, idx)
        binned_DataFrame <- do.call(rbind, binned_list_of_tables) |> DataFrame()
        colnames(binned_DataFrame) <- paste0(meta_prefix, name, ".", colnames(binned_DataFrame), ".count.total")
        mcols(genomeWindows) <- cbind(mcols(genomeWindows), binned_DataFrame)
      }
    }
  }
  genomeWindows
}
(metaPerWindow(genomeWindows=tmp, transcripts$Oki, meta_prefix = 'transcripts.'))[120:130]
```

```
## GRanges object with 11 ranges and 17 metadata columns:
##        seqnames          ranges strand | transcripts.tx_id.count
##           <Rle>       <IRanges>  <Rle> |               <integer>
##    [1]     chr1 1189429-1199424      * |                       4
##    [2]     chr1 1199425-1209419      * |                       3
##    [3]     chr1 1209420-1219414      * |                       3
##    [4]     chr1 1219415-1229409      * |                    <NA>
##    [5]     chr1 1229410-1239404      * |                       1
##    [6]     chr1 1239405-1249400      * |                       3
##    [7]     chr1 1249401-1259395      * |                       3
##    [8]     chr1 1259396-1269390      * |                       3
##    [9]     chr1 1269391-1279385      * |                       5
##   [10]     chr1 1279386-1289380      * |                       6
##   [11]     chr1 1289381-1299376      * |                       2
##        transcripts.tx_id.median transcripts.tx_id.mean transcripts.tx_id.sd
##                       <numeric>              <numeric>            <numeric>
##    [1]                    121.5                649.000             1056.000
##    [2]                    124.0                124.000                1.000
##    [3]                   2234.0               1531.667             1217.343
##    [4]                       NA                     NA                   NA
##    [5]                    127.0                127.000                   NA
##    [6]                    129.0                831.000             1216.766
##    [7]                    131.0                131.000                1.000
##    [8]                    134.0                834.667             1214.456
##    [9]                    137.0                556.800              939.820
##   [10]                    141.5                490.667              856.506
##   [11]                   1192.0               1192.000             1482.096
##        transcripts.tx_name.count transcripts.dNdS_GUIDANCE2.count
##                        <integer>                        <integer>
##    [1]                         4                                4
##    [2]                         3                                3
##    [3]                         3                                3
##    [4]                      <NA>                             <NA>
##    [5]                         1                                1
##    [6]                         3                                3
##    [7]                         3                                3
##    [8]                         3                                3
##    [9]                         5                                5
##   [10]                         6                                6
##   [11]                         2                                2
##        transcripts.dNdS_GUIDANCE2.median transcripts.dNdS_GUIDANCE2.mean
##                                <numeric>                       <numeric>
##    [1]                          0.031520                        0.031520
##    [2]                                NA                             NaN
##    [3]                          0.024815                        0.024815
##    [4]                                NA                              NA
##    [5]                                NA                             NaN
##    [6]                                NA                             NaN
##    [7]                                NA                             NaN
##    [8]                          0.065760                        0.065760
##    [9]                          0.010860                        0.010860
##   [10]                                NA                             NaN
##   [11]                                NA                             NaN
##        transcripts.dNdS_GUIDANCE2.sd transcripts.dNdS_HmmCleaner.count
##                            <numeric>                         <integer>
##    [1]                     0.0240275                                 4
##    [2]                            NA                                 3
##    [3]                     0.0067953                                 3
##    [4]                            NA                              <NA>
##    [5]                            NA                                 1
##    [6]                            NA                                 3
##    [7]                            NA                                 3
##    [8]                            NA                                 3
##    [9]                            NA                                 5
##   [10]                            NA                                 6
##   [11]                            NA                                 2
##        transcripts.dNdS_HmmCleaner.median transcripts.dNdS_HmmCleaner.mean
##                                 <numeric>                        <numeric>
##    [1]                           0.028550                         0.028550
##    [2]                                 NA                              NaN
##    [3]                           0.023425                         0.023425
##    [4]                                 NA                               NA
##    [5]                                 NA                              NaN
##    [6]                                 NA                              NaN
##    [7]                                 NA                              NaN
##    [8]                           0.043460                         0.043460
##    [9]                           0.010860                         0.010860
##   [10]                                 NA                              NaN
##   [11]                                 NA                              NaN
##        transcripts.dNdS_HmmCleaner.sd transcripts.dNdS_PRANK.count
##                             <numeric>                    <integer>
##    [1]                     0.01982727                            4
##    [2]                             NA                            3
##    [3]                     0.00876105                            3
##    [4]                             NA                         <NA>
##    [5]                             NA                            1
##    [6]                             NA                            3
##    [7]                             NA                            3
##    [8]                             NA                            3
##    [9]                             NA                            5
##   [10]                             NA                            6
##   [11]                             NA                            2
##        transcripts.dNdS_PRANK.median transcripts.dNdS_PRANK.mean
##                            <numeric>                   <numeric>
##    [1]                      0.029315                    0.029315
##    [2]                            NA                         NaN
##    [3]                      0.026540                    0.026540
##    [4]                            NA                          NA
##    [5]                            NA                         NaN
##    [6]                            NA                         NaN
##    [7]                            NA                         NaN
##    [8]                      0.067020                    0.067020
##    [9]                      0.010860                    0.010860
##   [10]                            NA                         NaN
##   [11]                            NA                         NaN
##        transcripts.dNdS_PRANK.sd
##                        <numeric>
##    [1]                0.02090915
##    [2]                        NA
##    [3]                0.00435578
##    [4]                        NA
##    [5]                        NA
##    [6]                        NA
##    [7]                        NA
##    [8]                        NA
##    [9]                        NA
##   [10]                        NA
##   [11]                        NA
##   -------
##   seqinfo: 19 sequences from an unspecified genome; no seqlengths
```

```
# Function to do everything
grWindows <- function(genome, meta, windowSize=10000, meta_prefix=NULL){
  genome <- BSgenomeToGR(genome)                                 # Make GRanges from genome
  genome <- genomeGRToWindows(genome, windowSize=windowSize)     # Make a windowed GRange from chromosome GRanges
  mpw <-    metaPerWindow(genome, meta, meta_prefix=meta_prefix) # Make metadata column windows and intersect
  mpw
}
(grWindows(genomes$Oki, transcripts$Oki, meta_prefix = "transcripts"))[120:130]
```

```
## GRanges object with 11 ranges and 17 metadata columns:
##        seqnames          ranges strand | transcriptstx_id.count
##           <Rle>       <IRanges>  <Rle> |              <integer>
##    [1]     chr1 1189429-1199424      * |                      4
##    [2]     chr1 1199425-1209419      * |                      3
##    [3]     chr1 1209420-1219414      * |                      3
##    [4]     chr1 1219415-1229409      * |                   <NA>
##    [5]     chr1 1229410-1239404      * |                      1
##    [6]     chr1 1239405-1249400      * |                      3
##    [7]     chr1 1249401-1259395      * |                      3
##    [8]     chr1 1259396-1269390      * |                      3
##    [9]     chr1 1269391-1279385      * |                      5
##   [10]     chr1 1279386-1289380      * |                      6
##   [11]     chr1 1289381-1299376      * |                      2
##        transcriptstx_id.median transcriptstx_id.mean transcriptstx_id.sd
##                      <numeric>             <numeric>           <numeric>
##    [1]                   121.5               649.000            1056.000
##    [2]                   124.0               124.000               1.000
##    [3]                  2234.0              1531.667            1217.343
##    [4]                      NA                    NA                  NA
##    [5]                   127.0               127.000                  NA
##    [6]                   129.0               831.000            1216.766
##    [7]                   131.0               131.000               1.000
##    [8]                   134.0               834.667            1214.456
##    [9]                   137.0               556.800             939.820
##   [10]                   141.5               490.667             856.506
##   [11]                  1192.0              1192.000            1482.096
##        transcriptstx_name.count transcriptsdNdS_GUIDANCE2.count
##                       <integer>                       <integer>
##    [1]                        4                               4
##    [2]                        3                               3
##    [3]                        3                               3
##    [4]                     <NA>                            <NA>
##    [5]                        1                               1
##    [6]                        3                               3
##    [7]                        3                               3
##    [8]                        3                               3
##    [9]                        5                               5
##   [10]                        6                               6
##   [11]                        2                               2
##        transcriptsdNdS_GUIDANCE2.median transcriptsdNdS_GUIDANCE2.mean
##                               <numeric>                      <numeric>
##    [1]                         0.031520                       0.031520
##    [2]                               NA                            NaN
##    [3]                         0.024815                       0.024815
##    [4]                               NA                             NA
##    [5]                               NA                            NaN
##    [6]                               NA                            NaN
##    [7]                               NA                            NaN
##    [8]                         0.065760                       0.065760
##    [9]                         0.010860                       0.010860
##   [10]                               NA                            NaN
##   [11]                               NA                            NaN
##        transcriptsdNdS_GUIDANCE2.sd transcriptsdNdS_HmmCleaner.count
##                           <numeric>                        <integer>
##    [1]                    0.0240275                                4
##    [2]                           NA                                3
##    [3]                    0.0067953                                3
##    [4]                           NA                             <NA>
##    [5]                           NA                                1
##    [6]                           NA                                3
##    [7]                           NA                                3
##    [8]                           NA                                3
##    [9]                           NA                                5
##   [10]                           NA                                6
##   [11]                           NA                                2
##        transcriptsdNdS_HmmCleaner.median transcriptsdNdS_HmmCleaner.mean
##                                <numeric>                       <numeric>
##    [1]                          0.028550                        0.028550
##    [2]                                NA                             NaN
##    [3]                          0.023425                        0.023425
##    [4]                                NA                              NA
##    [5]                                NA                             NaN
##    [6]                                NA                             NaN
##    [7]                                NA                             NaN
##    [8]                          0.043460                        0.043460
##    [9]                          0.010860                        0.010860
##   [10]                                NA                             NaN
##   [11]                                NA                             NaN
##        transcriptsdNdS_HmmCleaner.sd transcriptsdNdS_PRANK.count
##                            <numeric>                   <integer>
##    [1]                    0.01982727                           4
##    [2]                            NA                           3
##    [3]                    0.00876105                           3
##    [4]                            NA                        <NA>
##    [5]                            NA                           1
##    [6]                            NA                           3
##    [7]                            NA                           3
##    [8]                            NA                           3
##    [9]                            NA                           5
##   [10]                            NA                           6
##   [11]                            NA                           2
##        transcriptsdNdS_PRANK.median transcriptsdNdS_PRANK.mean
##                           <numeric>                  <numeric>
##    [1]                     0.029315                   0.029315
##    [2]                           NA                        NaN
##    [3]                     0.026540                   0.026540
##    [4]                           NA                         NA
##    [5]                           NA                        NaN
##    [6]                           NA                        NaN
##    [7]                           NA                        NaN
##    [8]                     0.067020                   0.067020
##    [9]                     0.010860                   0.010860
##   [10]                           NA                        NaN
##   [11]                           NA                        NaN
##        transcriptsdNdS_PRANK.sd
##                       <numeric>
##    [1]               0.02090915
##    [2]                       NA
##    [3]               0.00435578
##    [4]                       NA
##    [5]                       NA
##    [6]                       NA
##    [7]                       NA
##    [8]                       NA
##    [9]                       NA
##   [10]                       NA
##   [11]                       NA
##   -------
##   seqinfo: 19 sequences from an unspecified genome; no seqlengths
```

## Genomic features

### Transcripts

In the following sections we annotate and initialize the
`chrw` object, which is a `SimpleList()`
containing windowed chromosome regions.

We use these windows to evaluate the distribution of various features
across the chromosomes. This section concerns one-dimensional
features.

```
windowSize <- 2e5
bins_Oki <- genomes$Oki |> BSgenomeToGR() |> genomeGRToWindows(windowSize)
bins_Osa <- genomes$Osa |> BSgenomeToGR() |> genomeGRToWindows(windowSize)
bins_Bar <- genomes$Bar |> BSgenomeToGR() |> genomeGRToWindows(windowSize)

transcripts$Oki$tx_len <- width(transcripts$Oki)
transcripts$Osa$tx_len <- width(transcripts$Osa)
transcripts$Bar$tx_len <- width(transcripts$Bar)

chrw <- SimpleList()
chrw$Oki <- grWindows(genome=genomes$Oki, meta=transcripts$Oki, windowSize=windowSize, meta_prefix = 'transcripts.')
chrw$Oki <- flagLongShort(chrw$Oki, longShort$OKI2018.I69)
chrw$Osa <- grWindows(genome=genomes$Osa, meta=transcripts$Osa, windowSize=windowSize, meta_prefix = 'transcripts.')
chrw$Osa <- flagLongShort(chrw$Osa, longShort$OSKA2016v1.9)
chrw$Bar <- grWindows(genome=genomes$Bar, meta=transcripts$Bar, windowSize=windowSize, meta_prefix = 'transcripts.')
chrw$Bar <- flagLongShort(chrw$Bar, longShort$Bar2.p4)
```

```
per_chrom_plot <- function(x, Y = FALSE) {
  seqnames_to_keep <- c('chr1', 'Chr1', 'chr2', 'Chr2', 'PAR', 'XSR')
  if (Y) seqnames_to_keep <- c(seqnames_to_keep, 'YSR')
  x |>
    plyranges::filter(seqnames %in% seqnames_to_keep) |>
    as.data.frame() |>
    ggplot() +
      aes(x = start, col = Arm) +
      geom_point() +
      facet_wrap(~seqnames, nrow = 1) +
      geom_smooth(method = 'loess', formula = y ~ x,  method.args = list(family = "symmetric")) +
      theme_bw()
}

# Create a list of plots. We save some of these plots for faceting later.
plots <- list()
```

```
# Density of transcript names (i.e., gene counts).
plots$Oki$transcripts$density <- chrw$Oki |> per_chrom_plot() + aes(y=transcripts.tx_name.count) + ggtitle(paste("Okinawa: transcripts per window (size = ", windowSize/1000, " kbp)", sep=""))

# dN/dS
plots$Oki$transcripts$dnds <- chrw$Oki |> per_chrom_plot() + aes(y=transcripts.dNdS_PRANK.median) + ggtitle(paste("Okinawa: dN/dS per window (size = ", windowSize/1000, " kbp)", sep=""))

#Lengths
plots$Oki$transcripts$lengths <- chrw$Oki |> per_chrom_plot() + aes(y=transcripts.tx_len.median) + ggtitle(paste("Okinawa: median transcript length per window (size = ", windowSize/1000, " kbp)", sep=""))
```

#### Transcript-related chromosome plots, windows: Osaka

```
# Density of transcript names (i.e., gene counts).
plots$Osa$transcripts$density <- chrw$Osa |> per_chrom_plot() + aes(y=transcripts.tx_name.count) + ggtitle(paste("Osaka: transcripts per window (size = ", windowSize/1000, " kbp)", sep=""))

# dN/dS
plots$Osa$transcripts$dnds <- chrw$Osa |> per_chrom_plot() + aes(y=transcripts.dNdS_PRANK.median) + ggtitle(paste("Osaka: dN/dS per window (size = ", windowSize/1000, " kbp)", sep=""))

#Lengths
plots$Osa$transcripts$lengths <- chrw$Osa |> per_chrom_plot() + aes(y=transcripts.tx_len.median) + ggtitle(paste("Osaka: median transcript length per window (size = ", windowSize/1000, " kbp)", sep=""))
```

#### Transcript-related chromosome plots, windows: Barcelona

```
# Density of transcript names (i.e., gene counts).
plots$Bar$transcripts$density <- chrw$Bar |> per_chrom_plot() + aes(y=transcripts.tx_name.count) + ggtitle(paste("Barcelona: transcripts per window (size = ", windowSize/1000, " kbp)", sep=""))

# dN/dS
plots$Bar$transcripts$dnds <- chrw$Bar |> per_chrom_plot() + aes(y=transcripts.dNdS_PRANK.median) + ggtitle(paste("Barcelona: dN/dS per window (size = ", windowSize/1000, " kbp)", sep=""))

#Lengths
plots$Bar$transcripts$lengths <- chrw$Bar |> per_chrom_plot() + aes(y=transcripts.tx_len.median) + ggtitle(paste("Barcelona: median transcript length per window (size = ", windowSize/1000, " kbp)", sep=""))
```

#### Transcript-related chromosome plots, windows: Joined plots

```
plots$Oki$transcripts$density / plots$Osa$transcripts$density / plots$Bar$transcripts$density
```

```
plots$Oki$transcripts$dnds / plots$Osa$transcripts$dnds / plots$Bar$transcripts$dnds
```

```
plots$Oki$transcripts$lengths / plots$Osa$transcripts$lengths / plots$Bar$transcripts$lengths
```

### Operons

In this section we add operon data to the `chrw` object.
Since the windows are the same for every genome, this is simple
accomplished with a `cbind` operation to the windowed
object’s `mcols`.

```
# Get operons for each species
operons <- SimpleList()
operons$Oki <- OikScrambling:::calcOperons(annots$Oki |> genes(), window = 500)
operons$Osa <- OikScrambling:::calcOperons(annots$Osa |> genes(), window = 500)
operons$Bar <- OikScrambling:::calcOperons(annots$Bar |> genes(), window = 500)

# Since the coordinates of the windows are the same, you can simply cbind more metadata columns to the data frame.
mcols(chrw$Oki) <- cbind(mcols(chrw$Oki), mcols(grWindows(genome=genomes$Oki, meta=operons$Oki, windowSize=windowSize, meta_prefix = 'operons.'))) |> suppressWarnings()

mcols(chrw$Osa) <- cbind(mcols(chrw$Osa), mcols(grWindows(genome=genomes$Osa, meta=operons$Osa, windowSize=windowSize, meta_prefix = 'operons.'))) |> suppressWarnings()

mcols(chrw$Bar) <- cbind(mcols(chrw$Bar), mcols(grWindows(genome=genomes$Bar, meta=operons$Bar, windowSize=windowSize, meta_prefix = 'operons.')))
```

#### Operon-related chromosome plots, windows: Okinawa

```
plots$Oki$operons$density <- chrw$Oki |> per_chrom_plot() + aes(y=operons.n.count) + ggtitle(paste("Okinawa: operons per window (size = ", windowSize/1000, " kbp)", sep=""))

plots$Oki$operons$length  <- chrw$Oki |> per_chrom_plot() + aes(y=operons.n.mean)  + ggtitle(paste("Okinawa: mean operon size per window (size = ", windowSize/1000, " kbp)", sep=""))
```

#### Operon-related chromosome plots, windows: Osaka

```
plots$Osa$operons$density <- chrw$Osa |> per_chrom_plot() + aes(y=operons.n.count) + ggtitle(paste("Osaka: operons per window (size = ", windowSize/1000, " kbp)", sep=""))

plots$Osa$operons$length  <- chrw$Osa |> per_chrom_plot() + aes(y=operons.n.mean)  + ggtitle(paste("Osaka: mean operon size per window (size = ", windowSize/1000, " kbp)", sep=""))
```

#### Operon-related chromosome plots, windows: Barcelona

```
plots$Bar$operons$density <- chrw$Bar |> per_chrom_plot() + aes(y=operons.n.count) + ggtitle(paste("Barcelona: operons per window (size = ", windowSize/1000, " kbp)", sep=""))

plots$Bar$operons$length  <- chrw$Bar |> per_chrom_plot() + aes(y=operons.n.mean)  + ggtitle(paste("Barcelona: mean operon size per window (size = ", windowSize/1000, " kbp)", sep=""))
```

#### Operon-related chromosome plots, windows: Joined plots

```
plots$Oki$operons$density / plots$Osa$operons$density / plots$Bar$operons$density
```

```
plots$Oki$operons$length  / plots$Osa$operons$length  / plots$Bar$operons$length
```

### Repeats

Like operons, it is simple to add repeat density, since the X
coordinates (`start` and `end` positions of a
`GRanges` are shared).

```
mcols(chrw$Oki) <- cbind(mcols(chrw$Oki), mcols(grWindows(genome=genomes$Oki, meta=reps$Oki, windowSize=windowSize, meta_prefix = 'repeats.')))
mcols(chrw$Osa) <- cbind(mcols(chrw$Osa), mcols(grWindows(genome=genomes$Osa, meta=reps$Osa, windowSize=windowSize, meta_prefix = 'repeats.')))
mcols(chrw$Bar) <- cbind(mcols(chrw$Bar), mcols(grWindows(genome=genomes$Bar, meta=reps$Bar, windowSize=windowSize, meta_prefix = 'repeats.')))
```

```
plots$Oki$repeats$density <- chrw$Oki |> per_chrom_plot(Y=TRUE) + aes(y=repeats.Class.count.total) +  ggtitle(paste("Okinawa: repeats per window (size = ", windowSize/1000, " kbp)", sep=""))

plots$Osa$repeats$density <- chrw$Osa |> per_chrom_plot(Y=TRUE) + aes(y=repeats.Class.count.total) +  ggtitle(paste("Osaka: repeats per window (size = ", windowSize/1000, " kbp)", sep=""))

plots$Bar$repeats$density <- chrw$Bar |> per_chrom_plot(Y=TRUE) + aes(y=repeats.Class.count.total) +  ggtitle(paste("Barcelona: repeats per window (size = ", windowSize/1000, " kbp)", sep=""))
```

#### Supplemental figure S5

```
### Repeat-related chromosome plots, windows: Joined plots
(plots$Oki$repeats$density / plots$Osa$repeats$density / plots$Bar$repeats$density)
```

See also the repeat classes in the Extra section.

### `GenomicBreaks` objects: Scores and widths

In the next sections, we explore the distribution of alignment
parameters (scores and widths) for different populations vs. chromosomal
position.

```
# Okinawa
chrw$Oki$Oki_Osa.score.mean <- binApply(mean, na.rm = T, bins_Oki, score(gbs$Oki_Osa), matchToOneBin(gbs$Oki_Osa, bins_Oki))
chrw$Oki$Oki_Aom.score.mean <- binApply(mean, na.rm = T, bins_Oki, score(gbs$Oki_Aom), matchToOneBin(gbs$Oki_Aom, bins_Oki))
chrw$Oki$Oki_Bar.score.mean <- binApply(mean, na.rm = T, bins_Oki, score(gbs$Oki_Bar), matchToOneBin(gbs$Oki_Bar, bins_Oki))
chrw$Oki$Oki_Nor.score.mean <- binApply(mean, na.rm = T, bins_Oki, score(gbs$Oki_Nor), matchToOneBin(gbs$Oki_Nor, bins_Oki))

# Osaka
chrw$Osa$Osa_Oki.score.mean <- binApply(mean, na.rm = T, bins_Osa, score(gbs$Osa_Oki), matchToOneBin(gbs$Osa_Oki, bins_Osa))
chrw$Osa$Osa_Kum.score.mean <- binApply(mean, na.rm = T, bins_Osa, score(gbs$Osa_Kum), matchToOneBin(gbs$Osa_Kum, bins_Osa))
chrw$Osa$Osa_Bar.score.mean <- binApply(mean, na.rm = T, bins_Osa, score(gbs$Osa_Bar), matchToOneBin(gbs$Osa_Bar, bins_Osa))
chrw$Osa$Osa_Nor.score.mean <- binApply(mean, na.rm = T, bins_Osa, score(gbs$Osa_Nor), matchToOneBin(gbs$Osa_Nor, bins_Osa))

# Barcelona
chrw$Bar$Bar_Osa.score.mean <- binApply(mean, na.rm = T, bins_Bar, score(gbs$Bar_Osa), matchToOneBin(gbs$Bar_Osa, bins_Bar))
chrw$Bar$Bar_Aom.score.mean <- binApply(mean, na.rm = T, bins_Bar, score(gbs$Bar_Aom), matchToOneBin(gbs$Bar_Aom, bins_Bar))
chrw$Bar$Bar_Oki.score.mean <- binApply(mean, na.rm = T, bins_Bar, score(gbs$Bar_Oki), matchToOneBin(gbs$Bar_Oki, bins_Bar))
chrw$Bar$Bar_Kum.score.mean <- binApply(mean, na.rm = T, bins_Bar, score(gbs$Bar_Kum), matchToOneBin(gbs$Bar_Kum, bins_Bar))
```

#### Score-related chromosome plots, windows: Joined plots

```
plots$Oki$scores$oki_osa <- chrw$Oki |> per_chrom_plot(Y=TRUE) + aes(y=Oki_Osa.score.mean) +  ggtitle("Oki-Osa: Chromosomal position vs. alignment score")

plots$Oki$scores$oki_bar <- chrw$Oki |> per_chrom_plot(Y=TRUE) + aes(y=Oki_Bar.score.mean) +  ggtitle("Oki-Bar: Chromosomal position vs. alignment score")

plots$Oki$scores$combined <- chrw$Oki |>
  as.data.frame() |>
  subset(select=c("seqnames", "start", "end", "Oki_Osa.score.mean", "Oki_Aom.score.mean", "Oki_Bar.score.mean", "Oki_Nor.score.mean" )) |>
  plyranges::filter(seqnames %in% c('chr1', 'chr2', 'PAR', 'XSR')) |>
  tidyr::pivot_longer(c("Oki_Osa.score.mean", "Oki_Aom.score.mean", "Oki_Bar.score.mean", "Oki_Nor.score.mean")) |>
  ggplot() +
    aes(start, value, col = name) +
    facet_wrap(~seqnames, nrow = 1) +
    geom_smooth(span = 0.25, se = FALSE) +
    ggtitle("Okinawa: Chromosomal position vs. alignment score")
```

```
plots$Osa$scores$combined <- chrw$Osa |>
  as.data.frame() |>
  subset(select=c("seqnames", "start", "end", "Osa_Oki.score.mean", "Osa_Kum.score.mean", "Osa_Bar.score.mean", "Osa_Nor.score.mean" )) |>
  plyranges::filter(seqnames %in% c('Chr1', 'Chr2', 'PAR', 'XSR')) |>
  tidyr::pivot_longer(c("Osa_Oki.score.mean", "Osa_Kum.score.mean", "Osa_Bar.score.mean", "Osa_Nor.score.mean")) |>
  ggplot() +
    aes(start, value, col = name) +
    facet_wrap(~seqnames, nrow = 1) +
    geom_smooth(span = 0.25, se = FALSE) +
    ggtitle("Osaka: Chromosomal position vs. alignment score")
```

```
plots$Bar$scores$combined <- chrw$Bar |>
  as.data.frame() |>
  subset(select=c("seqnames", "start", "end", "Bar_Oki.score.mean", "Bar_Kum.score.mean", "Bar_Osa.score.mean", "Bar_Aom.score.mean" )) |>
  plyranges::filter(seqnames %in% c('Chr1', 'Chr2', 'PAR', 'XSR')) |>
  tidyr::pivot_longer(c("Bar_Oki.score.mean", "Bar_Kum.score.mean", "Bar_Osa.score.mean", "Bar_Aom.score.mean")) |>
  ggplot() +
    aes(start, value, col = name) +
    facet_wrap(~seqnames, nrow = 1) +
    geom_smooth(span = 0.25, se = FALSE) +
    ggtitle("Barcelona: Chromosomal position vs. alignment score")
```

```
(plots$Oki$scores$combined / plots$Osa$scores$combined / plots$Bar$scores$combined)
```

See also the scaled scores in the Extra section.

### Width-related chromosome plots

```
# Okinawa
chrw$Oki$Oki_Osa.width.mean <- binApply(mean, na.rm = T, bins_Oki, width(gbs$Oki_Osa), matchToOneBin(gbs$Oki_Osa, bins_Oki))
chrw$Oki$Oki_Aom.width.mean <- binApply(mean, na.rm = T, bins_Oki, width(gbs$Oki_Aom), matchToOneBin(gbs$Oki_Aom, bins_Oki))
chrw$Oki$Oki_Bar.width.mean <- binApply(mean, na.rm = T, bins_Oki, width(gbs$Oki_Bar), matchToOneBin(gbs$Oki_Bar, bins_Oki))
chrw$Oki$Oki_Nor.width.mean <- binApply(mean, na.rm = T, bins_Oki, width(gbs$Oki_Nor), matchToOneBin(gbs$Oki_Nor, bins_Oki))

# Osaka
chrw$Osa$Osa_Oki.width.mean <- binApply(mean, na.rm = T, bins_Osa, width(gbs$Osa_Oki), matchToOneBin(gbs$Osa_Oki, bins_Osa))
chrw$Osa$Osa_Kum.width.mean <- binApply(mean, na.rm = T, bins_Osa, width(gbs$Osa_Kum), matchToOneBin(gbs$Osa_Kum, bins_Osa))
chrw$Osa$Osa_Bar.width.mean <- binApply(mean, na.rm = T, bins_Osa, width(gbs$Osa_Bar), matchToOneBin(gbs$Osa_Bar, bins_Osa))
chrw$Osa$Osa_Nor.width.mean <- binApply(mean, na.rm = T, bins_Osa, width(gbs$Osa_Nor), matchToOneBin(gbs$Osa_Nor, bins_Osa))

# Barcelona
chrw$Bar$Bar_Osa.width.mean <- binApply(mean, na.rm = T, bins_Bar, width(gbs$Bar_Osa), matchToOneBin(gbs$Bar_Osa, bins_Bar))
chrw$Bar$Bar_Aom.width.mean <- binApply(mean, na.rm = T, bins_Bar, width(gbs$Bar_Aom), matchToOneBin(gbs$Bar_Aom, bins_Bar))
chrw$Bar$Bar_Oki.width.mean <- binApply(mean, na.rm = T, bins_Bar, width(gbs$Bar_Oki), matchToOneBin(gbs$Bar_Oki, bins_Bar))
chrw$Bar$Bar_Kum.width.mean <- binApply(mean, na.rm = T, bins_Bar, width(gbs$Bar_Kum), matchToOneBin(gbs$Bar_Kum, bins_Bar))
```

#### Width-related chromosome plots, windows: Okinawa

```
plots$Oki$widths$widths <- chrw$Oki |> per_chrom_plot(Y=TRUE) + aes(y=Oki_Osa.width.mean) +
  scale_y_log10() +   ggtitle("Oki-Osa: Chromosomal position vs. aligned segment width")

plots$Oki$widths$combined <- chrw$Oki |>
  as.data.frame() |>
  subset(select=c("seqnames", "start", "end", "Oki_Osa.width.mean", "Oki_Aom.width.mean", "Oki_Bar.width.mean", "Oki_Nor.width.mean" )) |>
  plyranges::filter(seqnames %in% c('chr1', 'chr2', 'PAR', 'XSR')) |>
  tidyr::pivot_longer(c("Oki_Osa.width.mean", "Oki_Aom.width.mean", "Oki_Bar.width.mean", "Oki_Nor.width.mean")) |>
  ggplot() +
    aes(start, value, col = name) +
    facet_wrap(~seqnames, nrow = 1) +
    geom_smooth(method='loess', formula = y ~ x, span = 0.25, se = FALSE) +
    ggtitle("Okinawa: Chromosomal position vs. aligned segment width")
```

```
plots$Osa$widths$widths <- chrw$Osa |> per_chrom_plot(Y=TRUE) + aes(y=Osa_Bar.width.mean) +
  scale_y_log10() +  ggtitle("Osa-Bar: Chromosomal position vs. aligned segment width")

plots$Osa$widths$combined <- chrw$Osa |>
  as.data.frame() |>
  subset(select=c("seqnames", "start", "end", "Osa_Oki.width.mean", "Osa_Kum.width.mean", "Osa_Bar.width.mean", "Osa_Nor.width.mean" )) |>
  plyranges::filter(seqnames %in% c('Chr1', 'Chr2', 'PAR', 'XSR')) |>
  tidyr::pivot_longer(c("Osa_Oki.width.mean", "Osa_Kum.width.mean", "Osa_Bar.width.mean", "Osa_Nor.width.mean")) |>
  ggplot() +
  aes(start, value, col = name) +
  facet_wrap(~seqnames, nrow = 1) +
  geom_smooth(method='loess', formula = y ~ x, span = 0.25, se = FALSE) +
  ggtitle("Osaka: Chromosomal position vs. aligned segment width")
```

```
plots$Bar$widths$widths <- chrw$Bar |> per_chrom_plot(Y=TRUE) + aes(y=Bar_Aom.width.mean) +
  scale_y_log10() +  ggtitle("Bar-Aom: Chromosomal position vs. aligned segment width")

plots$Bar$widths$combined <- chrw$Bar |>
  as.data.frame() |>
  subset(select=c("seqnames", "start", "end", "Bar_Oki.width.mean", "Bar_Kum.width.mean", "Bar_Osa.width.mean", "Bar_Aom.width.mean" )) |>
  plyranges::filter(seqnames %in% c('Chr1', 'Chr2', 'PAR', 'XSR')) |>
  tidyr::pivot_longer(c("Bar_Oki.width.mean", "Bar_Kum.width.mean", "Bar_Osa.width.mean", "Bar_Aom.width.mean")) |>
  ggplot() +
  aes(start, value, col = name) +
  facet_wrap(~seqnames, nrow = 1) +
  geom_smooth(method='loess', formula = y ~ x, span = 0.25, se = FALSE) +
  ggtitle("Barcelona: Chromosomal position vs. aligned segment width")
```

#### Width-related chromosome plots, windows: Joined plots

```
(plots$Oki$widths$combined / plots$Osa$widths$combined / plots$Bar$widths$combined)
```

### Breakpoints

```
# get_bps returns a GRanges, but we need mcols to use grWindows.
# Add some mcols to the output of get_bps.
get_bps_mcol <- function(gr) {
  gr <- get_bps(gr)
  gr$breaks <- "break"
  gr
}
```

#### Breakpoint-related chromosome plots, windows: Okinawa

```
mcols(chrw$Oki) <- cbind(mcols(chrw$Oki), mcols(grWindows(genome=genomes$Oki, meta=get_bps_mcol(gbs$Oki_Osa), windowSize=windowSize, meta_prefix = 'bp.Oki_Osa.')))

mcols(chrw$Oki) <- cbind(mcols(chrw$Oki), mcols(grWindows(genome=genomes$Oki, meta=get_bps_mcol(gbs$Oki_Bar), windowSize=windowSize, meta_prefix = 'bp.Oki_Bar.')))

mcols(chrw$Oki) <- cbind(mcols(chrw$Oki), mcols(grWindows(genome=genomes$Oki, meta=get_bps_mcol(gbs$Oki_Kum), windowSize=windowSize, meta_prefix = 'bp.Oki_Kum.')))

mcols(chrw$Oki) <- cbind(mcols(chrw$Oki), mcols(grWindows(genome=genomes$Oki, meta=get_bps_mcol(gbs$Oki_Aom), windowSize=windowSize, meta_prefix = 'bp.Oki_Aom.')))

mcols(chrw$Oki) <- cbind(mcols(chrw$Oki), mcols(grWindows(genome=genomes$Oki, meta=get_bps_mcol(gbs$Oki_Nor), windowSize=windowSize, meta_prefix = 'bp.Oki_Nor.')))

# And a mean count
chrw$Oki$bp.mean.breaks <- chrw$Oki |> as.data.frame()|> select("bp.Oki_Osa.breaks.count", "bp.Oki_Bar.breaks.count", "bp.Oki_Kum.breaks.count", "bp.Oki_Aom.breaks.count",  "bp.Oki_Nor.breaks.count") |> as.data.frame() |> rowMeans()

p1_ysr <- chrw$Oki |>
  as.data.frame() |>
  subset(select=c("seqnames", "start", "end", "bp.Oki_Osa.breaks.count", "bp.Oki_Bar.breaks.count", "bp.Oki_Kum.breaks.count", "bp.Oki_Aom.breaks.count",  "bp.Oki_Nor.breaks.count" )) |>
  plyranges::filter(seqnames %in% c('chr1', 'chr2', 'PAR', 'XSR', 'YSR')) |>
  tidyr::pivot_longer(c("bp.Oki_Osa.breaks.count", "bp.Oki_Bar.breaks.count", "bp.Oki_Kum.breaks.count", "bp.Oki_Aom.breaks.count",  "bp.Oki_Nor.breaks.count" )) |>
  ggplot() +
    aes(start, value, col = name) +
    facet_wrap(~seqnames, nrow = 1) +
    geom_smooth(method='loess', formula = y ~ x, span = 0.25, se = FALSE) +
    ggtitle("Okinawa: Chromosomal position vs. number of breakpoints")

p1_noysr <- chrw$Oki |>
  as.data.frame() |>
  subset(select=c("seqnames", "start", "end", "bp.Oki_Osa.breaks.count", "bp.Oki_Bar.breaks.count", "bp.Oki_Kum.breaks.count", "bp.Oki_Aom.breaks.count",  "bp.Oki_Nor.breaks.count" )) |>
  plyranges::filter(seqnames %in% c('chr1', 'chr2', 'PAR', 'XSR')) |>
  tidyr::pivot_longer(c("bp.Oki_Osa.breaks.count", "bp.Oki_Bar.breaks.count", "bp.Oki_Kum.breaks.count", "bp.Oki_Aom.breaks.count",  "bp.Oki_Nor.breaks.count" )) |>
  ggplot() +
    aes(start, value, col = name) +
    facet_wrap(~seqnames, nrow = 1) +
    geom_smooth(method='loess', formula = y ~ x, span = 0.25, se = FALSE) +
    ggtitle("Okinawa: Chromosomal position vs. number of breakpoints")
```

#### Breakpoint-related chromosome plots, windows: Osaka

```
mcols(chrw$Osa) <- cbind(mcols(chrw$Osa), mcols(grWindows(genome=genomes$Osa, meta=get_bps_mcol(gbs$Osa_Oki), windowSize=windowSize, meta_prefix = 'bp.Osa_Oki.')))

mcols(chrw$Osa) <- cbind(mcols(chrw$Osa), mcols(grWindows(genome=genomes$Osa, meta=get_bps_mcol(gbs$Osa_Bar), windowSize=windowSize, meta_prefix = 'bp.Osa_Bar.')))

mcols(chrw$Osa) <- cbind(mcols(chrw$Osa), mcols(grWindows(genome=genomes$Osa, meta=get_bps_mcol(gbs$Osa_Kum), windowSize=windowSize, meta_prefix = 'bp.Osa_Kum.')))

mcols(chrw$Osa) <- cbind(mcols(chrw$Osa), mcols(grWindows(genome=genomes$Osa, meta=get_bps_mcol(gbs$Osa_Aom), windowSize=windowSize, meta_prefix = 'bp.Osa_Aom.')))

mcols(chrw$Osa) <- cbind(mcols(chrw$Osa), mcols(grWindows(genome=genomes$Osa, meta=get_bps_mcol(gbs$Osa_Nor), windowSize=windowSize, meta_prefix = 'bp.Osa_Nor.')))

chrw$Osa$bp.mean.breaks <- chrw$Osa |> as.data.frame() |> select("bp.Osa_Oki.breaks.count", "bp.Osa_Bar.breaks.count", "bp.Osa_Kum.breaks.count", "bp.Osa_Aom.breaks.count",  "bp.Osa_Nor.breaks.count") |> as.data.frame() |> rowMeans()

p2_ysr <- chrw$Osa |>
  as.data.frame() |>
  subset(select=c("seqnames", "start", "end", "bp.Osa_Oki.breaks.count", "bp.Osa_Bar.breaks.count", "bp.Osa_Kum.breaks.count", "bp.Osa_Aom.breaks.count",  "bp.Osa_Nor.breaks.count" )) |>
  plyranges::filter(seqnames %in% c('Chr1', 'Chr2', 'PAR', 'XSR', 'YSR')) |>
  tidyr::pivot_longer(c("bp.Osa_Oki.breaks.count", "bp.Osa_Bar.breaks.count", "bp.Osa_Kum.breaks.count", "bp.Osa_Aom.breaks.count",  "bp.Osa_Nor.breaks.count" )) |>
  ggplot() +
    aes(start, value, col = name) +
    facet_wrap(~seqnames, nrow = 1) +
    geom_smooth(method='loess', formula = y ~ x, span = 0.25, se = FALSE) +
    ggtitle("Osaka: Chromosomal position vs. number of breakpoints")

p2_noysr <- chrw$Osa |>
  as.data.frame() |>
  subset(select=c("seqnames", "start", "end", "bp.Osa_Oki.breaks.count", "bp.Osa_Bar.breaks.count", "bp.Osa_Kum.breaks.count", "bp.Osa_Aom.breaks.count",  "bp.Osa_Nor.breaks.count" )) |>
  plyranges::filter(seqnames %in% c('Chr1', 'Chr2', 'PAR', 'XSR')) |>
  tidyr::pivot_longer(c("bp.Osa_Oki.breaks.count", "bp.Osa_Bar.breaks.count", "bp.Osa_Kum.breaks.count", "bp.Osa_Aom.breaks.count",  "bp.Osa_Nor.breaks.count" )) |>
  ggplot() +
    aes(start, value, col = name) +
    facet_wrap(~seqnames, nrow = 1) +
    geom_smooth(method='loess', formula = y ~ x, span = 0.25, se = FALSE) +
    ggtitle("Osaka: Chromosomal position vs. number of breakpoints")
```

#### Breakpoint-related chromosome plots, windows: Barcelona

```
mcols(chrw$Bar) <- cbind(mcols(chrw$Bar), mcols(grWindows(genome=genomes$Bar, meta=get_bps_mcol(gbs$Bar_Oki), windowSize=windowSize, meta_prefix = 'bp.Bar_Oki.')))

mcols(chrw$Bar) <- cbind(mcols(chrw$Bar), mcols(grWindows(genome=genomes$Bar, meta=get_bps_mcol(gbs$Bar_Osa), windowSize=windowSize, meta_prefix = 'bp.Bar_Osa.')))

mcols(chrw$Bar) <- cbind(mcols(chrw$Bar), mcols(grWindows(genome=genomes$Bar, meta=get_bps_mcol(gbs$Bar_Kum), windowSize=windowSize, meta_prefix = 'bp.Bar_Kum.')))

mcols(chrw$Bar) <- cbind(mcols(chrw$Bar), mcols(grWindows(genome=genomes$Bar, meta=get_bps_mcol(gbs$Bar_Aom), windowSize=windowSize, meta_prefix = 'bp.Bar_Aom.')))

mcols(chrw$Bar) <- cbind(mcols(chrw$Bar), mcols(grWindows(genome=genomes$Bar, meta=get_bps_mcol(gbs$Bar_Nor), windowSize=windowSize, meta_prefix = 'bp.Bar_Nor.')))

p3_ysr <- chrw$Bar |>
  as.data.frame() |>
  subset(select=c("seqnames", "start", "end", "bp.Bar_Oki.breaks.count", "bp.Bar_Osa.breaks.count", "bp.Bar_Kum.breaks.count", "bp.Bar_Aom.breaks.count",  "bp.Bar_Nor.breaks.count" )) |>
  plyranges::filter(seqnames %in% c('Chr1', 'Chr2', 'PAR', 'XSR', 'YSR')) |>
  tidyr::pivot_longer(c("bp.Bar_Oki.breaks.count", "bp.Bar_Osa.breaks.count", "bp.Bar_Kum.breaks.count", "bp.Bar_Aom.breaks.count",  "bp.Bar_Nor.breaks.count" )) |>
  ggplot() +
  aes(start, value, col = name) +
  facet_wrap(~seqnames, nrow = 1) +
  geom_smooth(method='loess', formula = y ~ x, span = 0.25, se = FALSE) +
  ggtitle("Barcelona: Chromosomal position vs. number of breakpoints")

p3_noysr <- chrw$Bar |>
  as.data.frame() |>
  subset(select=c("seqnames", "start", "end", "bp.Bar_Oki.breaks.count", "bp.Bar_Osa.breaks.count", "bp.Bar_Kum.breaks.count", "bp.Bar_Aom.breaks.count",  "bp.Bar_Nor.breaks.count" )) |>
  plyranges::filter(seqnames %in% c('Chr1', 'Chr2', 'PAR', 'XSR')) |>
  tidyr::pivot_longer(c("bp.Bar_Oki.breaks.count", "bp.Bar_Osa.breaks.count", "bp.Bar_Kum.breaks.count", "bp.Bar_Aom.breaks.count",  "bp.Bar_Nor.breaks.count" )) |>
  ggplot() +
  aes(start, value, col = name) +
  facet_wrap(~seqnames, nrow = 1) +
  geom_smooth(method='loess', formula = y ~ x, span = 0.25, se = FALSE) +
  ggtitle("Barcelona: Chromosomal position vs. number of breakpoints")
```

#### Breakpoint-related chromosome plots, windows: Joined plots

```
(p1_ysr / p2_ysr / p3_ysr )
```

```
#(p1_noysr / p2_noysr / p3_noysr )
```

#### Joining windowed plots

Note that there are two Y axes for these plots. Repeat and gene
density are shown on the same Y axis (left), while dN/dS is shown on the
second Y axis (right).

```
tmp <- chrw$Oki |> as.data.frame() |> plyranges::filter(seqnames %in% c("chr1", "chr2", "PAR", "XSR"))

tmp$bp.mean.breaks <- tmp |> select("bp.Oki_Osa.breaks.count", "bp.Oki_Bar.breaks.count", "bp.Oki_Kum.breaks.count", "bp.Oki_Aom.breaks.count",  "bp.Oki_Nor.breaks.count") |> as.data.frame() |> rowMeans()

scaleFactor <- max(tmp$bp.mean.breaks,na.rm=T)/max(tmp$transcripts.dNdS_PRANK.median, na.rm=T)
p1 <- ggplot(tmp, aes(x=start)) +
  geom_smooth(method='loess', formula = y ~ x, aes(y=bp.mean.breaks, color="genes")) +
  geom_smooth(method='loess', formula = y ~ x, aes(y=transcripts.dNdS_PRANK.median*scaleFactor, color="dN/dS")) +
  geom_smooth(method='loess', formula = y ~ x, aes(y=repeats.Class.count.total, color="repeats")) +
  scale_y_continuous(name="breakpoints/repeats per window", sec.axis=sec_axis(~./scaleFactor, name="median dN/dS per window")) +
  ggtitle("Okinawa: Breakpoints, genes, and dN/dS per window") + 
  facet_wrap(~seqnames, nrow=1)

# And a correlation plot
p1_c <- ggplot(tmp) + aes(x=bp.mean.breaks, y=transcripts.dNdS_GUIDANCE2.median) + geom_point() + geom_smooth(method='lm', col='red') + ggpubr::stat_cor() + facet_wrap(seqnames~Arm, ncol=2, scales = 'free') + ggtitle('Okinawa: Breakpoints vs. dN/dS') + ylab('dN/dS')
```

```
tmp <- chrw$Osa |> as.data.frame() |> plyranges::filter(seqnames %in% c("Chr1", "Chr2", "PAR", "XSR"))

tmp$bp.mean.breaks <- tmp |> select("bp.Osa_Oki.breaks.count", "bp.Osa_Bar.breaks.count", "bp.Osa_Kum.breaks.count", "bp.Osa_Aom.breaks.count",  "bp.Osa_Nor.breaks.count") |> as.data.frame() |> rowMeans()

scaleFactor <- max(tmp$bp.mean.breaks,na.rm=T)/max(tmp$transcripts.dNdS_PRANK.median, na.rm=T)

p2 <- ggplot(tmp, aes(x=start)) +
  geom_smooth(method='loess', formula = y ~ x, aes(y=bp.mean.breaks, color="genes")) +
  geom_smooth(method='loess', formula = y ~ x, aes(y=transcripts.dNdS_PRANK.median*scaleFactor, color="dN/dS")) +
  geom_smooth(method='loess', formula = y ~ x, aes(y=repeats.Class.count.total, color="repeats")) +
  scale_y_continuous(name="breakpoints/repeats per window", sec.axis=sec_axis(~./scaleFactor, name="median dN/dS per window")) +
  ggtitle("Osaka: Breakpoints, genes, and dN/dS per window") + 
  facet_wrap(~seqnames, nrow=1)

p2_c <- ggplot(tmp) + aes(x=bp.mean.breaks, y=transcripts.dNdS_GUIDANCE2.median) + geom_point() + geom_smooth(method='lm', col='red') + ggpubr::stat_cor() + facet_wrap(seqnames~Arm, ncol=2, scales = 'free') + ggtitle('Osaka: Breakpoints vs. dN/dS') + ylab('dN/dS')
```

```
tmp <- chrw$Bar |> as.data.frame() |> plyranges::filter(seqnames %in% c("Chr1", "Chr2", "PAR", "XSR"))

tmp$bp.mean.breaks <- tmp |> select("bp.Bar_Oki.breaks.count", "bp.Bar_Osa.breaks.count", "bp.Bar_Kum.breaks.count", "bp.Bar_Aom.breaks.count",  "bp.Bar_Nor.breaks.count") |> as.data.frame() |> rowMeans()

scaleFactor <- max(tmp$bp.mean.breaks,na.rm=T)/max(tmp$transcripts.dNdS_PRANK.median, na.rm=T)

p3 <- ggplot(tmp, aes(x=start)) +
  geom_smooth(method='loess', formula = y ~ x,aes(y=bp.mean.breaks, color="genes")) +
  geom_smooth(method='loess', formula = y ~ x,aes(y=transcripts.dNdS_PRANK.median*scaleFactor, color="dN/dS")) +
  geom_smooth(method='loess', formula = y ~ x,aes(y=repeats.Class.count.total, color="repeats")) +
  scale_y_continuous(name="breakpoints/repeats per window", sec.axis=sec_axis(~./scaleFactor, name="median dN/dS per window")) +
  ggtitle("Barcelona: Breakpoints, genes, and dN/dS per window") + 
  facet_wrap(~seqnames, nrow=1)

p3_c <- ggplot(tmp) + aes(x=bp.mean.breaks, y=transcripts.dNdS_GUIDANCE2.median) + geom_point() + geom_smooth(method='lm', col='red') + ggpubr::stat_cor() + facet_wrap(seqnames~Arm, ncol=2, scales = 'free') + ggtitle('Barcelona: Breakpoints vs. dN/dS') + ylab('dN/dS')
```

```
(  p1 /  p2  / p3 )
```

```
(p1_c + p2_c + p3_c )
```

## Statistics

### Transcript widths.

```
library(ggpubr)

append_width <- function(gr) {
  gr$w <- width(gr)
  gr
}

# All transcript widths - no binning, no windows, etc.
# Okinawa
tb <- transcripts$Oki |> plyranges::filter(seqnames %in% c('chr1', 'chr2', 'PAR', 'XSR', 'YSR')) |> flagLongShort(longShort$OKI2018.I69) |> append_width() |> as.data.frame()
tb$seqnames_arm <- paste0(tb$seqnames, "_", tb$Arm)
# Transcript length
p1 <- ggboxplot(tb, x='seqnames_arm', y='w', color='seqnames', add='jitter') + theme(axis.text.x=element_text(angle=90)) + ylab('transcript length') + stat_compare_means(comparison=list(c('chr1_short', 'chr1_long'), c('chr2_short', 'chr2_long'), c('PAR_short', 'PAR_long'), c('XSR_XSR', 'YSR_YSR'), c('XSR_XSR', 'PAR_short'), c('XSR_XSR', 'PAR_long'))) + ggtitle('Okinawa: Transcript lengths by chr+arm')
# Operon length
tb <- operons$Oki |> plyranges::filter(seqnames %in% c('chr1', 'chr2', 'PAR', 'XSR', 'YSR')) |> flagLongShort(longShort$OKI2018.I69) |> append_width()
tb$seqnames_arm <- paste0(seqnames(tb), "_", tb$Arm)
names(tb) <- NULL
tb <- tb |> as.data.frame()
p2 <- ggboxplot(tb, x='seqnames_arm', y='n', color='seqnames', add='jitter') + theme(axis.text.x=element_text(angle=90)) + ylab('operon length') + stat_compare_means(comparison=list(c('chr1_short', 'chr1_long'), c('chr2_short', 'chr2_long'), c('PAR_short', 'PAR_long'), c('XSR_XSR', 'YSR_YSR'), c('XSR_XSR', 'PAR_short'), c('XSR_XSR', 'PAR_long'))) + ggtitle('Okinawa: Operon lengths by chr+arm')

# Osaka
tb <- transcripts$Osa |> plyranges::filter(seqnames %in% c('Chr1', 'Chr2', 'PAR', 'XSR', 'YSR')) |> flagLongShort(longShort$OSKA2016v1.9) |> append_width() |> as.data.frame()
tb$seqnames_arm <- paste0(tb$seqnames, "_", tb$Arm)
p3 <- ggboxplot(tb, x='seqnames_arm', y='w', color='seqnames', add='jitter') + theme(axis.text.x=element_text(angle=90)) + ylab('transcript length') + stat_compare_means(comparison=list(c('Chr1_short', 'Chr1_long'), c('Chr2_short', 'Chr2_long'), c('PAR_short', 'PAR_long'), c('XSR_XSR', 'YSR_YSR'), c('XSR_XSR', 'PAR_short'), c('XSR_XSR', 'PAR_long'))) + ggtitle('Osaka: Transcript lengths by chr+arm')
# Operon length
tb <- operons$Osa |> plyranges::filter(seqnames %in% c('Chr1', 'Chr2', 'PAR', 'XSR', 'YSR')) |> flagLongShort(longShort$OSKA2016v1.9) |> append_width()
tb$seqnames_arm <- paste0(seqnames(tb), "_", tb$Arm)
names(tb) <- NULL
tb <- tb |> as.data.frame()
p4 <- ggboxplot(tb, x='seqnames_arm', y='n', color='seqnames', add='jitter') + theme(axis.text.x=element_text(angle=90)) + ylab('operon length') + stat_compare_means(comparison=list(c('Chr1_short', 'Chr1_long'), c('Chr2_short', 'Chr2_long'), c('PAR_short', 'PAR_long'), c('XSR_XSR', 'YSR_YSR'), c('XSR_XSR', 'PAR_short'), c('XSR_XSR', 'PAR_long'))) + ggtitle('Osaka: Operon lengths by chr+arm')


# Barcelona
tb <- transcripts$Bar |> plyranges::filter(seqnames %in% c('Chr1', 'Chr2', 'PAR', 'XSR', 'YSR')) |> flagLongShort(longShort$Bar2.p4) |> append_width() |> as.data.frame()
tb$seqnames_arm <- paste0(tb$seqnames, "_", tb$Arm)
p5 <- ggboxplot(tb, x='seqnames_arm', y='w', color='seqnames', add='jitter') + theme(axis.text.x=element_text(angle=90)) + ylab('transcript length') + stat_compare_means(comparison=list(c('Chr1_short', 'Chr1_long'), c('Chr2_short', 'Chr2_long'), c('PAR_short', 'PAR_long'), c('XSR_XSR', 'YSR_YSR'), c('XSR_XSR', 'PAR_short'), c('XSR_XSR', 'PAR_long'))) + ggtitle('Barcelona: Transcript lengths by chr+arm')
# Operon length
tb <- operons$Bar |> plyranges::filter(seqnames %in% c('Chr1', 'Chr2', 'PAR', 'XSR', 'YSR')) |> flagLongShort(longShort$Bar2.p4) |> append_width()
tb$seqnames_arm <- paste0(seqnames(tb), "_", tb$Arm)
names(tb) <- NULL
tb <- tb |> as.data.frame()
p6 <- ggboxplot(tb, x='seqnames_arm', y='n', color='seqnames', add='jitter') + theme(axis.text.x=element_text(angle=90)) + ylab('operon length') + stat_compare_means(comparison=list(c('Chr1_short', 'Chr1_long'), c('Chr2_short', 'Chr2_long'), c('PAR_short', 'PAR_long'), c('XSR_XSR', 'YSR_YSR'), c('XSR_XSR', 'PAR_short'), c('XSR_XSR', 'PAR_long'))) + ggtitle('Barcelona: Operon lengths by chr+arm')

p1 + p3 + p5
```

```
p2 + p4 + p6
```

#### Statistics: Windows, Okinawa

```
library(ggpubr)
tb <- chrw$Oki |> as.data.frame() |> filter(seqnames %in% c('chr1', 'chr2', 'PAR', 'XSR', 'YSR'))
tb$seqnames_arm <- paste0(tb$seqnames, "_", tb$Arm)
plots$Oki$boxplots <- list()
plots$Oki$boxplots$operon_density <- ggboxplot(tb, x='seqnames_arm', y='operons.n.count', color='seqnames', add='jitter') +
  theme(axis.text.x=element_text(angle=90)) +
  ylab('operon density per window') +
  stat_compare_means(comparison=list(c('chr1_short', 'chr1_long'), c('chr2_short', 'chr2_long'), c('PAR_short', 'PAR_long'), c('XSR_XSR', 'YSR_YSR'), c('XSR_XSR', 'PAR_short'), c('XSR_XSR', 'PAR_long'))) +
  ggtitle('Okinawa: operon density by chr+arm')

plots$Oki$boxplots$operon_size <- ggboxplot(tb, x='seqnames_arm', y='operons.n.mean', color='seqnames', add='jitter') +
  theme(axis.text.x=element_text(angle=90)) +
  ylab('operon size per window') +
  stat_compare_means(comparison=list(c('chr1_short', 'chr1_long'), c('chr2_short', 'chr2_long'), c('PAR_short', 'PAR_long'), c('XSR_XSR', 'YSR_YSR'), c('XSR_XSR', 'PAR_short'), c('XSR_XSR', 'PAR_long'))) +
  ggtitle('Okinawa: operon size by chr+arm')

plots$Oki$boxplots$transcript_density <- ggboxplot(tb, x='seqnames_arm', y='transcripts.tx_id.count', color='seqnames', add='jitter') +
  theme(axis.text.x=element_text(angle=90)) +
  ylab('transcript size per window') +
  stat_compare_means(comparison=list(c('chr1_short', 'chr1_long'), c('chr2_short', 'chr2_long'), c('PAR_short', 'PAR_long'), c('XSR_XSR', 'YSR_YSR'), c('XSR_XSR', 'PAR_short'), c('XSR_XSR', 'PAR_long'))) +
  ggtitle('Okinawa: transcript density by chr+arm')

plots$Oki$boxplots$transcript_size <- ggboxplot(tb, x='seqnames_arm', y='transcripts.tx_len.median', color='seqnames', add='jitter') +
  theme(axis.text.x=element_text(angle=90)) +
  ylab('transcript size per window') +
  stat_compare_means(comparison=list(c('chr1_short', 'chr1_long'), c('chr2_short', 'chr2_long'), c('PAR_short', 'PAR_long'), c('XSR_XSR', 'YSR_YSR'), c('XSR_XSR', 'PAR_short'), c('XSR_XSR', 'PAR_long'))) +
  ggtitle('Okinawa: transcript size by chr+arm')

plots$Oki$boxplots$dnds <- ggboxplot(tb, x='seqnames_arm', y='transcripts.dNdS_GUIDANCE2.median', color='seqnames', add='jitter') +
  theme(axis.text.x=element_text(angle=90)) +
  ylab('median dN/dS per window') +
  stat_compare_means(comparison=list(c('chr1_short', 'chr1_long'), c('chr2_short', 'chr2_long'), c('PAR_short', 'PAR_long'), c('XSR_XSR', 'PAR_short'), c('XSR_XSR', 'PAR_long'))) +
  ggtitle('Okinawa: median dN/dS by chr+arm')
```

#### Statistics: Windows, Osaka

```
tb <- chrw$Osa |> as.data.frame() |> filter(seqnames %in% c('Chr1', 'Chr2', 'PAR', 'XSR', 'YSR'))
tb$seqnames_arm <- paste0(tb$seqnames, "_", tb$Arm)
plots$Osa$boxplots <- list()
plots$Osa$boxplots$operon_density <- ggboxplot(tb, x='seqnames_arm', y='operons.n.count', color='seqnames', add='jitter') +
  theme(axis.text.x=element_text(angle=90)) +
  ylab('operon density per window') +
  stat_compare_means(comparison=list(c('Chr1_short', 'Chr1_long'), c('Chr2_short', 'Chr2_long'), c('PAR_short', 'PAR_long'), c('XSR_XSR', 'YSR_YSR'), c('XSR_XSR', 'PAR_short'), c('XSR_XSR', 'PAR_long'))) +
  ggtitle('Osaka: operon density by chr+arm')

plots$Osa$boxplots$operon_size <- ggboxplot(tb, x='seqnames_arm', y='operons.n.mean', color='seqnames', add='jitter') +
  theme(axis.text.x=element_text(angle=90)) +
  ylab('operon size per window') +
  stat_compare_means(comparison=list(c('Chr1_short', 'Chr1_long'), c('Chr2_short', 'Chr2_long'), c('PAR_short', 'PAR_long'), c('XSR_XSR', 'YSR_YSR'), c('XSR_XSR', 'PAR_short'), c('XSR_XSR', 'PAR_long'))) +
  ggtitle('Osaka: operon size by chr+arm')

plots$Osa$boxplots$transcript_density <- ggboxplot(tb, x='seqnames_arm', y='transcripts.tx_id.count', color='seqnames', add='jitter') +
  theme(axis.text.x=element_text(angle=90)) +
  ylab('transcript size per window') +
  stat_compare_means(comparison=list(c('Chr1_short', 'Chr1_long'), c('Chr2_short', 'Chr2_long'), c('PAR_short', 'PAR_long'), c('XSR_XSR', 'YSR_YSR'), c('XSR_XSR', 'PAR_short'), c('XSR_XSR', 'PAR_long'))) +
  ggtitle('Osaka: transcript density by chr+arm')

plots$Osa$boxplots$transcript_size <- ggboxplot(tb, x='seqnames_arm', y='transcripts.tx_len.median', color='seqnames', add='jitter') +
  theme(axis.text.x=element_text(angle=90)) +
  ylab('transcript size per window') +
  stat_compare_means(comparison=list(c('Chr1_short', 'Chr1_long'), c('Chr2_short', 'Chr2_long'), c('PAR_short', 'PAR_long'), c('XSR_XSR', 'YSR_YSR'), c('XSR_XSR', 'PAR_short'), c('XSR_XSR', 'PAR_long'))) +
  ggtitle('Osaka: transcript size by chr+arm')

plots$Osa$boxplots$dnds <- ggboxplot(tb, x='seqnames_arm', y='transcripts.dNdS_GUIDANCE2.median', color='seqnames', add='jitter') +
  theme(axis.text.x=element_text(angle=90)) +
  ylab('median dN/dS per window') +
  stat_compare_means(comparison=list(c('Chr1_short', 'Chr1_long'), c('Chr2_short', 'Chr2_long'), c('PAR_short', 'PAR_long'), c('XSR_XSR', 'PAR_short'), c('XSR_XSR', 'PAR_long'))) +
  ggtitle('Osaka: median dN/dS by chr+arm')
```

#### Statistics: Windows, Barcelona

```
tb <- chrw$Bar |> as.data.frame() |> filter(seqnames %in% c('Chr1', 'Chr2', 'PAR', 'XSR', 'YSR'))
tb$seqnames_arm <- paste0(tb$seqnames, "_", tb$Arm)
plots$Bar$boxplots <- list()
plots$Bar$boxplots$operon_density <- ggboxplot(tb, x='seqnames_arm', y='operons.n.count', color='seqnames', add='jitter') +
  theme(axis.text.x=element_text(angle=90)) +
  ylab('operon density per window') +
  stat_compare_means(comparison=list(c('Chr1_short', 'Chr1_long'), c('Chr2_short', 'Chr2_long'), c('PAR_short', 'PAR_long'), c('XSR_XSR', 'YSR_YSR'), c('XSR_XSR', 'PAR_short'), c('XSR_XSR', 'PAR_long'))) +
  ggtitle('Barcelona: operon density by chr+arm')

plots$Bar$boxplots$operon_size <- ggboxplot(tb, x='seqnames_arm', y='operons.n.mean', color='seqnames', add='jitter') +
  theme(axis.text.x=element_text(angle=90)) +
  ylab('operon size per window') +
  stat_compare_means(comparison=list(c('Chr1_short', 'Chr1_long'), c('Chr2_short', 'Chr2_long'), c('PAR_short', 'PAR_long'), c('XSR_XSR', 'YSR_YSR'), c('XSR_XSR', 'PAR_short'), c('XSR_XSR', 'PAR_long'))) +
  ggtitle('Barcelona: operon size by chr+arm')

plots$Bar$boxplots$transcript_density <- ggboxplot(tb, x='seqnames_arm', y='transcripts.tx_id.count', color='seqnames', add='jitter') +
  theme(axis.text.x=element_text(angle=90)) +
  ylab('transcript size per window') +
  stat_compare_means(comparison=list(c('Chr1_short', 'Chr1_long'), c('Chr2_short', 'Chr2_long'), c('PAR_short', 'PAR_long'), c('XSR_XSR', 'YSR_YSR'), c('XSR_XSR', 'PAR_short'), c('XSR_XSR', 'PAR_long'))) +
  ggtitle('Barcelona: transcript density by chr+arm')

plots$Bar$boxplots$transcript_size <- ggboxplot(tb, x='seqnames_arm', y='transcripts.tx_len.median', color='seqnames', add='jitter') +
  theme(axis.text.x=element_text(angle=90)) +
  ylab('transcript size per window') +
  stat_compare_means(comparison=list(c('Chr1_short', 'Chr1_long'), c('Chr2_short', 'Chr2_long'), c('PAR_short', 'PAR_long'), c('XSR_XSR', 'YSR_YSR'), c('XSR_XSR', 'PAR_short'), c('XSR_XSR', 'PAR_long'))) +
  ggtitle('Barcelona: transcript size by chr+arm')

plots$Bar$boxplots$dnds <- ggboxplot(tb, x='seqnames_arm', y='transcripts.dNdS_GUIDANCE2.median', color='seqnames', add='jitter') +
  theme(axis.text.x=element_text(angle=90)) +
  ylab('median dN/dS per window') +
  stat_compare_means(comparison=list(c('Chr1_short', 'Chr1_long'), c('Chr2_short', 'Chr2_long'), c('PAR_short', 'PAR_long'), c('XSR_XSR', 'YSR_YSR'), c('XSR_XSR', 'PAR_short'), c('XSR_XSR', 'PAR_long'))) +
  ggtitle('Barcelona: median dN/dS by chr+arm')
detach('package:ggpubr')
```

```
(plots$Oki$boxplots$transcript_density + plots$Osa$boxplots$transcript_density + plots$Bar$boxplots$transcript_density) /
(plots$Oki$boxplots$transcript_size    + plots$Osa$boxplots$transcript_size    + plots$Bar$boxplots$transcript_size)
```

```
(plots$Oki$boxplots$operon_density + plots$Osa$boxplots$operon_density + plots$Bar$boxplots$operon_density) /
(plots$Oki$boxplots$operon_size    + plots$Osa$boxplots$operon_size    + plots$Bar$boxplots$operon_size)
```

```
plots$Oki$boxplots$dnds + plots$Osa$boxplots$dnds + plots$Bar$boxplots$dnds
```

### Transforming to a single coordinate system

In this section, I create some utility functions to scale genomic
ranges to the same coordinate system, then calculate averages of some
statistics over the windows in these regions.

First, create some helper functions for tile-based indexing.

```
genomeGRToTiles <- function(gr, n=100) {
  tile(gr, n=n) |> unlist() |> unname()
}

grTiles <- function(genome, meta, n=100, meta_prefix=NULL){
  genome <- BSgenomeToGR(genome)                                 # Make GRanges from genome
  genome <- genomeGRToTiles(genome, n=n)                         # Make a tiled GRange from chromosome GRanges
  mpw <-    metaPerWindow(genome, meta, meta_prefix=meta_prefix) # Make metadata column windows and intersect
  mpw
}
```

```
nTiles <- 100
tiles_Oki <- genomes$Oki |> BSgenomeToGR() |> genomeGRToTiles(nTiles)
tiles_Osa <- genomes$Osa |> BSgenomeToGR() |> genomeGRToTiles(nTiles)
tiles_Bar <- genomes$Bar |> BSgenomeToGR() |> genomeGRToTiles(nTiles)

chrt <- SimpleList()
chrt$Oki <- grTiles(genome=genomes$Oki, meta=transcripts$Oki, n=nTiles, meta_prefix = 'transcripts.')
chrt$Oki <- flagLongShort(chrt$Oki, longShort$OKI2018.I69)
chrt$Osa <- grTiles(genome=genomes$Osa, meta=transcripts$Osa, n=nTiles, meta_prefix = 'transcripts.')
chrt$Osa <- flagLongShort(chrt$Osa, longShort$OSKA2016v1.9)
chrt$Bar <- grTiles(genome=genomes$Bar, meta=transcripts$Bar, n=nTiles, meta_prefix = 'transcripts.')
chrt$Bar <- flagLongShort(chrt$Bar, longShort$Bar2.p4)

# Operons
mcols(chrt$Oki) <- cbind(mcols(chrt$Oki), mcols(grTiles(genome=genomes$Oki, meta=operons$Oki, n=nTiles, meta_prefix = 'operons.'))) |> suppressWarnings()
mcols(chrt$Osa) <- cbind(mcols(chrt$Osa), mcols(grTiles(genome=genomes$Osa, meta=operons$Osa, n=nTiles, meta_prefix = 'operons.'))) |> suppressWarnings()
mcols(chrt$Bar) <- cbind(mcols(chrt$Bar), mcols(grTiles(genome=genomes$Bar, meta=operons$Bar, n=nTiles, meta_prefix = 'operons.'))) |> suppressWarnings()

# Repeats
mcols(chrt$Oki) <- cbind(mcols(chrt$Oki), mcols(grTiles(genome=genomes$Oki, meta=reps$Oki, n=nTiles, meta_prefix = 'repeats.')))
mcols(chrt$Osa) <- cbind(mcols(chrt$Osa), mcols(grTiles(genome=genomes$Osa, meta=reps$Osa, n=nTiles, meta_prefix = 'repeats.')))
mcols(chrt$Bar) <- cbind(mcols(chrt$Bar), mcols(grTiles(genome=genomes$Bar, meta=reps$Bar, n=nTiles, meta_prefix = 'repeats.')))

# Widths
chrt$Oki$Oki_Osa.width.mean <- binApply(mean, na.rm = T, tiles_Oki, width(gbs$Oki_Osa), matchToOneBin(gbs$Oki_Osa, tiles_Oki))
chrt$Oki$Oki_Aom.width.mean <- binApply(mean, na.rm = T, tiles_Oki, width(gbs$Oki_Aom), matchToOneBin(gbs$Oki_Aom, tiles_Oki))
chrt$Oki$Oki_Bar.width.mean <- binApply(mean, na.rm = T, tiles_Oki, width(gbs$Oki_Bar), matchToOneBin(gbs$Oki_Bar, tiles_Oki))
chrt$Oki$Oki_Nor.width.mean <- binApply(mean, na.rm = T, tiles_Oki, width(gbs$Oki_Nor), matchToOneBin(gbs$Oki_Nor, tiles_Oki))
chrt$Osa$Osa_Oki.width.mean <- binApply(mean, na.rm = T, tiles_Osa, width(gbs$Osa_Oki), matchToOneBin(gbs$Osa_Oki, tiles_Osa))
chrt$Osa$Osa_Kum.width.mean <- binApply(mean, na.rm = T, tiles_Osa, width(gbs$Osa_Kum), matchToOneBin(gbs$Osa_Kum, tiles_Osa))
chrt$Osa$Osa_Bar.width.mean <- binApply(mean, na.rm = T, tiles_Osa, width(gbs$Osa_Bar), matchToOneBin(gbs$Osa_Bar, tiles_Osa))
chrt$Osa$Osa_Nor.width.mean <- binApply(mean, na.rm = T, tiles_Osa, width(gbs$Osa_Nor), matchToOneBin(gbs$Osa_Nor, tiles_Osa))
chrt$Bar$Bar_Osa.width.mean <- binApply(mean, na.rm = T, bins_Bar, width(gbs$Bar_Osa), matchToOneBin(gbs$Bar_Osa, bins_Bar))
chrt$Bar$Bar_Aom.width.mean <- binApply(mean, na.rm = T, bins_Bar, width(gbs$Bar_Aom), matchToOneBin(gbs$Bar_Aom, bins_Bar))
chrt$Bar$Bar_Oki.width.mean <- binApply(mean, na.rm = T, bins_Bar, width(gbs$Bar_Oki), matchToOneBin(gbs$Bar_Oki, bins_Bar))
chrt$Bar$Bar_Kum.width.mean <- binApply(mean, na.rm = T, bins_Bar, width(gbs$Bar_Kum), matchToOneBin(gbs$Bar_Kum, bins_Bar))

# Breakpoints
mcols(chrt$Oki) <- cbind(mcols(chrt$Oki), mcols(grTiles(genome=genomes$Oki, meta=get_bps_mcol(gbs$Oki_Osa), n=nTiles, meta_prefix = 'bp.Oki_Osa.')))
mcols(chrt$Oki) <- cbind(mcols(chrt$Oki), mcols(grTiles(genome=genomes$Oki, meta=get_bps_mcol(gbs$Oki_Bar), n=nTiles, meta_prefix = 'bp.Oki_Bar.')))
mcols(chrt$Oki) <- cbind(mcols(chrt$Oki), mcols(grTiles(genome=genomes$Oki, meta=get_bps_mcol(gbs$Oki_Kum), n=nTiles, meta_prefix = 'bp.Oki_Kum.')))
mcols(chrt$Oki) <- cbind(mcols(chrt$Oki), mcols(grTiles(genome=genomes$Oki, meta=get_bps_mcol(gbs$Oki_Aom), n=nTiles, meta_prefix = 'bp.Oki_Aom.')))
mcols(chrt$Oki) <- cbind(mcols(chrt$Oki), mcols(grTiles(genome=genomes$Oki, meta=get_bps_mcol(gbs$Oki_Nor), n=nTiles, meta_prefix = 'bp.Oki_Nor.')))
chrt$Oki$bp.mean.breaks <- chrt$Oki |> as.data.frame()|> select("bp.Oki_Osa.breaks.count", "bp.Oki_Bar.breaks.count", "bp.Oki_Kum.breaks.count", "bp.Oki_Aom.breaks.count",  "bp.Oki_Nor.breaks.count") |> as.data.frame() |> rowMeans()

mcols(chrt$Osa) <- cbind(mcols(chrt$Osa), mcols(grTiles(genome=genomes$Osa, meta=get_bps_mcol(gbs$Osa_Oki), n=nTiles, meta_prefix = 'bp.Osa_Oki.')))
mcols(chrt$Osa) <- cbind(mcols(chrt$Osa), mcols(grTiles(genome=genomes$Osa, meta=get_bps_mcol(gbs$Osa_Bar), n=nTiles, meta_prefix = 'bp.Osa_Bar.')))
mcols(chrt$Osa) <- cbind(mcols(chrt$Osa), mcols(grTiles(genome=genomes$Osa, meta=get_bps_mcol(gbs$Osa_Kum), n=nTiles, meta_prefix = 'bp.Osa_Kum.')))
mcols(chrt$Osa) <- cbind(mcols(chrt$Osa), mcols(grTiles(genome=genomes$Osa, meta=get_bps_mcol(gbs$Osa_Aom), n=nTiles, meta_prefix = 'bp.Osa_Aom.')))
mcols(chrt$Osa) <- cbind(mcols(chrt$Osa), mcols(grTiles(genome=genomes$Osa, meta=get_bps_mcol(gbs$Osa_Nor), n=nTiles, meta_prefix = 'bp.Osa_Nor.')))
chrt$Osa$bp.mean.breaks <- chrt$Osa |> as.data.frame()|> select("bp.Osa_Oki.breaks.count", "bp.Osa_Bar.breaks.count", "bp.Osa_Kum.breaks.count", "bp.Osa_Aom.breaks.count", "bp.Osa_Nor.breaks.count") |> as.data.frame() |> rowMeans()

mcols(chrt$Bar) <- cbind(mcols(chrt$Bar), mcols(grTiles(genome=genomes$Bar, meta=get_bps_mcol(gbs$Bar_Oki), n=nTiles, meta_prefix = 'bp.Bar_Oki.')))
mcols(chrt$Bar) <- cbind(mcols(chrt$Bar), mcols(grTiles(genome=genomes$Bar, meta=get_bps_mcol(gbs$Bar_Osa), n=nTiles, meta_prefix = 'bp.Bar_Osa.')))
mcols(chrt$Bar) <- cbind(mcols(chrt$Bar), mcols(grTiles(genome=genomes$Bar, meta=get_bps_mcol(gbs$Bar_Kum), n=nTiles, meta_prefix = 'bp.Bar_Kum.')))
mcols(chrt$Bar) <- cbind(mcols(chrt$Bar), mcols(grTiles(genome=genomes$Bar, meta=get_bps_mcol(gbs$Bar_Aom), n=nTiles, meta_prefix = 'bp.Bar_Aom.')))
mcols(chrt$Bar) <- cbind(mcols(chrt$Bar), mcols(grTiles(genome=genomes$Bar, meta=get_bps_mcol(gbs$Bar_Nor), n=nTiles, meta_prefix = 'bp.Bar_Nor.')))
chrt$Bar$bp.mean.breaks <- chrt$Bar |> as.data.frame()|> select("bp.Bar_Oki.breaks.count", "bp.Bar_Osa.breaks.count", "bp.Bar_Kum.breaks.count", "bp.Bar_Aom.breaks.count", "bp.Bar_Nor.breaks.count") |> as.data.frame() |> rowMeans()

# Alignment categories
# Okinawa
mcols(chrt$Oki) <- cbind(mcols(chrt$Oki), mcols(grTiles(genome=genomes$Oki, meta=wgo$Oki_Osa, n=nTiles, meta_prefix = 'ac.Oki_Osa.')))
mcols(chrt$Oki) <- cbind(mcols(chrt$Oki), mcols(grTiles(genome=genomes$Oki, meta=wgo$Oki_Bar, n=nTiles, meta_prefix = 'ac.Oki_Bar.')))
mcols(chrt$Oki) <- cbind(mcols(chrt$Oki), mcols(grTiles(genome=genomes$Oki, meta=wgo$Oki_Kum, n=nTiles, meta_prefix = 'ac.Oki_Kum.')))
mcols(chrt$Oki) <- cbind(mcols(chrt$Oki), mcols(grTiles(genome=genomes$Oki, meta=wgo$Oki_Aom, n=nTiles, meta_prefix = 'ac.Oki_Aom.')))
mcols(chrt$Oki) <- cbind(mcols(chrt$Oki), mcols(grTiles(genome=genomes$Oki, meta=wgo$Oki_Nor, n=nTiles, meta_prefix = 'ac.Oki_Nor.')))
chrt$Oki$ac.isolated.mean <- chrt$Oki |> as.data.frame()|> select("ac.Oki_Osa.type.isolated.alignment.count.total", "ac.Oki_Bar.type.isolated.alignment.count.total", "ac.Oki_Kum.type.isolated.alignment.count.total", "ac.Oki_Aom.type.isolated.alignment.count.total", "ac.Oki_Nor.type.isolated.alignment.count.total") |> as.data.frame() |> rowMeans()
chrt$Oki$ac.collinear.mean <- chrt$Oki |> as.data.frame()|> select("ac.Oki_Osa.type.collinear.alignment.count.total", "ac.Oki_Bar.type.collinear.alignment.count.total", "ac.Oki_Kum.type.collinear.alignment.count.total", "ac.Oki_Aom.type.collinear.alignment.count.total", "ac.Oki_Nor.type.collinear.alignment.count.total") |> as.data.frame() |> rowMeans()
chrt$Oki$ac.breakpoint.mean <- chrt$Oki |> as.data.frame()|> select("ac.Oki_Osa.type.breakpoint.region.count.total", "ac.Oki_Bar.type.breakpoint.region.count.total", "ac.Oki_Kum.type.breakpoint.region.count.total", "ac.Oki_Aom.type.breakpoint.region.count.total", "ac.Oki_Nor.type.breakpoint.region.count.total") |> as.data.frame() |> rowMeans()
chrt$Oki$ac.bridge.mean <- chrt$Oki |> as.data.frame()|> select("ac.Oki_Osa.type.bridge.region.count.total", "ac.Oki_Bar.type.bridge.region.count.total", "ac.Oki_Kum.type.bridge.region.count.total", "ac.Oki_Aom.type.bridge.region.count.total", "ac.Oki_Nor.type.bridge.region.count.total") |> as.data.frame() |> rowMeans()

mcols(chrt$Osa) <- cbind(mcols(chrt$Osa), mcols(grTiles(genome=genomes$Osa, meta=wgo$Osa_Oki, n=nTiles, meta_prefix = 'ac.Osa_Oki.')))
mcols(chrt$Osa) <- cbind(mcols(chrt$Osa), mcols(grTiles(genome=genomes$Osa, meta=wgo$Osa_Bar, n=nTiles, meta_prefix = 'ac.Osa_Bar.')))
mcols(chrt$Osa) <- cbind(mcols(chrt$Osa), mcols(grTiles(genome=genomes$Osa, meta=wgo$Osa_Kum, n=nTiles, meta_prefix = 'ac.Osa_Kum.')))
mcols(chrt$Osa) <- cbind(mcols(chrt$Osa), mcols(grTiles(genome=genomes$Osa, meta=wgo$Osa_Aom, n=nTiles, meta_prefix = 'ac.Osa_Aom.')))
mcols(chrt$Osa) <- cbind(mcols(chrt$Osa), mcols(grTiles(genome=genomes$Osa, meta=wgo$Osa_Nor, n=nTiles, meta_prefix = 'ac.Osa_Nor.')))
chrt$Osa$ac.isolated.mean <- chrt$Osa |> as.data.frame()|> select("ac.Osa_Oki.type.isolated.alignment.count.total", "ac.Osa_Bar.type.isolated.alignment.count.total", "ac.Osa_Kum.type.isolated.alignment.count.total", "ac.Osa_Aom.type.isolated.alignment.count.total", "ac.Osa_Nor.type.isolated.alignment.count.total") |> as.data.frame() |> rowMeans()
chrt$Osa$ac.collinear.mean <- chrt$Osa |> as.data.frame()|> select("ac.Osa_Oki.type.collinear.alignment.count.total", "ac.Osa_Bar.type.collinear.alignment.count.total", "ac.Osa_Kum.type.collinear.alignment.count.total", "ac.Osa_Aom.type.collinear.alignment.count.total", "ac.Osa_Nor.type.collinear.alignment.count.total") |> as.data.frame() |> rowMeans()
chrt$Osa$ac.breakpoint.mean <- chrt$Osa |> as.data.frame()|> select("ac.Osa_Oki.type.breakpoint.region.count.total", "ac.Osa_Bar.type.breakpoint.region.count.total", "ac.Osa_Kum.type.breakpoint.region.count.total", "ac.Osa_Aom.type.breakpoint.region.count.total", "ac.Osa_Nor.type.breakpoint.region.count.total") |> as.data.frame() |> rowMeans()
chrt$Osa$ac.bridge.mean <- chrt$Osa |> as.data.frame()|> select("ac.Osa_Oki.type.bridge.region.count.total", "ac.Osa_Bar.type.bridge.region.count.total", "ac.Osa_Kum.type.bridge.region.count.total", "ac.Osa_Aom.type.bridge.region.count.total", "ac.Osa_Nor.type.bridge.region.count.total") |> as.data.frame() |> rowMeans()

mcols(chrt$Bar) <- cbind(mcols(chrt$Bar), mcols(grTiles(genome=genomes$Bar, meta=wgo$Bar_Oki, n=nTiles, meta_prefix = 'ac.Bar_Oki.')))
mcols(chrt$Bar) <- cbind(mcols(chrt$Bar), mcols(grTiles(genome=genomes$Bar, meta=wgo$Bar_Osa, n=nTiles, meta_prefix = 'ac.Bar_Osa.')))
mcols(chrt$Bar) <- cbind(mcols(chrt$Bar), mcols(grTiles(genome=genomes$Bar, meta=wgo$Bar_Kum, n=nTiles, meta_prefix = 'ac.Bar_Kum.')))
mcols(chrt$Bar) <- cbind(mcols(chrt$Bar), mcols(grTiles(genome=genomes$Bar, meta=wgo$Bar_Aom, n=nTiles, meta_prefix = 'ac.Bar_Aom.')))
mcols(chrt$Bar) <- cbind(mcols(chrt$Bar), mcols(grTiles(genome=genomes$Bar, meta=wgo$Bar_Nor, n=nTiles, meta_prefix = 'ac.Bar_Nor.')))
chrt$Bar$ac.isolated.mean <- chrt$Bar |> as.data.frame()|> select("ac.Bar_Oki.type.isolated.alignment.count.total", "ac.Bar_Osa.type.isolated.alignment.count.total", "ac.Bar_Kum.type.isolated.alignment.count.total", "ac.Bar_Aom.type.isolated.alignment.count.total", "ac.Bar_Nor.type.isolated.alignment.count.total") |> as.data.frame() |> rowMeans()
chrt$Bar$ac.collinear.mean <- chrt$Bar |> as.data.frame()|> select("ac.Bar_Oki.type.collinear.alignment.count.total", "ac.Bar_Osa.type.collinear.alignment.count.total", "ac.Bar_Kum.type.collinear.alignment.count.total", "ac.Bar_Aom.type.collinear.alignment.count.total", "ac.Bar_Nor.type.collinear.alignment.count.total") |> as.data.frame() |> rowMeans()
chrt$Bar$ac.breakpoint.mean <- chrt$Bar |> as.data.frame()|> select("ac.Bar_Oki.type.breakpoint.region.count.total", "ac.Bar_Osa.type.breakpoint.region.count.total", "ac.Bar_Kum.type.breakpoint.region.count.total", "ac.Bar_Aom.type.breakpoint.region.count.total", "ac.Bar_Nor.type.breakpoint.region.count.total") |> as.data.frame() |> rowMeans()
chrt$Bar$ac.bridge.mean <- chrt$Bar |> as.data.frame()|> select("ac.Bar_Oki.type.bridge.region.count.total", "ac.Bar_Osa.type.bridge.region.count.total", "ac.Bar_Kum.type.bridge.region.count.total", "ac.Bar_Aom.type.bridge.region.count.total", "ac.Bar_Nor.type.bridge.region.count.total") |> as.data.frame() |> rowMeans()


# Now, combining it all into a single unified tiled chromosome object.
chrt_unified <- data.frame(seqnames=c(rep('Chr1', nTiles), rep('Chr2', nTiles), rep('PAR', nTiles), rep('XSR', nTiles)), start=c(seq(1, nTiles), seq(1, nTiles), seq(1, nTiles), seq(1, nTiles)), end=c(seq(1, nTiles)+1, seq(1, nTiles)+1, seq(1, nTiles)+1, seq(1, nTiles)+1) )  |> GRanges()
seqlevels(chrt_unified) <- c('Chr1', 'Chr2', 'PAR', 'XSR')

# Add a prefix to mcols to prevent collisions between colnames between similar objects.
prefix_cbind_mcols <- function(gr1, gr2, prefix) {
  gr <- gr1
  q_df <- mcols(gr1)
  t_df <- mcols(gr2)
  colnames(t_df) <- lapply(colnames(t_df), function(x) paste0(prefix, '.', x)) |> unlist()
  mcols(gr) <- cbind(q_df, t_df)
  gr
}
chrt_unified <- prefix_cbind_mcols(chrt_unified, chrt$Oki |> as.data.frame() |> filter(seqnames %in% c('chr1', 'chr2', 'PAR', 'XSR')) |> GRanges() , prefix='Oki')
chrt_unified <- prefix_cbind_mcols(chrt_unified, chrt$Osa |> as.data.frame() |> filter(seqnames %in% c('Chr1', 'Chr2', 'PAR', 'XSR')) |> GRanges() , prefix='Osa')
chrt_unified <- prefix_cbind_mcols(chrt_unified, chrt$Bar |> as.data.frame() |> filter(seqnames %in% c('Chr1', 'Chr2', 'PAR', 'XSR')) |> GRanges() , prefix='Bar')

# Making some mean values across all species
chrt_unified$transcripts.count.mean <- data.frame(chrt_unified$Oki.transcripts.tx_id.count, chrt_unified$Osa.transcripts.tx_id.count, chrt_unified$Bar.transcripts.tx_id.count) |> rowMeans()

chrt_unified$transcripts.length.mean <- data.frame(chrt_unified$Oki.transcripts.tx_len.mean, chrt_unified$Osa.transcripts.tx_len.mean, chrt_unified$Bar.transcripts.tx_len.mean) |> rowMeans()

chrt_unified$operons.count.mean <- data.frame(chrt_unified$Oki.operons.n.count, chrt_unified$Osa.operons.n.count, chrt_unified$Bar.operons.n.count) |> rowMeans()
chrt_unified$operons.length.mean <- data.frame(chrt_unified$Oki.operons.n.mean, chrt_unified$Osa.operons.n.mean, chrt_unified$Bar.operons.n.mean) |> rowMeans()

chrt_unified$transcripts.dNdS.mean <- data.frame(chrt_unified$Oki.transcripts.dNdS_GUIDANCE2.median, chrt_unified$Osa.transcripts.dNdS_GUIDANCE2.median, chrt_unified$Bar.transcripts.dNdS_GUIDANCE2.median) |> rowMeans()

chrt_unified$repeats.count.mean <- data.frame(chrt_unified$Oki.repeats.type.count.total, chrt_unified$Osa.repeats.type.count.total, chrt_unified$Bar.repeats.type.count.total) |> rowMeans()

chrt_unified$breakpoints.count.mean <- data.frame(chrt_unified$Oki.bp.mean.breaks, chrt_unified$Osa.bp.mean.breaks, chrt_unified$Bar.bp.mean.breaks) |> rowMeans()

chrt_unified$categories.isolated.mean <- data.frame(chrt_unified$Oki.ac.isolated.mean, chrt_unified$Osa.ac.isolated.mean, chrt_unified$Bar.ac.isolated.mean) |> rowMeans()
chrt_unified$categories.collinear.mean <- data.frame(chrt_unified$Oki.ac.collinear.mean, chrt_unified$Osa.ac.collinear.mean, chrt_unified$Bar.ac.collinear.mean) |> rowMeans()
chrt_unified$categories.breakpoint.mean <- data.frame(chrt_unified$Oki.ac.breakpoint.mean, chrt_unified$Osa.ac.breakpoint.mean, chrt_unified$Bar.ac.breakpoint.mean) |> rowMeans()
chrt_unified$categories.bridge.mean <- data.frame(chrt_unified$Oki.ac.bridge.mean, chrt_unified$Osa.ac.bridge.mean, chrt_unified$Bar.ac.bridge.mean) |> rowMeans()
```

```
# ggplot(chrt_unified |> as.data.frame()) +
#   geom_smooth(aes(x=start, y=transcripts.count.mean/max(transcripts.count.mean, na.rm=T), col='transcripts'), se=FALSE) +
#   geom_smooth(aes(x=start, y=repeats.count.mean/max(repeats.count.mean, na.rm = T), col='repeats'), se=FALSE) +
#   geom_smooth(aes(x=start, y=transcripts.dNdS.mean/max(transcripts.dNdS.mean, na.rm = T), col='dN/dS'), se=FALSE) +
#   geom_smooth(aes(x=start, y=operons.count.mean/max(operons.count.mean, na.rm = T), col='operons'), se=FALSE) +
#   geom_smooth(aes(x=start, y=breakpoints.count.mean/max(breakpoints.count.mean, na.rm = T), col='breakpoints'), se=FALSE) +
#   ylab('pct') +
#   facet_wrap(~seqnames, nrow=1) +
#   ggtitle('Tiles (averages across Oki, Osa, Bar): Properties across genome')
```

What features correlate with breakpoints?

```
tb <- chrt_unified |> as.data.frame()
tb$seqnames_arm <- paste0(tb$seqnames, "_", tb$Oki.Arm)

p1 <- ggplot(tb) + aes(x=breakpoints.count.mean, y=transcripts.dNdS.mean) + geom_point() + geom_smooth(method='lm', col='red' , formula=y~x) + ggpubr::stat_cor() + facet_wrap(seqnames~Oki.Arm, nrow=1, scales = 'free') + ylab('dN/dS')

p2 <- ggplot(tb) + aes(x=breakpoints.count.mean, y=transcripts.count.mean) + geom_point() + geom_smooth(method='lm', col='red' , formula=y~x) + ggpubr::stat_cor() + facet_wrap(seqnames~Oki.Arm, nrow=1, scales = 'free') + ylab('transcripts')

p3 <- ggplot(tb) + aes(x=breakpoints.count.mean, y=operons.count.mean) + geom_point() + geom_smooth(method='lm', col='red' , formula=y~x) + ggpubr::stat_cor() + facet_wrap(seqnames~Oki.Arm, nrow=1, scales = 'free') + ylab('transcripts')

p4 <- ggplot(tb) + aes(x=breakpoints.count.mean, y=repeats.count.mean) + geom_point() + geom_smooth(method='lm', col='red' , formula=y~x) + ggpubr::stat_cor() + facet_wrap(seqnames~Oki.Arm, nrow=1, scales = 'free') + ylab('repeats')

( p1 / p2 / p3 / p4)
```

What alignment categories correlate with alignment categories?

```
tb <- chrt_unified |> as.data.frame()
tb$seqnames_arm <- paste0(tb$seqnames, "_", tb$Oki.Arm)

p1 <- ggplot(tb) + aes(x=breakpoints.count.mean, y=categories.isolated.mean) + geom_point() + geom_smooth(method='lm', col='red' , formula=y~x) + ggpubr::stat_cor() + facet_wrap(seqnames~Oki.Arm, nrow=1, scales = 'free') + ylab('isolated')

p2 <- ggplot(tb) + aes(x=breakpoints.count.mean, y=categories.collinear.mean) + geom_point() + geom_smooth(method='lm', col='red' , formula=y~x) + ggpubr::stat_cor() + facet_wrap(seqnames~Oki.Arm, nrow=1, scales = 'free') + ylab('collinear')

p3 <- ggplot(tb) + aes(x=breakpoints.count.mean, y=categories.breakpoint.mean) + geom_point() + geom_smooth(method='lm', col='red' , formula=y~x) + ggpubr::stat_cor() + facet_wrap(seqnames~Oki.Arm, nrow=1, scales = 'free') + ylab('breakpoint')

p4 <- ggplot(tb) + aes(x=breakpoints.count.mean, y=categories.bridge.mean) + geom_point() + geom_smooth(method='lm', col='red' , formula=y~x) + ggpubr::stat_cor() + facet_wrap(seqnames~Oki.Arm, nrow=1, scales = 'free') + ylab('bridge')

( p1 / p2 / p3 / p4)
```

```
# ggplot(chrt_unified |> as.data.frame()) +
#   geom_smooth(aes(x=start, y=categories.isolated.mean,  col='isolated'), se=FALSE) +
#   geom_smooth(aes(x=start, y=categories.collinear.mean, col='collinear'), se=FALSE) +
#   geom_smooth(aes(x=start, y=categories.breakpoint.mean,  col='breakpoint'), se=FALSE) +
#   geom_smooth(aes(x=start, y=categories.bridge.mean,    col='bridge'), se=FALSE) +
#   ylab('pct') +
#   facet_wrap(~seqnames, nrow=1) +
#   ggtitle('Tiles (averages across Oki, Osa, Bar): Alignment categories by location')
```

#### Gene size and density vs. operon size and density.

```
tb <- chrt_unified |> as.data.frame()
tb$seqnames_arm <- paste0(tb$seqnames, "_", tb$Oki.Arm)
tb$Arm <- tb$Oki.Arm

p0 <- ggplot(tb) +
  geom_smooth(aes(x=start, y=transcripts.count.mean/max(transcripts.count.mean, na.rm = T),  col='gene density'), formula=y~x, method='loess', se=FALSE) +
  geom_smooth(aes(x=start, y=transcripts.length.mean/max(transcripts.length.mean, na.rm = T),  col='gene length'), formula=y~x, method='loess', se=FALSE) +
  geom_smooth(aes(x=start, y=operons.length.mean/max(operons.length.mean,na.rm=T),  col='operon length'), formula=y~x, method='loess', se=FALSE) +
  geom_smooth(aes(x=start, y=operons.count.mean/max(operons.count.mean,na.rm=T),  col='operon density'), formula=y~x, method='loess', se=FALSE) +
  geom_smooth(aes(x=start, y=transcripts.dNdS.mean/max(transcripts.dNdS.mean, na.rm = T),  col='dN/dS'), formula=y~x, method='loess', se=FALSE) +
  geom_smooth(aes(x=start, y=breakpoints.count.mean/max(breakpoints.count.mean, na.rm = T),  col='breakpoints'), formula=y~x, method='loess', se=FALSE) +
  ylab("Normalized mean per tile across all species") +
  facet_wrap(~seqnames, nrow=1)

p1 <- ggplot(tb) +
  geom_smooth(aes(x=start, y=transcripts.count.mean/max(transcripts.count.mean, na.rm = T),  col='mean gene density'), formula=y~x, method='loess', se=FALSE) +
  geom_smooth(aes(x=start, y=transcripts.length.mean/max(transcripts.length.mean, na.rm = T),  col='mean gene length'), formula=y~x, method='loess', se=FALSE) +
  geom_smooth(aes(x=start, y=operons.length.mean/max(operons.length.mean,na.rm=T),  col='mean operon length'), formula=y~x, method='loess', se=FALSE) +
  geom_smooth(aes(x=start, y=operons.count.mean/max(operons.count.mean,na.rm=T),  col='mean operon density'), formula=y~x, method='loess', se=FALSE) +
  ylab("Normalized mean per tile across all species") +
  facet_wrap(seqnames~Oki.Arm, nrow=1)

p2 <- tb |> per_chrom_plot() + aes(y=transcripts.count.mean)  + ylab('gene density')
p3 <- tb |> per_chrom_plot() + aes(y=transcripts.length.mean) + ylab('gene size')
p4 <- tb |> per_chrom_plot() + aes(y=operons.count.mean)      + ylab('operon density')
p5 <- tb |> per_chrom_plot() + aes(y=operons.length.mean)     + ylab('operon size')
p6 <- tb |> per_chrom_plot() + aes(y=transcripts.dNdS.mean)   + ylab('dN/dS')
p7 <- tb |> per_chrom_plot() + aes(y=breakpoints.count.mean)  + ylab('breakpoints')
p8 <- tb |> per_chrom_plot() + aes(y=repeats.count.mean)      + ylab('repeats')

p9 <- ggplot(tb) + aes(x=breakpoints.count.mean, y=transcripts.dNdS.mean) + geom_point() + geom_smooth(method='lm', col='red' , formula=y~x) + ggpubr::stat_cor() + facet_wrap(seqnames~Oki.Arm, nrow=1, scales = 'free') + ylab('dN/dS') + theme(axis.title.x=element_blank())

p10 <- ggboxplot(tb, x='Arm', y='operons.length.mean', color='Oki.Arm', add='jitter') + theme(axis.text.x=element_text(angle=90)) + ylab('operon size') + stat_compare_means(comparison=list(c('short', 'long'), c('short', 'XSR'), c('long', 'XSR')), paired = FALSE, na.rm = TRUE, method='t.test', size=2.8) + theme_bw()

p11 <- ggboxplot(tb, x='Arm', y='breakpoints.count.mean', color='Oki.Arm', add='jitter') + theme(axis.text.x=element_text(angle=90)) + ylab('breakpoints') + stat_compare_means(comparison=list(c('short', 'long'), c('short', 'XSR'), c('long', 'XSR')), paired = FALSE, na.rm = TRUE, method='t.test', size=2.8) + theme_bw()

p12 <- ggboxplot(tb, x='Arm', y='transcripts.dNdS.mean', color='Oki.Arm', add='jitter') + theme(axis.text.x=element_text(angle=90)) + ylab('dN/dS') + stat_compare_means(comparison=list(c('short', 'long'), c('short', 'XSR'), c('long', 'XSR')), paired = FALSE, na.rm = TRUE, method='t.test', size=2.8) + theme_bw()
```

```
(p2 / p3 / p4 / p5 / p6 / p7 / p8) # all plots
```

## Figure 5

### Figure 5

```
(p5 / p7 / p6) # Figure 4A
```

### Figure 5B

```
(p10 + p11 + p12) + plot_layout(guides = 'collect') # Figure 4B
```

### Figure S5

See the Figure S5 subsection of the
Repeats section above.

## Extra

### Other representations in a single-coordinate system.

#### Gene size and density vs. operon size and density, boxplot edition.

```
tb <- chrt_unified |> as.data.frame()
tb$seqnames_arm <- paste0(tb$seqnames, "_", tb$Oki.Arm)

toBoxPlot <- function(p) {
  p$layers <- NULL
  p + geom_boxplot(outlier.shape=NA) +
      geom_point()
}

( ( p2 |> toBoxPlot() ) /
  ( p3 |> toBoxPlot() ) /
  ( p4 |> toBoxPlot() ) /
  ( p5 |> toBoxPlot() ) / 
  ( p6 |> toBoxPlot() ) /
  ( p7 |> toBoxPlot() ) /
  ( p8 |> toBoxPlot() )
) + plot_layout(guides = 'collect')
```

`ggboxplot` version, for statistics.

```
library(ggpubr)
tb <- chrt_unified |> as.data.frame()
tb$seqnames_arm <- paste0(tb$seqnames, "_", tb$Oki.Arm)

p2 <- ggboxplot(tb, x='seqnames_arm', y='transcripts.count.mean', color='Oki.Arm', add='jitter') + theme(axis.text.x=element_text(angle=90)) + ylab('gene density') + stat_compare_means(comparison=list(c('Chr1_short', 'Chr1_long'), c('Chr2_short', 'Chr2_long'), c('PAR_short', 'PAR_long'), c('XSR_XSR', 'PAR_short'), c('XSR_XSR', 'PAR_long')), paired = FALSE, na.rm = TRUE, method='t.test') + theme_classic() + theme(axis.title.x=element_blank())

p3 <- ggboxplot(tb, x='seqnames_arm', y='transcripts.length.mean', color='Oki.Arm', add='jitter') + theme(axis.text.x=element_text(angle=90)) + ylab('gene size') + stat_compare_means(comparison=list(c('Chr1_short', 'Chr1_long'), c('Chr2_short', 'Chr2_long'), c('PAR_short', 'PAR_long'), c('XSR_XSR', 'PAR_short'), c('XSR_XSR', 'PAR_long')), paired = FALSE, na.rm = TRUE, method='t.test') + theme_classic() + theme(axis.title.x=element_blank())

p4 <- ggboxplot(tb, x='seqnames_arm', y='operons.count.mean', color='Oki.Arm', add='jitter') + theme(axis.text.x=element_text(angle=90)) + ylab('operon density') + stat_compare_means(comparison=list(c('Chr1_short', 'Chr1_long'), c('Chr2_short', 'Chr2_long'), c('PAR_short', 'PAR_long'), c('XSR_XSR', 'PAR_short'), c('XSR_XSR', 'PAR_long')), paired = FALSE, na.rm = TRUE, method='t.test') + theme_classic() + theme(axis.title.x=element_blank())

p5 <- ggboxplot(tb, x='seqnames_arm', y='operons.length.mean', color='Oki.Arm', add='jitter') + theme(axis.text.x=element_text(angle=90)) + ylab('operon size') + stat_compare_means(comparison=list(c('Chr1_short', 'Chr1_long'), c('Chr2_short', 'Chr2_long'), c('PAR_short', 'PAR_long'), c('XSR_XSR', 'PAR_short'), c('XSR_XSR', 'PAR_long')), paired = FALSE, na.rm = TRUE, method='t.test') + theme_classic() + theme(axis.title.x=element_blank())

p6 <- ggboxplot(tb, x='seqnames_arm', y='transcripts.dNdS.mean', color='Oki.Arm', add='jitter') + theme(axis.text.x=element_text(angle=90)) + ylab('dN/dS') + stat_compare_means(comparison=list(c('Chr1_short', 'Chr1_long'), c('Chr2_short', 'Chr2_long'), c('PAR_short', 'PAR_long'), c('XSR_XSR', 'PAR_short'), c('XSR_XSR', 'PAR_long')), paired = FALSE, na.rm = TRUE, method='t.test') + theme_classic() + theme(axis.title.x=element_blank())

p7 <- ggboxplot(tb, x='seqnames_arm', y='breakpoints.count.mean', color='Oki.Arm', add='jitter') + theme(axis.text.x=element_text(angle=90)) + ylab('breakpoints') + stat_compare_means(comparison=list(c('Chr1_short', 'Chr1_long'), c('Chr2_short', 'Chr2_long'), c('PAR_short', 'PAR_long'), c('XSR_XSR', 'PAR_short'), c('XSR_XSR', 'PAR_long')), paired = FALSE, na.rm = TRUE, method='t.test') + theme_classic() + theme(axis.title.x=element_blank())

p8 <- ggboxplot(tb, x='seqnames_arm', y='repeats.count.mean', color='Oki.Arm', add='jitter') + theme(axis.text.x=element_text(angle=90)) + ylab('repeats') + stat_compare_means(comparison=list(c('Chr1_short', 'Chr1_long'), c('Chr2_short', 'Chr2_long'), c('PAR_short', 'PAR_long'), c('XSR_XSR', 'PAR_short'), c('XSR_XSR', 'PAR_long')), paired = FALSE, na.rm = TRUE, method='t.test') + theme_classic() + theme(axis.title.x=element_blank())

(p2 / p3 / p4 / p5 / p6 / p7 / p8 )
```

```
chrt_unified |>
  as.data.frame() |>
  subset(select=c("seqnames", "start", "end", "Oki.Arm", "categories.isolated.mean", "categories.collinear.mean", "categories.breakpoint.mean", "categories.bridge.mean" )) |>
 tidyr::pivot_longer(c("categories.isolated.mean", "categories.collinear.mean", "categories.breakpoint.mean", "categories.bridge.mean")) |>
  ggplot() +
    aes(value, paste0(seqnames, "_", Oki.Arm),  fill = name) +
    geom_bar(position='fill', stat='identity') +
    ylab("chr and arm") +
    ggtitle("Tiles (averages across Oki, Osa, Bar): Alignment categories by location")
```

```
library(ggpubr)
tb <- chrt_unified |> as.data.frame()
tb$seqnames_arm <- paste0(tb$seqnames, "_", tb$Oki.Arm)

p0 <- tb |> subset(select=c("seqnames", "seqnames_arm", "start", "end", "Oki.Arm", "categories.isolated.mean", "categories.collinear.mean", "categories.breakpoint.mean", "categories.bridge.mean" )) |> tidyr::pivot_longer(c("categories.isolated.mean", "categories.collinear.mean", "categories.breakpoint.mean", "categories.bridge.mean")) |>
   ggplot() +
    aes(value, seqnames_arm,  fill = name) +
    facet_wrap(seqnames~Oki.Arm, nrow=1, scales='free') +
    geom_bar(position='fill', stat='identity') +
    theme(axis.text.y=element_blank(), axis.ticks.y =element_blank()) +
    ylab("chr and arm") +
    xlab("category")
p0 + ggtitle("Tiles (averages across Oki, Osa, Bar): Alignment categories across genome")
```

```
p1 <- ggplot( tb ) + aes(x=start, y=transcripts.dNdS.mean, col = Oki.Arm) + geom_point() + facet_wrap(~seqnames, nrow = 1) + geom_smooth(formula=y~x, method = 'loess', method.args = list(family = "symmetric")) + ylab('dN/dS') 
p1 + ggtitle("Tiles (averages across Oki, Osa, Bar): Properties across genome")
```

```
p2 <- ggplot( tb ) + aes(x=start, y=breakpoints.count.mean , col = Oki.Arm) + geom_point() + facet_wrap(~seqnames, nrow = 1) + geom_smooth(formula=y~x, method = 'loess', method.args = list(family = "symmetric")) + ylab('breakpoints') 
p2 + ggtitle("Tiles (averages across Oki, Osa, Bar): Properties across genome")
```

```
p3 <- ggboxplot(tb, x='seqnames_arm', y='breakpoints.count.mean', color='Oki.Arm', add='jitter') +
  theme(axis.text.x=element_text(angle=90)) +
  ylab('breakpoints') +
  stat_compare_means(comparison=list(c('Chr1_short', 'Chr1_long'), c('Chr2_short', 'Chr2_long'), c('PAR_short', 'PAR_long'), c('XSR_XSR', 'PAR_short'), c('XSR_XSR', 'PAR_long')), paired = FALSE, na.rm = TRUE, method='t.test') +
  theme_classic()
p3 + ggtitle('Tiles (averages across Oki, Osa, Bar): Breakpoints per chr+arm')
```

```
p4 <- ggboxplot(tb, x='seqnames_arm', y='transcripts.dNdS.mean', color='Oki.Arm', add='jitter') +
  theme(axis.text.x=element_text(angle=90)) +
  ylab('dN/dS') +
  stat_compare_means(comparison=list(c('Chr1_short', 'Chr1_long'), c('Chr2_short', 'Chr2_long'), c('PAR_short', 'PAR_long'), c('XSR_XSR', 'PAR_short'), c('XSR_XSR', 'PAR_long')), paired = FALSE, na.rm = TRUE, method='t.test') +
  theme_classic()
p4 +  ggtitle('Tiles (averages across Oki, Osa, Bar): dNdS per chr+arm')
```

```
# And a correlation plot
p5 <- ggplot(tb) + aes(x=breakpoints.count.mean, y=transcripts.dNdS.mean) + geom_point() + geom_smooth(method='lm', col='red' , formula=y~x) + ggpubr::stat_cor() + facet_wrap(seqnames~Oki.Arm, nrow=1, scales = 'free') + ylab('dN/dS')

p5 + ggtitle('Tiles (averages across Oki, Osa, Bar): dNdS per chr+arm')
```

```
p6 <- ggplot(tb) + aes(x=breakpoints.count.mean, y=repeats.count.mean) + geom_point() + geom_smooth(method='lm', col='red' , formula=y~x) + ggpubr::stat_cor() + facet_wrap(seqnames~Oki.Arm, nrow=1, scales = 'free') + ylab('repeats')

p6
```

```
layout <- "
A
B
C
D
E
F
"

( p0 / p1 / p4 / p2 / p3 / p5 ) #+ plot_layout(design = layout, guides = 'collect')
```

```
( p0 / p1 / p4 / p2 / p3 )
```

```
( p1 / p2 / p3 / p4 )
```

```
( p1 / p2 / p3 )
```

```
( p0 / p1 / p2 / p3 )
```

```
detach('package:ggpubr')
```

#### Version with boxplots superimposed on chromosomes.

```
library(ggpubr)
tb <- chrt_unified |> as.data.frame()
tb$seqnames_arm <- paste0(tb$seqnames, "_", tb$Oki.Arm)
#tb$seqnames_arm <- paste0(tb$seqnames, "_", tb$Osa.Arm)
#tb$seqnames_arm <- paste0(tb$seqnames, "_", tb$Bar.Arm)

p0 <- tb |> subset(select=c("seqnames", "seqnames_arm", "start", "end", "Oki.Arm", "categories.isolated.mean", "categories.collinear.mean", "categories.breakpoint.mean", "categories.bridge.mean" )) |> tidyr::pivot_longer(c("categories.isolated.mean", "categories.collinear.mean", "categories.breakpoint.mean", "categories.bridge.mean")) |>
   ggplot() +
    aes(value, seqnames_arm,  fill = name) +
    facet_wrap(seqnames~Oki.Arm, nrow=1, scales='free') +
    geom_bar(position='fill', stat='identity') +
    theme(axis.text.y=element_blank(), axis.ticks.y =element_blank()) +
    ylab("chr and arm") +
    xlab("category")

p0 + ggtitle("Tiles (averages across Oki, Osa, Bar): Alignment categories by position")
```

```
p1 <- ggplot(tb) + aes(x=start, y=transcripts.dNdS.mean, col = Oki.Arm) + geom_boxplot(outlier.shape=NA) + geom_point() + facet_wrap(~seqnames, nrow = 1) + ylab('dN/dS')
p1 + ggtitle("Tiles (averages across Oki, Osa, Bar): dN/dS across genome")
```

```
p1 <- p1 + theme(axis.title.x=element_blank()) # remove X labels for later

p2 <- ggplot( chrt_unified |> as.data.frame() ) + aes(x=start, y=breakpoints.count.mean , col = Oki.Arm) + geom_boxplot(outlier.shape=NA) + geom_point() + facet_wrap(~seqnames, nrow = 1) + ylab('Breakpoints')
p2 + ggtitle("Tiles (averages across Oki, Osa, Bar): Breakpoints across genome")
```

```
# And a correlation plot
p3 <- ggplot(tb) + aes(x=breakpoints.count.mean, y=transcripts.dNdS.mean) + geom_point() + geom_smooth(method='lm', col='red' , formula=y~x) + ggpubr::stat_cor() + facet_wrap(seqnames~Oki.Arm, nrow=1, scales = 'free') + ylab('dN/dS')

p3 + ggtitle('Tiles (averages across Oki, Osa, Bar): dNdS per chr+arm')
```

```
p3 <- p3 + theme(axis.title.x=element_blank()) # remove X labels for later

p4 <- ggplot(tb) + aes(x=breakpoints.count.mean, y=repeats.count.mean) + geom_point() + geom_smooth(method='lm', col='red' , formula=y~x) + ggpubr::stat_cor() + facet_wrap(seqnames~Oki.Arm, nrow=1, scales = 'free') + ylab('repeats') + xlab('Breakpoints')
p4
```

```
( p0 / p1 / p2 / p3 / p4 )
```

### Edges vs. centres of chromosomes

```
# Flag the first and last (percentile) of total width for every chromosome.
flag_chromosome_percentiles <- function(chrt, percentile=0.1) {
  original_names <- names(chrt)
  gr_list <- split(chrt, seqnames(chrt))
  edge_annotations <- endoapply(gr_list, function(gr){
    gr$chr.edge <- "centre"
    total_length <- gr |> width() |> sum()
    length_percentile <- end(gr)/total_length
    cumulative_length <- cumsum(gr |> width())
    cumulative_percentile <- cumulative_length/total_length

    gr$chr.edge[cumulative_percentile<percentile] <- "edge"
    gr$chr.edge[cumulative_percentile>(1-percentile)] <- "edge"
    
    gr
  })
  edge_annotations <- unlist(edge_annotations)
  names(edge_annotations) <- original_names
  edge_annotations
}
#chrt_unified |> flag_chromosome_percentiles()

# Flag the first and last (percentile) of total width for every chromosome's individual arm.
flag_chromosome_arm_percentiles <- function(chrt, percentile=0.1, arm="Oki.Arm") {
  original_names <- names(chrt)
  chrt$seqnames_arm <- paste0(seqnames(chrt), "_", mcols(chrt)[[arm]])
  gr_list <- split(chrt, chrt$seqnames_arm)
  edge_annotations <- endoapply(gr_list, function(gr){
    gr$chr.arm.edge <- "centre"
    total_length <- gr |> width() |> sum()
    length_percentile <- end(gr)/total_length
    cumulative_length <- cumsum(gr |> width())
    cumulative_percentile <- cumulative_length/total_length

    gr$chr.arm.edge[cumulative_percentile<percentile] <- "edge"
    gr$chr.arm.edge[cumulative_percentile>(1-percentile)] <- "edge"
    
    gr
  })
  edge_annotations <- unlist(edge_annotations)
  names(edge_annotations) <- original_names
  edge_annotations
}
```

Assess the edges vs. centres of chromosomes for feature
densities/counts.

```
library(ggpubr)
compare_method = "t.test"
#compare_method = "wilcox.test"

tb <- chrt_unified |> flag_chromosome_percentiles() |> flag_chromosome_arm_percentiles() |> as.data.frame()
tb$seqnames_arm_edge <- paste0(tb$seqnames_arm, "_", tb$chr.arm.edge)
tb$seqnames_arm_edge <- factor(tb$seqnames_arm_edge, levels=c("Chr1_short_edge", "Chr1_short_centre", "Chr1_long_edge",  "Chr1_long_centre", "Chr2_short_edge", "Chr2_short_centre", "Chr2_long_edge", "Chr2_long_centre", "PAR_short_edge", "PAR_short_centre", "PAR_long_edge", "PAR_long_centre", "XSR_XSR_edge", "XSR_XSR_centre"))

p1 <- ggboxplot(tb, x='seqnames_arm_edge', y='repeats.count.mean', color='seqnames_arm', add='jitter') + ylab('repeats') + stat_compare_means(comparison=list(c('Chr1_short_edge', 'Chr1_short_centre'), c('Chr1_long_edge', 'Chr1_long_centre'), c('Chr2_short_edge', 'Chr2_short_centre'), c('Chr2_long_edge', 'Chr2_long_centre'), c('PAR_short_edge', 'PAR_short_centre'), c('PAR_long_edge', 'PAR_long_centre'), c('XSR_XSR_edge', 'XSR_XSR_centre')), label='p.signif', paired = FALSE, na.rm = TRUE, method=compare_method) + theme_classic() + theme(axis.text.x=element_text(angle=90))
p1 + ggtitle('Tiles (averages across Oki, Osa, Bar): Repeats per chr+arm, edges vs. centres')
```

```
p1 <- ggboxplot(tb, x='chr.arm.edge', y='repeats.count.mean', color='chr.arm.edge', add='jitter') + ylab('repeats') + stat_compare_means(comparison=list(c('centre', 'edge')), label='p.signif', paired = FALSE, na.rm = TRUE, method=compare_method) + theme_classic() + theme(axis.text.x=element_text(angle=90))
p1 + ggtitle('Tiles (averages across Oki, Osa, Bar): Repeats per chr+arm, edges vs. centres')
```

```
p2 <- ggboxplot(tb, x='seqnames_arm_edge', y='transcripts.count.mean', color='seqnames_arm', add='jitter') + ylab('transcripts') + stat_compare_means(comparison=list(c('Chr1_short_edge', 'Chr1_short_centre'), c('Chr1_long_edge', 'Chr1_long_centre'), c('Chr2_short_edge', 'Chr2_short_centre'), c('Chr2_long_edge', 'Chr2_long_centre'), c('PAR_short_edge', 'PAR_short_centre'), c('PAR_long_edge', 'PAR_long_centre'), c('XSR_XSR_edge', 'XSR_XSR_centre')), label='p.signif', paired = FALSE, na.rm = TRUE, method=compare_method) + theme_classic() + theme(axis.text.x=element_text(angle=90))
p2 + ggtitle('Tiles (averages across Oki, Osa, Bar): Transcript number per chr+arm, edges vs. centres')
```

```
p2 <- ggboxplot(tb, x='chr.arm.edge', y='transcripts.count.mean', color='chr.arm.edge', add='jitter') + ylab('transcripts') + stat_compare_means(comparison=list(c('centre', 'edge')), label='p.signif', paired = FALSE, na.rm = TRUE, method=compare_method) + theme_classic() + theme(axis.text.x=element_text(angle=90))
p2 + ggtitle('Tiles (averages across Oki, Osa, Bar): Transcript number per chr+arm, edges vs. centres')
```

```
p3 <- ggboxplot(tb, x='seqnames_arm_edge', y='transcripts.length.mean', color='seqnames_arm', add='jitter') + ylab('transcript length') + stat_compare_means(comparison=list(c('Chr1_short_edge', 'Chr1_short_centre'), c('Chr1_long_edge', 'Chr1_long_centre'), c('Chr2_short_edge', 'Chr2_short_centre'), c('Chr2_long_edge', 'Chr2_long_centre'), c('PAR_short_edge', 'PAR_short_centre'), c('PAR_long_edge', 'PAR_long_centre'), c('XSR_XSR_edge', 'XSR_XSR_centre')), label='p.signif', paired = FALSE, na.rm = TRUE, method=compare_method) + theme_classic() + theme(axis.text.x=element_text(angle=90))
p3 + ggtitle('Tiles (averages across Oki, Osa, Bar): Transcript length per chr+arm, edges vs. centres')
```

```
p3 <- ggboxplot(tb, x='chr.arm.edge', y='transcripts.length.mean', color='chr.arm.edge', add='jitter') + ylab('transcript length') + stat_compare_means(comparison=list(c('centre', 'edge')), label='p.signif', paired = FALSE, na.rm = TRUE, method=compare_method) + theme_classic() + theme(axis.text.x=element_text(angle=90))
p3 + ggtitle('Tiles (averages across Oki, Osa, Bar): Transcript length per chr+arm, edges vs. centres')
```

```
p4 <- ggboxplot(tb, x='seqnames_arm_edge', y='operons.count.mean', color='seqnames_arm', add='jitter') + ylab('operon count') + stat_compare_means(comparison=list(c('Chr1_short_edge', 'Chr1_short_centre'), c('Chr1_long_edge', 'Chr1_long_centre'), c('Chr2_short_edge', 'Chr2_short_centre'), c('Chr2_long_edge', 'Chr2_long_centre'), c('PAR_short_edge', 'PAR_short_centre'), c('PAR_long_edge', 'PAR_long_centre'), c('XSR_XSR_edge', 'XSR_XSR_centre')), label='p.signif', paired = FALSE, na.rm = TRUE, method=compare_method) + theme_classic() + theme(axis.text.x=element_text(angle=90))
p4 + ggtitle('Tiles (averages across Oki, Osa, Bar): Operon number per chr+arm, edges vs. centres')
```

```
p4 <- ggboxplot(tb, x='chr.arm.edge', y='operons.count.mean', color='chr.arm.edge', add='jitter') + ylab('operon count') + stat_compare_means(comparison=list(c('centre', 'edge')), label='p.signif', paired = FALSE, na.rm = TRUE, method=compare_method) + theme_classic() + theme(axis.text.x=element_text(angle=90))
p4 + ggtitle('Tiles (averages across Oki, Osa, Bar): Operon number per chr+arm, edges vs. centres')
```

```
p5 <- ggboxplot(tb, x='seqnames_arm_edge', y='operons.length.mean', color='seqnames_arm', add='jitter') + ylab('operon size') + stat_compare_means(comparison=list(c('Chr1_short_edge', 'Chr1_short_centre'), c('Chr1_long_edge', 'Chr1_long_centre'), c('Chr2_short_edge', 'Chr2_short_centre'), c('Chr2_long_edge', 'Chr2_long_centre'), c('PAR_short_edge', 'PAR_short_centre'), c('PAR_long_edge', 'PAR_long_centre'), c('XSR_XSR_edge', 'XSR_XSR_centre')), label='p.signif', paired = FALSE, na.rm = TRUE, method=compare_method) + theme_classic() + theme(axis.text.x=element_text(angle=90))
p5 + ggtitle('Tiles (averages across Oki, Osa, Bar): Operon size per chr+arm, edges vs. centres')
```

```
p5 <- ggboxplot(tb, x='chr.arm.edge', y='operons.length.mean', color='chr.arm.edge', add='jitter') + ylab('operon size') + stat_compare_means(comparison=list(c('centre', 'edge')), label='p.signif', paired = FALSE, na.rm = TRUE, method=compare_method) + theme_classic() + theme(axis.text.x=element_text(angle=90))
p5 + ggtitle('Tiles (averages across Oki, Osa, Bar): Operon size per chr+arm, edges vs. centres')
```

```
p6 <- ggboxplot(tb, x='seqnames_arm_edge', y='breakpoints.count.mean', color='seqnames_arm', add='jitter') + ylab('breakpoints') + stat_compare_means(comparison=list(c('Chr1_short_edge', 'Chr1_short_centre'), c('Chr1_long_edge', 'Chr1_long_centre'), c('Chr2_short_edge', 'Chr2_short_centre'), c('Chr2_long_edge', 'Chr2_long_centre'), c('PAR_short_edge', 'PAR_short_centre'), c('PAR_long_edge', 'PAR_long_centre'), c('XSR_XSR_edge', 'XSR_XSR_centre')), label='p.signif', paired = FALSE, na.rm = TRUE, method=compare_method) + theme_classic() + theme(axis.text.x=element_text(angle=90))
p6 + ggtitle('Tiles (averages across Oki, Osa, Bar): Breakpoints per chr+arm, edges vs. centres')
```

```
p6 <- ggboxplot(tb, x='chr.arm.edge', y='breakpoints.count.mean', color='chr.arm.edge', add='jitter') + ylab('breakpoints') + stat_compare_means(comparison=list(c('centre', 'edge')), label='p.signif', paired = FALSE, na.rm = TRUE, method=compare_method) + theme_classic() + theme(axis.text.x=element_text(angle=90))
p6 + ggtitle('Tiles (averages across Oki, Osa, Bar): Breakpoints per chr+arm, edges vs. centres')
```

```
p7 <- ggboxplot(tb, x='seqnames_arm_edge', y='transcripts.dNdS.mean', color='seqnames_arm', add='jitter') + ylab('dN/dS') + stat_compare_means(comparison=list(c('Chr1_short_edge', 'Chr1_short_centre'), c('Chr1_long_edge', 'Chr1_long_centre'), c('Chr2_short_edge', 'Chr2_short_centre'), c('Chr2_long_edge', 'Chr2_long_centre'), c('PAR_short_edge', 'PAR_short_centre'), c('PAR_long_edge', 'PAR_long_centre'), c('XSR_XSR_edge', 'XSR_XSR_centre')), label='p.signif', paired = FALSE, na.rm = TRUE, method=compare_method) + theme_classic() + theme(axis.text.x=element_text(angle=90))

p7 <- ggboxplot(tb, x='chr.arm.edge', y='transcripts.dNdS.mean', color='chr.arm.edge', add='jitter') + ylab('dN/dS') + stat_compare_means(comparison=list(c('centre', 'edge')), label='p.signif', paired = FALSE, na.rm = TRUE, method=compare_method) + theme_classic() + theme(axis.text.x=element_text(angle=90))
p7 + ggtitle('Tiles (averages across Oki, Osa, Bar): dN/dS per chr+arm, edges vs. centres')
```

```
detach('package:ggpubr')
```

### Pericentromeric and subtelomeric regions

```
# Flag the first and last (percentile) of total width for every chromosome's individual arm.
flag_subtelomeric_pericentromeric <- function(chrt, percentile=0.1, arm="Oki.Arm") {
  original_names <- names(chrt)
  chrt$seqnames_arm <- paste0(seqnames(chrt), "_", mcols(chrt)[[arm]])
  gr_list <- split(chrt, chrt$seqnames_arm)
  edge_annotations <- endoapply(gr_list, function(gr){
    gr$chr.arm.edge <- "centre"
    total_length <- gr |> width() |> sum()
    length_percentile <- end(gr)/total_length
    cumulative_length <- cumsum(gr |> width())
    cumulative_percentile <- cumulative_length/total_length

    gr$chr.arm.label <- "centre"
    gr$chr.arm.label[ mcols(gr)[[arm]] == "short" & cumulative_percentile<percentile ] <- "subtelomeric"
    gr$chr.arm.label[ mcols(gr)[[arm]] == "short" & cumulative_percentile>(1-percentile) ] <- "pericentromeric"
    gr$chr.arm.label[ mcols(gr)[[arm]] == "long" & cumulative_percentile<percentile ] <- "pericentromeric"
    gr$chr.arm.label[ mcols(gr)[[arm]] == "long" & cumulative_percentile>(1-percentile) ] <- "subtelomeric"
    gr$chr.arm.label[ mcols(gr)[[arm]] == "XSR" & cumulative_percentile<percentile ] <- "centre"
    gr$chr.arm.label[ mcols(gr)[[arm]] == "XSR" & cumulative_percentile>(1-percentile) ] <- "subtelomeric"
    gr
  })
  edge_annotations <- unlist(edge_annotations)
  names(edge_annotations) <- original_names
  
  edge_annotations
}
```

```
library(ggpubr)
compare_method = "t.test"
#compare_method = "wilcox.test"

percentile <- 0.1
tb <- chrt_unified |> flag_chromosome_percentiles(percentile = percentile) |> flag_chromosome_arm_percentiles(percentile = percentile) |> flag_subtelomeric_pericentromeric(percentile = percentile) |> as.data.frame()

tb$seqnames_arm_category <- paste0(tb$seqnames_arm, "_", tb$chr.arm.label)

tb$seqnames_arm_category <- factor(tb$seqnames_arm_category, levels=c("Chr1_long_pericentromeric", "Chr1_long_centre", "Chr1_long_subtelomeric", "Chr1_short_subtelomeric", "Chr1_short_centre", "Chr1_short_pericentromeric", "Chr2_long_pericentromeric", "Chr2_long_centre", "Chr2_long_subtelomeric", "Chr2_short_subtelomeric", "Chr2_short_centre", "Chr2_short_pericentromeric", "PAR_long_pericentromeric", "PAR_long_centre", "PAR_long_subtelomeric", "PAR_short_subtelomeric", "PAR_short_centre", "PAR_short_pericentromeric", "XSR_XSR_centre", "XSR_XSR_subtelomeric"))

# Check everything individually.
# Repeats
p1 <- ggboxplot(tb, x='seqnames_arm_category', y='repeats.count.mean', color='seqnames_arm', add='jitter') + ylab('repeats') + theme_classic() + theme(axis.text.x=element_text(angle=90))
p1 + ggtitle('Tiles (averages across Oki, Osa, Bar): repeats by genomic region')
```

```
p2 <- ggboxplot(tb, x='chr.arm.label', y='repeats.count.mean', color='chr.arm.label', add='jitter') + ylab('repeats') + theme_classic() + theme(axis.text.x=element_text(angle=90)) + stat_compare_means(comparison=list(c('pericentromeric', 'centre'), c('pericentromeric', 'subtelomeric'), c('centre', 'subtelomeric')), label='p.signif', paired = FALSE, na.rm = TRUE, method=compare_method)
p2 + ggtitle('Tiles (averages across Oki, Osa, Bar): repeats by genomic region')
```

```
# Genes
p3 <- ggboxplot(tb, x='seqnames_arm_category', y='transcripts.count.mean', color='seqnames_arm', add='jitter') + ylab('transcripts') + theme_classic() + theme(axis.text.x=element_text(angle=90))
p3 + ggtitle('Tiles (averages across Oki, Osa, Bar): transcripts by genomic region')
```

```
p4 <- ggboxplot(tb, x='chr.arm.label', y='transcripts.count.mean', color='chr.arm.label', add='jitter') + ylab('transcripts') + theme_classic() + theme(axis.text.x=element_text(angle=90)) + stat_compare_means(comparison=list(c('pericentromeric', 'centre'), c('pericentromeric', 'subtelomeric'), c('centre', 'subtelomeric')), label='p.signif', paired = FALSE, na.rm = TRUE, method=compare_method)
p4 + ggtitle('Tiles (averages across Oki, Osa, Bar): transcripts by genomic region')
```

```
p5 <- ggboxplot(tb, x='seqnames_arm_category', y='transcripts.length.mean', color='seqnames_arm', add='jitter') + ylab('transcripts') + theme_classic() + theme(axis.text.x=element_text(angle=90))
p5 + ggtitle('Tiles (averages across Oki, Osa, Bar): transcript lengths by genomic region')
```

```
p6 <- ggboxplot(tb, x='chr.arm.label', y='transcripts.length.mean', color='chr.arm.label', add='jitter') + ylab('transcripts') + theme_classic() + theme(axis.text.x=element_text(angle=90)) + stat_compare_means(comparison=list(c('pericentromeric', 'centre'), c('pericentromeric', 'subtelomeric'), c('centre', 'subtelomeric')), label='p.signif', paired = FALSE, na.rm = TRUE, method=compare_method)
p6 + ggtitle('Tiles (averages across Oki, Osa, Bar): transcript lengths by genomic region')
```

```
# Operons
p7 <- ggboxplot(tb, x='seqnames_arm_category', y='operons.count.mean', color='seqnames_arm', add='jitter') + ylab('operons') + theme_classic() + theme(axis.text.x=element_text(angle=90))
p7 + ggtitle('Tiles (averages across Oki, Osa, Bar): operons by genomic region')
```

```
p8 <- ggboxplot(tb, x='chr.arm.label', y='operons.count.mean', color='chr.arm.label', add='jitter') + ylab('operons') + theme_classic() + theme(axis.text.x=element_text(angle=90)) + stat_compare_means(comparison=list(c('pericentromeric', 'centre'), c('pericentromeric', 'subtelomeric'), c('centre', 'subtelomeric')), label='p.signif', paired = FALSE, na.rm = TRUE, method=compare_method)
p8 + ggtitle('Tiles (averages across Oki, Osa, Bar): operons by genomic region')
```

```
p9 <- ggboxplot(tb, x='seqnames_arm_category', y='operons.length.mean', color='seqnames_arm', add='jitter') + ylab('operon length') + theme_classic() + theme(axis.text.x=element_text(angle=90))
p9 + ggtitle('Tiles (averages across Oki, Osa, Bar): operon lengths by genomic region')
```

```
p10 <- ggboxplot(tb, x='chr.arm.label', y='operons.length.mean', color='chr.arm.label', add='jitter') + ylab('operon length') + theme_classic() + theme(axis.text.x=element_text(angle=90)) + stat_compare_means(comparison=list(c('pericentromeric', 'centre'), c('pericentromeric', 'subtelomeric'), c('centre', 'subtelomeric')), label='p.signif', paired = FALSE, na.rm = TRUE, method=compare_method)
p10 + ggtitle('Tiles (averages across Oki, Osa, Bar): operon lengths by genomic region')
```

```
# Breakpoints
p11 <- ggboxplot(tb, x='seqnames_arm_category', y='breakpoints.count.mean', color='seqnames_arm', add='jitter') + ylab('breakpoints') + theme_classic() + theme(axis.text.x=element_text(angle=90))
p11 + ggtitle('Tiles (averages across Oki, Osa, Bar): breakpoints by genomic region')
```

```
p12 <- ggboxplot(tb, x='chr.arm.label', y='breakpoints.count.mean', color='chr.arm.label', add='jitter') + ylab('breakpoints') + theme_classic() + theme(axis.text.x=element_text(angle=90)) + stat_compare_means(comparison=list(c('pericentromeric', 'centre'), c('pericentromeric', 'subtelomeric'), c('centre', 'subtelomeric')), label='p.signif', paired = FALSE, na.rm = TRUE, method=compare_method)
p12 + ggtitle('Tiles (averages across Oki, Osa, Bar): breakpoints by genomic region')
```

```
# dN/dS
p13 <- ggboxplot(tb, x='seqnames_arm_category', y='transcripts.dNdS.mean', color='seqnames_arm', add='jitter') + ylab('dN/dS') + theme_classic() + theme(axis.text.x=element_text(angle=90))
p13 + ggtitle('Tiles (averages across Oki, Osa, Bar): dN/dS by genomic region')
```

```
p14 <- ggboxplot(tb, x='chr.arm.label', y='transcripts.dNdS.mean', color='chr.arm.label', add='jitter') + ylab('dN/dS') + theme_classic() + theme(axis.text.x=element_text(angle=90)) + stat_compare_means(comparison=list(c('pericentromeric', 'centre'), c('pericentromeric', 'subtelomeric'), c('centre', 'subtelomeric')), label='p.signif', paired = FALSE, na.rm = TRUE, method=compare_method)
p14 + ggtitle('Tiles (averages across Oki, Osa, Bar): dN/dS by genomic region')
```

```
detach('package:ggpubr')
```

### Checking my work

Double-checking breakpoint patterns to make sure they make sense and
match the averages.

```
p1 <- chrt_unified |> as.data.frame() |> subset(select=c("seqnames", "start", "end", "Oki.bp.Oki_Osa.breaks.count", "Oki.bp.Oki_Osa.breaks.count", "Oki.bp.Oki_Bar.breaks.count", "Oki.bp.Oki_Kum.breaks.count", "Oki.bp.Oki_Aom.breaks.count", "Oki.bp.Oki_Nor.breaks.count")) |> tidyr::pivot_longer(c("Oki.bp.Oki_Osa.breaks.count", "Oki.bp.Oki_Osa.breaks.count", "Oki.bp.Oki_Bar.breaks.count", "Oki.bp.Oki_Kum.breaks.count", "Oki.bp.Oki_Aom.breaks.count", "Oki.bp.Oki_Nor.breaks.count")) |> ggplot() + aes(start, value, col=name) + facet_wrap(~seqnames, nrow=1) + geom_smooth(method='loess', formula=y~x, span=0.25, se=FALSE, method.args=list(family="symmetric"))

p2 <- chrt_unified |> as.data.frame() |> subset(select=c("seqnames", "start", "end", "Osa.bp.Osa_Oki.breaks.count", "Osa.bp.Osa_Bar.breaks.count", "Osa.bp.Osa_Aom.breaks.count", "Osa.bp.Osa_Kum.breaks.count", "Osa.bp.Osa_Nor.breaks.count")) |> tidyr::pivot_longer(c("Osa.bp.Osa_Oki.breaks.count", "Osa.bp.Osa_Bar.breaks.count", "Osa.bp.Osa_Aom.breaks.count", "Osa.bp.Osa_Kum.breaks.count", "Osa.bp.Osa_Nor.breaks.count")) |> ggplot() + aes(start, value, col=name) + facet_wrap(~seqnames, nrow=1) + geom_smooth(method='loess', formula=y~x, span=0.25, se=FALSE, method.args=list(family="symmetric"))

p3 <- chrt_unified |> as.data.frame() |> subset(select=c("seqnames", "start", "end", "Bar.bp.Bar_Oki.breaks.count", "Bar.bp.Bar_Osa.breaks.count", "Bar.bp.Bar_Aom.breaks.count", "Bar.bp.Bar_Kum.breaks.count", "Bar.bp.Bar_Nor.breaks.count")) |> tidyr::pivot_longer(c("Bar.bp.Bar_Oki.breaks.count", "Bar.bp.Bar_Osa.breaks.count", "Bar.bp.Bar_Aom.breaks.count", "Bar.bp.Bar_Kum.breaks.count", "Bar.bp.Bar_Nor.breaks.count")) |> ggplot() + aes(start, value, col=name) + facet_wrap(~seqnames, nrow=1) + geom_smooth(method='loess', formula=y~x, span=0.25, se=FALSE, method.args=list(family="symmetric"))

(p1 / p2 / p3)
```

Same with dN/dS.

```
p1 <- chrt_unified |> as.data.frame() |> ggplot() + aes(start, Oki.transcripts.dNdS_GUIDANCE2.median, col=Oki.Arm) + facet_wrap(~seqnames, nrow=1) + geom_point() + geom_smooth(method='loess', formula=y~x, span=0.25, se=T, method.args=list(family="symmetric"))
p2 <- chrt_unified |> as.data.frame() |> ggplot() + aes(start, Osa.transcripts.dNdS_GUIDANCE2.median, col=Osa.Arm) + facet_wrap(~seqnames, nrow=1) + geom_point() + geom_smooth(method='loess', formula=y~x, span=0.25, se=T, method.args=list(family="symmetric"))
p3 <- chrt_unified |> as.data.frame() |> ggplot() + aes(start, Bar.transcripts.dNdS_GUIDANCE2.median, col=Bar.Arm) + facet_wrap(~seqnames, nrow=1) + geom_point() + geom_smooth(method='loess', formula=y~x, span=0.25, se=T, method.args=list(family="symmetric"))
(p1 / p2 / p3)
```

Double-check the smoothing.

```
p1 <- chrt_unified |> as.data.frame() |> subset(select=c("seqnames", "start", "end", "Oki.bp.Oki_Osa.breaks.count", "Oki.bp.Oki_Osa.breaks.count", "Oki.bp.Oki_Bar.breaks.count", "Oki.bp.Oki_Kum.breaks.count", "Oki.bp.Oki_Aom.breaks.count", "Oki.bp.Oki_Nor.breaks.count")) |> tidyr::pivot_longer(c("Oki.bp.Oki_Osa.breaks.count", "Oki.bp.Oki_Osa.breaks.count", "Oki.bp.Oki_Bar.breaks.count", "Oki.bp.Oki_Kum.breaks.count", "Oki.bp.Oki_Aom.breaks.count", "Oki.bp.Oki_Nor.breaks.count")) |> ggplot() + aes(start, value, col=name) + facet_wrap(~seqnames, nrow=1) + geom_smooth(method='loess', formula=y~x, span=0.25, se=FALSE)

p2 <- chrt_unified |> as.data.frame() |> subset(select=c("seqnames", "start", "end", "Osa.bp.Osa_Oki.breaks.count", "Osa.bp.Osa_Bar.breaks.count", "Osa.bp.Osa_Aom.breaks.count", "Osa.bp.Osa_Kum.breaks.count", "Osa.bp.Osa_Nor.breaks.count")) |> tidyr::pivot_longer(c("Osa.bp.Osa_Oki.breaks.count", "Osa.bp.Osa_Bar.breaks.count", "Osa.bp.Osa_Aom.breaks.count", "Osa.bp.Osa_Kum.breaks.count", "Osa.bp.Osa_Nor.breaks.count")) |> ggplot() + aes(start, value, col=name) + facet_wrap(~seqnames, nrow=1) + geom_smooth(method='loess', formula=y~x, span=0.25, se=FALSE)

p3 <- chrt_unified |> as.data.frame() |> subset(select=c("seqnames", "start", "end", "Bar.bp.Bar_Oki.breaks.count", "Bar.bp.Bar_Osa.breaks.count", "Bar.bp.Bar_Aom.breaks.count", "Bar.bp.Bar_Kum.breaks.count", "Bar.bp.Bar_Nor.breaks.count")) |> tidyr::pivot_longer(c("Bar.bp.Bar_Oki.breaks.count", "Bar.bp.Bar_Osa.breaks.count", "Bar.bp.Bar_Aom.breaks.count", "Bar.bp.Bar_Kum.breaks.count", "Bar.bp.Bar_Nor.breaks.count")) |> ggplot() + aes(start, value, col=name) + facet_wrap(~seqnames, nrow=1) + geom_smooth(method='loess', formula=y~x, span=0.25, se=FALSE)

(p1 / p2 / p3)
```

```
p1 <- chrt_unified |> as.data.frame() |> ggplot() + aes(start, Oki.transcripts.dNdS_GUIDANCE2.median, col=Oki.Arm) + facet_wrap(~seqnames, nrow=1) + geom_point() + geom_smooth(method='loess', formula=y~x, span=0.25, se=T)

p2 <- chrt_unified |> as.data.frame() |> ggplot() + aes(start, Osa.transcripts.dNdS_GUIDANCE2.median, col=Osa.Arm) + facet_wrap(~seqnames, nrow=1) + geom_point() + geom_smooth(method='loess', formula=y~x, span=0.25, se=T)

p3 <- chrt_unified |> as.data.frame() |> ggplot() + aes(start, Bar.transcripts.dNdS_GUIDANCE2.median, col=Bar.Arm) + facet_wrap(~seqnames, nrow=1) + geom_point() + geom_smooth(method='loess', formula=y~x, span=0.25, se=T)

(p1 / p2 / p3)
```

The same figure as above, but without symmetric smoothing.

```
p1 <- ggplot( chrt_unified |> as.data.frame() ) + aes(x=start, y=transcripts.dNdS.mean, col = Oki.Arm) + geom_point() + facet_wrap(~seqnames, nrow = 1) + geom_smooth(formula=y~x, method = 'loess') + ylab('dN/dS')

p2 <- ggplot( chrt_unified |> as.data.frame() ) + aes(x=start, y=breakpoints.count.mean , col = Oki.Arm) + geom_point() + facet_wrap(~seqnames, nrow = 1) + geom_smooth(formula=y~x, method = 'loess') + ylab('breakpoints')

library(ggpubr)
tb <- chrt_unified |> as.data.frame()
tb$seqnames_arm <- paste0(tb$seqnames, "_", tb$Oki.Arm)

p3 <- ggboxplot(tb, x='seqnames_arm', y='breakpoints.count.mean', color='Oki.Arm', add='jitter') +
  theme(axis.text.x=element_text(angle=90)) +
  ylab('breakpoints') +
  stat_compare_means(comparison=list(c('Chr1_short', 'Chr1_long'), c('Chr2_short', 'Chr2_long'), c('PAR_short', 'PAR_long'), c('XSR_XSR', 'PAR_short'), c('XSR_XSR', 'PAR_long')), paired = FALSE, na.rm = TRUE, method='t.test') + theme_classic()

p4 <- ggboxplot(tb, x='seqnames_arm', y='transcripts.dNdS.mean', color='Oki.Arm', add='jitter') +
  theme(axis.text.x=element_text(angle=90)) +
  ylab('dN/dS') +
  stat_compare_means(comparison=list(c('Chr1_short', 'Chr1_long'), c('Chr2_short', 'Chr2_long'), c('PAR_short', 'PAR_long'), c('XSR_XSR', 'PAR_short'), c('XSR_XSR', 'PAR_long')), paired = FALSE, na.rm = TRUE, method='t.test') + theme_classic()

p5 <- ggplot(tb) + aes(x=breakpoints.count.mean, y=transcripts.dNdS.mean) + geom_point() + geom_smooth(method='lm', col='red', formula=y~x) + ggpubr::stat_cor() + facet_wrap(seqnames~Oki.Arm, nrow=1, scales = 'free') + ylab('dN/dS')

layout <- "
A
B
C
D
E
"

( p1 / p4 / p2 / p3 / p5 ) + plot_layout(design = layout, guides = 'collect')
```

```
detach('package:ggpubr')
```

Finally, double check the look of the figure with different pairs of
species.

Start with Okinawa-Osaka.

```
p1 <- ggplot( chrt_unified |> as.data.frame() ) + aes(x=start, y=Oki.transcripts.dNdS_GUIDANCE2.median, col = Oki.Arm) + geom_point() + facet_wrap(~seqnames, nrow = 1) + geom_smooth(formula=y~x, method = 'loess') + ylab('dN/dS')

p2 <- ggplot( chrt_unified |> as.data.frame() ) + aes(x=start, y=Oki.bp.Oki_Osa.breaks.count , col = Oki.Arm) + geom_point() + facet_wrap(~seqnames, nrow = 1) + geom_smooth(formula=y~x, method = 'loess') + ylab('breakpoints')

library(ggpubr)
tb <- chrt_unified |> as.data.frame()
tb$seqnames_arm <- paste0(tb$seqnames, "_", tb$Oki.Arm)

p3 <- ggboxplot(tb, x='seqnames_arm', y='Oki.bp.Oki_Osa.breaks.count', color='Oki.Arm', add='jitter') +
  theme(axis.text.x=element_text(angle=90)) +
  ylab('breakpoints') +
  stat_compare_means(comparison=list(c('Chr1_short', 'Chr1_long'), c('Chr2_short', 'Chr2_long'), c('PAR_short', 'PAR_long'), c('XSR_XSR', 'PAR_short'), c('XSR_XSR', 'PAR_long')), paired = FALSE, na.rm = TRUE, method='t.test') + theme_classic()

p4 <- ggboxplot(tb, x='seqnames_arm', y='Oki.transcripts.dNdS_GUIDANCE2.median', color='Oki.Arm', add='jitter') +
  theme(axis.text.x=element_text(angle=90)) +
  ylab('dN/dS') +
  stat_compare_means(comparison=list(c('Chr1_short', 'Chr1_long'), c('Chr2_short', 'Chr2_long'), c('PAR_short', 'PAR_long'), c('XSR_XSR', 'PAR_short'), c('XSR_XSR', 'PAR_long')), paired = FALSE, na.rm = TRUE, method='t.test') + theme_classic()

p5 <- ggplot(tb) + aes(x=Oki.bp.Oki_Osa.breaks.count, y=Oki.transcripts.dNdS_GUIDANCE2.median) + geom_point() + geom_smooth(method='lm', col='red', formula=y~x) + ggpubr::stat_cor() + facet_wrap(seqnames~Oki.Arm, nrow=1, scales = 'free') + ylab('dN/dS')

layout <- "
A
B
C
D
E
"

( p1 / p4 / p2 / p3 / p5 ) + plot_layout(design = layout, guides = 'collect')
```

#### Then Okinawa-Barcelona…

```
p1 <- ggplot( chrt_unified |> as.data.frame() ) + aes(x=start, y=Oki.transcripts.dNdS_GUIDANCE2.median, col = Oki.Arm) + geom_point() + facet_wrap(~seqnames, nrow = 1) + geom_smooth(formula=y~x, method = 'loess') + ylab('dN/dS')

p2 <- ggplot( chrt_unified |> as.data.frame() ) + aes(x=start, y=Oki.bp.Oki_Bar.breaks.count , col = Oki.Arm) + geom_point() + facet_wrap(~seqnames, nrow = 1) + geom_smooth(formula=y~x, method = 'loess') + ylab('breakpoints')

tb <- chrt_unified |> as.data.frame()
tb$seqnames_arm <- paste0(tb$seqnames, "_", tb$Oki.Arm)
tb$seqnames_arm <- paste0(tb$seqnames, "_", tb$Oki.Arm)
tb$Arm <- tb$Oki.Arm

customGGboxplot <- function(tb, y, ylab) {
  ggboxplot(tb, x='seqnames_arm', y=y, color='Arm', add='jitter') +
    theme(axis.text.x=element_text(angle=90)) +
    ylab(ylab) +
    stat_compare_means(comparison=list(c('Chr1_short', 'Chr1_long'), c('Chr2_short', 'Chr2_long'),
                                       c('PAR_short', 'PAR_long'), c('XSR_XSR', 'PAR_short'),
                                       c('XSR_XSR', 'PAR_long')), paired = FALSE, na.rm = TRUE, method='t.test') +
    theme_classic()
}

customCorrelationPlot <- function(tb, x, y, xlab, ylab)
  ggplot(tb) +
    aes(x=.data[[x]], y=.data[[y]]) +
    geom_point() +
    geom_smooth(method='lm', col='red', formula=y~x) +
    ggpubr::stat_cor() +
    facet_wrap(seqnames~Arm, nrow=1, scales = 'free') + ylab(ylab) +
    theme_bw()

p3 <- tb |> customGGboxplot('Oki.bp.Oki_Bar.breaks.count', 'breakpoints')
p4 <- tb |> customGGboxplot('Oki.transcripts.dNdS_GUIDANCE2.median', 'dN/dS')

p5 <- tb |> customCorrelationPlot('Oki.bp.Oki_Bar.breaks.count', 'Oki.transcripts.dNdS_GUIDANCE2.median',  'breakpoints', 'dN/dS')

( p1 / p4 / p2 / p3 / p5 )
```

#### Then Osaka-Barcelona…

```
tb <- chrt_unified |> as.data.frame()
tb$seqnames_arm <- paste0(tb$seqnames, "_", tb$Osa.Arm)
tb$Arm <- tb$Osa.Arm

p1 <- tb |> per_chrom_plot() + aes(y=Osa.transcripts.dNdS_GUIDANCE2.median, col = Arm) + ylab('dN/dS')
p2 <- tb |> per_chrom_plot() + aes(y=Osa.bp.Osa_Bar.breaks.count, col = Arm)           + ylab('breakpoints')

p3 <- tb |> customGGboxplot('Osa.bp.Osa_Bar.breaks.count', 'breakpoints')
p4 <- tb |> customGGboxplot('Osa.transcripts.dNdS_GUIDANCE2.median', 'dN/dS')

p5 <- tb |> customCorrelationPlot('Osa.bp.Osa_Bar.breaks.count', 'Osa.transcripts.dNdS_GUIDANCE2.median',  'breakpoints', 'dN/dS')

( p1 / p4 / p2 / p3 / p5 )
```

#### And finally Okinawa-Kume.

```
tb$seqnames_arm <- paste0(tb$seqnames, "_", tb$Oki.Arm)
tb$Arm <- tb$Oki.Arm

p1 <- tb |> per_chrom_plot() + aes(y=Oki.transcripts.dNdS_GUIDANCE2.median, col = Arm) + ylab('dN/dS')
p2 <- tb |> per_chrom_plot() + aes(y=Oki.bp.Oki_Kum.breaks.count, col = Arm)           + ylab('breakpoints')

p3 <- tb |> customGGboxplot('Oki.bp.Oki_Kum.breaks.count', 'breakpoints')
p4 <- tb |> customGGboxplot('Oki.transcripts.dNdS_GUIDANCE2.median', 'dN/dS')

p5 <- tb |> customCorrelationPlot('Oki.bp.Oki_Kum.breaks.count', 'Oki.transcripts.dNdS_GUIDANCE2.median',  'breakpoints', 'dN/dS')

( p1 / p4 / p2 / p3 / p5 )
```

### Classes of repeats

We can use the `[mcol].counts.tables` metadata columns to
calculate statistics between different genomic regions.

#### Repeat-related chromosome plots, windows: Okinawa

```
(plots$Oki$repeats$classes <- chrw$Oki |>
  as.data.frame() |>
  subset(select=c("seqnames", "start", "end", "repeats.Class.count.total", "repeats.Class.LowComplexity.count.total", "repeats.Class.ltr.1.count.total", "repeats.Class.rnd.count.total", "repeats.Class.SINE.count.total", "repeats.Class.tandem.count.total", "repeats.Class.unknown.count.total" )) |>
  plyranges::filter(seqnames %in% c('chr1', 'chr2', 'PAR', 'XSR')) |>
  tidyr::pivot_longer(c("repeats.Class.count.total", "repeats.Class.LowComplexity.count.total", "repeats.Class.ltr.1.count.total", "repeats.Class.rnd.count.total", "repeats.Class.SINE.count.total", "repeats.Class.tandem.count.total", "repeats.Class.unknown.count.total")) |>
  ggplot() +
    aes(start, value, col = name) +
    facet_wrap(~seqnames, nrow = 1) +
    geom_smooth(method='loess', formula = y ~ x, span = 0.25, se = FALSE) +
    ggtitle("Okinawa: Density of repeat classes across genome"))
```

#### Repeat-related chromosome plots, windows: Osaka

```
(plots$Osa$repeats$classes <- chrw$Osa |>
  as.data.frame() |>
  subset(select=c("seqnames", "start", "end", "repeats.Class.count.total", "repeats.Class.LowComplexity.count.total", "repeats.Class.rnd.count.total", "repeats.Class.tandem.count.total", "repeats.Class.unknown.count.total" )) |>
  plyranges::filter(seqnames %in% c('Chr1', 'Chr2', 'PAR', 'XSR')) |>
  tidyr::pivot_longer(c("repeats.Class.count.total", "repeats.Class.LowComplexity.count.total", "repeats.Class.rnd.count.total", "repeats.Class.tandem.count.total", "repeats.Class.unknown.count.total" )) |>
  ggplot() +
    aes(start, value, col = name) +
    facet_wrap(~seqnames, nrow = 1) +
    geom_smooth(method='loess', formula = y ~ x, span = 0.25, se = FALSE) +
    ggtitle("Osaka: distribution of repeat classes across genome"))
```

#### Repeat-related chromosome plots, windows: Barcelona

```
(plots$Bar$repeats$classes <- chrw$Bar |>
  as.data.frame() |>
  subset(select=c("seqnames", "start", "end", "repeats.Class.count.total", "repeats.Class.LowComplexity.count.total", "repeats.Class.rnd.count.total", "repeats.Class.tandem.count.total", "repeats.Class.unknown.count.total" )) |>
  plyranges::filter(seqnames %in% c('Chr1', 'Chr2', 'PAR', 'XSR')) |>
  tidyr::pivot_longer(c("repeats.Class.count.total", "repeats.Class.LowComplexity.count.total", "repeats.Class.rnd.count.total", "repeats.Class.tandem.count.total", "repeats.Class.unknown.count.total" )) |>
  ggplot() +
    aes(start, value, col = name) +
    facet_wrap(~seqnames, nrow = 1) +
    geom_smooth(method='loess', formula = y ~ x, span = 0.25, se = FALSE) +
    ggtitle("Barcelona: distribution of repeat classes across genome"))
```

### Scaled alignment scores

In the following section I divide the scores by the widths of the
alignments, creating a scaled score.

```
chrw$Oki$Oki_Osa.score.norm.mean <- binApply(mean, na.rm = T, bins_Oki, score(gbs$Oki_Osa)/lengths(gbs$Oki_Osa), matchToOneBin(gbs$Oki_Osa, bins_Oki))
chrw$Oki$Oki_Aom.score.norm.mean <- binApply(mean, na.rm = T, bins_Oki, score(gbs$Oki_Aom)/lengths(gbs$Oki_Aom), matchToOneBin(gbs$Oki_Aom, bins_Oki))
chrw$Oki$Oki_Bar.score.norm.mean <- binApply(mean, na.rm = T, bins_Oki, score(gbs$Oki_Bar)/lengths(gbs$Oki_Bar), matchToOneBin(gbs$Oki_Bar, bins_Oki))
chrw$Oki$Oki_Nor.score.norm.mean <- binApply(mean, na.rm = T, bins_Oki, score(gbs$Oki_Nor)/lengths(gbs$Oki_Nor), matchToOneBin(gbs$Oki_Nor, bins_Oki))

ggplot(as.data.frame(chrw$Oki) |> plyranges::filter(seqnames %in% c('chr1', 'chr2', 'PAR', 'XSR', 'YSR')) ) +
  aes(x=start, y=Oki_Osa.score.norm.mean) +
  geom_point() +
  facet_wrap(~seqnames) +
  geom_smooth(method='loess', formula = y ~ x) +
  scale_y_log10() +
  ggtitle("Oki-Osa: Chromosomal position vs. scaled alignment score")
```

```
ggplot(as.data.frame(chrw$Oki) |> plyranges::filter(seqnames %in% c('chr1', 'chr2', 'PAR', 'XSR', 'YSR')) ) +
  aes(x=start, y=Oki_Bar.score.norm.mean) +
  geom_point() +
  facet_wrap(~seqnames) +
  geom_smooth(method='loess', formula = y ~ x) +
  scale_y_log10() +
  ggtitle("Oki-Bar: Chromosomal position vs. scaled alignment score")
```

```
chrw$Oki |>
  as.data.frame() |>
  subset(select=c("seqnames", "start", "end", "Oki_Osa.score.norm.mean", "Oki_Aom.score.norm.mean", "Oki_Bar.score.norm.mean", "Oki_Nor.score.norm.mean" )) |>
  plyranges::filter(seqnames %in% c('chr1', 'chr2', 'PAR', 'XSR')) |>
  tidyr::pivot_longer(c("Oki_Osa.score.norm.mean", "Oki_Aom.score.norm.mean", "Oki_Bar.score.norm.mean", "Oki_Nor.score.norm.mean")) |>
  ggplot() +
    aes(start, value, col = name) +
    facet_wrap(~seqnames, nrow = 1) +
    geom_smooth(method='loess', formula = y ~ x, span = 0.25, se = FALSE) +
    ggtitle("Okinawa: Chromosomal position vs. scaled alignment score")
```

### Alignment categories

The 4 alignment categories we describe can vary between genomic
compartments. In this section, I analyse their distributions.

```
# Okinawa
mcols(chrw$Oki) <- cbind(mcols(chrw$Oki), mcols(grWindows(genome=genomes$Oki, meta=wgo$Oki_Osa, windowSize=windowSize, meta_prefix = 'ac.Oki_Osa.')))
mcols(chrw$Oki) <- cbind(mcols(chrw$Oki), mcols(grWindows(genome=genomes$Oki, meta=wgo$Oki_Bar, windowSize=windowSize, meta_prefix = 'ac.Oki_Bar.')))
mcols(chrw$Oki) <- cbind(mcols(chrw$Oki), mcols(grWindows(genome=genomes$Oki, meta=wgo$Oki_Kum, windowSize=windowSize, meta_prefix = 'ac.Oki_Kum.')))
mcols(chrw$Oki) <- cbind(mcols(chrw$Oki), mcols(grWindows(genome=genomes$Oki, meta=wgo$Oki_Aom, windowSize=windowSize, meta_prefix = 'ac.Oki_Aom.')))
mcols(chrw$Oki) <- cbind(mcols(chrw$Oki), mcols(grWindows(genome=genomes$Oki, meta=wgo$Oki_Nor, windowSize=windowSize, meta_prefix = 'ac.Oki_Nor.')))

chrw$Oki$ac.isolated.mean <- chrw$Oki |> as.data.frame()|> select("ac.Oki_Osa.type.isolated.alignment.count.total", "ac.Oki_Bar.type.isolated.alignment.count.total", "ac.Oki_Kum.type.isolated.alignment.count.total", "ac.Oki_Aom.type.isolated.alignment.count.total", "ac.Oki_Nor.type.isolated.alignment.count.total") |> as.data.frame() |> rowMeans()
chrw$Oki$ac.collinear.mean <- chrw$Oki |> as.data.frame()|> select("ac.Oki_Osa.type.collinear.alignment.count.total", "ac.Oki_Bar.type.collinear.alignment.count.total", "ac.Oki_Kum.type.collinear.alignment.count.total", "ac.Oki_Aom.type.collinear.alignment.count.total", "ac.Oki_Nor.type.collinear.alignment.count.total") |> as.data.frame() |> rowMeans()
chrw$Oki$ac.breakpoint.mean <- chrw$Oki |> as.data.frame() |> select("ac.Oki_Osa.type.breakpoint.region.count.total", "ac.Oki_Bar.type.breakpoint.region.count.total", "ac.Oki_Kum.type.breakpoint.region.count.total", "ac.Oki_Aom.type.breakpoint.region.count.total", "ac.Oki_Nor.type.breakpoint.region.count.total") |> as.data.frame() |> rowMeans()
chrw$Oki$ac.bridge.mean <- chrw$Oki |> as.data.frame()|> select("ac.Oki_Osa.type.bridge.region.count.total", "ac.Oki_Bar.type.bridge.region.count.total", "ac.Oki_Kum.type.bridge.region.count.total", "ac.Oki_Aom.type.bridge.region.count.total", "ac.Oki_Nor.type.bridge.region.count.total") |> as.data.frame() |> rowMeans()

plots$Oki$categories <- list()

plots$Oki$categories$oki_osa_classes <- chrw$Oki |>
  as.data.frame() |>
  subset(select=c("seqnames", "start", "end", "ac.Oki_Osa.type.isolated.alignment.count.total", "ac.Oki_Osa.type.collinear.alignment.count.total", "ac.Oki_Osa.type.breakpoint.region.count.total", "ac.Oki_Osa.type.bridge.region.count.total" )) |>
  plyranges::filter(seqnames %in% c('chr1', 'chr2', 'PAR', 'XSR')) |>
  tidyr::pivot_longer(c("ac.Oki_Osa.type.isolated.alignment.count.total", "ac.Oki_Osa.type.collinear.alignment.count.total", "ac.Oki_Osa.type.breakpoint.region.count.total", "ac.Oki_Osa.type.bridge.region.count.total")) |>
  ggplot() +
    aes(start, value, col = name) +
    facet_wrap(~seqnames, nrow = 1) +
    geom_smooth(method='loess', formula = y ~ x, span = 0.25, se = FALSE) +
    ggtitle("Okinawa-Osaka: Distribution of alignment categories across genome")

plots$Oki$categories$oki_osa_overall <- chrw$Oki |>
  as.data.frame() |>
  subset(select=c("seqnames", "start", "end", "Arm", "ac.Oki_Osa.type.isolated.alignment.count.total", "ac.Oki_Osa.type.collinear.alignment.count.total", "ac.Oki_Osa.type.breakpoint.region.count.total", "ac.Oki_Osa.type.bridge.region.count.total" )) |>
  plyranges::filter(seqnames %in% c('chr1', 'chr2', 'PAR', 'XSR')) |>
 tidyr::pivot_longer(c("ac.Oki_Osa.type.isolated.alignment.count.total", "ac.Oki_Osa.type.collinear.alignment.count.total", "ac.Oki_Osa.type.breakpoint.region.count.total", "ac.Oki_Osa.type.bridge.region.count.total")) |>
  ggplot() +
    aes(value, paste0(seqnames, "_", Arm),  fill = name) +
    geom_bar(position='fill', stat='identity') +
    ylab("chr and arm") +
    ggtitle("Okinawa-Osaka: Overall proportion of alignment categories across genome")

plots$Oki$categories$oki_osa_by_chr_position <- chrw$Oki |>
  as.data.frame() |>
  subset(select=c("seqnames", "start", "end", "Arm", "ac.Oki_Osa.type.isolated.alignment.count.total", "ac.Oki_Osa.type.collinear.alignment.count.total", "ac.Oki_Osa.type.breakpoint.region.count.total", "ac.Oki_Osa.type.bridge.region.count.total" )) |>
  plyranges::filter(seqnames %in% c('chr1', 'chr2', 'PAR', 'XSR')) |>
 tidyr::pivot_longer(c("ac.Oki_Osa.type.isolated.alignment.count.total", "ac.Oki_Osa.type.collinear.alignment.count.total", "ac.Oki_Osa.type.breakpoint.region.count.total", "ac.Oki_Osa.type.bridge.region.count.total")) |>
  ggplot() +
    aes(as.factor(start), value, fill = name) +
    facet_wrap(~seqnames, nrow = 1, scales='free') +
    theme(axis.text.x=element_blank(), axis.ticks.x=element_blank()) +
    geom_bar(position='fill', stat='identity') +
    xlab("start") +
    ggtitle("Okinawa-Osaka: Proportion of alignment categories by position")

# Next, plot the means of ALL populations vs. Oki.
plots$Oki$categories$mean_classes <- chrw$Oki |>
  as.data.frame() |>
  subset(select=c("seqnames", "start", "end", "Arm", "ac.isolated.mean", "ac.collinear.mean", "ac.breakpoint.mean", "ac.bridge.mean" )) |>
  plyranges::filter(seqnames %in% c('chr1', 'chr2', 'PAR', 'XSR')) |>
 tidyr::pivot_longer(c("ac.isolated.mean", "ac.collinear.mean", "ac.breakpoint.mean", "ac.bridge.mean")) |>
  ggplot() +
    aes(start, value, col = name) +
    facet_wrap(~seqnames, nrow = 1) +
    geom_smooth(method='loess', formula = y ~ x, span = 0.25, se = FALSE) +
    ggtitle("Okinawa vs. all: Distribution of alignment categories across genome")

plots$Oki$categories$mean_classes_overall <- chrw$Oki |>
  as.data.frame() |>
  subset(select=c("seqnames", "start", "end", "Arm", "ac.isolated.mean", "ac.collinear.mean", "ac.breakpoint.mean", "ac.bridge.mean" )) |>
  plyranges::filter(seqnames %in% c('chr1', 'chr2', 'PAR', 'XSR')) |>
 tidyr::pivot_longer(c("ac.isolated.mean", "ac.collinear.mean", "ac.breakpoint.mean", "ac.bridge.mean")) |>
  ggplot() +
    aes(value, paste0(seqnames, "_", Arm),  fill = name) +
    geom_bar(position='fill', stat='identity') +
    ylab("chr and arm") +
    ggtitle("Okinawa vs. all: Overall proportion of alignment categories across genome")

plots$Oki$categories$mean_classes_by_chr_position <- chrw$Oki |>
  as.data.frame() |>
  subset(select=c("seqnames", "start", "end", "Arm", "ac.isolated.mean", "ac.collinear.mean", "ac.breakpoint.mean", "ac.bridge.mean" )) |>
  plyranges::filter(seqnames %in% c('chr1', 'chr2', 'PAR', 'XSR')) |>
 tidyr::pivot_longer(c("ac.isolated.mean", "ac.collinear.mean", "ac.breakpoint.mean", "ac.bridge.mean")) |>
  ggplot() +
    aes(as.factor(start), value, fill = name) +
    facet_wrap(~seqnames, nrow = 1, scales='free') +
    theme(axis.text.x=element_blank(), axis.ticks.x=element_blank()) +
    geom_bar(position='fill', stat='identity') +
    xlab("start") +
    ggtitle("Okinawa vs. all: Proportion of alignment categories by position")

lapply(plots$Oki$categories, \(x) x)
```

```
## $oki_osa_classes
```

```
## 
## $oki_osa_overall
```

```
## 
## $oki_osa_by_chr_position
```

```
## 
## $mean_classes
```

```
## 
## $mean_classes_overall
```

```
## 
## $mean_classes_by_chr_position
```

### Extra Okinawa Oik plots for a presentation

```
(
  (chrw$Oki |>
     per_chrom_plot(Y=TRUE) + aes(y=transcripts.tx_name.count) +
     ggtitle(paste("Okinawa: transcripts per window (size = ", windowSize/1000, " kbp)", sep=""))) / # plots$Oki$transcripts$density with YSR
  (chrw$Oki |>
     per_chrom_plot(Y=TRUE) + aes(y=operons.n.count) +
     ggtitle(paste("Okinawa: operons per window (size = ", windowSize/1000, " kbp)", sep=""))) / # plots$Oki$operons$density with YSR
  (chrw$Oki |>
    per_chrom_plot(Y=TRUE) + aes(y=operons.n.mean)  +
    ggtitle(paste("Okinawa: mean operon size per window (size = ", windowSize/1000, " kbp)", sep=""))) / # Ditto
  plots$Oki$repeats$density /
  plots$Oki$scores$oki_osa /
  plots$Oki$widths$widths
)  + plot_layout(guides = 'collect')
```

## Session information

```
sessionInfo()
```

```
## R version 4.3.1 (2023-06-16)
## Platform: x86_64-pc-linux-gnu (64-bit)
## Running under: Debian GNU/Linux 12 (bookworm)
## 
## Matrix products: default
## BLAS:   /usr/lib/x86_64-linux-gnu/blas/libblas.so.3.11.0 
## LAPACK: /usr/lib/x86_64-linux-gnu/lapack/liblapack.so.3.11.0
## 
## locale:
##  [1] LC_CTYPE=C.UTF-8       LC_NUMERIC=C           LC_TIME=C.UTF-8       
##  [4] LC_COLLATE=C.UTF-8     LC_MONETARY=C.UTF-8    LC_MESSAGES=C.UTF-8   
##  [7] LC_PAPER=C.UTF-8       LC_NAME=C              LC_ADDRESS=C          
## [10] LC_TELEPHONE=C         LC_MEASUREMENT=C.UTF-8 LC_IDENTIFICATION=C   
## 
## time zone: Etc/UTC
## tzcode source: system (glibc)
## 
## attached base packages:
## [1] stats4    stats     graphics  grDevices utils     datasets  methods  
## [8] base     
## 
## other attached packages:
##  [1] ggpubr_0.6.0                           
##  [2] BSgenome.Oidioi.genoscope.OdB3_1.0.0   
##  [3] BSgenome.Oidioi.OIST.AOM.5.5f_1.0.1    
##  [4] BSgenome.Oidioi.OIST.KUM.M3.7f_1.0.1   
##  [5] BSgenome.Oidioi.OIST.Bar2.p4_1.0.1     
##  [6] BSgenome.Oidioi.OIST.OSKA2016v1.9_1.0.0
##  [7] BSgenome.Oidioi.OIST.OKI2018.I69_1.0.1 
##  [8] patchwork_1.1.2                        
##  [9] plyranges_1.20.0                       
## [10] dplyr_1.1.3                            
## [11] viridis_0.6.4                          
## [12] viridisLite_0.4.2                      
## [13] GenomicFeatures_1.52.1                 
## [14] AnnotationDbi_1.62.2                   
## [15] Biobase_2.60.0                         
## [16] OikScrambling_5.0.0                    
## [17] ggplot2_3.4.3                          
## [18] GenomicBreaks_0.14.2                   
## [19] BSgenome_1.68.0                        
## [20] rtracklayer_1.60.0                     
## [21] Biostrings_2.68.1                      
## [22] XVector_0.40.0                         
## [23] GenomicRanges_1.52.0                   
## [24] GenomeInfoDb_1.36.1                    
## [25] IRanges_2.34.1                         
## [26] S4Vectors_0.38.1                       
## [27] BiocGenerics_0.46.0                    
## 
## loaded via a namespace (and not attached):
##   [1] splines_4.3.1               BiocIO_1.10.0              
##   [3] filelock_1.0.2              bitops_1.0-7               
##   [5] tibble_3.2.1                R.oo_1.25.0                
##   [7] XML_3.99-0.14               rpart_4.1.19               
##   [9] lifecycle_1.0.3             rstatix_0.7.2              
##  [11] rprojroot_2.0.3             lattice_0.20-45            
##  [13] MASS_7.3-58.2               backports_1.4.1            
##  [15] magrittr_2.0.3              Hmisc_5.1-0                
##  [17] sass_0.4.7                  rmarkdown_2.23             
##  [19] jquerylib_0.1.4             yaml_2.3.7                 
##  [21] plotrix_3.8-2               DBI_1.1.3                  
##  [23] CNEr_1.36.0                 minqa_1.2.5                
##  [25] RColorBrewer_1.1-3          ade4_1.7-22                
##  [27] abind_1.4-5                 zlibbioc_1.46.0            
##  [29] purrr_1.0.2                 R.utils_2.12.2             
##  [31] RCurl_1.98-1.12             nnet_7.3-18                
##  [33] pracma_2.4.2                rappdirs_0.3.3             
##  [35] GenomeInfoDbData_1.2.10     gdata_2.19.0               
##  [37] annotate_1.78.0             pkgdown_2.0.7              
##  [39] codetools_0.2-19            DelayedArray_0.26.7        
##  [41] xml2_1.3.5                  tidyselect_1.2.0           
##  [43] shape_1.4.6                 farver_2.1.1               
##  [45] lme4_1.1-34                 BiocFileCache_2.8.0        
##  [47] matrixStats_1.0.0           base64enc_0.1-3            
##  [49] GenomicAlignments_1.36.0    jsonlite_1.8.7             
##  [51] mitml_0.4-5                 Formula_1.2-5              
##  [53] survival_3.5-3              iterators_1.0.14           
##  [55] systemfonts_1.0.5           foreach_1.5.2              
##  [57] progress_1.2.2              tools_4.3.1                
##  [59] ragg_1.2.5                  Rcpp_1.0.11                
##  [61] glue_1.6.2                  gridExtra_2.3              
##  [63] pan_1.8                     mgcv_1.8-41                
##  [65] xfun_0.40                   MatrixGenerics_1.12.2      
##  [67] EBImage_4.42.0              withr_2.5.1                
##  [69] fastmap_1.1.1               boot_1.3-28.1              
##  [71] fansi_1.0.5                 digest_0.6.33              
##  [73] R6_2.5.1                    mice_3.16.0                
##  [75] textshaping_0.3.7           colorspace_2.1-0           
##  [77] GO.db_3.17.0                gtools_3.9.4               
##  [79] poweRlaw_0.70.6             jpeg_0.1-10                
##  [81] biomaRt_2.56.1              RSQLite_2.3.1              
##  [83] weights_1.0.4               R.methodsS3_1.8.2          
##  [85] utf8_1.2.3                  tidyr_1.3.0                
##  [87] generics_0.1.3              data.table_1.14.8          
##  [89] prettyunits_1.2.0           httr_1.4.7                 
##  [91] htmlwidgets_1.6.2           S4Arrays_1.0.5             
##  [93] pkgconfig_2.0.3             gtable_0.3.4               
##  [95] blob_1.2.4                  htmltools_0.5.6.1          
##  [97] carData_3.0-5               fftwtools_0.9-11           
##  [99] scales_1.2.1                png_0.1-8                  
## [101] knitr_1.44                  heatmaps_1.24.0            
## [103] rstudioapi_0.15.0           tzdb_0.4.0                 
## [105] reshape2_1.4.4              rjson_0.2.21               
## [107] curl_5.1.0                  checkmate_2.2.0            
## [109] nlme_3.1-162                nloptr_2.0.3               
## [111] cachem_1.0.8                stringr_1.5.0              
## [113] KernSmooth_2.23-20          parallel_4.3.1             
## [115] genoPlotR_0.8.11            foreign_0.8-84             
## [117] restfulr_0.0.15             desc_1.4.2                 
## [119] pillar_1.9.0                grid_4.3.1                 
## [121] vctrs_0.6.3                 car_3.1-2                  
## [123] jomo_2.7-6                  dbplyr_2.3.3               
## [125] xtable_1.8-4                cluster_2.1.4              
## [127] htmlTable_2.4.1             evaluate_0.22              
## [129] readr_2.1.4                 cli_3.6.1                  
## [131] locfit_1.5-9.8              compiler_4.3.1             
## [133] Rsamtools_2.16.0            rlang_1.1.1                
## [135] crayon_1.5.2                ggsignif_0.6.4             
## [137] labeling_0.4.3              plyr_1.8.8                 
## [139] fs_1.6.3                    stringi_1.7.12             
## [141] BiocParallel_1.34.2         munsell_0.5.0              
## [143] tiff_0.1-11                 glmnet_4.1-7               
## [145] Matrix_1.5-3                hms_1.1.3                  
## [147] bit64_4.0.5                 KEGGREST_1.40.0            
## [149] SummarizedExperiment_1.30.2 broom_1.0.5                
## [151] memoise_2.0.1               bslib_0.5.1                
## [153] bit_4.0.5
```

## Contents

Developed by Aleksandra Bliznina, Michael Mansfield, Charles Plessy.

Site built with pkgdown 2.0.7.
