# Supplementary material for: Extreme genome scrambling in marine planktonic Oikopleura dioica cryptic species
Source: Genome Res. 2024 Mar;34(3):426–40. doi: 10.1101/gr.278295.123 (PMC11067885; doi:10.1101/gr.278295.123)
Supplement: Supplement 4 [file Supplemental_Data.zip › Supplemental_Data.tar.xz!/Supplemental_Data/articles/ScalingBreakpoints.html]

Scaling breakpoints • OikScrambling
 


  


 


Toggle navigation


OikScrambling
5.1.0

- Get started
- Data load
- Supplemental material
  - Figure 1B DNA line plots
  - Figure 1C chromosome parallel plots
  - Figure 1D scrambling index
  - Figure 2A orthogroup dot plots and synteny block histograms
  - Figure 5 genome-wide feature patterns
  - Figure 7A–D DNA line plots
  - Figure 7E breakpoint number barplots
- Articles
  - Scaffolding of the AOM-5 genome
  - Scaffolding of the OdB3 genome
  - Conserved non-coding elements
  - Chromosome-scale feature density plots
  - Modifying coalescent cutoffs
  - Regions interrupting colinearity
  - Distance Measures
  - Genomic features of the breakpoints
  - Genomic features of the breakpoints (heatmaps)
  - Inversions
  - Scaffolding of the KUM-M3 genome
  - Load genomic break data
  - Operon conservation analysis
  - Orthogroup syntenies
  - Alignment line plots, whole genomes
  - Region of interest: PAC3
  - Motif search and analysis
  - Whole-chromosome parallel plots
  - Width of the aligned and mapped regions.
  - Regions of interest
  - Repeat regions
  - Scaling breakpoints
  - Segment movement distances
  - Alignment line plots, 10×10 Mbp windows
  - Transcription factor binding sites
  - Tandem site duplications
  - Translocations
- Changelog
- Reference

# Scaling breakpoints

#### Michael Mansfield

#### Charles Plessy

#### 15 November, 2023

Source: `vignettes/ScalingBreakpoints.Rmd`

`ScalingBreakpoints.Rmd`

```
knitr::opts_chunk$set(cache = TRUE)
```

## Introduction

In this vignette computes the observed number of breakpoints for a
given species pair by a few key metrics:

- Millions of years since divergence
- Branch length (i.e., a kind of substitution rate)
- dN/dS

**Panel E of Figure 7 was generated with this vignette (colors
were edited by hand).**

**The vignette also produce trees for panels 7A–D.**

The core functions used here are maintained in our
*GenomicBreaks* R package, which is fully documented at: https://oist.github.io/GenomicBreaks.

## Setup

### Load R packages and data

```
library('OikScrambling') |> suppressPackageStartupMessages()
library('dplyr') |> suppressPackageStartupMessages()
genomes <- OikScrambling:::loadAllGenomes()
```

```
## Warning in runHook(".onLoad", env, package.lib, package): input string
## 'Génoscope' cannot be translated from 'ANSI_X3.4-1968' to UTF-8, but is valid
## UTF-8

## Warning in runHook(".onLoad", env, package.lib, package): input string
## 'Génoscope' cannot be translated from 'ANSI_X3.4-1968' to UTF-8, but is valid
## UTF-8
```

```
library('ggtree') |> suppressPackageStartupMessages()
library('treeio') |> suppressPackageStartupMessages()
library('phangorn') |> suppressPackageStartupMessages()
load("BreakPoints.Rdata")
```

### Service functions

```
toTreedata <- function(tree) as.treedata(midpoint(read.tree(text=tree)))
plotTree <- function(sp) {
  ggtree(sp, ladderize=T) +
    geom_tiplab(align=T) + hexpand(0.5) +
    geom_text(aes(x=branch, label=round(branch.length,2)), nudge_y=0.1) +
    geom_treescale(width = 0.02) + geom_rootedge()
}
```

## Inputting distance metrics

The resulting trees are displayed to help proofchecking.

### *Oikopleura*

```
species <- SimpleList()
species$Oikopleura <- "(Kum:0.01136111178249175555,((Osa:0.00925951950869623869,Aom:0.00920798155370218530)100:0.05729289951873162146,(Bar:0.00721724152898447612,Nor:0.00688754343189192098)100:0.05951986243208125943)100:0.31437914450027298896,Oki:0.01142149265410641150);" |> toTreedata()
plotTree(species$Oikopleura)
```

### *Ciona*

```
species$Ciona <- "(Rob:0.03128841990749874763,(Ros:0.01304087257513942937,Ply:0.01288782021364278871)100:0.02128682526879402057,Sav:0.55033545948338902232);" |> toTreedata()
plotTree(species$Ciona)
```

### *Drosophila*

The tree originates from https://github.com/SchriderLab/Drosophila\_phylogeny, and
the relevant clades were extracted with:

```
tree <- read.tree('Drosophila_phylogeny/tree/iqtree.tre')
tree <- drop.tip(tree, which(!tmp$tip.label %in% c('D_melanogaster', 'D_busckii', 'D_yakuba', 'D_subpulchrella', 'D_mauritiana')))
write.tree(tree, 'drosophila_4spp.newick')
```

```
species$Drosophila <- "(Dbu:0.3372973403,(Dsu:0.0947791254,(Dya:0.0703231461,(Dma:0.0280994984,Dme:0.0264284139)100_1_100:0.0267889071)100_1_100:0.0834632289)100_1_100:0.3712228549)100_1_100;" |> toTreedata()
plotTree(species$Drosophila)
```

### *Caenorhabditis*

Tree topologies taken from https://zenodo.org/record/1402254/files/RAxML\_species\_tree.nwk

The Zenodo link also has trees calculated using ASTRAL and
PhyloBayes. However, since the other trees here are ML trees, the RAxML
tree was elected for maximum comparability.

```
species$Caenorhabditis <- ape::read.tree(text="(CGUAD:0.10367478589587060533,(((CSP43:0.12383810371576783882,CVIRI:0.15037624074929095697)100:0.01497242793907414826,((CPLIC:0.26149908669946614337,((DCORO:0.59783158562631621979,CMONO:0.24005751591713977988)100:0.04780411675113813846,CSP21:0.36368990218252150726)100:0.10208494374306537056)100:0.03524817284922288768,((CCAST:0.01140212554998711741,CANGA:0.01414003367272922800)100:0.05852891527717005854,CSP38:0.05923127343611131468)100:0.11896541306229711787)98:0.01073075219687256478)100:0.02583935505413810507,((CKAMA:0.05811607292620620674,((CELEG:0.04500887872372311138,CSP34:0.07438201522663016874)100:0.00705149839556874856,(((CLATE:0.00465806789922303816,CREMA:0.00437786777324163708)100:0.02881157060040358958,((CNIGO:0.00418426739023885358,CBRIG:0.00712154940320354219)100:0.02811281384583694601,(CSINI:0.01686763323477479329,(CSP26:0.01529017647605383778,CSP40:0.02038338447129417474)100:0.00329577341321305025)100:0.01374371022227172112)100:0.01161394824780575923)100:0.00600824892975430989,((CDOUG:0.02983523068133404899,CBREN:0.03506566315430653669)80:0.00382524869406435401,(CWALL:0.01854144710471255370,CTROP:0.02398428150833109909)100:0.01471009970302557951)100:0.00927549430957550110)100:0.00763851852828213371)100:0.00805135107229970441)100:0.01328077288786689257,((((CNOUR:0.00956145701022028879,CSP29:0.00805739385901662990)100:0.01930453335852322372,(CSP39:0.03253018121281636005,CSP28:0.02417332848702777315)100:0.00890124689939411802)100:0.00884911431899864341,CMACR:0.03525690105425406673)100:0.02033464491626665177,(CJAPO:0.08234400443891473631,(CAFRA:0.02943481705945342336,CSP32:0.02434121084253646969)100:0.04055371130568757865)79:0.00548050967800332942)100:0.00698694826746024816)100:0.06205429593773054836)100:0.02963813048003952413,CSP31:0.12329044085858320567);") |>
  root("DCORO") |>
  reorder.phylo(order='cladewise')

species$Caenorhabditis <- drop.tip(species$Caenorhabditis, which(!species$Caenorhabditis$tip.label %in% c('CELEG', 'CSP34', 'CREMA', 'CNIGO', 'CBRIG')))
Caeno4 <-  drop.tip(species$Caenorhabditis, which(!species$Caenorhabditis$tip.label %in% c('CELEG', 'CREMA', 'CNIGO', 'CBRIG')))

species$Caenorhabditis$tip.label <- c('Cel', 'Cin', 'Cre', 'Cni', 'Cbr')
Caeno4$Caenorhabditis$tip.label <- c('Cel', 'Cre', 'Cni', 'Cbr')

write.tree(species$Caenorhabditis)
```

```
## [1] "((Cel:0.04500887872,Cin:0.07438201523)100:0.007051498396,(Cre:0.03318943837,(Cni:0.00418426739,Cbr:0.007121549403)100:0.03972676209)100:0.01364676746)100;"
```

```
species$Caenorhabditis <- as.treedata(species$Caenorhabditis)
Caeno4 <- as.treedata(Caeno4)
plotTree(species$Caenorhabditis)
```

```
plotTree(Caeno4)
```

### Distances

With the species trees loaded, we can calculate per-pair branch
length distances using `ape::cophenetic()`.

```
species_dist <- SimpleList()
species_dist$cophenetic <- lapply(species, function(sp) cophenetic.phylo(sp@phylo)) |> SimpleList()
```

#### Tunicates

For tunicates, the distances expressed in million years are taken
from the molecular clock analysis **except** for the
same-species pairs where they are arbitrarily set to 3, as an
upper-bound estimate. The rationale is that as we are testing for speed,
using the maximally conceivable distance prevents us from overestimating
that speed.

```
# These are taken from the molecular clock analysis.
species_dist$mya <- SimpleList()
species_dist$mya$Oikopleura <- data.frame(
  'Oki'=c(0,       3,       24.5347, 24.5347, 24.5347),
  'Kum'=c(3,       0,       24.5347, 24.5347, 24.5347),
  'Osa'=c(24.5347, 24.5347, 0,       7.2383,  3),
  'Bar'=c(24.5347, 24.5347, 7.2383,  0,       7.2383),
  'Aom'=c(24.5347, 24.5347, 3,       7.2383,  0)
)

species_dist$mya$Ciona <- data.frame(
  'Sav'=c(0,        101.4633, 101.4633, 101.4633),
  'Rob'=c(101.4633, 0,        11.8551, 11.8551),
  'Ply'=c(101.4633, 11.8551,  0, 3),
  'Ros'=c(101.4633, 11.8551,  3, 0)
)
```

#### Drosophila

For Drosophila, the numbers are taken from https://www.sciencedirect.com/science/article/pii/S0960982221014962
which links to https://github.com/SchriderLab/Drosophila\_phylogeny.
Note that the smallest distance here is 3.6 million years, close to our
upper-bound estimate for same-species tunicate populations.

```
species_dist$mya$Drosophila <- data.frame(
  'Dbu'=c(0,      46.836,  46.836,  46.836,  46.836),
  'Dsu'=c(46.836, 0,       13.1873, 13.1873, 13.1873),
  'Dya'=c(46.836, 13.1873, 0,       6.7256,  6.7256),
  'Dme'=c(46.836, 13.1873, 6.7256,  0,       3.6217),
  'Dma'=c(46.836, 13.1873, 6.7256,  3.6217,  0)
)
```

#### Caenorhabditis

See https://academic.oup.com/nar/article/46/3/1295/4793371
Figure S2 for all distances.

```
species_dist$mya$Caenorhabditis <- data.frame(
  'Cin'=c(0,        30, 30, 30, NA),
  'Cre'=c(30, 0,        25, 25, 30),
  'Cbr'=c(30, 25,  0, 3.7, 30),
  'Cni'=c(30, 25,  3.7, 0, 30),
  'Cel'=c(NA, 30,  30, 30, 0)
)
```

```
species_dist$mya <- lapply(species_dist$mya, function(x) {
  rownames(x) <- colnames(x)
  x
}) |> SimpleList()
```

## Normalised breakpoint accumulation

```
bp_vs_dist <- function(query, target, wgo, dist_type="mya", species, extra = FALSE) {
  dist_df <- species_dist[[dist_type]][[species]]
  relevant_dist <- dist_df[rownames(dist_df)==query, colnames(dist_df)==target]
  breakpoints <- wgo[wgo$type == "breakpoint region"]
  wgo_size <- wgo |> width() |> sum()
  wgo_size_mbp <- wgo_size / 1000000
  aln_length <- wgo[wgo$type %in% c("collinear alignment", "isolated alignment")] |> width() |> sum()
  breakpoints_per_scale = length(breakpoints)/relevant_dist
  breakpoints_per_mbp = length(breakpoints)/wgo_size_mbp
  breakpoints_per_aln_mbp <- length(breakpoints) / aln_length * 1e6
  data.frame(
    species                    = species,
    query                      = query,
    target                     = target,
    pair                       = paste0(query, "_", target),
    dist_type                  = dist_type,
    dist                       = relevant_dist,
    breakpoints                = length(breakpoints),
    breakpoints_per_scale      = breakpoints_per_scale,
    breakpoints_per_mbp        = breakpoints_per_mbp,
    breakpoints_per_mbp_scaled = breakpoints_per_mbp / relevant_dist,
    breakpoints_per_aln_mbp        = breakpoints_per_aln_mbp,
    breakpoints_per_aln_mbp_scaled = breakpoints_per_aln_mbp / relevant_dist,
    extra                      = extra)
}

mya_df <- rbind(
  bp_vs_dist(query="Osa", target="Oki", wgo$Osa_Oki, dist_type="mya", "Oikopleura"),
  bp_vs_dist(query="Oki", target="Bar", wgo$Oki_Bar, dist_type="mya", "Oikopleura", extra = TRUE),
  bp_vs_dist(query="Osa", target="Bar", wgo$Osa_Bar, dist_type="mya", "Oikopleura"),
  bp_vs_dist(query="Bar", target="Aom", wgo$Bar_Aom, dist_type="mya", "Oikopleura", extra = TRUE),
  bp_vs_dist(query="Osa", target="Aom", wgo$Osa_Aom, dist_type="mya", "Oikopleura"),
  bp_vs_dist(query="Oki", target="Kum", wgo$Oki_Kum, dist_type="mya", "Oikopleura", extra = TRUE),

  bp_vs_dist(query="Ply", target="Sav", wgo$Ply_Sav, dist_type="mya", "Ciona"),
  bp_vs_dist(query="Rob", target="Sav", wgo$Rob_Sav, dist_type="mya", "Ciona", extra = TRUE),
  bp_vs_dist(query="Ply", target="Rob", wgo$Ply_Rob, dist_type="mya", "Ciona"),
  bp_vs_dist(query="Rob", target="Ros", wgo$Rob_Ros, dist_type="mya", "Ciona", extra = TRUE),
  bp_vs_dist(query="Ply", target="Ros", wgo$Ply_Ros, dist_type="mya", "Ciona"),

  bp_vs_dist(query="Dme", target="Dbu", wgo$Dme_Dbu, dist_type="mya", "Drosophila"),
  bp_vs_dist(query="Dme", target="Dsu", wgo$Dme_Dsu, dist_type="mya", "Drosophila"),
  bp_vs_dist(query="Dme", target="Dya", wgo$Dme_Dya, dist_type="mya", "Drosophila"),
  
  bp_vs_dist(query="Cni", target="Cbr", wgo$Cni_Cbr, dist_type="mya", "Caenorhabditis", extra = TRUE),
  bp_vs_dist(query="Cni", target="Cre", wgo$Cni_Cre, dist_type="mya", "Caenorhabditis", extra = TRUE),
  bp_vs_dist(query="Cni", target="Cin", wgo$Cni_Cin, dist_type="mya", "Caenorhabditis", extra = TRUE),
  bp_vs_dist(query="Cbr", target="Cni", wgo$Cbr_Cni, dist_type="mya", "Caenorhabditis"),
  bp_vs_dist(query="Cbr", target="Cre", wgo$Cbr_Cre, dist_type="mya", "Caenorhabditis"),
  bp_vs_dist(query="Cbr", target="Cel", wgo$Cbr_Cel, dist_type="mya", "Caenorhabditis")
  
)
mya_df$species <- factor(mya_df$species, levels=c("Caenorhabditis","Drosophila", "Ciona", "Oikopleura"))
mya_df$pair <- factor(mya_df$pair,
                      levels=c("Cni_Cin", "Cbr_Cel", "Cni_Cre", "Cbr_Cre", "Cni_Cbr", "Cbr_Cni",
                               "Dme_Dbu", "Dme_Dsu", "Dme_Dya",
                               "Ply_Sav", "Rob_Sav", "Ply_Rob", "Rob_Ros", "Ply_Ros",
                               "Osa_Oki", "Oki_Bar", "Osa_Bar", "Bar_Aom", "Osa_Aom", "Oki_Kum"))
rownames(mya_df) <- NULL
mya_df$comp <- factor(c("far", "far", "mid", "mid", "close", "close",
                        "far", "far", "mid", "mid", "close",
                        "far", "mid", "close",
                        "close", "mid", "far", "close", "mid", "far"),
                      levels=c("close", "mid", "far"))
(mya_df)
```

```
##           species query target    pair dist_type     dist breakpoints
## 1      Oikopleura   Osa    Oki Osa_Oki       mya  24.5347        8815
## 2      Oikopleura   Oki    Bar Oki_Bar       mya  24.5347        8515
## 3      Oikopleura   Osa    Bar Osa_Bar       mya   7.2383        4034
## 4      Oikopleura   Bar    Aom Bar_Aom       mya   7.2383        4129
## 5      Oikopleura   Osa    Aom Osa_Aom       mya   3.0000        4063
## 6      Oikopleura   Oki    Kum Oki_Kum       mya   3.0000        4119
## 7           Ciona   Ply    Sav Ply_Sav       mya 101.4633        4027
## 8           Ciona   Rob    Sav Rob_Sav       mya 101.4633        4110
## 9           Ciona   Ply    Rob Ply_Rob       mya  11.8551        4668
## 10          Ciona   Rob    Ros Rob_Ros       mya  11.8551        4199
## 11          Ciona   Ply    Ros Ply_Ros       mya   3.0000        6304
## 12     Drosophila   Dme    Dbu Dme_Dbu       mya  46.8360        5951
## 13     Drosophila   Dme    Dsu Dme_Dsu       mya  13.1873         984
## 14     Drosophila   Dme    Dya Dme_Dya       mya   6.7256        1776
## 15 Caenorhabditis   Cni    Cbr Cni_Cbr       mya   3.7000        9504
## 16 Caenorhabditis   Cni    Cre Cni_Cre       mya  25.0000        3480
## 17 Caenorhabditis   Cni    Cin Cni_Cin       mya  30.0000        2853
## 18 Caenorhabditis   Cbr    Cni Cbr_Cni       mya   3.7000        9394
## 19 Caenorhabditis   Cbr    Cre Cbr_Cre       mya  25.0000        3434
## 20 Caenorhabditis   Cbr    Cel Cbr_Cel       mya  30.0000        3675
##    breakpoints_per_scale breakpoints_per_mbp breakpoints_per_mbp_scaled
## 1              359.28705          161.499480                  6.5824926
## 2              347.05947          135.508993                  5.5231567
## 3              557.31318           73.885230                 10.2075391
## 4              570.43781           75.692099                 10.4571652
## 5             1354.33333           74.325365                 24.7751216
## 6             1373.00000           64.753470                 21.5844899
## 7               39.68923           31.374368                  0.3092189
## 8               40.50726           34.708245                  0.3420768
## 9              393.75459           36.322159                  3.0638425
## 10             354.19355           34.975105                  2.9502159
## 11            2101.33333           49.021171                 16.3403902
## 12             127.06038           43.816224                  0.9355245
## 13              74.61725            7.275702                  0.5517204
## 14             264.06566           12.999373                  1.9328198
## 15            2568.64865           74.345755                 20.0934473
## 16             139.20000           27.341206                  1.0936482
## 17              95.10000           22.411011                  0.7470337
## 18            2538.91892           88.673653                 23.9658522
## 19             137.36000           32.416237                  1.2966495
## 20             122.50000           34.715517                  1.1571839
##    breakpoints_per_aln_mbp breakpoints_per_aln_mbp_scaled extra  comp
## 1                276.71488                     11.2785110 FALSE   far
## 2                269.76013                     10.9950449  TRUE   far
## 3                103.79013                     14.3390199 FALSE   mid
## 4                106.10175                     14.6583797  TRUE   mid
## 5                 85.95773                     28.6525752 FALSE close
## 6                 77.34894                     25.7829811  TRUE close
## 7                238.82854                      2.3538416 FALSE   far
## 8                239.13353                      2.3568476  TRUE   far
## 9                 51.62846                      4.3549580 FALSE   mid
## 10                47.49993                      4.0067082  TRUE   mid
## 11                60.25053                     20.0835106 FALSE close
## 12               201.50338                      4.3023182 FALSE   far
## 13                12.15188                      0.9214837 FALSE   mid
## 14                16.89433                      2.5119445 FALSE close
## 15               127.93670                     34.5774875  TRUE close
## 16               106.71003                      4.2684014  TRUE   mid
## 17               136.47305                      4.5491015  TRUE   far
## 18               126.62831                     34.2238664 FALSE close
## 19               104.47614                      4.1790457 FALSE   mid
## 20               147.64673                      4.9215578 FALSE   far
```

Breakpoint computation is tricky as expected number increases with
time but the sensitivity of the counting decreases with distance. For
instance we might need to mask repeat movements, which count as
breakpoints but are better detected in closely related genomes. Also the
Drosophila centromeres may need to be masked. But at this level of
manual curation it may be better to find a more robust metric
instead.

```
sub_df <- rbind(
  bp_vs_dist(query="Osa", target="Oki", wgo$Osa_Oki, dist_type="cophenetic", "Oikopleura"),
  bp_vs_dist(query="Osa", target="Bar", wgo$Osa_Bar, dist_type="cophenetic", "Oikopleura"),
  bp_vs_dist(query="Osa", target="Aom", wgo$Osa_Aom, dist_type="cophenetic", "Oikopleura"),

  bp_vs_dist(query="Ply", target="Ros", wgo$Ply_Ros, dist_type="cophenetic", "Ciona"),
  bp_vs_dist(query="Ply", target="Rob", wgo$Ply_Rob, dist_type="cophenetic", "Ciona"),
  bp_vs_dist(query="Ply", target="Sav", wgo$Ply_Sav, dist_type="cophenetic", "Ciona"),

  bp_vs_dist(query="Dme", target="Dbu", wgo$Dme_Dbu, dist_type="cophenetic", "Drosophila"),
  bp_vs_dist(query="Dme", target="Dsu", wgo$Dme_Dsu, dist_type="cophenetic", "Drosophila"),
  bp_vs_dist(query="Dme", target="Dya", wgo$Dme_Dya, dist_type="cophenetic", "Drosophila"),
  
  bp_vs_dist(query="Cni", target="Cin", wgo$Cni_Cin, dist_type="cophenetic", "Caenorhabditis"),
  bp_vs_dist(query="Cni", target="Cre", wgo$Cni_Cre, dist_type="cophenetic", "Caenorhabditis"),
  bp_vs_dist(query="Cni", target="Cbr", wgo$Cni_Cbr, dist_type="cophenetic", "Caenorhabditis")
)
sub_df$species <- factor(sub_df$species, levels=c("Caenorhabditis", "Drosophila", "Ciona", "Oikopleura"))
sub_df <- sub_df[order(sub_df$species, sub_df$breakpoints_per_mbp_scaled),]
rownames(sub_df) <- NULL
sub_df$pair <- factor(sub_df$pair, levels=sub_df$pair)
sub_df$comp <- factor(c("far", "mid", "close"), levels=c("close", "mid", "far"))
(sub_df)
```

```
##           species query target    pair  dist_type       dist breakpoints
## 1  Caenorhabditis   Cni    Cin Cni_Cin cophenetic 0.13899131        2853
## 2  Caenorhabditis   Cni    Cre Cni_Cre cophenetic 0.07710047        3480
## 3  Caenorhabditis   Cni    Cbr Cni_Cbr cophenetic 0.01130582        9504
## 4      Drosophila   Dme    Dsu Dme_Dsu cophenetic 0.23145968         984
## 5      Drosophila   Dme    Dbu Dme_Dbu cophenetic 0.84520075        5951
## 6      Drosophila   Dme    Dya Dme_Dya cophenetic 0.12354047        1776
## 7           Ciona   Ply    Sav Ply_Sav cophenetic 0.58451010        4027
## 8           Ciona   Ply    Rob Ply_Rob cophenetic 0.06546307        4668
## 9           Ciona   Ply    Ros Ply_Ros cophenetic 0.02592869        6304
## 10     Oikopleura   Osa    Oki Osa_Oki cophenetic 0.39235306        8815
## 11     Oikopleura   Osa    Bar Osa_Bar cophenetic 0.13328952        4034
## 12     Oikopleura   Osa    Aom Osa_Aom cophenetic 0.01846750        4063
##    breakpoints_per_scale breakpoints_per_mbp breakpoints_per_mbp_scaled
## 1              20526.463           22.411011                  161.24037
## 2              45135.913           27.341206                  354.61790
## 3             840629.224           74.345755                 6575.88535
## 4               4251.280            7.275702                   31.43400
## 5               7040.931           43.816224                   51.84120
## 6              14375.856           12.999373                  105.22360
## 7               6889.530           31.374368                   53.67635
## 8              71307.385           36.322159                  554.84966
## 9             243128.339           49.021171                 1890.61481
## 10             22467.010          161.499480                  411.61775
## 11             30264.944           73.885230                  554.32137
## 12            220008.110           74.325365                 4024.65739
##    breakpoints_per_aln_mbp breakpoints_per_aln_mbp_scaled extra  comp
## 1                136.47305                      981.88185 FALSE   far
## 2                106.71003                     1384.03874 FALSE   mid
## 3                127.93670                    11316.00715 FALSE close
## 4                 12.15188                       52.50108 FALSE   far
## 5                201.50338                      238.40889 FALSE   mid
## 6                 16.89433                      136.75142 FALSE close
## 7                238.82854                      408.59608 FALSE   far
## 8                 51.62846                      788.66551 FALSE   mid
## 9                 60.25053                     2323.70109 FALSE close
## 10               276.71488                      705.27011 FALSE   far
## 11               103.79013                      778.68182 FALSE   mid
## 12                85.95773                     4654.54017 FALSE close
```

This said, O. dioica is always ahead of other species pairs after
normalising by sequence length and evolutionary distance.

```
customPlot <- function(df, what, title, extra = "drop") {
  if (extra == "drop" & ! is.null(df$extra)) df <- df[! df$extra,]
  ggplot(df) +
    aes(x=.data[[what]], y = pair, fill = species) +
    geom_bar(stat='identity') +
    facet_wrap(~comp, scales="free") +
    theme_bw() +
    ggtitle(title)
}
```

### Panel used for Figure 7

```
# Show the nematodes between Oiks and ascidians.

mya_df$pair <- mya_df$pair |> factor(c("Dme_Dbu", "Dme_Dsu", "Dme_Dya",
  "Ply_Sav", "Rob_Sav", "Ply_Rob", "Rob_Ros", "Ply_Ros",
  "Cni_Cin", "Cbr_Cel", "Cni_Cre", "Cbr_Cre", "Cni_Cbr", "Cbr_Cni",
  "Osa_Oki", "Oki_Bar", "Osa_Bar", "Bar_Aom", "Osa_Aom", "Oki_Kum"))

customPlot(mya_df, 'breakpoints_per_mbp_scaled', 'Number of breakpoints per megabase per million years diverged')
```

### Supplemental panels for figure 7

```
customPlot(mya_df, 'breakpoints', 'Number of breakpoints')
```

```
customPlot(mya_df, 'breakpoints_per_scale', 'Number of breakpoints per million years')
```

```
customPlot(mya_df, 'breakpoints_per_mbp', 'Number of breakpoints per megabase')
```

```
customPlot(mya_df, 'breakpoints_per_aln_mbp', 'Number of breakpoints per megabase aligned')
```

```
customPlot(mya_df, 'breakpoints_per_mbp_scaled', 'Number of breakpoints per megabase per million years diverged', extra = TRUE)
```

```
customPlot(mya_df, 'breakpoints_per_aln_mbp_scaled', 'Number of breakpoints per megabase aligned per million years diverged')
```

```
customPlot(mya_df, 'breakpoints_per_aln_mbp_scaled', 'Number of breakpoints per megabase aligned per million years diverged', extra = TRUE)
```

```
customPlot(sub_df, 'breakpoints_per_mbp_scaled', 'Number of breakpoints per megabase per substitution distance')
```

```
customPlot(sub_df, 'breakpoints_per_aln_mbp_scaled', 'Number of breakpoints per megabase aligned per substitution distance')
```

## Session information

```
sessionInfo()
```

```
## R version 4.3.1 (2023-06-16)
## Platform: x86_64-pc-linux-gnu (64-bit)
## Running under: Debian GNU/Linux 12 (bookworm)
## 
## Matrix products: default
## BLAS:   /usr/lib/x86_64-linux-gnu/blas/libblas.so.3.11.0 
## LAPACK: /usr/lib/x86_64-linux-gnu/lapack/liblapack.so.3.11.0
## 
## locale:
##  [1] LC_CTYPE=C.UTF-8       LC_NUMERIC=C           LC_TIME=C.UTF-8       
##  [4] LC_COLLATE=C.UTF-8     LC_MONETARY=C.UTF-8    LC_MESSAGES=C.UTF-8   
##  [7] LC_PAPER=C.UTF-8       LC_NAME=C              LC_ADDRESS=C          
## [10] LC_TELEPHONE=C         LC_MEASUREMENT=C.UTF-8 LC_IDENTIFICATION=C   
## 
## time zone: Etc/UTC
## tzcode source: system (glibc)
## 
## attached base packages:
## [1] stats4    stats     graphics  grDevices utils     datasets  methods  
## [8] base     
## 
## other attached packages:
##  [1] phangorn_2.11.1                        
##  [2] ape_5.7-1                              
##  [3] treeio_1.24.1                          
##  [4] ggtree_3.8.0                           
##  [5] BSgenome.Oidioi.genoscope.OdB3_1.0.0   
##  [6] BSgenome.Oidioi.OIST.AOM.5.5f_1.0.1    
##  [7] BSgenome.Oidioi.OIST.KUM.M3.7f_1.0.1   
##  [8] BSgenome.Oidioi.OIST.Bar2.p4_1.0.1     
##  [9] BSgenome.Oidioi.OIST.OSKA2016v1.9_1.0.0
## [10] BSgenome.Oidioi.OIST.OKI2018.I69_1.0.1 
## [11] dplyr_1.1.3                            
## [12] OikScrambling_5.0.0                    
## [13] ggplot2_3.4.3                          
## [14] GenomicBreaks_0.14.2                   
## [15] BSgenome_1.68.0                        
## [16] rtracklayer_1.60.0                     
## [17] Biostrings_2.68.1                      
## [18] XVector_0.40.0                         
## [19] GenomicRanges_1.52.0                   
## [20] GenomeInfoDb_1.36.1                    
## [21] IRanges_2.34.1                         
## [22] S4Vectors_0.38.1                       
## [23] BiocGenerics_0.46.0                    
## 
## loaded via a namespace (and not attached):
##   [1] splines_4.3.1               BiocIO_1.10.0              
##   [3] ggplotify_0.1.1             bitops_1.0-7               
##   [5] tibble_3.2.1                R.oo_1.25.0                
##   [7] XML_3.99-0.14               rpart_4.1.19               
##   [9] lifecycle_1.0.3             rprojroot_2.0.3            
##  [11] lattice_0.20-45             MASS_7.3-58.2              
##  [13] backports_1.4.1             magrittr_2.0.3             
##  [15] Hmisc_5.1-0                 sass_0.4.7                 
##  [17] rmarkdown_2.23              jquerylib_0.1.4            
##  [19] yaml_2.3.7                  plotrix_3.8-2              
##  [21] DBI_1.1.3                   CNEr_1.36.0                
##  [23] minqa_1.2.5                 RColorBrewer_1.1-3         
##  [25] ade4_1.7-22                 abind_1.4-5                
##  [27] zlibbioc_1.46.0             quadprog_1.5-8             
##  [29] purrr_1.0.2                 R.utils_2.12.2             
##  [31] RCurl_1.98-1.12             yulab.utils_0.0.6          
##  [33] nnet_7.3-18                 pracma_2.4.2               
##  [35] GenomeInfoDbData_1.2.10     tidytree_0.4.2             
##  [37] gdata_2.19.0                annotate_1.78.0            
##  [39] pkgdown_2.0.7               codetools_0.2-19           
##  [41] DelayedArray_0.26.7         tidyselect_1.2.0           
##  [43] shape_1.4.6                 aplot_0.1.10               
##  [45] farver_2.1.1                lme4_1.1-34                
##  [47] matrixStats_1.0.0           base64enc_0.1-3            
##  [49] GenomicAlignments_1.36.0    jsonlite_1.8.7             
##  [51] mitml_0.4-5                 Formula_1.2-5              
##  [53] survival_3.5-3              iterators_1.0.14           
##  [55] systemfonts_1.0.5           foreach_1.5.2              
##  [57] tools_4.3.1                 ragg_1.2.5                 
##  [59] Rcpp_1.0.11                 glue_1.6.2                 
##  [61] gridExtra_2.3               pan_1.8                    
##  [63] xfun_0.40                   MatrixGenerics_1.12.2      
##  [65] EBImage_4.42.0              withr_2.5.1                
##  [67] fastmap_1.1.1               boot_1.3-28.1              
##  [69] fansi_1.0.5                 digest_0.6.33              
##  [71] gridGraphics_0.5-1          R6_2.5.1                   
##  [73] mice_3.16.0                 textshaping_0.3.7          
##  [75] colorspace_2.1-0            GO.db_3.17.0               
##  [77] gtools_3.9.4                poweRlaw_0.70.6            
##  [79] jpeg_0.1-10                 RSQLite_2.3.1              
##  [81] weights_1.0.4               R.methodsS3_1.8.2          
##  [83] utf8_1.2.3                  tidyr_1.3.0                
##  [85] generics_0.1.3              data.table_1.14.8          
##  [87] httr_1.4.7                  htmlwidgets_1.6.2          
##  [89] S4Arrays_1.0.5              pkgconfig_2.0.3            
##  [91] gtable_0.3.4                blob_1.2.4                 
##  [93] htmltools_0.5.6.1           fftwtools_0.9-11           
##  [95] scales_1.2.1                Biobase_2.60.0             
##  [97] png_0.1-8                   ggfun_0.1.1                
##  [99] knitr_1.44                  heatmaps_1.24.0            
## [101] rstudioapi_0.15.0           tzdb_0.4.0                 
## [103] reshape2_1.4.4              rjson_0.2.21               
## [105] checkmate_2.2.0             nlme_3.1-162               
## [107] nloptr_2.0.3                cachem_1.0.8               
## [109] stringr_1.5.0               KernSmooth_2.23-20         
## [111] parallel_4.3.1              genoPlotR_0.8.11           
## [113] foreign_0.8-84              AnnotationDbi_1.62.2       
## [115] restfulr_0.0.15             desc_1.4.2                 
## [117] pillar_1.9.0                grid_4.3.1                 
## [119] vctrs_0.6.3                 jomo_2.7-6                 
## [121] xtable_1.8-4                cluster_2.1.4              
## [123] htmlTable_2.4.1             evaluate_0.22              
## [125] readr_2.1.4                 cli_3.6.1                  
## [127] locfit_1.5-9.8              compiler_4.3.1             
## [129] Rsamtools_2.16.0            rlang_1.1.1                
## [131] crayon_1.5.2                labeling_0.4.3             
## [133] plyr_1.8.8                  fs_1.6.3                   
## [135] stringi_1.7.12              BiocParallel_1.34.2        
## [137] munsell_0.5.0               lazyeval_0.2.2             
## [139] tiff_0.1-11                 glmnet_4.1-7               
## [141] Matrix_1.5-3                patchwork_1.1.2            
## [143] hms_1.1.3                   bit64_4.0.5                
## [145] KEGGREST_1.40.0             SummarizedExperiment_1.30.2
## [147] igraph_1.5.0.1              broom_1.0.5                
## [149] memoise_2.0.1               bslib_0.5.1                
## [151] fastmatch_1.1-3             bit_4.0.5
```

## Contents

Developed by Aleksandra Bliznina, Michael Mansfield, Charles Plessy.

Site built with pkgdown 2.0.7.
