# Supplementary material for: Extreme genome scrambling in marine planktonic Oikopleura dioica cryptic species
Source: Genome Res. 2024 Mar;34(3):426–40. doi: 10.1101/gr.278295.123 (PMC11067885; doi:10.1101/gr.278295.123)
Supplement: Supplement 4 [file Supplemental_Data.zip › Supplemental_Data.tar.xz!/Supplemental_Data/articles/ParallelPlots_files/figure-html/plot_intersect_GBs_with_tree_with_Kume_and_aom-1.pdf]

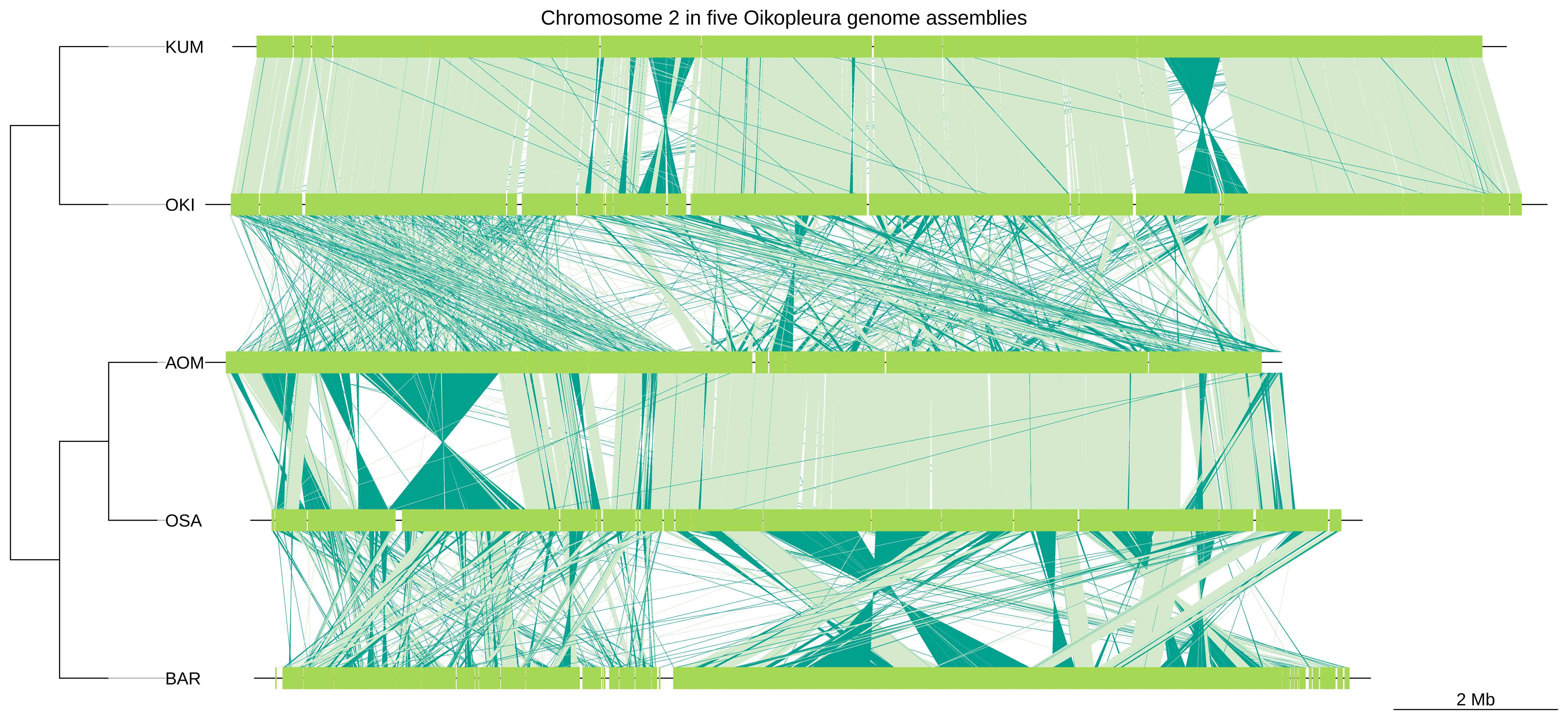

Chromosome 2 in five Oikopleura genome assemblies
KUM
OKI
AOM
OSA
BAR
2 Mb
