# Supplementary material for: Extreme genome scrambling in marine planktonic Oikopleura dioica cryptic species
Source: Genome Res. 2024 Mar;34(3):426–40. doi: 10.1101/gr.278295.123 (PMC11067885; doi:10.1101/gr.278295.123)
Supplement: Supplement 4 [file Supplemental_Data.zip › Supplemental_Data.tar.xz!/Supplemental_Data/articles/OxfordPlots.html]

Alignment line plots, whole genomes • OikScrambling
 


  


 


Toggle navigation


OikScrambling
5.0.0

- Get started
- Data load
- Supplemental material
  - Figure 1A DNA line plots
  - Figure 1B chromosome parallel plots
  - Table 1 scrambling index
  - Figure 5 genome-wide feature patterns
  - Figure 7 DNA line plots
  - Figure 7 breakpoint number barplots
- Articles
  - Conserved non-coding elements
  - Chromosome-scale feature density plots
  - Modifying coalescent cutoffs
  - Regions interrupting colinearity
  - Distance Measures
  - Genomic features of the breakpoints
  - Genomic features of the breakpoints (heatmaps)
  - Inversions
  - Load genomic break data
  - Operon conservation analysis
  - Orthogroup syntenies
  - Alignment line plots, whole genomes
  - Region of interest: PAC3
  - Motif search and analysis
  - Whole-chromosome parallel plots
  - Width of the aligned and mapped regions.
  - Regions of interest
  - Repeat regions
  - Scaling breakpoints
  - Segment movement distances
  - Alignment line plots, 10×10 Mbp windows
  - Transcription factor binding sites
  - Tandem site duplications
  - Translocations
- Changelog
- Reference

# Alignment line plots, whole genomes

#### Charles Plessy

#### 24 October, 2023

Source: `vignettes/OxfordPlots.Rmd`

`OxfordPlots.Rmd`

```
knitr::opts_chunk$set(cache = TRUE,
                      dev    = c('png', 'svg', 'pdf'),
                      fig.ext= c('png', 'svg', 'pdf'),
                      fig.width  = 15,
                      fig.height = 15)
```

## Introduction

This vignette produces alignment line plots of pairs of genomes to
illustrate differences in the extent of scrambling in
*Oikopleura* compared to equivalent pairs in other genera.

Figure 1 panel A was generated in this vignette.

## Load R pacakges and data

```
library('OikScrambling') |> suppressPackageStartupMessages()
ggplot2::theme_set(theme_bw())
genomes <- OikScrambling:::loadAllGenomes()
```

```
## Warning in runHook(".onLoad", env, package.lib, package): input string
## 'Génoscope' cannot be translated from 'ANSI_X3.4-1968' to UTF-8, but is valid
## UTF-8

## Warning in runHook(".onLoad", env, package.lib, package): input string
## 'Génoscope' cannot be translated from 'ANSI_X3.4-1968' to UTF-8, but is valid
## UTF-8
```

```
load("BreakPoints.Rdata")
```

## Oikopleura

### Fig 1A

Line plot used for panel A of Figure 1. Is based on aligned regions
(before coalescing), stored in the `gbs` object. The manually
set colors are used throughout the manuscript to represent chromosomes 1
and 2, the PAR, the XSR and the YSR.

```
makeOxfordPlots(gbs$Oki_Osa, diag = FALSE) +
  scale_color_manual(values = c("#E78AC3", "#A6D854", "#FFD92F", "#8DA0CB", "#66C2A5")) +
  theme_bw() +
  theme(legend.position="none")
```

### Okinawa vs others

#### Oki vs Osa (distant)

Same as above, but colored by strand.

```
makeOxfordPlots(gbs$Oki_Osa, diag = FALSE, col = "strand") + theme(legend.position="none")
```

#### Oki vs Bar (distant)

```
makeOxfordPlots(gbs$Oki_Bar, diag = FALSE, col = "strand") + theme(legend.position="none")
```

#### Oki vs Kum (near)

```
makeOxfordPlots(gbs$Oki_Kum, col = "strand") + theme(legend.position="none")
```

### Osaka vs others

#### Osa vs Oki (distant)

```
makeOxfordPlots(gbs$Osa_Oki, diag = FALSE, col = "strand") + theme(legend.position="none")
```

#### Osa vs Bar (intermediate)

```
makeOxfordPlots(gbs$Osa_Bar, diag = FALSE, col = "strand") + theme(legend.position="none")
```

#### Osa vs Aom (near)

```
makeOxfordPlots(gbs$Osa_Aom, col = "strand") + theme(legend.position="none")
```

### Barcelona vs others

#### Bar vs Oki (distant)

```
makeOxfordPlots(gbs$Bar_Oki, diag = FALSE, col = "strand") + theme(legend.position="none")
```

#### Bar vs Osa (intermediate)

```
makeOxfordPlots(gbs$Bar_Osa, diag = FALSE, col = "strand") + theme(legend.position="none")
```

#### Bar vs Nor (near)

In these vignettes, the manuscript’s “Bergen” genome is called
“Norway”.

**Note this plot does not demonstrate, nor rule out,
collinearity between the two genomes, because most contigs of the Bergen
assembly are too short. Alignment on the diagonal only demonstrates
collinearity *within* a contig, not
*between*.**

```
makeOxfordPlots(gbs$Bar_Nor, col = "strand") + theme(legend.position="none")
```

## Ciona

### *Ciona intestinalis* Plymouth vs Roscoff (near)

```
makeOxfordPlots(gbs$Ply_Ros, col = "strand") + theme(legend.position="none") + ggtitle("Ciona intestinalis Plymouth vs Roscoff")
```

### *Ciona intestinalis* Plymouth vs *Ciona robusta* (intermediate)

```
makeOxfordPlots(gbs$Ply_Rob, col = "strand") + theme(legend.position="none") + ggtitle("Ciona intestinalis Plymouth vs Ciona robusta")
```

### *Ciona intestinalis* Plymouth vs *Ciona savignyi* (distant)

```
makeOxfordPlots(gbs$Ply_Sav, col = "strand") + theme(legend.position="none") + ggtitle("Ciona intestinalis Plymouth vs Ciona savignyi")
```

## Drosophila

### vs Drosophila mauritania (near)

```
makeOxfordPlots(gbs$Dme_Dma, col = "strand") + theme(legend.position="none") + ggtitle("Dmel vs Dmau")
```

### vs Drosophila yakuba (intermediate)

```
makeOxfordPlots(gbs$Dme_Dya, col = "strand") + theme(legend.position="none") + ggtitle("Dmel vs Dyak")
```

### vs Drosophila subpulchrella (intermediate)

```
makeOxfordPlots(gbs$Dme_Dsu, col = "strand") + theme(legend.position="none") + ggtitle("Dmel vs Dsub")
```

### vs Drosophila buskii (distant)

```
makeOxfordPlots(gbs$Dme_Dbu, col = "strand") + theme(legend.position="none") + ggtitle("Dmel vs Dbus")
```

## Nematodes

### C. briggsae vs C. nigoni (near)

```
makeOxfordPlots(gbs$Cbr_Cni, col = "strand") + theme(legend.position="none") + ggtitle("C. briggsae vs C. nigoni")
```

### C. briggsae vs C. remanei (intermediate)

```
makeOxfordPlots(gbs$Cbr_Cre, col = "strand") + theme(legend.position="none") + ggtitle("C. briggsae vs C. remanei")
```

### C. nigoni vs C. remanei (intermediate)

```
makeOxfordPlots(gbs$Cni_Cre, col = "strand") + theme(legend.position="none") + ggtitle("C. nigoni vs C. remanei")
```

### C. briggsae vs C. elegans (distant)

```
makeOxfordPlots(gbs$Cbr_Cel, col = "strand") + theme(legend.position="none") + ggtitle("C. briggsae vs C. elegans")
```

### C. nigoni vs C. inopinata (distant)

```
makeOxfordPlots(gbs$Cni_Cin, col = "strand") + theme(legend.position="none") + ggtitle("C. nigoni vs C. inopinata")
```

## Mammals

### Human versus mouse chromosome 18

Chromosome 18 was chosen because it is small and it has the same name
in both species. Region was centered on the alignment with the longest
width, as otherwise the window out of target.

Note that each of these chromosomes is longer than any *Oikopleura
dioica* genome.

```
hgmm <- load_genomic_breaks(system.file(("extdata/hg38_mm10/hg38_chr18__mm10_chr18.gff3"), package = "BreakpointsData"))
makeOxfordPlots(hgmm, col = "strand") + ggtitle("Homo sapiens vs Mus musculus")
```

### Muntjaks

```
Mmun_Mree <- load_genomic_breaks(system.file("extdata/Muntjaks/Rrev_Rmun.07.postmasked.gff.gz", package = "BreakpointsData"), type = "match")
makeOxfordPlots(Mmun_Mree |> swap(), col = "strand") + ggtitle("Muntiacus reevesi vs Muntiacus muntjak")
```

## Contents

Developed by Aleksandra Bliznina, Michael Mansfield, Charles Plessy.

Site built with pkgdown 2.0.7.
