# Supplementary material for: Extreme genome scrambling in marine planktonic Oikopleura dioica cryptic species
Source: Genome Res. 2024 Mar;34(3):426–40. doi: 10.1101/gr.278295.123 (PMC11067885; doi:10.1101/gr.278295.123)
Supplement: Supplement 4 [file Supplemental_Data.zip › Supplemental_Data.tar.xz!/Supplemental_Data/articles/OperonDetection.html]

Operon detection and counting • OikScrambling
 


  


 


Toggle navigation


OikScrambling
5.1.0

- Get started
- Data load
- Supplemental material
  - Figure 1B and S1 DNA line plots
  - Figure 1C chromosome parallel plots
  - Figure 1D scrambling index
  - Figure 2A and S1 orthogroup dot plots and synteny block histograms
  - Figure 3C breakpoint feature density plots
  - Figure 4B and 4C operon barplots
  - Figure 5 genome-wide feature patterns
  - Figure 7A–D DNA line plots
  - Figure 7E breakpoint number barplots
- Articles
  - Scaffolding of the AOM-5 genome
  - Scaffolding of the OdB3 genome
  - Conserved non-coding elements
  - Chromosome-scale feature density plots
  - Modifying coalescent cutoffs
  - Regions interrupting colinearity
  - Distance Measures
  - Genomic features of the breakpoints
  - Genomic features of the breakpoints (heatmaps)
  - Inversions
  - Scaffolding of the KUM-M3 genome
  - Load genomic break data
  - Operon conservation analysis
  - Operon detection and counting
  - Orthogroup syntenies
  - Alignment line plots, whole genomes
  - Region of interest: PAC3
  - Motif search and analysis
  - Whole-chromosome parallel plots
  - Width of the aligned and mapped regions.
  - Regions of interest
  - Repeat regions
  - Scaling breakpoints
  - Segment movement distances
  - Alignment line plots, 10×10 Mbp windows
  - Transcription factor binding sites
  - Tandem site duplications
  - Translocations
- Changelog
- Reference

# Operon detection and counting

#### Michael Mansfield

#### Charles Plessy

#### 20 November, 2023

Source: `vignettes/OperonDetection.Rmd`

`OperonDetection.Rmd`

## Introduction

Computes operons as we need their boundaries in the figure panel.

\*\* Figure 4 panels B and C are produced by this vignette\*\*

## Load packages and data

See `?OikScrambling:::loadAllGenomes`,
`?OikScrambling:::loadAllTranscriptsGR`, and
`vignette("LoadGenomicBreaks", package = "OikScrambling")`
for how the different objects are prepared.

```
library('OikScrambling')   |> suppressPackageStartupMessages()
library('GenomicFeatures') |> suppressPackageStartupMessages()
library('heatmaps')        |> suppressPackageStartupMessages()
library('patchwork')       |> suppressPackageStartupMessages()
genomes     <- OikScrambling:::loadAllGenomes(compat = F)
```

```
## Warning in runHook(".onLoad", env, package.lib, package): input string
## 'Génoscope' cannot be translated from 'ANSI_X3.4-1968' to UTF-8, but is valid
## UTF-8

## Warning in runHook(".onLoad", env, package.lib, package): input string
## 'Génoscope' cannot be translated from 'ANSI_X3.4-1968' to UTF-8, but is valid
## UTF-8
```

```
# Cannot use Knitr cache as long as "annot" objects are used.
annots      <- OikScrambling:::loadAllAnnotations()   |> suppressWarnings()
reps        <- OikScrambling:::loadAllRepeats(compat = F)
genes       <- sapply(annots, function(a) sort(genes(a))) |> SimpleList()
load("BreakPoints.Rdata")
requireNamespace("rGADEM")    |> suppressPackageStartupMessages()
requireNamespace("ggseqlogo") |> suppressPackageStartupMessages()
ces <- readRDS("CEs.Rds")
tfbs <- readRDS("pwmMatchesOki_12_95.Rds")
```

## Model operon areas

We need operon boundaries in the plots generated later.

### Notes about operons

The operon of Ganot
et al., 2004 appears to be conserved in OKI and OSA (near
`OSKA2016v1.9::PAR:10984831-10995276`). In the annotation
visible in ZENBU and NCBI:

- `Oidioi.mRNA.OKI2018_I69.PAR.g12978` is the most likely
  start of the Ganot operon as it starts downstream of a CAGE TSS peak and
  has no trans-splicing site. It is homologous to
  `g10133 (PAR:10985271..10985910+)` in OSKA2016v1.9 which also
  has a transcription start site and no trans-splicing site. Best
  Norwegian hit is `CBY19033`. Gene name is unclear but weaker
  blast hits suggest *centrosomal POC5 isoform X1*.
- IQ domain-containing protein K isoform X2 is
  `Oidioi.mRNA.OKI2018_I69.PAR.g12977` and is
  `g10134` in OSKA2016. It has a bona fide trans-splicing site
  in both genomes. It is `CBY19034` in Norway. (Not reported in
  Ganot et al., 2004).
- Exportin 7 is `Oidioi.mRNA.OKI2018_I69.PAR.g12976`.
  (First member of the operon of Ganot et al., 2004, which calls it
  Ran-binding protein 16). In the current annotation of the OSKA2016v1.9
  genome, `g10135` matches well but misses the N-terminal part.
  Nevertheless a blastx search in region
  `PAR:10986742..10988032+` finds it. In line with this, there
  is a trans-splicing site upstream the unannotated area.
- Dynein light chain is
  `Oidioi.mRNA.OKI2018_I69.PAR.g12975`.
- Delta tubulin is
  `Oidioi.mRNA.OKI2018_I69.PAR.g12974`.
- *MBF* is `Oidioi.mRNA.OKI2018_I69.PAR.g12973`.
  Also called *enthelial differenciation-related factor 1* in other
  species.
- CycD is `Oidioi.mRNA.OKI2018_I69.PAR.g12972` and
  `g10138` in OSKA. Polyadenylation signal conserved.

(Note that the transcripts IDs here differ from the public ones.)

Ganot et al., 2004 reported very small (<30) intercistronic
regions and Denoeud et al., 2010 stated: “*The operons were predicted
as co-oriented genes separated by 60 nucleotides at most : 1761 operons
containing 4997 genes were predicted on the reference assembly*”.
However, in our annotation, we do not have the UTRs, but only the
distances between translation stops and starts, so we need a broader
window. The example below shows that a window of 400 would break the
Ganot 2004 operon, so let’s use 500 instead.

```
transcripts(annots$Oki) |> subsetByOverlaps(GRanges("PAR:15956430-15967745"))
```

```
## GRanges object with 8 ranges and 2 metadata columns:
##       seqnames            ranges strand |     tx_id     tx_name
##          <Rle>         <IRanges>  <Rle> | <integer> <character>
##   [1]      PAR 15956464-15957432      - |     14303   g12997.t1
##   [2]      PAR 15957795-15958315      - |     14304   g12998.t1
##   [3]      PAR 15958381-15959726      - |     14305   g12999.t1
##   [4]      PAR 15959875-15960185      - |     14306   g13000.t1
##   [5]      PAR 15960589-15964701      - |     14307   g13001.t1
##   [6]      PAR 15960653-15964701      - |     14308   g13001.t2
##   [7]      PAR 15964824-15965481      - |     14309   g13002.t1
##   [8]      PAR 15965608-15967583      - |     14310   g13003.t1
##   -------
##   seqinfo: 19 sequences from OKI2018.I69 genome
```

```
transcripts(annots$Oki) |> subsetByOverlaps(GRanges("PAR:15956430-15967745")) |> cleanGaps() |> width()
```

```
## [1] 362  65 148 403 122 126
```

### Compute operons

This function is reused (cut-and-paste) in other vignettes.

```
operons <- lapply(annots, \(a) {a |> genes() |> OikScrambling:::calcOperons(window = 500) }) |> SimpleList() |> suppressWarnings()
operons$Nor <- OikScrambling:::calcOperons(window = 500, transcripts(annots$Nor) |> reduce()) |> suppressWarnings()
sapply(operons, length)
```

```
##  Oki  Osa  Bar  Kum  Aom  Nor  Ply  Ros 
## 3124 2741 2379 2974 2648 2996 1088 1472
```

```
sapply(operons, \(o) {table(o$n)})
```

```
## $Oki
## 
##    2    3    4    5    6    7    8    9   10   11   14 
## 1943  647  259  134   69   33   19   10    6    3    1 
## 
## $Osa
## 
##    2    3    4    5    6    7    8    9   10   11   12   13 
## 1714  557  248   98   63   32   13   10    1    3    1    1 
## 
## $Bar
## 
##    2    3    4    5    6    7    8    9   10   11   12   13   14   17 
## 1450  475  221  106   57   30   23    9    3    1    1    1    1    1 
## 
## $Kum
## 
##    2    3    4    5    6    7    8    9   10   11   12   13   14   17 
## 1855  648  233  114   56   28   18    4    7    6    1    1    2    1 
## 
## $Aom
## 
##    2    3    4    5    6    7    8    9   10   11   12   14 
## 1689  492  239  107   52   36   14   10    3    4    1    1 
## 
## $Nor
## 
##    2    3    4    5    6    7    8    9   10   11   12   13   14   15   16   17 
## 1724  562  264  138   93   65   38   40   16   21   11   10    3    4    3    1 
##   20   22 
##    1    2 
## 
## $Ply
## 
##   2   3   4   5   6   7   8   9  13  14  15 
## 937 114  22   6   2   1   1   1   1   1   2 
## 
## $Ros
## 
##    2    3    4    5    6    7    9   10   11   21   26   29 
## 1262  163   33    5    1    1    1    1    1    1    1    2
```

With the OdB3 genome, the results are apparently close to those of
Denoeud et al., 2010 (see above for why the difference). We have a few
more (1844 instead of 1761), they contain more genes (5814 instead of
4997), mostly because our computation outputs 50 operons longer than 9
genes (617 genes in total). Nevertheless, the proportion of operons of
length 2, 3 and 4 are visually similar in both cases.

## Operons and genes

Do alignments often cross gene or operon boundaries ?

### Operon conservation

```
# Distribution of operon lengths
operons$Oki$n |> table()
```

```
## 
##    2    3    4    5    6    7    8    9   10   11   14 
## 1943  647  259  134   69   33   19   10    6    3    1
```

```
# An operon is scrambled if it overlaps with an unaligned noncoalesced (= unmapped) region
scrambled_operons     <- subsetByOverlaps(operons$Oki, unal$Oki_Osa[unal$Oki_Osa$nonCoa])
not_scrambled_operons <- operons$Oki[! operons$Oki %in% scrambled_operons ]

# Distribution of operon lengths (scrambled)
scrambled_operons$n     |> table()
```

```
## 
##   2   3   4   5   6   7   8   9  10  11 
## 905 298 106  44  31   9   7   6   4   3
```

```
not_scrambled_operons$n |> table()
```

```
## 
##    2    3    4    5    6    7    8    9   10   14 
## 1038  349  153   90   38   24   12    4    2    1
```

```
# Example of long scrambled operons
scrambled_operons[scrambled_operons$n > 5]
```

```
## GRanges object with 60 ranges and 2 metadata columns:
##        seqnames            ranges strand |         n                  gene_id
##           <Rle>         <IRanges>  <Rle> | <integer>          <CharacterList>
##    [1]     chr1   5715727-5730794      + |         7    g1521,g1522,g1523,...
##    [2]     chr1   6147428-6157837      + |         6    g1613,g1614,g1615,...
##    [3]     chr1   9809914-9836205      + |        10    g2620,g2621,g2622,...
##    [4]     chr1 10022216-10035952      + |         7    g2668,g2669,g2670,...
##    [5]     chr1 10547772-10569235      - |         9    g2832,g2833,g2834,...
##    ...      ...               ...    ... .       ...                      ...
##   [56]      XSR   6611822-6622862      + |         7 g15207,g15208,g15209,...
##   [57]      XSR   6843015-6861410      + |         8 g15282,g15283,g15284,...
##   [58]      XSR   7875989-7897781      - |         7 g15606,g15607,g15608,...
##   [59]      XSR 10693937-10705412      + |         6 g16499,g16500,g16501,...
##   [60]      XSR 12736159-12757464      - |         8 g17016,g17017,g17018,...
##   -------
##   seqinfo: 19 sequences from OKI2018.I69 genome
```

In comparison: genes

```
all_genes <- genes(annots$Oki)
scrambled_genes <- (subsetByOverlaps(all_genes, unal$Oki_Osa[unal$Oki_Osa$nonCoa])) |> sort(i=F) |> flagLongShort(longShort$OKI2018.I69)
not_scrambled_genes <- all_genes[! all_genes %in% scrambled_genes]                  |> sort(i=F) |> flagLongShort(longShort$OKI2018.I69)

length(scrambled_genes)
```

```
## [1] 5294
```

```
length(all_genes)
```

```
## [1] 17291
```

```
scr_g_tbl <- table(paste(seqnames(    scrambled_genes), scrambled_genes$Arm))
nsc_g_tbl <- table(paste(seqnames(not_scrambled_genes), not_scrambled_genes$Arm))

Oki_Osa_scr_ratio_long_arm <- c(
  scr_g_tbl[["chr1 long"]] / ( scr_g_tbl[["chr1 long"]] + nsc_g_tbl[["chr1 long"]]),
  scr_g_tbl[["chr2 long"]] / ( scr_g_tbl[["chr2 long"]] + nsc_g_tbl[["chr2 long"]]),
  scr_g_tbl[["PAR long"]]  / ( scr_g_tbl[["PAR long"]]  + nsc_g_tbl[["PAR long"]])
)
mean(Oki_Osa_scr_ratio_long_arm)
```

```
## [1] 0.2314521
```

```
Oki_Osa_scr_ratio_short_arm <- c(
  scr_g_tbl[["chr1 short"]] / ( scr_g_tbl[["chr1 short"]] + nsc_g_tbl[["chr1 short"]]),
  scr_g_tbl[["chr2 short"]] / ( scr_g_tbl[["chr2 short"]] + nsc_g_tbl[["chr2 short"]]),
  scr_g_tbl[["PAR short"]]  / ( scr_g_tbl[["PAR short"]]  + nsc_g_tbl[["PAR short"]])
)
mean(Oki_Osa_scr_ratio_short_arm)
```

```
## [1] 0.5374672
```

```
t.test(Oki_Osa_scr_ratio_long_arm, Oki_Osa_scr_ratio_short_arm, paired = T)
```

```
## 
##  Paired t-test
## 
## data:  Oki_Osa_scr_ratio_long_arm and Oki_Osa_scr_ratio_short_arm
## t = -9.5775, df = 2, p-value = 0.01073
## alternative hypothesis: true mean difference is not equal to 0
## 95 percent confidence interval:
##  -0.4434904 -0.1685397
## sample estimates:
## mean difference 
##      -0.3060151
```

Replicate on an independent pair of genomes.

```
all_genes <- genes(annots$Bar)
scrambled_genes <- (subsetByOverlaps(all_genes, unal$Bar_Kum[unal$Bar_Kum$nonCoa])) |> sort(i=F) |> flagLongShort(longShort$Bar2.p4)
not_scrambled_genes <- all_genes[! all_genes %in% scrambled_genes]                  |> sort(i=F) |> flagLongShort(longShort$Bar2.p4)

length(scrambled_genes)
```

```
## [1] 4002
```

```
length(all_genes)
```

```
## [1] 14272
```

```
scr_g_tbl <- table(paste(seqnames(    scrambled_genes), scrambled_genes$Arm))
nsc_g_tbl <- table(paste(seqnames(not_scrambled_genes), not_scrambled_genes$Arm))

Bar_Kum_scr_ratio_long_arm <- c(
  scr_g_tbl[["Chr1 long"]] / ( scr_g_tbl[["Chr1 long"]] + nsc_g_tbl[["Chr1 long"]]),
  scr_g_tbl[["Chr2 long"]] / ( scr_g_tbl[["Chr2 long"]] + nsc_g_tbl[["Chr2 long"]]),
  scr_g_tbl[["PAR long"]]  / ( scr_g_tbl[["PAR long"]]  + nsc_g_tbl[["PAR long"]])
)
Bar_Kum_scr_ratio_short_arm <- c(
  scr_g_tbl[["Chr1 short"]] / ( scr_g_tbl[["Chr1 short"]] + nsc_g_tbl[["Chr1 short"]]),
  scr_g_tbl[["Chr2 short"]] / ( scr_g_tbl[["Chr2 short"]] + nsc_g_tbl[["Chr2 short"]]),
  scr_g_tbl[["PAR short"]]  / ( scr_g_tbl[["PAR short"]]  + nsc_g_tbl[["PAR short"]])
)

t.test(Bar_Kum_scr_ratio_long_arm, Bar_Kum_scr_ratio_short_arm, paired = TRUE)
```

```
## 
##  Paired t-test
## 
## data:  Bar_Kum_scr_ratio_long_arm and Bar_Kum_scr_ratio_short_arm
## t = -16.994, df = 2, p-value = 0.003445
## alternative hypothesis: true mean difference is not equal to 0
## 95 percent confidence interval:
##  -0.3825454 -0.2279699
## sample estimates:
## mean difference 
##      -0.3052576
```

```
mean(c(Oki_Osa_scr_ratio_long_arm, Bar_Kum_scr_ratio_long_arm))
```

```
## [1] 0.2055334
```

```
mean(c(Oki_Osa_scr_ratio_short_arm, Bar_Kum_scr_ratio_short_arm))
```

```
## [1] 0.5111697
```

```
t.test(
  c(Oki_Osa_scr_ratio_long_arm, Bar_Kum_scr_ratio_long_arm),
  c(Oki_Osa_scr_ratio_short_arm, Bar_Kum_scr_ratio_short_arm),
  paired = TRUE
)
```

```
## 
##  Paired t-test
## 
## data:  c(Oki_Osa_scr_ratio_long_arm, Bar_Kum_scr_ratio_long_arm) and c(Oki_Osa_scr_ratio_short_arm, Bar_Kum_scr_ratio_short_arm)
## t = -18.644, df = 5, p-value = 8.172e-06
## alternative hypothesis: true mean difference is not equal to 0
## 95 percent confidence interval:
##  -0.3477765 -0.2634962
## sample estimates:
## mean difference 
##      -0.3056363
```

How about exons ?

```
all_exons <- exonicParts(annots$Oki)
scrambled_exons <- (subsetByOverlaps(all_exons, unal$Oki_Osa[unal$Oki_Osa$nonCoa])) |> sort(i=F) |> flagLongShort(longShort$OKI2018.I69)
not_scrambled_exons <- all_exons[! all_exons %in% scrambled_exons]                  |> sort(i=F) |> flagLongShort(longShort$OKI2018.I69)

length(scrambled_exons)
```

```
## [1] 16787
```

```
length(all_exons)
```

```
## [1] 106811
```

```
scr_g_tbl <- table(paste(seqnames(    scrambled_exons), scrambled_exons$Arm))
nsc_g_tbl <- table(paste(seqnames(not_scrambled_exons), not_scrambled_exons$Arm))

Oki_Osa_scr_ratio_long_arm <- c(
  scr_g_tbl[["chr1 long"]] / ( scr_g_tbl[["chr1 long"]] + nsc_g_tbl[["chr1 long"]]),
  scr_g_tbl[["chr2 long"]] / ( scr_g_tbl[["chr2 long"]] + nsc_g_tbl[["chr2 long"]]),
  scr_g_tbl[["PAR long"]]  / ( scr_g_tbl[["PAR long"]]  + nsc_g_tbl[["PAR long"]])
)
mean(Oki_Osa_scr_ratio_long_arm)
```

```
## [1] 0.1172023
```

```
Oki_Osa_scr_ratio_short_arm <- c(
  scr_g_tbl[["chr1 short"]] / ( scr_g_tbl[["chr1 short"]] + nsc_g_tbl[["chr1 short"]]),
  scr_g_tbl[["chr2 short"]] / ( scr_g_tbl[["chr2 short"]] + nsc_g_tbl[["chr2 short"]]),
  scr_g_tbl[["PAR short"]]  / ( scr_g_tbl[["PAR short"]]  + nsc_g_tbl[["PAR short"]])
)
mean(Oki_Osa_scr_ratio_short_arm)
```

```
## [1] 0.3003286
```

```
t.test(Oki_Osa_scr_ratio_long_arm, Oki_Osa_scr_ratio_short_arm, paired = T)
```

```
## 
##  Paired t-test
## 
## data:  Oki_Osa_scr_ratio_long_arm and Oki_Osa_scr_ratio_short_arm
## t = -5.9254, df = 2, p-value = 0.02732
## alternative hypothesis: true mean difference is not equal to 0
## 95 percent confidence interval:
##  -0.31610038 -0.05015222
## sample estimates:
## mean difference 
##      -0.1831263
```

## Operon summary plots for Figure 3

```
# Add an index for matching to scrambled set
operons$Oki$index <- 1:length(operons$Oki)
operons$Oki$scrambled <- 'no_breakpoint_region'
operons$Oki$scrambled[operons$Oki %over% unal$Oki_Osa[unal$Oki_Osa$nonCoa]] <- 'overlaps_breakpoint_region'

operons$Osa$index <- 1:length(operons$Osa)
operons$Osa$scrambled <- 'no_breakpoint_region'
operons$Osa$scrambled[operons$Osa %over% unal$Osa_Oki[unal$Osa_Oki$nonCoa]] <- 'overlaps_breakpoint_region'

operons$Bar$index <- 1:length(operons$Bar)
operons$Bar$scrambled <- 'no_breakpoint_region'
operons$Bar$scrambled[operons$Bar %over% unal$Bar_Oki[unal$Bar_Oki$nonCoa]] <- 'overlaps_breakpoint_region'

tb1 <- operons$Oki
# Note that the operon objects do not have unique names, which makes as.data.frame() complain.
names(tb1) <- NULL
p1 <- ggplot(as.data.frame(tb1)) + aes(y=factor(n, levels = 2:max(operons$Oki$n, operons$Osa$n))) + geom_bar() + geom_text(stat='count', aes(label=..count.., hjust=-0.2)) + expand_limits(x=c(0,750)) + ylab("number of genes in operon") + xlab("Count") + facet_wrap(~scrambled, nrow=2) + ggtitle("Okinawa operons") + scale_y_discrete(drop=F)
p1
```

```
tb2 <- operons$Osa
names(tb2) <- NULL
p2 <- ggplot(as.data.frame(tb2)) + aes(y=factor(n, levels = 2:max(operons$Oki$n, operons$Osa$n))) + geom_bar() + geom_text(stat='count', aes(label=..count.., hjust=-0.2)) + expand_limits(x=c(0,750)) + ylab("number of genes in operon") + xlab("Count") + facet_wrap(~scrambled, nrow=2) + ggtitle("Osaka operons") + scale_y_discrete(drop=F)
p2
```

```
rbind(
  operons$Oki |> as.data.frame() |> dplyr::mutate(species="Okinawa"),
  operons$Osa |> as.data.frame() |> dplyr::mutate(species="Osaka"),
  operons$Bar |> as.data.frame() |> dplyr::mutate(species="Barcelona")
) |> ggplot() +
  aes(y=factor(n)) +
  geom_bar() +
  facet_wrap(~scrambled + species) +
  geom_text(stat='count', aes(label=..count.., hjust=-0.2)) +
  theme_bw() +
  scale_x_continuous("Number operons", limits = c(0,1200)) +
  scale_y_discrete("Number of genes in operon")
```

```
p3 <- ggplot(as.data.frame(tb1)) + aes(y=factor(n, levels = 2:max(operons$Oki$n, operons$Osa$n))) + geom_bar() + geom_text(stat='count', aes(label=..count.., hjust=-0.2)) + expand_limits(x=c(0,750)) + ylab("number of genes in operon") + xlab("Count") + facet_wrap(~scrambled, ncol=2) + ggtitle("Okinawa operons") + scale_y_discrete(drop=F)
p3
```

```
p4 <- ggplot(as.data.frame(tb2)) + aes(y=factor(n, levels = 2:max(operons$Oki$n, operons$Osa$n))) + geom_bar() + geom_text(stat='count', aes(label=..count.., hjust=-0.2)) + expand_limits(x=c(0,750)) + ylab("number of genes in operon") + xlab("Count") + facet_wrap(~scrambled, ncol=2) + ggtitle("Osaka operons") + scale_y_discrete(drop=F)
p4
```

```
( p3 / p4 )
```

```
tb1$sp <- "Oki"
tb2$sp <- "Osa"
tb3 <- rbind(as.data.frame(tb1), as.data.frame(tb2))
ggplot(tb3) + aes(x=factor(n, levels = 2:max(operons$Oki$n, operons$Osa$n))) + geom_bar(position='dodge', aes(fill=paste0(sp, "_", scrambled))) + scale_x_discrete(drop=F)
```

```
genes$Oki$scrambled <- 'no_breakpoint_region'
genes$Oki$scrambled[genes$Oki %over% unal$Oki_Osa[unal$Oki_Osa$nonCoa]] <- 'overlaps_breakpoint_region'
genes$Oki$scrambled |> table()
```

```
## 
##       no_breakpoint_region overlaps_breakpoint_region 
##                      11997                       5294
```

```
genes$Osa$scrambled <- 'no_breakpoint_region'
genes$Osa$scrambled[genes$Osa %over% unal$Osa_Oki[unal$Osa_Oki$nonCoa]] <- 'overlaps_breakpoint_region'
genes$Osa$scrambled |> table()
```

```
## 
##       no_breakpoint_region overlaps_breakpoint_region 
##                      11617                       4103
```

```
genes$Bar$scrambled <- 'no_breakpoint_region'
genes$Bar$scrambled[genes$Bar %over% unal$Bar_Oki[unal$Bar_Oki$nonCoa]] <- 'overlaps_breakpoint_region'
genes$Bar$scrambled |> table()
```

```
## 
##       no_breakpoint_region overlaps_breakpoint_region 
##                      10265                       4007
```

```
exons <- sapply(annots, function(a) sort(exons(a))) |> SimpleList()
exons <- sapply(exons, function(e) {
  names(e) <- e$exon_id
  e
}) |> SimpleList()

exons$Oki$scrambled <- 'no_breakpoint_region'
exons$Oki$scrambled[exons$Oki %over% unal$Oki_Osa[unal$Oki_Osa$nonCoa]] <- 'overlaps_breakpoint_region'
exons$Oki$scrambled |> table()
```

```
## 
##       no_breakpoint_region overlaps_breakpoint_region 
##                     102165                      17383
```

```
exons$Osa$scrambled <- 'no_breakpoint_region'
exons$Osa$scrambled[exons$Osa %over% unal$Osa_Oki[unal$Osa_Oki$nonCoa]] <- 'overlaps_breakpoint_region'
exons$Osa$scrambled |> table()
```

```
## 
##       no_breakpoint_region overlaps_breakpoint_region 
##                      96290                      10615
```

```
exons$Bar$scrambled <- 'no_breakpoint_region'
exons$Bar$scrambled[exons$Bar %over% unal$Bar_Oki[unal$Bar_Oki$nonCoa]] <- 'overlaps_breakpoint_region'
exons$Bar$scrambled |> table()
```

```
## 
##       no_breakpoint_region overlaps_breakpoint_region 
##                      89412                      10498
```

```
oki_feat_bp_ovl <- rbind(
  operons$Oki$scrambled |> table(),
  genes$Oki$scrambled |> table(),
  exons$Oki$scrambled |> table()
) |> as.data.frame(row.names = c('operons', 'genes', 'exons'))
oki_feat_bp_ovl$pct <- oki_feat_bp_ovl[,2]/(oki_feat_bp_ovl[,1]+oki_feat_bp_ovl[,2])*100
oki_feat_bp_ovl$type <- rownames(oki_feat_bp_ovl)
oki_feat_bp_ovl$species <- "Okinawa"

osa_feat_bp_ovl <- rbind(
  operons$Osa$scrambled |> table(),
  genes$Osa$scrambled |> table(),
  exons$Osa$scrambled |> table()
) |> as.data.frame(row.names = c('operons', 'genes', 'exons'))
osa_feat_bp_ovl$pct <- osa_feat_bp_ovl[,2]/(osa_feat_bp_ovl[,1]+osa_feat_bp_ovl[,2])*100
osa_feat_bp_ovl$type <- rownames(osa_feat_bp_ovl)
osa_feat_bp_ovl$species <- "Osaka"

bar_feat_bp_ovl <- rbind(
  operons$Bar$scrambled |> table(),
  genes$Bar$scrambled |> table(),
  exons$Bar$scrambled |> table()
) |> as.data.frame(row.names = c('operons', 'genes', 'exons'))
bar_feat_bp_ovl$pct <- bar_feat_bp_ovl[,2]/(bar_feat_bp_ovl[,1]+bar_feat_bp_ovl[,2])*100
bar_feat_bp_ovl$type <- rownames(bar_feat_bp_ovl)
bar_feat_bp_ovl$species <- "Barcelona"

df <- operons$Oki
names(df) <- NULL
oki_feat_bp_ovl_join <- rbind(
  as.data.frame(df)        |> dplyr::mutate(type='operon') |> dplyr::select(seqnames, start, end, scrambled, type),
  as.data.frame(genes$Oki) |> dplyr::mutate(type='gene')   |> dplyr::select(seqnames, start, end, scrambled, type),
  as.data.frame(exons$Oki) |> dplyr::mutate(type='exon')   |> dplyr::select(seqnames, start, end, scrambled, type)
) |> dplyr::mutate(species='Oki')

df <- operons$Osa
names(df) <- NULL
osa_feat_bp_ovl_join <- rbind(
  as.data.frame(df)        |> dplyr::mutate(type='operon') |> dplyr::select(seqnames, start, end, scrambled, type),
  as.data.frame(genes$Osa) |> dplyr::mutate(type='gene')   |> dplyr::select(seqnames, start, end, scrambled, type),
  as.data.frame(exons$Osa) |> dplyr::mutate(type='exon')   |> dplyr::select(seqnames, start, end, scrambled, type)
) |> dplyr::mutate(species='Osa')

p1 <- ggplot(oki_feat_bp_ovl) + aes(x=type, y=pct, fill=type) + geom_bar(stat='identity') + ylab('percent overlapping breakpoint region') + ggtitle('Okinawa feature plot') + ylim(c(0, 50)) + theme_bw()

p2 <- ggplot(osa_feat_bp_ovl) + aes(x=type, y=pct, fill=type) + geom_bar(stat='identity') + ylab('percent overlapping breakpoint region') + ggtitle('Osaka feature plot') + ylim(c(0, 50)) + theme_bw()

p3 <- ggplot(bar_feat_bp_ovl) + aes(x=type, y=pct, fill=type) + geom_bar(stat='identity') + ylab('percent overlapping breakpoint region') + ggtitle('Barcelona feature plot') + ylim(c(0, 50)) + theme_bw()

( p1 | p2 | p3)
```

```
rbind(oki_feat_bp_ovl, osa_feat_bp_ovl, bar_feat_bp_ovl) |>
  ggplot() +
  aes(type, pct, fill = species) +
  geom_bar(stat = "identity", position="dodge")  +
  scale_y_continuous("Percent features overlaping breakpoint regions") +
  scale_x_discrete("Feature type") +
  theme_bw()
```

## Session information

```
sessionInfo()
```

```
## R version 4.3.1 (2023-06-16)
## Platform: x86_64-pc-linux-gnu (64-bit)
## Running under: Debian GNU/Linux 12 (bookworm)
## 
## Matrix products: default
## BLAS:   /usr/lib/x86_64-linux-gnu/blas/libblas.so.3.11.0 
## LAPACK: /usr/lib/x86_64-linux-gnu/lapack/liblapack.so.3.11.0
## 
## locale:
##  [1] LC_CTYPE=en_US.UTF-8       LC_NUMERIC=C              
##  [3] LC_TIME=en_US.UTF-8        LC_COLLATE=en_US.UTF-8    
##  [5] LC_MONETARY=en_US.UTF-8    LC_MESSAGES=en_US.UTF-8   
##  [7] LC_PAPER=en_US.UTF-8       LC_NAME=C                 
##  [9] LC_ADDRESS=C               LC_TELEPHONE=C            
## [11] LC_MEASUREMENT=en_US.UTF-8 LC_IDENTIFICATION=C       
## 
## time zone: Etc/UTC
## tzcode source: system (glibc)
## 
## attached base packages:
## [1] stats4    stats     graphics  grDevices utils     datasets  methods  
## [8] base     
## 
## other attached packages:
##  [1] BSgenome.Oidioi.genoscope.OdB3_1.0.0   
##  [2] BSgenome.Oidioi.OIST.AOM.5.5f_1.0.1    
##  [3] BSgenome.Oidioi.OIST.KUM.M3.7f_1.0.1   
##  [4] BSgenome.Oidioi.OIST.Bar2.p4_1.0.1     
##  [5] BSgenome.Oidioi.OIST.OSKA2016v1.9_1.0.0
##  [6] BSgenome.Oidioi.OIST.OKI2018.I69_1.0.1 
##  [7] patchwork_1.1.2                        
##  [8] heatmaps_1.24.0                        
##  [9] GenomicFeatures_1.52.1                 
## [10] AnnotationDbi_1.62.2                   
## [11] Biobase_2.60.0                         
## [12] OikScrambling_5.1.0                    
## [13] ggplot2_3.4.4                          
## [14] GenomicBreaks_0.14.3                   
## [15] BSgenome_1.68.0                        
## [16] rtracklayer_1.60.1                     
## [17] Biostrings_2.68.1                      
## [18] XVector_0.40.0                         
## [19] GenomicRanges_1.52.1                   
## [20] GenomeInfoDb_1.36.4                    
## [21] IRanges_2.34.1                         
## [22] S4Vectors_0.38.2                       
## [23] BiocGenerics_0.46.0                    
## 
## loaded via a namespace (and not attached):
##   [1] splines_4.3.1               BiocIO_1.10.0              
##   [3] filelock_1.0.2              bitops_1.0-7               
##   [5] tibble_3.2.1                R.oo_1.25.0                
##   [7] XML_3.99-0.15               rpart_4.1.19               
##   [9] rGADEM_2.48.0               lifecycle_1.0.3            
##  [11] rprojroot_2.0.4             lattice_0.20-45            
##  [13] MASS_7.3-58.2               backports_1.4.1            
##  [15] magrittr_2.0.3              Hmisc_5.1-1                
##  [17] sass_0.4.7                  rmarkdown_2.25             
##  [19] jquerylib_0.1.4             yaml_2.3.7                 
##  [21] plotrix_3.8-3               DBI_1.1.3                  
##  [23] CNEr_1.36.0                 minqa_1.2.6                
##  [25] RColorBrewer_1.1-3          ade4_1.7-22                
##  [27] abind_1.4-5                 zlibbioc_1.46.0            
##  [29] purrr_1.0.2                 R.utils_2.12.2             
##  [31] RCurl_1.98-1.13             nnet_7.3-18                
##  [33] pracma_2.4.2                rappdirs_0.3.3             
##  [35] GenomeInfoDbData_1.2.10     seqLogo_1.66.0             
##  [37] gdata_3.0.0                 annotate_1.78.0            
##  [39] pkgdown_2.0.7               codetools_0.2-19           
##  [41] DelayedArray_0.26.7         xml2_1.3.5                 
##  [43] tidyselect_1.2.0            shape_1.4.6                
##  [45] ggseqlogo_0.1               farver_2.1.1               
##  [47] lme4_1.1-35.1               BiocFileCache_2.8.0        
##  [49] matrixStats_1.0.0           base64enc_0.1-3            
##  [51] GenomicAlignments_1.36.0    jsonlite_1.8.7             
##  [53] mitml_0.4-5                 Formula_1.2-5              
##  [55] survival_3.5-3              iterators_1.0.14           
##  [57] systemfonts_1.0.5           foreach_1.5.2              
##  [59] progress_1.2.2              tools_4.3.1                
##  [61] ragg_1.2.5                  Rcpp_1.0.11                
##  [63] glue_1.6.2                  gridExtra_2.3              
##  [65] pan_1.9                     xfun_0.41                  
##  [67] MatrixGenerics_1.12.3       EBImage_4.42.0             
##  [69] dplyr_1.1.3                 withr_2.5.2                
##  [71] fastmap_1.1.1               boot_1.3-28.1              
##  [73] fansi_1.0.5                 digest_0.6.33              
##  [75] R6_2.5.1                    mice_3.16.0                
##  [77] textshaping_0.3.7           colorspace_2.1-0           
##  [79] GO.db_3.17.0                gtools_3.9.4               
##  [81] poweRlaw_0.70.6             jpeg_0.1-10                
##  [83] biomaRt_2.56.1              RSQLite_2.3.3              
##  [85] weights_1.0.4               R.methodsS3_1.8.2          
##  [87] utf8_1.2.4                  tidyr_1.3.0                
##  [89] generics_0.1.3              data.table_1.14.8          
##  [91] prettyunits_1.2.0           httr_1.4.7                 
##  [93] htmlwidgets_1.6.2           S4Arrays_1.0.6             
##  [95] pkgconfig_2.0.3             gtable_0.3.4               
##  [97] blob_1.2.4                  htmltools_0.5.7            
##  [99] fftwtools_0.9-11            scales_1.2.1               
## [101] png_0.1-8                   knitr_1.45                 
## [103] rstudioapi_0.15.0           tzdb_0.4.0                 
## [105] reshape2_1.4.4              rjson_0.2.21               
## [107] curl_5.1.0                  checkmate_2.3.0            
## [109] nlme_3.1-162                nloptr_2.0.3               
## [111] cachem_1.0.8                stringr_1.5.0              
## [113] KernSmooth_2.23-20          parallel_4.3.1             
## [115] genoPlotR_0.8.11            foreign_0.8-84             
## [117] restfulr_0.0.15             desc_1.4.2                 
## [119] pillar_1.9.0                grid_4.3.1                 
## [121] vctrs_0.6.4                 jomo_2.7-6                 
## [123] dbplyr_2.3.3                xtable_1.8-4               
## [125] cluster_2.1.4               htmlTable_2.4.2            
## [127] evaluate_0.23               readr_2.1.4                
## [129] cli_3.6.1                   locfit_1.5-9.8             
## [131] compiler_4.3.1              Rsamtools_2.16.0           
## [133] rlang_1.1.2                 crayon_1.5.2               
## [135] labeling_0.4.3              plyr_1.8.9                 
## [137] fs_1.6.3                    stringi_1.7.12             
## [139] BiocParallel_1.34.2         munsell_0.5.0              
## [141] tiff_0.1-11                 glmnet_4.1-8               
## [143] Matrix_1.5-3                hms_1.1.3                  
## [145] bit64_4.0.5                 KEGGREST_1.40.1            
## [147] highr_0.10                  SummarizedExperiment_1.30.2
## [149] broom_1.0.5                 memoise_2.0.1              
## [151] bslib_0.5.1                 bit_4.0.5
```

## Contents

Developed by Aleksandra Bliznina, Michael Mansfield, Charles Plessy.

Site built with pkgdown 2.0.7.
