# Supplementary material for: Extreme genome scrambling in marine planktonic Oikopleura dioica cryptic species
Source: Genome Res. 2024 Mar;34(3):426–40. doi: 10.1101/gr.278295.123 (PMC11067885; doi:10.1101/gr.278295.123)
Supplement: Supplement 4 [file Supplemental_Data.zip › Supplemental_Data.tar.xz!/Supplemental_Data/articles/ChromosomePlots_files/figure-html/chromosome_plots_tiles_5_2_Fig4B-1.pdf]

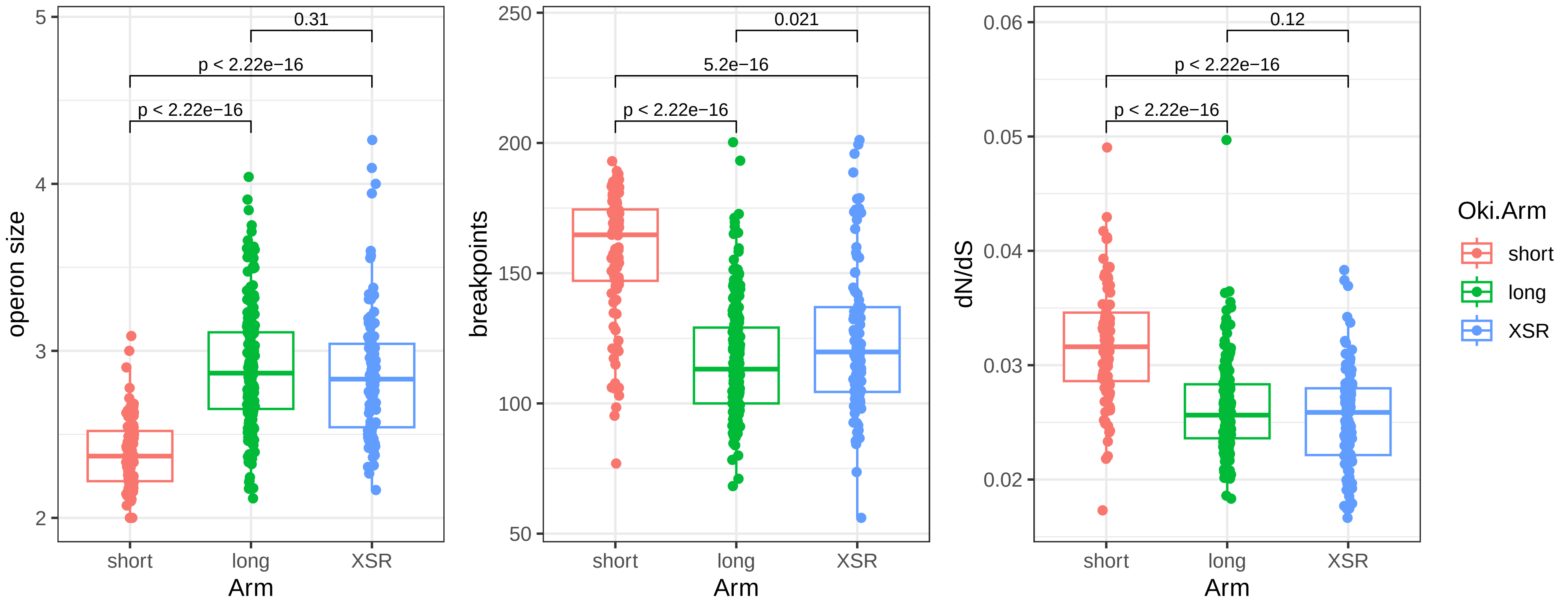

operon size
0.31
p < 2.22e−16
p < 2.22e−16
5
4
3
2
short
long
XSR
Arm
breakpoints
0.021
5.2e−16
p < 2.22e−16
250
200
150
100
50
short
long
XSR
Arm
dN/dS
0.12
p < 2.22e−16
p < 2.22e−16
0.06
0.05
0.04
0.03
0.02
short
long
XSR
Arm
Oki.Arm
short
long
XSR
