# Supplementary material for: Extreme genome scrambling in marine planktonic Oikopleura dioica cryptic species
Source: Genome Res. 2024 Mar;34(3):426–40. doi: 10.1101/gr.278295.123 (PMC11067885; doi:10.1101/gr.278295.123)
Supplement: Supplement 4 [file Supplemental_Data.zip › Supplemental_Data.tar.xz!/Supplemental_Data/articles/ChromosomePlots_files/figure-html/chromosome_plots_tiles_5_2_Fig4A-1.pdf]

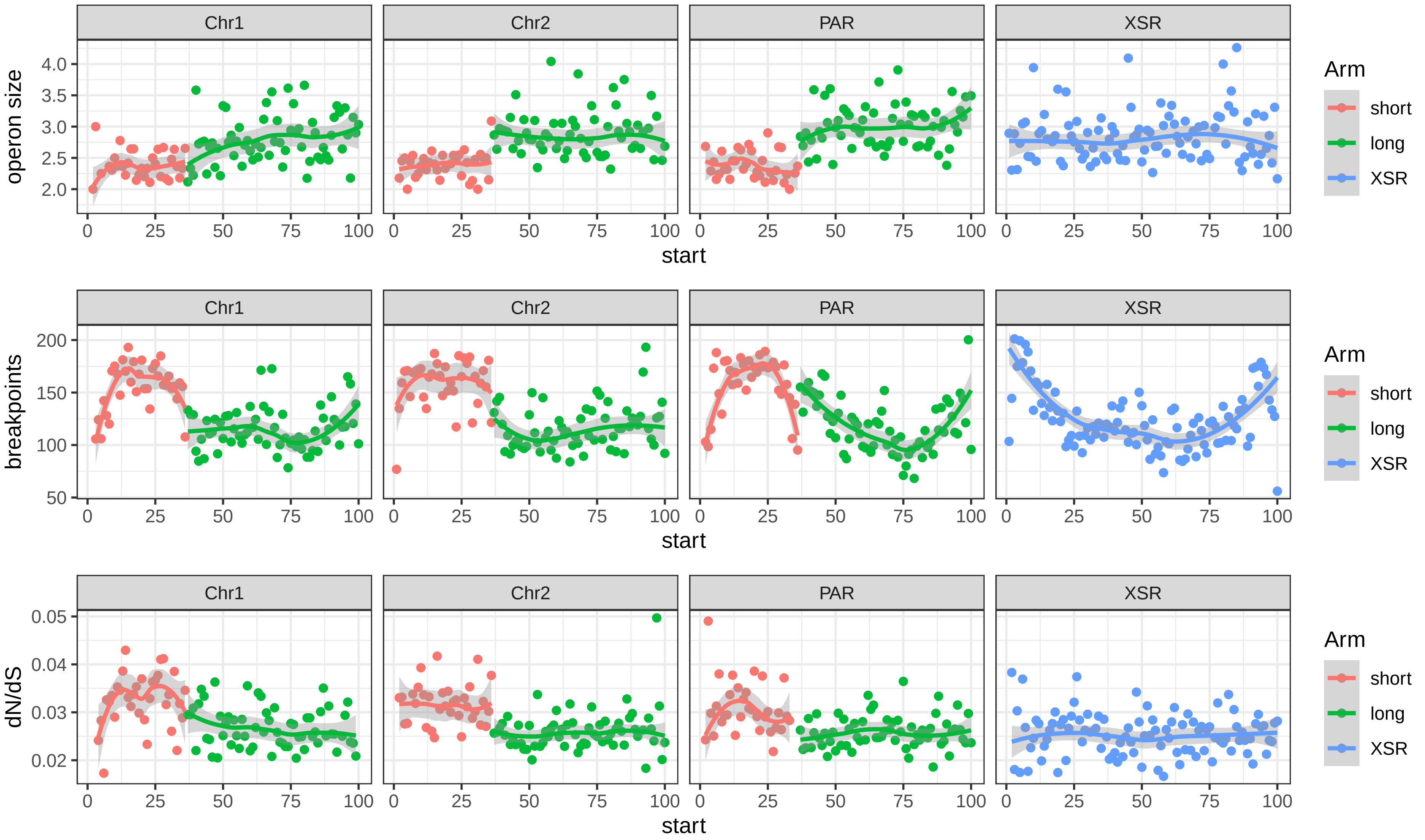

Chr1
Chr2
PAR
XSR
operon size
breakpoints
dN/dS
start
Arm
short
long
XSR
