# Supplementary material for: Extreme genome scrambling in marine planktonic Oikopleura dioica cryptic species
Source: Genome Res. 2024 Mar;34(3):426–40. doi: 10.1101/gr.278295.123 (PMC11067885; doi:10.1101/gr.278295.123)
Supplement: Supplement 4 [file Supplemental_Data.zip › Supplemental_Data.tar.xz!/Supplemental_Data/articles/ScalingBreakpoints_files/figure-html/breakpoints_2-6.pdf]

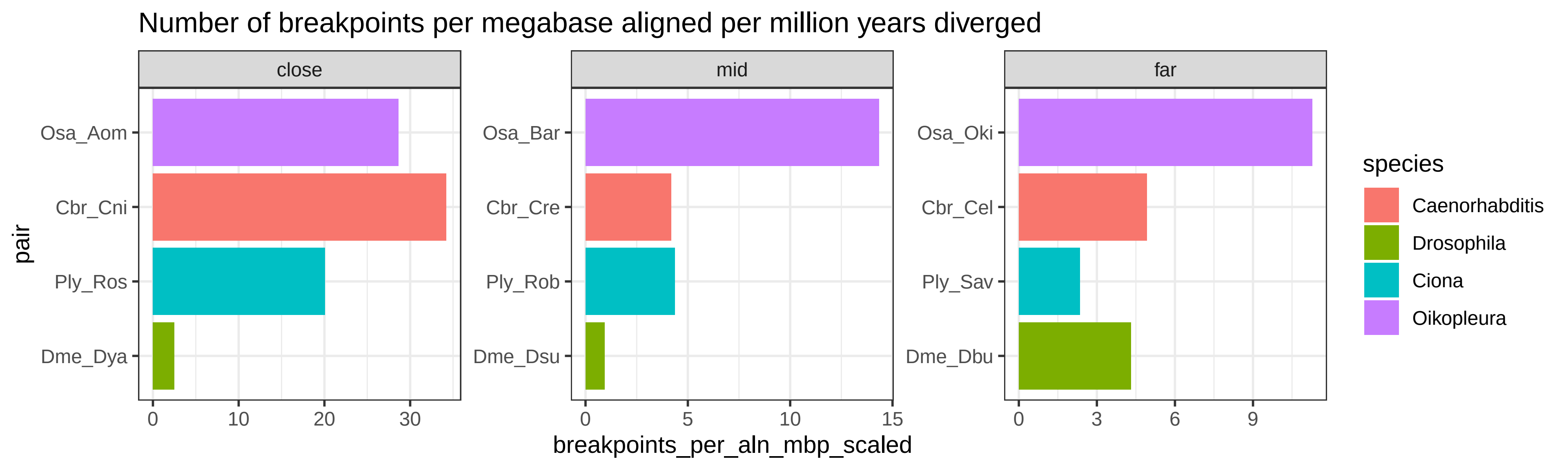

Number of breakpoints per megabase aligned per million years diverged
close
mid
far
pair
Osa_Aom
Cbr_Cni
Ply_Ros
Dme_Dya
Osa_Bar
Cbr_Cre
Ply_Rob
Dme_Dsu
Osa_Oki
Cbr_Cel
Ply_Sav
Dme_Dbu
0
10
20
30
0
5
10
15
0
3
6
9
breakpoints_per_aln_mbp_scaled
species
Caenorhabditis
Drosophila
Ciona
Oikopleura
