# Supplementary material for: Extreme genome scrambling in marine planktonic Oikopleura dioica cryptic species
Source: Genome Res. 2024 Mar;34(3):426–40. doi: 10.1101/gr.278295.123 (PMC11067885; doi:10.1101/gr.278295.123)
Supplement: Supplement 4 [file Supplemental_Data.zip › Supplemental_Data.tar.xz!/Supplemental_Data/articles/ScalingBreakpoints_files/figure-html/breakpoints_2-1.pdf]

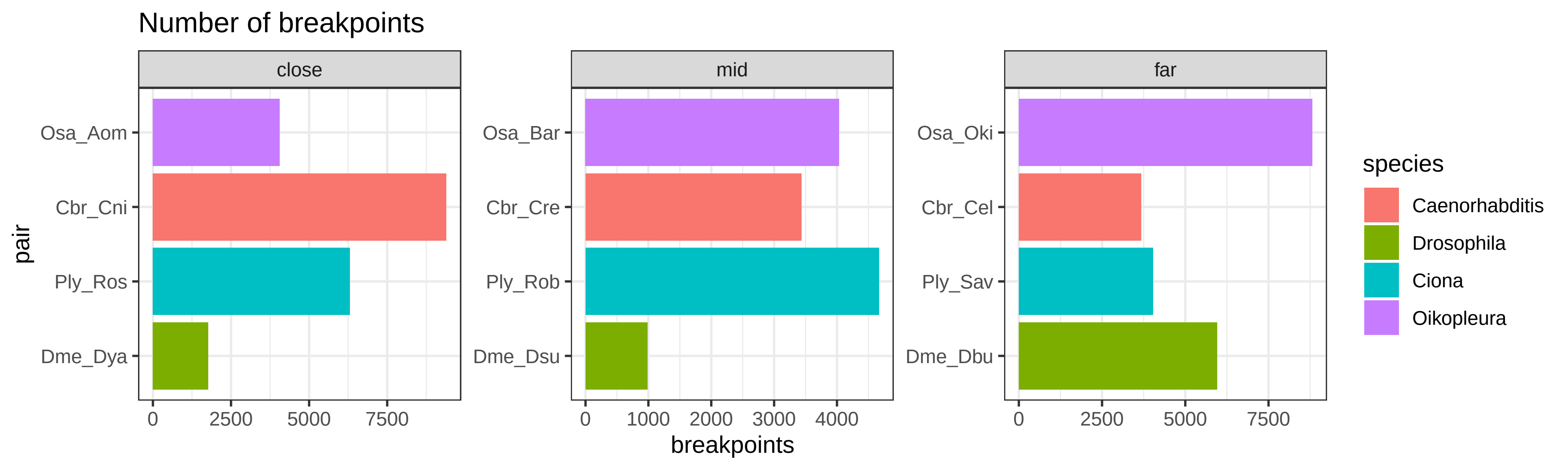

Number of breakpoints
close
mid
far
Osa_Aom
Cbr_Cni
Ply_Ros
Dme_Dya
Osa_Bar
Cbr_Cre
Ply_Rob
Dme_Dsu
Osa_Oki
Cbr_Cel
Ply_Sav
Dme_Dbu
pair
0
2500
5000
7500
0
1000
2000
3000
4000
0
2500
5000
7500
breakpoints
species
Caenorhabditis
Drosophila
Ciona
Oikopleura
