# Supplementary material for: Extreme genome scrambling in marine planktonic Oikopleura dioica cryptic species
Source: Genome Res. 2024 Mar;34(3):426–40. doi: 10.1101/gr.278295.123 (PMC11067885; doi:10.1101/gr.278295.123)
Supplement: Supplement 4 [file Supplemental_Data.zip › Supplemental_Data.tar.xz!/Supplemental_Data/articles/ScalingBreakpoints_files/figure-html/breakpoints_2-9.pdf]

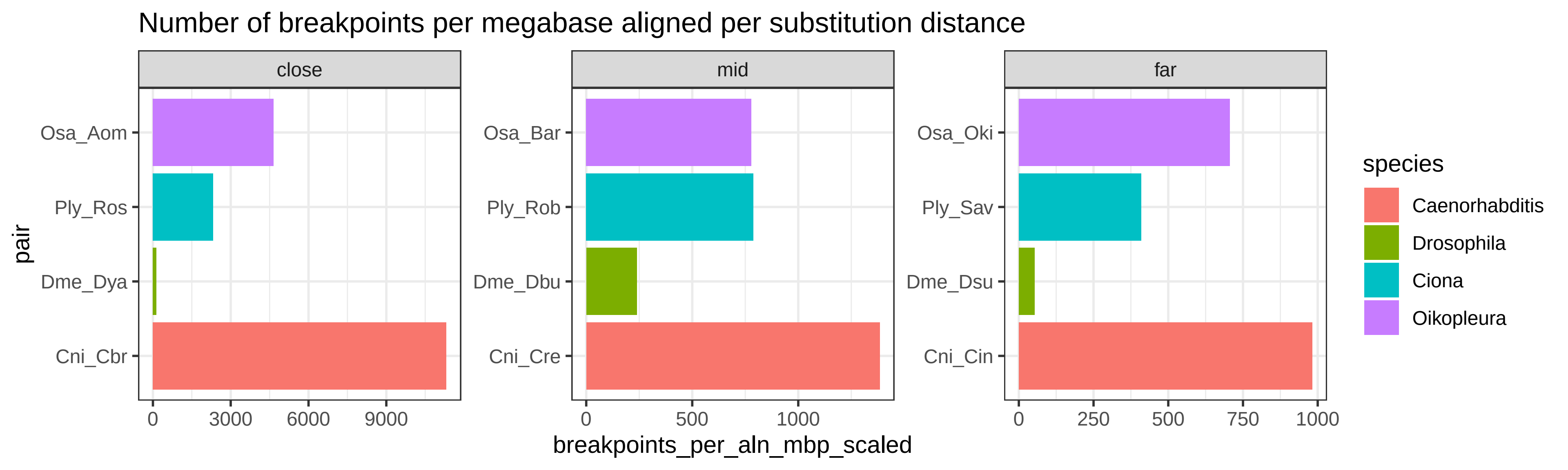
Number of breakpoints per megabase aligned per substitution distance
close
mid
far
pair
Osa_Aom
Ply_Ros
Dme_Dya
Cni_Cbr
Osa_Bar
Ply_Rob
Dme_Dbu
Cni_Cre
Osa_Oki
Ply_Sav
Dme_Dsu
Cni_Cin
0
3000
6000
9000
0
500
1000
0
250
500
750
1000
breakpoints_per_aln_mbp_scaled
species
Caenorhabditis
Drosophila
Ciona
Oikopleura
