# Supplementary material for: Extreme genome scrambling in marine planktonic Oikopleura dioica cryptic species
Source: Genome Res. 2024 Mar;34(3):426–40. doi: 10.1101/gr.278295.123 (PMC11067885; doi:10.1101/gr.278295.123)
Supplement: Supplement 4 [file Supplemental_Data.zip › Supplemental_Data.tar.xz!/Supplemental_Data/articles/OrthogroupSyntenies.html]

Orthogroup syntenies • OikScrambling
 


  


 


Toggle navigation


OikScrambling
5.1.0

- Get started
- Data load
- Supplemental material
  - Figure 1B DNA line plots
  - Figure 1C chromosome parallel plots
  - Figure 1D scrambling index
  - Figure 2A orthogroup dot plots and synteny block histograms
  - Figure 5 genome-wide feature patterns
  - Figure 7A–D DNA line plots
  - Figure 7E breakpoint number barplots
- Articles
  - Scaffolding of the AOM-5 genome
  - Scaffolding of the OdB3 genome
  - Conserved non-coding elements
  - Chromosome-scale feature density plots
  - Modifying coalescent cutoffs
  - Regions interrupting colinearity
  - Distance Measures
  - Genomic features of the breakpoints
  - Genomic features of the breakpoints (heatmaps)
  - Inversions
  - Scaffolding of the KUM-M3 genome
  - Load genomic break data
  - Operon conservation analysis
  - Orthogroup syntenies
  - Alignment line plots, whole genomes
  - Region of interest: PAC3
  - Motif search and analysis
  - Whole-chromosome parallel plots
  - Width of the aligned and mapped regions.
  - Regions of interest
  - Repeat regions
  - Scaling breakpoints
  - Segment movement distances
  - Alignment line plots, 10×10 Mbp windows
  - Transcription factor binding sites
  - Tandem site duplications
  - Translocations
- Changelog
- Reference

# Orthogroup syntenies

Source: `vignettes/OrthogroupSyntenies.Rmd`

`OrthogroupSyntenies.Rmd`

```
knitr::opts_knit$set(cache = TRUE)
```

## Introduction

Gene-centric synteny analyses.

Figure 2 panel A and Supplemental figure 2 were generated in this
vignette.

The vignette also contains extra material that is not entirely
relevant to the manuscript, but that can not be moved elsewhere because
it needs the orthogroup `GBreaks` object generated at the
beginning at the top.

## Load packages and data

See `?OikScrambling:::loadAllGenomes()`,
`?OikScrambling:::loadAllTranscriptsGR()`, and
`vignette("LoadGenomicBreaks", package = "OikScrambling")`
for how the different objects are prepared.

```
library('OikScrambling')   |> suppressPackageStartupMessages()
library('patchwork')       |> suppressPackageStartupMessages()
genomes <- OikScrambling:::loadAllGenomes(compat = F)
```

```
## Warning in runHook(".onLoad", env, package.lib, package): input string
## 'Génoscope' cannot be translated from 'ANSI_X3.4-1968' to UTF-8, but is valid
## UTF-8

## Warning in runHook(".onLoad", env, package.lib, package): input string
## 'Génoscope' cannot be translated from 'ANSI_X3.4-1968' to UTF-8, but is valid
## UTF-8
```

```
transcripts <- OikScrambling:::loadAllTranscriptsGR(compat = F) |> suppressWarnings()
ggplot2::theme_set(theme_bw())

load("BreakPoints.Rdata")
```

## Prepare GBreaks objects

Orthogroups for *Oikopleura* are loaded from the
`BreakpointsData` package with the
`OikScrambling::load_one_to_ones()` function. Original file
is in
`/bucket/LuscombeU/common/Breakpoints/Orthologues/selTun+Apps+Amph+Vert_lp_100clstr_blast/OrthoFinder/Results_Oct07/Phylogenetic_Hierarchical_Orthogroups/N19.tsv`.
See `news(package = "BreakpointsData")` for inspecting
changes in more details.

### Load Orthogroup data

This produces `GBreaks` objects that map orthologs from
between two genomes. In these objects, the *target* and
*query* roles are equivalent. The genomic coordinates are taken
from the annotation files (see
`?OikScrambling:::loadAllTranscriptsGR`.

#### Orthogroup pairs

To maximise number of entries, we load data from the larvacean and
the ascidian subtrees respectively. This produces the
`orthoPairs` collection of objects.

```
orthoGroupFileNames <- SimpleList(
  AOM.5.5f      = "AOM-5-5f.prot.longest.fa_1", 
  Bar2.p4       = "Bar2_p4.Flye.prot.longest.fa_1", 
  Ply           = "C_int_P.prot.longest.fa_1",
  Ros           = "C_int_R.prot.longest.fa_1",
  Int           = "Ciona_intestinalis.Uniprot.rn.fa_1",
  Sav           = "Ciona_savignyi.Uniprot.rn.fa_1",
  KUM.M3.7f     = "KUM-M3-7f.prot.longest.fa_1",
  OKI2018.I69   = "OKI2018_I69.v2.prot.longest.fa_1",
  OSKA2016v1.9  = "OSKA2016v1.9.prot.longest.fa_1",
  OdB3          = "OdB3.v1.0.prot.fa_1.nohaplo"
)

orthoPairToGBreaks <- function(genome1, genome2, transcripts, treeName="N19", HOGs=NULL) {
  if(is.null(HOGs))
    HOGs <- OikScrambling:::load_one_to_ones(
      system.file(paste0("extdata/OrthoFinder/",treeName,".tsv"), package = "BreakpointsData"),
      c(orthoGroupFileNames[[genome1]], orthoGroupFileNames[[genome2]]))

  IDs2GRanges <- function (IDs, annot) {
    prefix <- Biobase::lcPrefix(IDs)                  # Guess prefix in transcript IDs from HOG files
    not_prefix <- Biobase::lcPrefix(annot$tx_name)    # Remove trailing characters that are part of the name
    not_prefix <- paste0(not_prefix,"$")              # Anchor to end of the string
    prefix <- sub(not_prefix, "", prefix)             # Finalise prefix
    IDs <- sub(prefix, "", IDs)                       # Remove prefix from IDS
    names(annot) <- annot$tx_name                     # Store transcript name in names slot
    gr <- annot[IDs]                                  # Sort by ID
    strand(gr) <- "*"                                 # Make strandless
    gr                                                # Return the object
  }
  
  gb       <- IDs2GRanges(HOGs[,orthoGroupFileNames[[genome1]]], transcripts[[genome1]])
  if (!is.null(genomes[[genome1]]))
    seqinfo(gb) <- seqinfo(genomes[[genome1]])
  gb$query <- IDs2GRanges(HOGs[,orthoGroupFileNames[[genome2]]], transcripts[[genome2]])
  if (!is.null(genomes[[genome2]]))
   seqinfo(gb$query) <- seqinfo(genomes[[genome2]])
  gb <- GenomicBreaks:::GBreaks(gb)
  gb$HOG <- HOGs$HOG
  gb$OG  <- HOGs$OG
  sort(gb)
}

flagLongShort_ <- function(gr, transcripts) {
  genome <- unique(genome(gr))
  flagLongShort(gr, transcripts[[genome]])
}

orthoPairToGBreaks_all_Oiks <- function(treeName=NULL, HOGs=NULL) {
  orthoPairs <- SimpleList()
  orthoPairs$Oki_Osa <- orthoPairToGBreaks("OKI2018.I69", "OSKA2016v1.9",   transcripts, treeName, HOGs)
  orthoPairs$Oki_Bar <- orthoPairToGBreaks("OKI2018.I69", "Bar2.p4",        transcripts, treeName, HOGs)
  orthoPairs$Oki_Kum <- orthoPairToGBreaks("OKI2018.I69", "KUM.M3.7f",      transcripts, treeName, HOGs)
  orthoPairs$Oki_Aom <- orthoPairToGBreaks("OKI2018.I69", "AOM.5.5f",       transcripts, treeName, HOGs)
  orthoPairs$Oki_Nor <- orthoPairToGBreaks("OKI2018.I69", "OdB3",           transcripts, treeName, HOGs)
  
  orthoPairs$Osa_Bar <- orthoPairToGBreaks("OSKA2016v1.9",   "Bar2.p4",     transcripts, treeName, HOGs)
  orthoPairs$Osa_Oki <- orthoPairToGBreaks("OSKA2016v1.9",   "OKI2018.I69", transcripts, treeName, HOGs)
  orthoPairs$Osa_Kum <- orthoPairToGBreaks("OSKA2016v1.9",   "KUM.M3.7f",   transcripts, treeName, HOGs)
  orthoPairs$Osa_Aom <- orthoPairToGBreaks("OSKA2016v1.9",   "AOM.5.5f",    transcripts, treeName, HOGs)
  orthoPairs$Osa_Nor <- orthoPairToGBreaks("OSKA2016v1.9",   "OdB3",        transcripts, treeName, HOGs)
  
  orthoPairs$Bar_Osa <- orthoPairToGBreaks("Bar2.p4",   "OSKA2016v1.9",     transcripts, treeName, HOGs)
  orthoPairs$Bar_Oki <- orthoPairToGBreaks("Bar2.p4",   "OKI2018.I69",      transcripts, treeName, HOGs)
  orthoPairs$Bar_Kum <- orthoPairToGBreaks("Bar2.p4",   "KUM.M3.7f",        transcripts, treeName, HOGs)
  orthoPairs$Bar_Aom <- orthoPairToGBreaks("Bar2.p4",   "AOM.5.5f",         transcripts, treeName, HOGs)
  orthoPairs$Bar_Nor <- orthoPairToGBreaks("Bar2.p4",   "OdB3",             transcripts, treeName, HOGs)
  
  orthoPairs <- sapply(orthoPairs,  flagLongShort_, longShort)
  
  SimpleList(orthoPairs)
}

orthoPairs         <- orthoPairToGBreaks_all_Oiks("N19")
orthoPairs$Ply_Ros <- orthoPairToGBreaks("Ply", "Ros", transcripts, treeName="N20")
```

#### Orthogroup core pairs

The `orthoPairs_core` collection is built similarly, but
from “core” orthogroups, that have a member in each species of the
tunicate clade.

```
orthoPairs_core         <- orthoPairToGBreaks_all_Oiks("N3")
orthoPairs_core$Ply_Ros <- orthoPairToGBreaks("Ply", "Ros", transcripts, treeName="N3")
```

#### Orthogroups one-to-one in oiks.

Lastly we build an object for Oiks only where we require every
orthologue to be present as a single copy in every genome. This is the
`orthoPairs_one2oneInOiks` collection.

```
orthoPairs_one2oneInOiks <-
  orthoPairToGBreaks_all_Oiks(treeName = NULL,
                              HOGs = OikScrambling:::load_one_to_ones(
                                       system.file("extdata/OrthoFinder/N19.tsv", package = "BreakpointsData")))
```

#### Summary

Here is the number of entries in each object.

```
sapply(orthoPairs, length)
```

```
## Oki_Osa Oki_Bar Oki_Kum Oki_Aom Oki_Nor Osa_Bar Osa_Oki Osa_Kum Osa_Aom Osa_Nor 
##    9538    9273   11525    9604    8771    9172    9538    9465   10379    8415 
## Bar_Osa Bar_Oki Bar_Kum Bar_Aom Bar_Nor Ply_Ros 
##    9172    9273    9223    9250    8508    5438
```

```
sapply(orthoPairs_core, length)
```

```
## Oki_Osa Oki_Bar Oki_Kum Oki_Aom Oki_Nor Osa_Bar Osa_Oki Osa_Kum Osa_Aom Osa_Nor 
##    6794    6697    8315    6902    6118    6707    6794    6697    7644    5981 
## Bar_Osa Bar_Oki Bar_Kum Bar_Aom Bar_Nor Ply_Ros 
##    6707    6697    6643    6804    6151    2582
```

```
sapply(orthoPairs_one2oneInOiks, length)
```

```
## Oki_Osa Oki_Bar Oki_Kum Oki_Aom Oki_Nor Osa_Bar Osa_Oki Osa_Kum Osa_Aom Osa_Nor 
##    5162    5162    5162    5162    5162    5162    5162    5162    5162    5162 
## Bar_Osa Bar_Oki Bar_Kum Bar_Aom Bar_Nor 
##    5162    5162    5162    5162    5162
```

## Oxford plots

### Versus Osa

```
p1 <- orthoPairs$Oki_Osa |> removeUnplacedContigs() |>
  makeOxfordPlots(type='point', diag = F) + theme_bw() + theme(legend.position='none') +
  scale_color_manual(values = c("#E78AC3", "#A6D854", "#FFD92F", "#8DA0CB", "#66C2A5")) + ggtitle(NULL)
```

```
p2 <- orthoPairs$Bar_Osa |> removeUnplacedContigs() |>
  makeOxfordPlots(type='point', diag=F) + theme_bw() + theme(legend.position='none') +
  scale_color_manual(values = c("#E78AC3", "#A6D854", "#FFD92F", "#8DA0CB", "#66C2A5")) + ggtitle(NULL)
```

```
p3 <- orthoPairs$Osa_Aom |> swap() |>
  scaffoldByFlipAndMerge(scafs$Aom_Osa, drop = TRUE) |> removeUnplacedContigs() |>
  makeOxfordPlots(type='point', diag=F) +
    theme_bw() +
    theme(legend.position='none') + ggtitle(NULL) +
    scale_color_manual(values = c("#E78AC3", "#A6D854", "#FFD92F", "#8DA0CB", "#66C2A5"))
```

```
(p1 + p2 + p3)
```

### Versus Oki

```
p4 <- orthoPairs$Bar_Oki |> removeUnplacedContigs() |>
   makeOxfordPlots(type='point', diag = F) + theme_bw() + theme(legend.position='none') +
   scale_color_manual(values = c("#E78AC3", "#A6D854", "#FFD92F", "#8DA0CB", "#66C2A5")) + ggtitle(NULL)
```

```
p5 <- orthoPairs$Oki_Kum |> swap() |> scaffoldByFlipAndMerge(scafs$Kum_Oki, drop = TRUE) |>
    removeUnplacedContigs() |> 
    makeOxfordPlots(type='point', diag=T) + theme_bw() + theme(legend.position='none') +
   scale_color_manual(values = c("#E78AC3", "#A6D854", "#FFD92F", "#8DA0CB", "#66C2A5")) + ggtitle(NULL)
```

```
p6 <- orthoPairs$Bar_Nor |>
    removeUnplacedContigs(query = FALSE) |> 
    makeOxfordPlots(type='point', diag=T) + theme_bw() + theme(legend.position='none') +
   scale_color_manual(values = c("#E78AC3", "#A6D854", "#FFD92F", "#8DA0CB", "#66C2A5")) + ggtitle(NULL)
```

```
(p4 + p5 + p6)
```

## Collinear regions

### Remove overlapping annotations

Some transcript annotations overlap and this breaks the coalescing
algorithm.

Here we produce non-overlapping versions of the objects.

```
transcripts$Bar2.p4 |> length()
```

```
## [1] 15741
```

```
transcripts$Bar2.p4 |> reduce(min = 0) |> length()
```

```
## [1] 14262
```

```
flagOverlaps <- function(gr) {
  # Find overlaps
  ov <- findOverlaps(gr)
  # Index of self hits in the gr object
  idx <- queryHits(ov[!isSelfHit(ov)])
  # Convert to Boolean indexing the gr object
  seq_along(gr) %in% idx
}

transcripts$Bar2.p4[flagOverlaps(transcripts$Bar2.p4)]
```

```
## GRanges object with 2668 ranges and 5 metadata columns:
##             seqnames          ranges strand |     tx_id     tx_name
##                <Rle>       <IRanges>  <Rle> | <integer> <character>
##     g107.t2     Chr1   495446-496292      + |        58     g107.t2
##     g107.t1     Chr1   495446-496688      + |        59     g107.t1
##     g138.t1     Chr1   687958-689814      + |        76     g138.t1
##     g138.t2     Chr1   687958-689814      + |        77     g138.t2
##     g147.t2     Chr1   748328-751932      + |        82     g147.t2
##         ...      ...             ...    ... .       ...         ...
##   g13975.t2      YSR 4098404-4105602      - |     15657   g13975.t2
##   g14058.t1      YSR 4778101-4778538      - |     15701   g14058.t1
##   g14059.t1      YSR 4778480-4782151      - |     15702   g14059.t1
##   g14064.t2      YSR 4817799-4819944      - |     15705   g14064.t2
##   g14064.t1      YSR 4817799-4820671      - |     15706   g14064.t1
##             dNdS_GUIDANCE2 dNdS_HmmCleaner dNdS_PRANK
##                  <numeric>       <numeric>  <numeric>
##     g107.t2             NA              NA         NA
##     g107.t1             NA              NA         NA
##     g138.t1             NA              NA         NA
##     g138.t2             NA              NA    0.06608
##     g147.t2             NA              NA         NA
##         ...            ...             ...        ...
##   g13975.t2             NA              NA         NA
##   g14058.t1             NA              NA         NA
##   g14059.t1             NA              NA         NA
##   g14064.t2             NA              NA         NA
##   g14064.t1             NA              NA         NA
##   -------
##   seqinfo: 68 sequences from Bar2.p4 genome
```

```
# Show examples
orthoPairs$Oki_Osa[flagOverlaps(orthoPairs$Oki_Osa)]
```

```
## GBreaks object with 93 ranges and 9 metadata columns:
##             seqnames            ranges strand |     tx_id     tx_name
##                <Rle>         <IRanges>  <Rle> | <integer> <character>
##     g499.t1     chr1   2110061-2117154      * |       272     g499.t1
##     g500.t1     chr1   2115711-2116788      * |      2381     g500.t1
##     g544.t1     chr1   2238352-2256657      * |       297     g544.t1
##     g545.t1     chr1   2251048-2252855      * |      2402     g545.t1
##     g985.t1     chr1   3781719-3784744      * |       518     g985.t1
##         ...      ...               ...    ... .       ...         ...
##   g15884.t1      XSR   8785428-8786009      * |     15900   g15884.t1
##   g16057.t1      XSR   9342752-9345613      * |     18123   g16057.t1
##   g16058.t1      XSR   9344384-9344779      * |     15985   g16058.t1
##   g16592.t1      XSR 10958372-10959713      * |     18454   g16592.t1
##   g16593.t1      XSR 10958914-10959511      * |     16264   g16593.t1
##             dNdS_GUIDANCE2 dNdS_HmmCleaner dNdS_PRANK                 query
##                  <numeric>       <numeric>  <numeric>             <GRanges>
##     g499.t1             NA              NA    0.05102    Chr1:717839-724867
##     g500.t1             NA              NA         NA    Chr1:718410-719881
##     g544.t1             NA              NA         NA  Chr1:1777743-1781983
##     g545.t1             NA              NA         NA  Chr1:1783570-1784116
##     g985.t1             NA              NA         NA  Chr1:2597661-2601166
##         ...            ...             ...        ...                   ...
##   g15884.t1             NA              NA    0.40892   XSR:1917337-1918103
##   g16057.t1             NA              NA         NA   XSR:5950541-5952986
##   g16058.t1             NA              NA    0.12175   XSR:5950957-5951353
##   g16592.t1             NA              NA         NA XSR:10797233-10798365
##   g16593.t1             NA              NA         NA XSR:10797613-10797896
##                        HOG          OG      Arm
##                <character> <character> <factor>
##     g499.t1 N19.HOG0002425   OG0000263    short
##     g500.t1 N19.HOG0007945   OG0003451    short
##     g544.t1 N19.HOG0003237   OG0000486    short
##     g545.t1 N19.HOG0015828   OG0019308    short
##     g985.t1 N19.HOG0012972   OG0011801    short
##         ...            ...         ...      ...
##   g15884.t1 N19.HOG0013867   OG0014177      XSR
##   g16057.t1 N19.HOG0007856   OG0003342      XSR
##   g16058.t1 N19.HOG0013727   OG0013149      XSR
##   g16592.t1 N19.HOG0016102   OG0020123      XSR
##   g16593.t1 N19.HOG0016845   OG0024775      XSR
##   -------
##   seqinfo: 19 sequences from OKI2018.I69 genome
```

```
orthoPairs$Oki_Osa$query[flagOverlaps(orthoPairs$Oki_Osa$query)]
```

```
## GRanges object with 80 ranges and 5 metadata columns:
##             seqnames            ranges strand |     tx_id     tx_name
##                <Rle>         <IRanges>  <Rle> | <integer> <character>
##     g165.t1     Chr1     717839-724867      * |      1760     g165.t1
##     g166.t1     Chr1     718410-719881      * |        84     g166.t1
##     g343.t1     Chr1   1555362-1560721      * |       185     g343.t1
##     g344.t1     Chr1   1555709-1558669      * |      1852     g344.t1
##    g1318.t1     Chr1   5426128-5431903      * |       698    g1318.t1
##         ...      ...               ...    ... .       ...         ...
##   g14741.t1      XSR 10797613-10797896      * |     16354   g14741.t1
##   g13032.t1      XSR   5203787-5206862      * |     13405   g13032.t1
##   g13031.t2      XSR   5199352-5210190      * |     15385   g13031.t2
##   g13679.t1      XSR   7256274-7260299      * |     13797   g13679.t1
##   g13680.t1      XSR   7258070-7259639      * |     15749   g13680.t1
##             dNdS_GUIDANCE2 dNdS_HmmCleaner dNdS_PRANK
##                  <numeric>       <numeric>  <numeric>
##     g165.t1             NA              NA    0.05102
##     g166.t1             NA              NA         NA
##     g343.t1             NA              NA         NA
##     g344.t1             NA              NA    0.07528
##    g1318.t1             NA              NA         NA
##         ...            ...             ...        ...
##   g14741.t1             NA              NA         NA
##   g13032.t1             NA              NA         NA
##   g13031.t2             NA              NA    0.02158
##   g13679.t1             NA              NA         NA
##   g13680.t1             NA              NA         NA
##   -------
##   seqinfo: 483 sequences from OSKA2016v1.9 genome
```

```
# Remove and show how much was removed

orthoPairsFiltered <- sapply(orthoPairs, function(gb) {
  gb <- gb[!flagOverlaps(gb)]
  gb <- gb[!flagOverlaps(gb$query)]
  gb
}) |> SimpleList()

orthoPairsFiltered_core <- sapply(orthoPairs_core, function(gb) {
  gb <- gb[!flagOverlaps(gb)]
  gb <- gb[!flagOverlaps(gb$query)]
  gb
}) |> SimpleList()

orthoPairs_one2oneInOiks_Filtered <- sapply(orthoPairs_one2oneInOiks, function(gb) {
  gb <- gb[!flagOverlaps(gb)]
  gb <- gb[!flagOverlaps(gb$query)]
  gb
}) |> SimpleList()

sapply(orthoPairs, length)               - sapply(orthoPairsFiltered, length)
```

```
## Oki_Osa Oki_Bar Oki_Kum Oki_Aom Oki_Nor Osa_Bar Osa_Oki Osa_Kum Osa_Aom Osa_Nor 
##     132     131     202     166     119      98     132     114     121     103 
## Bar_Osa Bar_Oki Bar_Kum Bar_Aom Bar_Nor Ply_Ros 
##      98     132     119     108     125      26
```

```
sapply(orthoPairs_core, length)          - sapply(orthoPairsFiltered_core, length)
```

```
## Oki_Osa Oki_Bar Oki_Kum Oki_Aom Oki_Nor Osa_Bar Osa_Oki Osa_Kum Osa_Aom Osa_Nor 
##      74      85     125      93      58      72      74      67      75      48 
## Bar_Osa Bar_Oki Bar_Kum Bar_Aom Bar_Nor Ply_Ros 
##      72      85      85      72      64      10
```

```
sapply(orthoPairs_one2oneInOiks, length) - sapply(orthoPairs_one2oneInOiks_Filtered, length)
```

```
## Oki_Osa Oki_Bar Oki_Kum Oki_Aom Oki_Nor Osa_Bar Osa_Oki Osa_Kum Osa_Aom Osa_Nor 
##      18      26      14      18      38      26      18      20      16      44 
## Bar_Osa Bar_Oki Bar_Kum Bar_Aom Bar_Nor 
##      26      26      26      26      52
```

### Coalesce syntenic blocks and annotate them

```
coalOrtho <- function(gb) {
  # Coalesce
  coal <- coalesce_contigs(gb)
  
  # Map annotations to the coalesced blocks
  ov <- findOverlaps(coal, gb)
  
  # Extract gene names mapped to each blocks
  coal$geneNames <- CharacterList(split(names(gb)[subjectHits(ov)], queryHits(ov))) |> unname()
  # We can use the `ov` object for query ranges as well because of the way `coal` was constructed.
  coal$query$geneNames <- CharacterList(split(names(gb$query)[subjectHits(ov)], queryHits(ov))) |> unname()
  # HOG names
  coal$HOGs <- CharacterList(split(gb$HOG[subjectHits(ov)], queryHits(ov))) |> unname()
  
  # Extract dNdS values
  coal$dNdS_GUIDANCE2  <- NumericList(split(gb$dNdS_GUIDANCE2 [subjectHits(ov)], queryHits(ov)))
  coal$dNdS_HmmCleaner <- NumericList(split(gb$dNdS_HmmCleaner[subjectHits(ov)], queryHits(ov)))
  coal$dNdS_PRANK      <- NumericList(split(gb$dNdS_PRANK     [subjectHits(ov)], queryHits(ov)))
  # We just copy dNdS values to query ranges because they are the same.
  coal$query$dNdS_GUIDANCE2  <- coal$dNdS_GUIDANCE2
  coal$query$dNdS_HmmCleaner <- coal$dNdS_HmmCleaner
  coal$query$dNdS_PRANK      <- coal$dNdS_PRANK

  # Count genes per syntenic block
  coal$geneNumber <- sapply(coal$geneNames, length)
  
  coal
}

orthoBlocks <- sapply(orthoPairsFiltered[1:15], coalOrtho) |> SimpleList()
orthoBlocks$Ply_Ros <- coalesce_contigs(orthoPairsFiltered$Ply_Ros)
orthoBlocks[ 1:15 ] <- sapply(orthoBlocks[ 1:15 ],  flagLongShort_, longShort)

orthoBlocks_core <- sapply(orthoPairsFiltered_core[1:15], coalOrtho) |> SimpleList()
orthoBlocks_core$Ply_Ros <- coalesce_contigs(orthoPairsFiltered_core$Ply_Ros)
orthoBlocks_core[ 1:15 ] <- sapply(orthoBlocks_core[ 1:15 ],  flagLongShort_, longShort)

orthoBlocks_one2oneInOiks <- sapply(orthoPairs_one2oneInOiks_Filtered, coalOrtho) |> SimpleList()
orthoBlocks_one2oneInOiks <- sapply(orthoBlocks_one2oneInOiks,  flagLongShort_, longShort) |> SimpleList()

sapply(orthoPairsFiltered, length)
```

```
## Oki_Osa Oki_Bar Oki_Kum Oki_Aom Oki_Nor Osa_Bar Osa_Oki Osa_Kum Osa_Aom Osa_Nor 
##    9406    9142   11323    9438    8652    9074    9406    9351   10258    8312 
## Bar_Osa Bar_Oki Bar_Kum Bar_Aom Bar_Nor Ply_Ros 
##    9074    9141    9104    9142    8383    5412
```

```
sapply(orthoBlocks, length)
```

```
## Oki_Osa Oki_Bar Oki_Kum Oki_Aom Oki_Nor Osa_Bar Osa_Oki Osa_Kum Osa_Aom Osa_Nor 
##    3581    3375     316    3533    3514    1443    3581    3533     455    1806 
## Bar_Osa Bar_Oki Bar_Kum Bar_Aom Bar_Nor Ply_Ros 
##    1443    3373    3333    1418     907    2185
```

```
sapply(orthoPairsFiltered_core, length)
```

```
## Oki_Osa Oki_Bar Oki_Kum Oki_Aom Oki_Nor Osa_Bar Osa_Oki Osa_Kum Osa_Aom Osa_Nor 
##    6720    6612    8190    6809    6060    6635    6720    6630    7569    5933 
## Bar_Osa Bar_Oki Bar_Kum Bar_Aom Bar_Nor Ply_Ros 
##    6635    6612    6558    6732    6087    2572
```

```
sapply(orthoBlocks_core, length)
```

```
## Oki_Osa Oki_Bar Oki_Kum Oki_Aom Oki_Nor Osa_Bar Osa_Oki Osa_Kum Osa_Aom Osa_Nor 
##    2618    2484     226    2598    2496    1119    2618    2548     361    1403 
## Bar_Osa Bar_Oki Bar_Kum Bar_Aom Bar_Nor Ply_Ros 
##    1119    2482    2449    1078     773     664
```

```
sapply(orthoPairs_one2oneInOiks_Filtered, length)
```

```
## Oki_Osa Oki_Bar Oki_Kum Oki_Aom Oki_Nor Osa_Bar Osa_Oki Osa_Kum Osa_Aom Osa_Nor 
##    5144    5136    5148    5144    5124    5136    5144    5142    5146    5118 
## Bar_Osa Bar_Oki Bar_Kum Bar_Aom Bar_Nor 
##    5136    5136    5136    5136    5110
```

```
sapply(orthoBlocks_one2oneInOiks, length)
```

```
## Oki_Osa Oki_Bar Oki_Kum Oki_Aom Oki_Nor Osa_Bar Osa_Oki Osa_Kum Osa_Aom Osa_Nor 
##    2074    2013      93    2058    2227     848    2074    2076     188    1204 
## Bar_Osa Bar_Oki Bar_Kum Bar_Aom Bar_Nor 
##     848    2013    2013     826     601
```

### Compute bridge regions

Let’s pre-compute bridge regions with the
`GenomicBreaks::bridgeRegions()` function.

```
# oP_bridges               <- sapply(orthoPairsFiltered,                bridgeRegions) |> SimpleList()
# oP_core_bridges          <- sapply(orthoPairsFiltered_core,           bridgeRegions) |> SimpleList()
oP_one2oneInOiks_bridges <- sapply(orthoPairs_one2oneInOiks_Filtered, bridgeRegions) |> SimpleList()
```

## Size distribution of the blocks

```
hist(orthoBlocks$Oki_Osa$geneNumber)
```

```
table(orthoBlocks$Oki_Osa$geneNumber)
```

```
## 
##    1    2    3    4    5    6    7    8    9   10   11   12   13   14   15   16 
## 2096  561  244  190   94   84   52   47   37   30   24   16   21   16    9    7 
##   17   18   19   20   21   22   23   24   25   26   27   28   29   31   38   40 
##   11   10    6    4    3    2    2    5    1    1    2    1    1    1    1    1 
##   44 
##    1
```

```
orthoBlocks$Oki_Osa[order(orthoBlocks$Oki_Osa$geneNumber)] |> tail(11)
```

```
## GBreaks object with 11 ranges and 9 metadata columns:
##        seqnames            ranges strand |                 query     score
##           <Rle>         <IRanges>  <Rle> |             <GRanges> <integer>
##    [1]      XSR   7131851-7238581      * |   XSR:3999405-4095337    106731
##    [2]      PAR 15956464-16063993      * | PAR:10902456-10995025    107530
##    [3]      XSR   3436117-3526092      * |   XSR:2172085-2264761     89976
##    [4]      PAR 10906073-11000190      * |   PAR:9275473-9359780     94118
##    [5]      XSR   7246798-7345874      * |   XSR:4104726-4194323     99077
##    [6]      XSR   7562507-7713904      * |   XSR:3625761-3772339    151398
##    [7]      XSR   6441868-6594575      * | XSR:10009542-10156377    152708
##    [8]     chr2 14727816-14844427      * |  Chr2:8441071-8537126    116612
##    [9]     chr2 10668710-10828262      * |  Chr2:5679751-5827304    159553
##   [10]      PAR 15193339-15358002      * | PAR:11170991-11285909    164664
##   [11]      XSR   6597685-6797170      * |  XSR:9796684-10005669    199486
##                                geneNames
##                          <CharacterList>
##    [1] g15372.t1,g15373.t1,g15375.t1,...
##    [2] g12997.t1,g12998.t1,g12999.t1,...
##    [3] g14231.t1,g14232.t1,g14233.t1,...
##    [4] g11569.t1,g11570.t1,g11571.t1,...
##    [5] g15407.t1,g15408.t1,g15410.t1,...
##    [6] g15509.t1,g15511.t1,g15512.t4,...
##    [7] g15154.t1,g15156.t1,g15157.t1,...
##    [8]    g8160.t1,g8161.t1,g8163.t1,...
##    [9]    g6993.t1,g6996.t1,g6997.t2,...
##   [10] g12780.t1,g12781.t1,g12782.t1,...
##   [11] g15204.t1,g15205.t1,g15206.t1,...
##                                                    HOGs dNdS_GUIDANCE2
##                                         <CharacterList>  <NumericList>
##    [1] N19.HOG0013207,N19.HOG0004071,N19.HOG0012356,...   NA,NA,NA,...
##    [2] N19.HOG0004788,N19.HOG0008637,N19.HOG0010302,...   NA,NA,NA,...
##    [3] N19.HOG0005991,N19.HOG0009879,N19.HOG0008774,...   NA,NA,NA,...
##    [4] N19.HOG0000008,N19.HOG0000505,N19.HOG0014173,...   NA,NA,NA,...
##    [5] N19.HOG0003226,N19.HOG0003933,N19.HOG0014771,...   NA,NA,NA,...
##    [6] N19.HOG0002443,N19.HOG0013841,N19.HOG0001471,...   NA,NA,NA,...
##    [7] N19.HOG0013094,N19.HOG0015287,N19.HOG0008355,...   NA,NA,NA,...
##    [8] N19.HOG0007915,N19.HOG0003717,N19.HOG0007729,...   NA,NA,NA,...
##    [9] N19.HOG0005914,N19.HOG0005417,N19.HOG0007559,...   NA,NA,NA,...
##   [10] N19.HOG0009123,N19.HOG0008170,N19.HOG0004489,...   NA,NA,NA,...
##   [11] N19.HOG0004546,N19.HOG0009413,N19.HOG0005226,...   NA,NA,NA,...
##        dNdS_HmmCleaner                  dNdS_PRANK geneNumber      Arm
##          <NumericList>               <NumericList>  <integer> <factor>
##    [1]    NA,NA,NA,...      NA,0.05201,0.07261,...         24     XSR 
##    [2]    NA,NA,NA,... 0.07190,0.05628,     NA,...         25     long
##    [3]    NA,NA,NA,... 0.01959,     NA,     NA,...         26     XSR 
##    [4]    NA,NA,NA,... 0.05944,     NA,     NA,...         27     long
##    [5]    NA,NA,NA,... 0.01057,0.19074,     NA,...         27     XSR 
##    [6]    NA,NA,NA,... 0.08952,0.24384,0.09737,...         28     XSR 
##    [7]    NA,NA,NA,... 0.12848,     NA,0.11163,...         29     XSR 
##    [8]    NA,NA,NA,... 0.03991,0.06627,0.04440,...         31     long
##    [9]    NA,NA,NA,...      NA,     NA,0.04789,...         38     long
##   [10]    NA,NA,NA,...                NA,NA,NA,...         40     long
##   [11]    NA,NA,NA,... 0.05378,0.03146,0.15059,...         44     XSR 
##   -------
##   seqinfo: 19 sequences from OKI2018.I69 genome
```

```
hist(orthoBlocks$Oki_Kum$geneNumber)
```

```
table(orthoBlocks$Oki_Kum$geneNumber)
```

```
## 
##   1   2   3   4   5   6   7   8   9  10  11  12  13  14  15  16  17  18  19  20 
##  76  24  20  15   8   4   8   3   5   4   7   4   3   4   4   2   3   4   4   3 
##  22  23  24  25  26  27  28  29  30  31  32  33  34  36  37  39  40  42  44  47 
##   2   2   2   3   3   2   2   1   2   3   2   1   1   3   1   2   1   3   2   1 
##  48  49  50  51  54  55  56  57  58  61  63  64  65  68  69  70  71  75  76  78 
##   2   1   3   1   3   1   2   2   1   1   1   1   4   2   2   1   1   1   2   1 
##  80  81  83  87  90  92  94  96  97 102 103 104 106 107 114 117 118 141 142 143 
##   1   1   1   1   1   1   2   1   1   2   1   1   1   1   1   1   2   1   1   1 
## 149 161 170 180 197 199 200 264 269 308 356 367 423 438 459 475 
##   1   1   1   1   1   1   1   1   1   1   1   1   1   1   1   1
```

```
orthoBlocks$Oki_Kum[order(orthoBlocks$Oki_Kum$geneNumber)] |> tail(11)
```

```
## GBreaks object with 11 ranges and 9 metadata columns:
##        seqnames            ranges strand |                        query
##           <Rle>         <IRanges>  <Rle> |                    <GRanges>
##    [1]      PAR  9991049-10823433      * |       contig_28_1:906-916604
##    [2]      XSR   1398297-2355032      * |  contig_42_1:1522014-2519191
##    [3]     chr1   6729778-8085886      * |      contig_90_1:571-1399045
##    [4]     chr1  8676861-10061915      * |    contig_3_1:618849-1981910
##    [5]      PAR   7792256-9333620      * |   contig_82_1:846722-2300436
##    [6]      PAR 11974020-13925498      * |  contig_28_1:2026940-3782149
##    [7]      XSR   7904454-9304595      * |  contig_42_1:7880616-9254847
##    [8]     chr2   5748389-7696653      * |    contig_24_1:12160-1868534
##    [9]      XSR   4988262-6642828      * |  contig_42_1:4994508-6631430
##   [10]      XSR  9308304-11123841      * | contig_42_1:9258557-11063801
##   [11]      XSR   2649320-4576212      * |  contig_42_1:2787081-4661476
##            score                         geneNames
##        <integer>                   <CharacterList>
##    [1]    832385 g11259.t1,g11261.t1,g11262.t1,...
##    [2]    956736 g13662.t1,g13663.t1,g13665.t1,...
##    [3]   1356109    g1763.t1,g1772.t1,g1773.t1,...
##    [4]   1385055    g2316.t1,g2317.t1,g2319.t1,...
##    [5]   1541365 g10595.t1,g10596.t1,g10597.t1,...
##    [6]   1951479 g11881.t1,g11883.t1,g11885.t1,...
##    [7]   1400142 g15616.t1,g15617.t1,g15619.t1,...
##    [8]   1948265    g5514.t1,g5516.t1,g5518.t1,...
##    [9]   1654567 g14704.t1,g14705.t1,g14706.t1,...
##   [10]   1815538 g16044.t1,g16046.t1,g16049.t1,...
##   [11]   1926893 g13995.t1,g13996.t1,g13998.t1,...
##                                                    HOGs dNdS_GUIDANCE2
##                                         <CharacterList>  <NumericList>
##    [1] N19.HOG0017981,N19.HOG0008164,N19.HOG0008380,...   NA,NA,NA,...
##    [2] N19.HOG0010002,N19.HOG0007494,N19.HOG0004125,...   NA,NA,NA,...
##    [3] N19.HOG0006561,N19.HOG0000191,N19.HOG0000494,...   NA,NA,NA,...
##    [4] N19.HOG0007652,N19.HOG0009801,N19.HOG0001158,...   NA,NA,NA,...
##    [5] N19.HOG0016788,N19.HOG0005932,N19.HOG0005632,...   NA,NA,NA,...
##    [6] N19.HOG0014254,N19.HOG0006862,N19.HOG0010374,...   NA,NA,NA,...
##    [7] N19.HOG0005961,N19.HOG0010189,N19.HOG0005657,...   NA,NA,NA,...
##    [8] N19.HOG0017875,N19.HOG0010773,N19.HOG0006227,...   NA,NA,NA,...
##    [9] N19.HOG0010737,N19.HOG0010730,N19.HOG0002063,...   NA,NA,NA,...
##   [10] N19.HOG0009647,N19.HOG0008252,N19.HOG0009828,...   NA,NA,NA,...
##   [11] N19.HOG0012364,N19.HOG0013854,N19.HOG0001468,...   NA,NA,NA,...
##        dNdS_HmmCleaner                  dNdS_PRANK geneNumber      Arm
##          <NumericList>               <NumericList>  <integer> <factor>
##    [1]    NA,NA,NA,...      NA,     NA,0.03369,...        199     long
##    [2]    NA,NA,NA,...      NA,0.05362,0.05119,...        200     XSR 
##    [3]    NA,NA,NA,...                NA,NA,NA,...        264     long
##    [4]    NA,NA,NA,...      NA,0.03930,0.02269,...        269     long
##    [5]    NA,NA,NA,...      NA,0.03304,0.04454,...        308     long
##    [6]    NA,NA,NA,...                NA,NA,NA,...        356     long
##    [7]    NA,NA,NA,... 0.02965,0.14260,     NA,...        367     XSR 
##    [8]    NA,NA,NA,...                NA,NA,NA,...        423     long
##    [9]    NA,NA,NA,... 0.04027,0.03903,     NA,...        438     XSR 
##   [10]    NA,NA,NA,...      NA,0.04442,0.14655,...        459     XSR 
##   [11]    NA,NA,NA,... 0.10461,0.18810,     NA,...        475     XSR 
##   -------
##   seqinfo: 19 sequences from OKI2018.I69 genome
```

### Block size histograms and statistics

```
# Let's make this more general, sometimes you just want an annotated histogram...
# Any numeric vector to a summary statistics table
numeric_vector_to_summary_df <- function(vec, name=NULL, digits=2) {
  summary_df <- vec |> summary() |> cbind() |>  as.data.frame() |> tibble::rownames_to_column("stat") |> dplyr::rename(value=2)
  # Replace names with nicer names
  summary_df$stat <- c("min", "1st_q", "median", "mean", "3rd_q", "max")
  summary_df$value <- round(summary_df$value, digits=digits)
  summary_df
}
numeric_vector_to_summary_df(orthoBlocks$Oki_Osa$geneNumber)
```

```
##     stat value
## 1    min  1.00
## 2  1st_q  1.00
## 3 median  1.00
## 4   mean  2.63
## 5  3rd_q  3.00
## 6    max 44.00
```

```
# Summary statistics to data.frame for use with ggplot
summary_df_to_geom_data <-
  function( summary_df
          , size=c(0.4, 0.2, 0.4, 0.4, 0.2, 0.4)
          , lty=c(1, 3, 1, 1, 3, 1)
          , col=c("#595959", "darkgray", "darkred", "darkred", "darkgray", "#595959"))
{
  summary_df$size <- size
  summary_df$lty <- lty
  summary_df$col <- col
  summary_df$xintercept <- summary_df$value
  summary_df
}
numeric_vector_to_summary_df(orthoBlocks$Oki_Osa$geneNumber) |> summary_df_to_geom_data()
```

```
##     stat value size lty      col xintercept
## 1    min  1.00  0.4   1  #595959       1.00
## 2  1st_q  1.00  0.2   3 darkgray       1.00
## 3 median  1.00  0.4   1  darkred       1.00
## 4   mean  2.63  0.4   1  darkred       2.63
## 5  3rd_q  3.00  0.2   3 darkgray       3.00
## 6    max 44.00  0.4   1  #595959      44.00
```

```
# Plot a histogram with lines and labels of statistics
gg_stat_hist <-
  function( df
          , plot.what="geneNumber"
          , include_stats=c("median", "max")
          , size=c(0.5, 0.3, 0.5, 0.4, 0.3, 0.5)
          , lty=c(1, 3, 1, 1, 3, 1)
          , col=c("#595959", "darkgray", "darkred", "darkred", "darkgray", "#595959")
          , bins=30
          , include_lines = TRUE, include_text=TRUE
          , digits=2, ...)
{
  if(!is.null(plot.what)) {
    if (inherits(df, "GBreaks"))
      df <- as.data.frame(df)
    # Extract the numeric vector and put it in convenient format
    numbers <- df[[plot.what]]
    numbers <- numbers |> as.data.frame() |> dplyr::as_tibble() |> dplyr::rename(value=1)
    # Get summary statistics
    numbers_stats <- numeric_vector_to_summary_df(numbers$value, digits = digits)
    numbers_stats <- summary_df_to_geom_data(numbers_stats, size = size, lty=lty, col=col)
    # Filter based on what we want to include
    numbers_stats <- numbers_stats |> dplyr::filter(stat %in% include_stats)

    # Make plot, add elements as needed
    p <- ggplot(numbers) + aes(x=value, ... ) + geom_histogram(bins=bins)
    if(include_lines){
      p <- p + geom_vline(xintercept = numbers_stats$xintercept, lty=numbers_stats$lty, col=numbers_stats$col, size=numbers_stats$size) + xlab(plot.what)
    }
    if(include_text) {
      label_vec <- paste0(numbers_stats$stat, '\n', numbers_stats$value)
      # Need to expose the Y values of the bins in order to
      # plot the correctY values for the labels.
      p_build <- ggplot_build(p)
      label_y <- 0.75*max(p_build[["data"]][[1]][["count"]])
      p <- p + annotate(geom = 'label', x=numbers_stats$xintercept, y = label_y, label=label_vec, col=numbers_stats$col)
    }
    p 
  }
  }

gg_stat_hist_genes <- \(x) gg_stat_hist(x, lty=3) + xlab('Number of genes') + ylab('Count')
```

```
# Close
p1 <- orthoBlocks$Oki_Kum |> gg_stat_hist_genes() + ggtitle("Oki-Kum: synteny block size distribution")
```

```
## Warning: Using `size` aesthetic for lines was deprecated in ggplot2 3.4.0.
## ℹ Please use `linewidth` instead.
## This warning is displayed once every 8 hours.
## Call `lifecycle::last_lifecycle_warnings()` to see where this warning was
## generated.
```

```
p2 <- orthoBlocks$Osa_Aom |> gg_stat_hist_genes() + ggtitle("Osa-Aom: synteny block size distribution")
p3 <- orthoBlocks$Bar_Nor |> gg_stat_hist_genes() + ggtitle("Bar-Nor: synteny block size distribution")
# Intermediate
p4 <- orthoBlocks$Osa_Bar |> gg_stat_hist_genes() + ggtitle("Osa-Bar: synteny block size distribution")
p5 <- orthoBlocks$Osa_Nor |> gg_stat_hist_genes() + ggtitle("Osa-Nor: synteny block size distribution")
p6 <- orthoBlocks$Bar_Aom |> gg_stat_hist_genes() + ggtitle("Bar-Aom: synteny block size distribution")
# Distant
p7 <- orthoBlocks$Oki_Osa |> gg_stat_hist_genes() + ggtitle("Oki-Osa: synteny block size distribution")
p8 <- orthoBlocks$Oki_Bar |> gg_stat_hist_genes() + ggtitle("Oki-Bar: synteny block size distribution")
p9 <- orthoBlocks$Osa_Kum |> gg_stat_hist_genes() + ggtitle("Osa-Kum: synteny block size distribution")

(p1 + p2 + p3) /
(p4 + p5 + p6) /
(p7 + p8 + p9)
```

```
# Transformed on X axis to simplify things.
# Close
p1 <- orthoBlocks$Oki_Kum |> gg_stat_hist_genes() + ggtitle("Oki-Kum: synteny block size distribution") + scale_x_continuous(trans="log10", limits=c(NA, 1000))
p2 <- orthoBlocks$Osa_Aom |> gg_stat_hist_genes() + ggtitle("Osa-Aom: synteny block size distribution") + scale_x_continuous(trans="log10", limits=c(NA, 1000))
p3 <- orthoBlocks$Bar_Nor |> gg_stat_hist_genes() + ggtitle("Bar-Nor: synteny block size distribution") + scale_x_continuous(trans="log10", limits=c(NA, 1000))
# Intermediate
p4 <- orthoBlocks$Osa_Bar |> gg_stat_hist_genes() + ggtitle("Osa-Bar: synteny block size distribution") + scale_x_continuous(trans="log10", limits=c(NA, 1000))
p5 <- orthoBlocks$Osa_Nor |> gg_stat_hist_genes() + ggtitle("Osa-Nor: synteny block size distribution") + scale_x_continuous(trans="log10", limits=c(NA, 1000))
p6 <- orthoBlocks$Bar_Aom |> gg_stat_hist_genes() + ggtitle("Bar-Aom: synteny block size distribution") + scale_x_continuous(trans="log10", limits=c(NA, 1000))
# Distant
p7 <- orthoBlocks$Oki_Osa |> gg_stat_hist_genes() + ggtitle("Oki-Osa: synteny block size distribution") + scale_x_continuous(trans="log10", limits=c(NA, 1000))
p8 <- orthoBlocks$Oki_Bar |> gg_stat_hist_genes() + ggtitle("Oki-Bar: synteny block size distribution") + scale_x_continuous(trans="log10", limits=c(NA, 1000))
p9 <- orthoBlocks$Osa_Kum |> gg_stat_hist_genes() + ggtitle("Osa-Kum: synteny block size distribution") + scale_x_continuous(trans="log10", limits=c(NA, 1000))

(p1 + p2 + p3) /
(p4 + p5 + p6) /
(p7 + p8 + p9)
```

```
## Warning: Removed 1 rows containing missing values (`geom_bar()`).
## Removed 1 rows containing missing values (`geom_bar()`).
## Removed 1 rows containing missing values (`geom_bar()`).
## Removed 1 rows containing missing values (`geom_bar()`).
## Removed 1 rows containing missing values (`geom_bar()`).
## Removed 1 rows containing missing values (`geom_bar()`).
## Removed 1 rows containing missing values (`geom_bar()`).
## Removed 1 rows containing missing values (`geom_bar()`).
## Removed 1 rows containing missing values (`geom_bar()`).
```

That’s all well and good. Unfortunately, none of these are very
beautiful. How about binning the synteny blocks into a nice barplot
instead?

```
num_vec_to_bins <- function(num_vec, return="n", from=1, to=100, by=10){
  starts <- seq(from=from, to=to, by=by)
  ends <- seq(from=by, to=to, by=by)
  bin_names <- paste0(starts, "-", ends)
  bin_names <- c(bin_names, paste0(">", max(ends)))
  num_vec_counts <- table(num_vec) |> as.data.frame()
  colnames(num_vec_counts) <- c('value', 'count')
  num_vec_counts$value <- as.numeric(as.character(num_vec_counts$value))
  starts_stops <- data.frame(start=c(starts, max(ends)+1), end=c(ends, Inf))
  
  count_abundance <- lapply(1:nrow(starts_stops), function(r) {
    start <- starts_stops$start[r]
    end   <- starts_stops$end[r]
    bin <- bin_names[r]
    vec_count_slice <- num_vec_counts[num_vec_counts$value >= start & num_vec_counts$value <= end ,]
    count <- 0
    n <- 0
    if(nrow(vec_count_slice) > 0){
      # n = number of observations
      n <- sum(vec_count_slice$count)
      # count = number of observations times the value (e.g., 5-length blocks * 30 = 150 genes)
      count <- sum(vec_count_slice$value * vec_count_slice$count)
      names(counts) <- bin
    }

    data.frame(bin=NA, n=n, count=count)
  })
  count_abundance <- do.call(rbind, count_abundance)
  count_abundance$bin <- factor(bin_names, levels=bin_names)
  count_abundance
}
orthoBlocks$Oki_Osa$geneNumber |> num_vec_to_bins()
```

```
##       bin    n count
## 1    1-10 3435  7057
## 2   11-20  124  1761
## 3   21-30   18   435
## 4   31-40    3   109
## 5   41-50    1    44
## 6   51-60    0     0
## 7   61-70    0     0
## 8   71-80    0     0
## 9   81-90    0     0
## 10 91-100    0     0
## 11   >100    0     0
```

```
# Join a few together
sb_df <- do.call(rbind, lapply(c("Osa_Oki", "Osa_Bar", "Osa_Aom"), function(pair) {
  orthoBlocks[[pair]]$geneNumber |> num_vec_to_bins() |> dplyr::mutate(sp=pair) |> dplyr::rename(num_genes=count, block_count=n, block_size=bin)
}))
sb_df$sp <- factor(sb_df$sp, levels=c("Osa_Oki", "Osa_Bar", "Osa_Aom"))

# Highest to lowest plots.
ggplot(sb_df) + aes(y=block_size, x=block_count, fill=sp) + geom_bar(stat='identity') + facet_wrap(~sp) + xlab("Block count") + ylab("Block size") + ggtitle("Synteny block size distribution")
```

```
# Small numbers hard to see, so try log transform.
# I multiply the values by 10, then divide the labels by 10, so that 1-counts are represented in the graph.
#   Idea from: https://stackoverflow.com/questions/64660203/ggplot2-and-log-scale-dont-show-values-1
ggplot(sb_df) + aes(y=block_size, x=block_count*10, fill=sp) + geom_bar(stat='identity') + facet_wrap(~sp) + scale_x_log10(labels=function(x) x/10) + xlab("Block count") + ylab("Block size") + ggtitle("Synteny block size distribution")
```

```
## Warning: Transformation introduced infinite values in continuous x-axis
```

```
## Warning: Removed 6 rows containing missing values (`geom_bar()`).
```

```
ggplot(sb_df) + aes(y=block_size, x=num_genes, fill=sp) + geom_bar(stat='identity') + facet_wrap(~sp) + xlab("Number of genes") + ylab("Block size") + ggtitle("Number of genes in synteny blocks")
```

```
# Lowest to highest plots. Same as above, just in opposite factor order.
sb_df$block_size <- factor(sb_df$block_size, levels=rev(levels(sb_df$block_size)))
ggplot(sb_df) + aes(y=block_size, x=block_count, fill=sp) + geom_bar(stat='identity') + facet_wrap(~sp) + xlab("Block count") + ylab("Block size") + ggtitle("Synteny block size distribution")
```

```
ggplot(sb_df) + aes(y=block_size, x=block_count*10, fill=sp) + geom_bar(stat='identity') + facet_wrap(~sp) + scale_x_log10(labels=function(x) x/10) + xlab("Block count") + ylab("Block size") + ggtitle("Synteny block size distribution")
```

```
## Warning: Transformation introduced infinite values in continuous x-axis
## Removed 6 rows containing missing values (`geom_bar()`).
```

```
ggplot(sb_df) + aes(y=block_size, x=num_genes, fill=sp) + geom_bar(stat='identity') + facet_wrap(~sp) + xlab("Number of genes") + ylab("Block size") + ggtitle("Number of genes in synteny blocks")
```

```
# Rank-abundance plot
sb_df_ra <- do.call(rbind, lapply(c("Osa_Oki", "Osa_Bar", "Osa_Aom"), function(pair) {
  orthoBlocks[[pair]]$geneNumber |> num_vec_to_bins() |> dplyr::rename(num_genes=count, block_count=n, block_size=bin) |> dplyr::mutate(sp=pair, rank_abundance=cumsum(num_genes)/sum(num_genes)*100 )
}))
sb_df_ra$sp <- factor(sb_df_ra$sp, levels=c("Osa_Oki", "Osa_Bar", "Osa_Aom"))

# Rank-abundance plot of tte same data
ggplot(sb_df_ra) + aes(x=block_size, y=rank_abundance, fill=sp, col=sp) + geom_point() + geom_line(group=1) + facet_wrap(~sp) + theme(axis.text.x = element_text(angle=45, vjust=0.7, hjust=0.5)) + xlab("Block size") + ylab("Fraction of genes in blocks") + ggtitle("Rank-abundance of synteny blocks by number of genes") + ylim(c(0,100))
```

## Gene Order Conservation is smaller in short arms

```
orthoBlocks_LSnoNA <- sapply(orthoBlocks[1:15], function(gb) gb[!is.na(gb$Arm)]) |> SimpleList()
sapply(orthoBlocks_LSnoNA, length)
```

```
## Oki_Osa Oki_Bar Oki_Kum Oki_Aom Oki_Nor Osa_Bar Osa_Oki Osa_Kum Osa_Aom Osa_Nor 
##    3579    3373     313    3531    3512    1402    3524    3480     410    1763 
## Bar_Osa Bar_Oki Bar_Kum Bar_Aom Bar_Nor 
##    1423    3350    3308    1398     892
```

```
df <- sapply(orthoBlocks_LSnoNA,
             function(gb) tapply(gb, paste(seqnames(gb), gb$Arm), GOC))

apply(df, 2, \(x) {
  tst <- t.test(x[c(1,3,5)], x[c(2,4,6)])
  c(pval=tst$p.value, mx=tst$estimate[1], my=tst$estimate[2])
})
```

```
##                   Oki_Osa     Oki_Bar   Oki_Kum    Oki_Aom     Oki_Nor
## pval         0.0003445748 0.001195248 0.4563762 0.01734870 0.008449444
## mx.mean of x 0.1853163526 0.181080056 0.5647566 0.18213329 0.174234572
## my.mean of y 0.0629330841 0.061442980 0.6188030 0.06829154 0.062364224
##                 Osa_Bar     Osa_Oki    Osa_Kum   Osa_Aom    Osa_Nor   Bar_Osa
## pval         0.05013474 0.002520974 0.00755681 0.0337040 0.01972651 0.1684680
## mx.mean of x 0.42449520 0.196644110 0.18306629 0.7109280 0.32803812 0.3897294
## my.mean of y 0.28083551 0.073813557 0.07284716 0.5246518 0.20384140 0.2739641
##                   Bar_Oki     Bar_Kum    Bar_Aom   Bar_Nor
## pval         0.0007675276 0.008239524 0.09425889 0.7257221
## mx.mean of x 0.1855144293 0.183651905 0.41363040 0.3391719
## my.mean of y 0.0709205522 0.070638469 0.27714160 0.3885798
```

Click to expand results on a microbenchmark comparing
`sapply(split)` and `tapply`

```
> microbenchmark::microbenchmark(
  times = 10,
  sapply = sapply(split(x, paste(seqnames(x), x$Arm)), GOC),
  tapply = tapply(x, paste(seqnames(x), x$Arm), GOC)
)
Unit: seconds
   expr      min       lq     mean   median       uq      max neval
 sapply 6.853530 6.921293 7.050267 6.949315 7.270793 7.477390    10
 tapply 6.921118 6.923827 7.028373 6.939487 6.968288 7.431115    10
```

## Fraction of the genome covered or syntenic

```
uncov  <- BiocParallel::bplapply(orthoPairsFiltered, cleanGaps) |> SimpleList()
unsyn  <- BiocParallel::bplapply(orthoBlocks,        cleanGaps) |> SimpleList()

  covered_tot    <- sapply(orthoPairsFiltered,   \(x) sum(width(x)))
  syntenic_tot   <- sapply(orthoBlocks,          \(x) sum(width(x)))
uncovered_tot    <- sapply(uncov,                \(x) sum(width(x)))
unsyntenic_tot   <- sapply(unsyn,                \(x) sum(width(x)))

(syn_summary <- cbind(
  covered_frac   = covered_tot   / ( covered_tot   + uncovered_tot ) * 100,
  orthogenes_n   = sapply(orthoPairsFiltered, length),
  syntenic_frac  = syntenic_tot  / ( syntenic_tot  + unsyntenic_tot ) * 100,
  syntenic_reg   = sapply(orthoBlocks, length)
 )|> as.data.frame())
```

```
##         covered_frac orthogenes_n syntenic_frac syntenic_reg
## Oki_Osa     38.33865         9406      61.29621         3581
## Oki_Bar     38.21979         9142      60.42189         3375
## Oki_Kum     43.70838        11323      92.95563          316
## Oki_Aom     38.60839         9438      61.35116         3533
## Oki_Nor     34.90562         8652      54.26659         3514
## Osa_Bar     42.10034         9074      76.09101         1443
## Osa_Oki     43.87692         9406      65.46897         3581
## Osa_Kum     42.96814         9351      63.74943         3533
## Osa_Aom     45.38916        10258      88.99277          455
## Osa_Nor     38.20910         8312      69.36702         1806
## Bar_Osa     40.94778         9074      73.69212         1443
## Bar_Oki     41.86938         9141      61.92448         3373
## Bar_Kum     42.24314         9104      62.47976         3333
## Bar_Aom     41.88404         9142      74.65392         1418
## Bar_Nor     38.13315         8383      75.49597          907
## Ply_Ros     25.53249         5412      56.86698         2185
```

```
sapply(syn_summary, \(x) tapply(x, row.names(syn_summary) |> OikScrambling:::compDistance(), mean))   |> round(1)
```

```
##               covered_frac orthogenes_n syntenic_frac syntenic_reg
## In same pop           42.4       9988.0          85.8        559.3
## Int – Int             25.5       5412.0          56.9       2185.0
## North – North         40.8       8900.5          73.5       1527.5
## Oki – North           40.1       9205.0          61.4       3477.9
```

```
sapply(syn_summary, \(x) tapply(x, row.names(syn_summary) |> OikScrambling:::compDistance(), median)) |> round(1)
```

```
##               covered_frac orthogenes_n syntenic_frac syntenic_reg
## In same pop           43.7      10258.0          89.0        455.0
## Int – Int             25.5       5412.0          56.9       2185.0
## North – North         41.4       9074.0          74.2       1443.0
## Oki – North           40.2       9246.5          61.6       3523.5
```

```
sapply(syn_summary, \(x) tapply(x, row.names(syn_summary) |> OikScrambling:::compDistance(), sd))     |> round(1)
```

```
##               covered_frac orthogenes_n syntenic_frac syntenic_reg
## In same pop            3.8       1488.5           9.2        309.0
## Int – Int               NA           NA            NA           NA
## North – North          1.8        393.6           2.9        186.0
## Oki – North            3.1        262.0           3.3        100.9
```

Unfortunately, there are not enough transcripts from Ciona that are
part of the pan-tunicate orthogroup set, which makes the comparison
difficult between Oikopleura and Ciona…

```
uncov  <- BiocParallel::bplapply(orthoPairsFiltered_core, cleanGaps) |> SimpleList()
unsyn  <- BiocParallel::bplapply(orthoBlocks_core,        cleanGaps) |> SimpleList()

  covered_tot    <- sapply(orthoPairsFiltered_core,   \(x) sum(width(x)))
  syntenic_tot   <- sapply(orthoBlocks_core,          \(x) sum(width(x)))
uncovered_tot    <- sapply(uncov,                     \(x) sum(width(x)))
unsyntenic_tot   <- sapply(unsyn,                     \(x) sum(width(x)))

(syn_summary_core <- cbind(
  covered_frac   = covered_tot   / ( covered_tot   + uncovered_tot ) * 100,
  orthogenes_n   = sapply(orthoPairsFiltered_core, length),
  syntenic_frac  = syntenic_tot  / ( syntenic_tot  + unsyntenic_tot ) * 100,
  syntenic_reg   = sapply(orthoBlocks_core, length)
 )|> as.data.frame())
```

```
##         covered_frac orthogenes_n syntenic_frac syntenic_reg
## Oki_Osa     27.48424         6720      51.25475         2618
## Oki_Bar     28.30197         6612      53.38051         2484
## Oki_Kum     31.22509         8190      91.90604          226
## Oki_Aom     28.75649         6809      53.16578         2598
## Oki_Nor     24.52914         6060      43.63803         2496
## Osa_Bar     31.13847         6635      70.58493         1119
## Osa_Oki     30.76206         6720      53.18648         2618
## Osa_Kum     30.69252         6630      53.01613         2548
## Osa_Aom     33.29049         7569      86.52898          361
## Osa_Nor     27.50044         5933      62.12065         1403
## Bar_Osa     30.15263         6635      67.64302         1119
## Bar_Oki     30.84109         6612      52.63710         2482
## Bar_Kum     31.17019         6558      52.57260         2449
## Bar_Aom     31.42929         6732      69.47170         1078
## Bar_Nor     27.93441         6087      70.48772          773
## Ply_Ros     11.39509         2572      50.30680          664
```

```
sapply(syn_summary_core, \(x) tapply(x, row.names(syn_summary_core) |> OikScrambling:::compDistance(), mean))   |> round(1)
```

```
##               covered_frac orthogenes_n syntenic_frac syntenic_reg
## In same pop           30.8       7282.0          83.0        453.3
## Int – Int             11.4       2572.0          50.3        664.0
## North – North         30.1       6483.8          67.5       1179.8
## Oki – North           29.1       6590.1          51.6       2536.6
```

```
sapply(syn_summary_core, \(x) tapply(x, row.names(syn_summary_core) |> OikScrambling:::compDistance(), median)) |> round(1)
```

```
##               covered_frac orthogenes_n syntenic_frac syntenic_reg
## In same pop           31.2         7569          86.5          361
## Int – Int             11.4         2572          50.3          664
## North – North         30.6         6635          68.6         1119
## Oki – North           29.7         6621          52.8         2522
```

```
sapply(syn_summary_core, \(x) tapply(x, row.names(syn_summary_core) |> OikScrambling:::compDistance(), sd))     |> round(1)
```

```
##               covered_frac orthogenes_n syntenic_frac syntenic_reg
## In same pop            2.7       1080.5          11.1        284.9
## Int – Int               NA           NA            NA           NA
## North – North          1.8        370.0           3.8        150.1
## Oki – North            2.3        228.7           3.3         67.8
```

## Agreement with nucleotide alignments

```
(gb_P <- orthoBlocks$Bar_Osa)
```

```
## GBreaks object with 1443 ranges and 9 metadata columns:
##          seqnames          ranges strand |                query     score
##             <Rle>       <IRanges>  <Rle> |            <GRanges> <integer>
##      [1]     Chr1     17796-21383      * | Chr1:3581251-3591380      3588
##      [2]     Chr1     27418-60748      * | Chr1:3596010-3622624     33331
##      [3]     Chr1     69763-74781      * | Chr1:3644537-3650503      5019
##      [4]     Chr1     87558-91675      * | Chr1:3818944-3824036      4118
##      [5]     Chr1    92754-103548      * | Chr1:3833970-3843493     10795
##      ...      ...             ...    ... .                  ...       ...
##   [1439]      YSR 4033286-4033798      * |  YSR:1457970-1458977       513
##   [1440]      YSR 4086487-4086957      * | Chr1:8499823-8501898       471
##   [1441]      YSR 4321609-4322813      * |  YSR:1599784-1602684      1205
##   [1442]      YSR 4865878-4866414      * |    YSR:434383-434709       537
##   [1443]      YSR 5097970-5098632      * |  YSR:1729503-1738428       663
##                         geneNames
##                   <CharacterList>
##      [1]              g1.t1,g2.t1
##      [2]    g4.t1,g5.t1,g6.t1,...
##      [3] g16.t1,g17.t1,g18.t1,...
##      [4]            g22.t1,g23.t1
##      [5]            g24.t1,g25.t1
##      ...                      ...
##   [1439]                g13965.t1
##   [1440]                g13972.t1
##   [1441]                g13991.t1
##   [1442]                g14068.t1
##   [1443]                g14100.t1
##                                                      HOGs dNdS_GUIDANCE2
##                                           <CharacterList>  <NumericList>
##      [1]                    N19.HOG0005689,N19.HOG0003982          NA,NA
##      [2] N19.HOG0003457,N19.HOG0013902,N19.HOG0000362,...   NA,NA,NA,...
##      [3] N19.HOG0010654,N19.HOG0002933,N19.HOG0000127,...   NA,NA,NA,...
##      [4]                    N19.HOG0013826,N19.HOG0008116          NA,NA
##      [5]                    N19.HOG0004909,N19.HOG0012557          NA,NA
##      ...                                              ...            ...
##   [1439]                                   N19.HOG0017454             NA
##   [1440]                                   N19.HOG0011502             NA
##   [1441]                                   N19.HOG0000625             NA
##   [1442]                                   N19.HOG0017456             NA
##   [1443]                                   N19.HOG0012483             NA
##          dNdS_HmmCleaner                  dNdS_PRANK geneNumber      Arm
##            <NumericList>               <NumericList>  <integer> <factor>
##      [1]           NA,NA             0.05199,0.14450          2    short
##      [2]    NA,NA,NA,...                NA,NA,NA,...          8    short
##      [3]    NA,NA,NA,... 0.02980,0.05181,0.06409,...          4    short
##      [4]           NA,NA                       NA,NA          2    short
##      [5]           NA,NA                  NA,0.17757          2    short
##      ...             ...                         ...        ...      ...
##   [1439]              NA                          NA          1      YSR
##   [1440]              NA                          NA          1      YSR
##   [1441]              NA                          NA          1      YSR
##   [1442]              NA                          NA          1      YSR
##   [1443]              NA                          NA          1      YSR
##   -------
##   seqinfo: 68 sequences from Bar2.p4 genome
```

```
sum(width(gb_P))
```

```
## [1] 39929544
```

```
(gb_N <- coa$Bar_Osa)
```

```
## GBreaks object with 4073 ranges and 8 metadata columns:
##          seqnames          ranges strand |                query     score
##             <Rle>       <IRanges>  <Rle> |            <GRanges> <integer>
##      [1]     Chr1     15573-21624      - | Chr1:3585703-3594761      6052
##      [2]     Chr1     22042-22295      + | Chr1:3585140-3585392       254
##      [3]     Chr1     22421-24048      - | Chr1:3583028-3584936      1628
##      [4]     Chr1     27262-34408      + | Chr1:3595848-3601817      7147
##      [5]     Chr1     34435-34998      - | Chr1:3602092-3602509       564
##      ...      ...             ...    ... .                  ...       ...
##   [4069]      YSR 5273802-5274176      - |  YSR:1632176-1632556       375
##   [4070]      YSR 5288325-5289549      - |  YSR:1086332-1087481      1225
##   [4071]      YSR 5307497-5310556      - | Chr1:9561929-9564982      3060
##   [4072]      YSR 5322809-5325938      - |  PAR:5433966-5437098      3130
##   [4073]      YSR 5335965-5336405      - |   Chr2:928224-928659       441
##               Arm                rep   repOvlp transcripts        flag
##          <factor>    <CharacterList> <integer>       <Rle> <character>
##      [1]    short tandem,unknown,rnd       210       g2.t1         Inv
##      [2]    short               <NA>         0        <NA>        <NA>
##      [3]    short               <NA>         0        <NA>        <NA>
##      [4]    short                rnd       390        <NA>         Inv
##      [5]    short                rnd         0       g5.t1        <NA>
##      ...      ...                ...       ...         ...         ...
##   [4069]      YSR               <NA>         0        <NA>        <NA>
##   [4070]      YSR               <NA>         0   g14124.t1        <NA>
##   [4071]      YSR                rnd         0   g14124.t1        <NA>
##   [4072]      YSR                rnd         0        <NA>        <NA>
##   [4073]      YSR                rnd       304        <NA>        <NA>
##             nonCoa
##          <logical>
##      [1]     FALSE
##      [2]      TRUE
##      [3]     FALSE
##      [4]     FALSE
##      [5]      TRUE
##      ...       ...
##   [4069]      TRUE
##   [4070]      TRUE
##   [4071]      TRUE
##   [4072]      TRUE
##   [4073]      TRUE
##   -------
##   seqinfo: 68 sequences from Bar2.p4 genome
```

```
sum(width(gb_N))
```

```
## [1] 45289525
```

```
(ov <- subsetByOverlaps(gb_P, gb_N, type = "within"))
```

```
## GBreaks object with 918 ranges and 9 metadata columns:
##         seqnames          ranges strand |                query     score
##            <Rle>       <IRanges>  <Rle> |            <GRanges> <integer>
##     [1]     Chr1     17796-21383      * | Chr1:3581251-3591380      3588
##     [2]     Chr1     69763-74781      * | Chr1:3644537-3650503      5019
##     [3]     Chr1     87558-91675      * | Chr1:3818944-3824036      4118
##     [4]     Chr1    92754-103548      * | Chr1:3833970-3843493     10795
##     [5]     Chr1   103823-135737      * | Chr1:2195467-2232252     31915
##     ...      ...             ...    ... .                  ...       ...
##   [914]      YSR 3597539-3598992      * |   Chr1:269069-271045      1454
##   [915]      YSR 3599919-3601165      * | Chr1:4173793-4175304      1247
##   [916]      YSR 3631705-3633609      * | Chr1:4315390-4317451      1905
##   [917]      YSR 4086487-4086957      * | Chr1:8499823-8501898       471
##   [918]      YSR 4865878-4866414      * |    YSR:434383-434709       537
##                        geneNames
##                  <CharacterList>
##     [1]              g1.t1,g2.t1
##     [2] g16.t1,g17.t1,g18.t1,...
##     [3]            g22.t1,g23.t1
##     [4]            g24.t1,g25.t1
##     [5] g26.t1,g27.t1,g28.t1,...
##     ...                      ...
##   [914]                g13905.t1
##   [915]                g13906.t1
##   [916]                g13910.t1
##   [917]                g13972.t1
##   [918]                g14068.t1
##                                                     HOGs dNdS_GUIDANCE2
##                                          <CharacterList>  <NumericList>
##     [1]                    N19.HOG0005689,N19.HOG0003982          NA,NA
##     [2] N19.HOG0010654,N19.HOG0002933,N19.HOG0000127,...   NA,NA,NA,...
##     [3]                    N19.HOG0013826,N19.HOG0008116          NA,NA
##     [4]                    N19.HOG0004909,N19.HOG0012557          NA,NA
##     [5] N19.HOG0013544,N19.HOG0005691,N19.HOG0006532,...   NA,NA,NA,...
##     ...                                              ...            ...
##   [914]                                   N19.HOG0000317             NA
##   [915]                                   N19.HOG0005253             NA
##   [916]                                   N19.HOG0005027             NA
##   [917]                                   N19.HOG0011502             NA
##   [918]                                   N19.HOG0017456             NA
##         dNdS_HmmCleaner                  dNdS_PRANK geneNumber      Arm
##           <NumericList>               <NumericList>  <integer> <factor>
##     [1]           NA,NA             0.05199,0.14450          2    short
##     [2]    NA,NA,NA,... 0.02980,0.05181,0.06409,...          4    short
##     [3]           NA,NA                       NA,NA          2    short
##     [4]           NA,NA                  NA,0.17757          2    short
##     [5]    NA,NA,NA,... 0.03898,     NA,0.06625,...          5    short
##     ...             ...                         ...        ...      ...
##   [914]              NA                     0.08761          1      YSR
##   [915]              NA                          NA          1      YSR
##   [916]              NA                     0.05509          1      YSR
##   [917]              NA                          NA          1      YSR
##   [918]              NA                          NA          1      YSR
##   -------
##   seqinfo: 68 sequences from Bar2.p4 genome
```

```
(jaccard <- sum(width(ov)) / sum(width(reduce(c(gb_P, gb_N)))))
```

```
## [1] 0.1434809
```

```
gb_P <- gb_P[gb_P$geneNumber > 1]
gb_P
```

```
## GBreaks object with 867 ranges and 9 metadata columns:
##         seqnames            ranges strand |                  query     score
##            <Rle>         <IRanges>  <Rle> |              <GRanges> <integer>
##     [1]     Chr1       17796-21383      * |   Chr1:3581251-3591380      3588
##     [2]     Chr1       27418-60748      * |   Chr1:3596010-3622624     33331
##     [3]     Chr1       69763-74781      * |   Chr1:3644537-3650503      5019
##     [4]     Chr1       87558-91675      * |   Chr1:3818944-3824036      4118
##     [5]     Chr1      92754-103548      * |   Chr1:3833970-3843493     10795
##     ...      ...               ...    ... .                    ...       ...
##   [863]      XSR 12286992-12366325      * |    XSR:9201073-9267597     79334
##   [864]      YSR     268825-302025      * |   Chr1:1911761-1929212     33201
##   [865]      YSR     518298-532357      * | Chr2:12195648-12210212     14060
##   [866]      YSR   1387847-1390918      * |   Chr1:1811359-1814424      3072
##   [867]      YSR   2712461-2719315      * |    YSR:1128232-1134212      6855
##                                 geneNames
##                           <CharacterList>
##     [1]                       g1.t1,g2.t1
##     [2]             g4.t1,g5.t1,g6.t1,...
##     [3]          g16.t1,g17.t1,g18.t1,...
##     [4]                     g22.t1,g23.t1
##     [5]                     g24.t1,g25.t1
##     ...                               ...
##   [863] g13291.t1,g13292.t2,g13293.t2,...
##   [864] g13345.t1,g13347.t1,g13348.t1,...
##   [865]               g13390.t1,g13392.t1
##   [866]               g13542.t1,g13543.t1
##   [867]               g13745.t1,g13747.t1
##                                                     HOGs dNdS_GUIDANCE2
##                                          <CharacterList>  <NumericList>
##     [1]                    N19.HOG0005689,N19.HOG0003982          NA,NA
##     [2] N19.HOG0003457,N19.HOG0013902,N19.HOG0000362,...   NA,NA,NA,...
##     [3] N19.HOG0010654,N19.HOG0002933,N19.HOG0000127,...   NA,NA,NA,...
##     [4]                    N19.HOG0013826,N19.HOG0008116          NA,NA
##     [5]                    N19.HOG0004909,N19.HOG0012557          NA,NA
##     ...                                              ...            ...
##   [863] N19.HOG0015299,N19.HOG0009625,N19.HOG0001389,...   NA,NA,NA,...
##   [864] N19.HOG0017430,N19.HOG0013161,N19.HOG0016700,...   NA,NA,NA,...
##   [865]                    N19.HOG0011808,N19.HOG0012257          NA,NA
##   [866]                    N19.HOG0010219,N19.HOG0017438          NA,NA
##   [867]                    N19.HOG0013552,N19.HOG0012421          NA,NA
##         dNdS_HmmCleaner                  dNdS_PRANK geneNumber      Arm
##           <NumericList>               <NumericList>  <integer> <factor>
##     [1]           NA,NA             0.05199,0.14450          2    short
##     [2]    NA,NA,NA,...                NA,NA,NA,...          8    short
##     [3]    NA,NA,NA,... 0.02980,0.05181,0.06409,...          4    short
##     [4]           NA,NA                       NA,NA          2    short
##     [5]           NA,NA                  NA,0.17757          2    short
##     ...             ...                         ...        ...      ...
##   [863]    NA,NA,NA,...                NA,NA,NA,...         20      XSR
##   [864]    NA,NA,NA,...                NA,NA,NA,...          4      YSR
##   [865]           NA,NA                       NA,NA          2      YSR
##   [866]           NA,NA                       NA,NA          2      YSR
##   [867]           NA,NA                       NA,NA          2      YSR
##   -------
##   seqinfo: 68 sequences from Bar2.p4 genome
```

```
sum(gb_P$geneNumber)
```

```
## [1] 8498
```

```
(valid <- subsetByOverlaps(gb_P, gb_N, type = "within"))
```

```
## GBreaks object with 462 ranges and 9 metadata columns:
##         seqnames            ranges strand |                query     score
##            <Rle>         <IRanges>  <Rle> |            <GRanges> <integer>
##     [1]     Chr1       17796-21383      * | Chr1:3581251-3591380      3588
##     [2]     Chr1       69763-74781      * | Chr1:3644537-3650503      5019
##     [3]     Chr1       87558-91675      * | Chr1:3818944-3824036      4118
##     [4]     Chr1      92754-103548      * | Chr1:3833970-3843493     10795
##     [5]     Chr1     103823-135737      * | Chr1:2195467-2232252     31915
##     ...      ...               ...    ... .                  ...       ...
##   [458]      XSR 12170086-12180178      * |  XSR:9269610-9279934     10093
##   [459]      XSR 12180804-12192236      * |    XSR:811181-825617     11433
##   [460]      XSR 12279536-12286707      * |  XSR:1674570-1684997      7172
##   [461]      XSR 12286992-12366325      * |  XSR:9201073-9267597     79334
##   [462]      YSR   1387847-1390918      * | Chr1:1811359-1814424      3072
##                                 geneNames
##                           <CharacterList>
##     [1]                       g1.t1,g2.t1
##     [2]          g16.t1,g17.t1,g18.t1,...
##     [3]                     g22.t1,g23.t1
##     [4]                     g24.t1,g25.t1
##     [5]          g26.t1,g27.t1,g28.t1,...
##     ...                               ...
##   [458] g13261.t1,g13262.t1,g13263.t1,...
##   [459]               g13265.t1,g13267.t1
##   [460]               g13289.t1,g13290.t1
##   [461] g13291.t1,g13292.t2,g13293.t2,...
##   [462]               g13542.t1,g13543.t1
##                                                     HOGs dNdS_GUIDANCE2
##                                          <CharacterList>  <NumericList>
##     [1]                    N19.HOG0005689,N19.HOG0003982          NA,NA
##     [2] N19.HOG0010654,N19.HOG0002933,N19.HOG0000127,...   NA,NA,NA,...
##     [3]                    N19.HOG0013826,N19.HOG0008116          NA,NA
##     [4]                    N19.HOG0004909,N19.HOG0012557          NA,NA
##     [5] N19.HOG0013544,N19.HOG0005691,N19.HOG0006532,...   NA,NA,NA,...
##     ...                                              ...            ...
##   [458] N19.HOG0004798,N19.HOG0008402,N19.HOG0007671,...   NA,NA,NA,...
##   [459]                    N19.HOG0007002,N19.HOG0009694          NA,NA
##   [460]                    N19.HOG0007715,N19.HOG0013901          NA,NA
##   [461] N19.HOG0015299,N19.HOG0009625,N19.HOG0001389,...   NA,NA,NA,...
##   [462]                    N19.HOG0010219,N19.HOG0017438          NA,NA
##         dNdS_HmmCleaner                  dNdS_PRANK geneNumber      Arm
##           <NumericList>               <NumericList>  <integer> <factor>
##     [1]           NA,NA             0.05199,0.14450          2    short
##     [2]    NA,NA,NA,... 0.02980,0.05181,0.06409,...          4    short
##     [3]           NA,NA                       NA,NA          2    short
##     [4]           NA,NA                  NA,0.17757          2    short
##     [5]    NA,NA,NA,... 0.03898,     NA,0.06625,...          5    short
##     ...             ...                         ...        ...      ...
##   [458]    NA,NA,NA,... 0.03260,0.06604,     NA,...          4      XSR
##   [459]           NA,NA             0.01990,0.09299          2      XSR
##   [460]           NA,NA                  NA,0.10336          2      XSR
##   [461]    NA,NA,NA,...                NA,NA,NA,...         20      XSR
##   [462]           NA,NA                       NA,NA          2      YSR
##   -------
##   seqinfo: 68 sequences from Bar2.p4 genome
```

```
sum(valid$geneNumber)
```

```
## [1] 2825
```

```
sum(width(valid))
```

```
## [1] 11168468
```

Question: what does interrupt the colinearity of nucleotide regions
in gene-centric colinear regions ?

## HOX genes

### Hox 4

Appears to be duplicated in Bar so missing from HOG
`N19.HOG0000192` in that genome.

```
sapply(orthoPairs[1:15], \(gb) {
  x <- gb |> plyranges::filter(HOG == "N19.HOG0000192")
  if (length(x) == 0) return(NULL)
  x})
```

```
## $Oki_Osa
## GBreaks object with 1 range and 9 metadata columns:
##            seqnames          ranges strand |     tx_id     tx_name
##               <Rle>       <IRanges>  <Rle> | <integer> <character>
##   g5193.t1     chr2 4686018-4687168      * |      7545    g5193.t1
##            dNdS_GUIDANCE2 dNdS_HmmCleaner dNdS_PRANK                query
##                 <numeric>       <numeric>  <numeric>            <GRanges>
##   g5193.t1             NA              NA         NA Chr2:1156287-1157412
##                       HOG          OG      Arm
##               <character> <character> <factor>
##   g5193.t1 N19.HOG0000192   OG0000004    short
##   -------
##   seqinfo: 19 sequences from OKI2018.I69 genome
## 
## $Oki_Bar
## NULL
## 
## $Oki_Kum
## GBreaks object with 1 range and 9 metadata columns:
##            seqnames          ranges strand |     tx_id     tx_name
##               <Rle>       <IRanges>  <Rle> | <integer> <character>
##   g5193.t1     chr2 4686018-4687168      * |      7545    g5193.t1
##            dNdS_GUIDANCE2 dNdS_HmmCleaner dNdS_PRANK
##                 <numeric>       <numeric>  <numeric>
##   g5193.t1             NA              NA         NA
##                                  query            HOG          OG      Arm
##                              <GRanges>    <character> <character> <factor>
##   g5193.t1 contig_16_1:4184659-4186299 N19.HOG0000192   OG0000004    short
##   -------
##   seqinfo: 19 sequences from OKI2018.I69 genome
## 
## $Oki_Aom
## NULL
## 
## $Oki_Nor
## GBreaks object with 1 range and 9 metadata columns:
##            seqnames          ranges strand |     tx_id     tx_name
##               <Rle>       <IRanges>  <Rle> | <integer> <character>
##   g5193.t1     chr2 4686018-4687168      * |      7545    g5193.t1
##            dNdS_GUIDANCE2 dNdS_HmmCleaner dNdS_PRANK                     query
##                 <numeric>       <numeric>  <numeric>                 <GRanges>
##   g5193.t1             NA              NA         NA scaffold_47:176335-180537
##                       HOG          OG      Arm
##               <character> <character> <factor>
##   g5193.t1 N19.HOG0000192   OG0000004    short
##   -------
##   seqinfo: 19 sequences from OKI2018.I69 genome
## 
## $Osa_Bar
## NULL
## 
## $Osa_Oki
## GBreaks object with 1 range and 9 metadata columns:
##            seqnames          ranges strand |     tx_id     tx_name
##               <Rle>       <IRanges>  <Rle> | <integer> <character>
##   g3346.t1     Chr2 1156287-1157412      * |      5799    g3346.t1
##            dNdS_GUIDANCE2 dNdS_HmmCleaner dNdS_PRANK                query
##                 <numeric>       <numeric>  <numeric>            <GRanges>
##   g3346.t1             NA              NA         NA chr2:4686018-4687168
##                       HOG          OG      Arm
##               <character> <character> <factor>
##   g3346.t1 N19.HOG0000192   OG0000004    short
##   -------
##   seqinfo: 483 sequences from OSKA2016v1.9 genome
## 
## $Osa_Kum
## GBreaks object with 1 range and 9 metadata columns:
##            seqnames          ranges strand |     tx_id     tx_name
##               <Rle>       <IRanges>  <Rle> | <integer> <character>
##   g3346.t1     Chr2 1156287-1157412      * |      5799    g3346.t1
##            dNdS_GUIDANCE2 dNdS_HmmCleaner dNdS_PRANK
##                 <numeric>       <numeric>  <numeric>
##   g3346.t1             NA              NA         NA
##                                  query            HOG          OG      Arm
##                              <GRanges>    <character> <character> <factor>
##   g3346.t1 contig_16_1:4184659-4186299 N19.HOG0000192   OG0000004    short
##   -------
##   seqinfo: 483 sequences from OSKA2016v1.9 genome
## 
## $Osa_Aom
## NULL
## 
## $Osa_Nor
## GBreaks object with 1 range and 9 metadata columns:
##            seqnames          ranges strand |     tx_id     tx_name
##               <Rle>       <IRanges>  <Rle> | <integer> <character>
##   g3346.t1     Chr2 1156287-1157412      * |      5799    g3346.t1
##            dNdS_GUIDANCE2 dNdS_HmmCleaner dNdS_PRANK                     query
##                 <numeric>       <numeric>  <numeric>                 <GRanges>
##   g3346.t1             NA              NA         NA scaffold_47:176335-180537
##                       HOG          OG      Arm
##               <character> <character> <factor>
##   g3346.t1 N19.HOG0000192   OG0000004    short
##   -------
##   seqinfo: 483 sequences from OSKA2016v1.9 genome
## 
## $Bar_Osa
## NULL
## 
## $Bar_Oki
## NULL
## 
## $Bar_Kum
## NULL
## 
## $Bar_Aom
## NULL
## 
## $Bar_Nor
## NULL
```

```
Hox4 <- orthoPairs$Oki_Osa |> plyranges::filter(HOG == "N19.HOG0000192")
Hox4_1e4 <- GBreaks(target = granges(Hox4) + 1e4, query = Hox4$query + 1e4)
Hox4_4e4 <- GBreaks(target = granges(Hox4) + 4e4, query = Hox4$query + 4e4)
Hox4_1e5 <- GBreaks(target = granges(Hox4) + 1e5, query = Hox4$query + 1e5)

subsetByOverlaps(coa$Oki_Osa, Hox4_1e4) |> plotApairOfChrs("chr2", xlim = gb2xlim(Hox4_1e4))
```

```
subsetByOverlaps(coa$Oki_Osa, Hox4_4e4) |> plotApairOfChrs("chr2", xlim = gb2xlim(Hox4_4e4))
```

```
subsetByOverlaps(coa$Oki_Osa, Hox4_1e5) |> plotApairOfChrs("chr2", xlim = gb2xlim(Hox4_1e5))
```

```
## Genes
Hox4_4e4.genes <- transcripts$OKI2018.I69 |> subsetByOverlaps(Hox4_4e4 |> range())
mids <- Hox4_4e4.genes |> gr2dna_seg() |> dplyr::mutate(mid = (start + end) / 2) |> dplyr::pull(mid)
text <- Hox4_4e4.genes$tx_name |> as.character()
annot <- genoPlotR::annotation(x1=start(Hox4_4e4.genes), x2=end(Hox4_4e4.genes), text=text, rot=30)
gbs$Oki_Osa |> plotApairOfChrs("chr2", xlim = gb2xlim(Hox4_4e4), annotations = annot)
```

Click to see details on where the non-plotted alignments go.

```
subsetByOverlaps(gbs$Oki_Osa, granges(Hox4_4e4)) |> sort(i=T)  |> data.frame()
```

```
##    seqnames   start     end width strand score query.seqnames query.start
## 1      chr2 4680017 4680185   169      -   450           Chr2     1130131
## 2      chr2 4680306 4680615   310      -   751           Chr2     1129482
## 3      chr2 4680646 4680944   299      -   821           Chr2     1128741
## 4      chr2 4681217 4681508   292      -   717           Chr2     1128441
## 5      chr2 4682067 4682396   330      -   482           Chr2     1127536
## 6      chr2 4682468 4683740  1273      -  1764           Chr2     1126128
## 7      chr2 4684457 4684873   417      -   442           Chr2     1125551
## 8      chr2 4685956 4686922   967      +  1473           Chr2     1156232
## 9      chr2 4687083 4688536  1454      +  1728           Chr2     1157327
## 10     chr2 4692590 4693427   838      -  1128           Chr2     3612906
## 11     chr2 4695931 4696033   103      +   355           Chr2     3611932
## 12     chr2 4696242 4696708   467      -   982           Chr2     2917549
## 13     chr2 4707204 4708706  1503      -  2221           Chr2     2916035
## 14     chr2 4714542 4717249  2708      +  4868           Chr2     2842120
## 15     chr2 4717795 4717995   201      +   258           Chr2     2844493
## 16     chr2 4718344 4718458   115      +   315           Chr2     2844977
##    query.end query.width query.strand query.Arm query.rep query.repOvlp
## 1    1130308         178            *     short        NA             0
## 2    1129785         304            *     short       rnd             6
## 3    1129039         299            *     short        NA             0
## 4    1128739         299            *     short        NA             0
## 5    1127867         332            *     short        NA             0
## 6    1127291        1164            *     short       rnd            18
## 7    1125975         425            *     short        NA             0
## 8    1157087         856            *     short        NA             0
## 9    1158757        1431            *     short    tandem            58
## 10   3613565         660            *     short        NA             0
## 11   3612034         103            *     short        NA             0
## 12   2917998         450            *     short        NA             0
## 13   2917544        1510            *     short        NA             0
## 14   2844401        2282            *     short       rnd            51
## 15   2844717         225            *     short        NA             0
## 16   2845091         115            *     short        NA             0
##    query.transcripts   Arm         rep repOvlp transcripts flag nonCoa
## 1  g3343.t1;g3343.t2 short          NA       0        <NA>  Col  FALSE
## 2  g3343.t1;g3343.t2 short          NA       0        <NA>  Col  FALSE
## 3  g3343.t1;g3343.t2 short          NA       0        <NA>  Col  FALSE
## 4  g3343.t1;g3343.t2 short          NA       0        <NA>  Col  FALSE
## 5  g3343.t1;g3343.t2 short          NA       0        <NA>  Col  FALSE
## 6  g3343.t1;g3343.t2 short          NA       0        <NA>  Col  FALSE
## 7  g3343.t1;g3343.t2 short          NA       0        <NA> <NA>  FALSE
## 8           g3346.t1 short          NA       0        <NA>  Col  FALSE
## 9           g3346.t1 short          NA       0        <NA> <NA>  FALSE
## 10          g4058.t1 short   rnd, SINE      89    g5195.t1 <NA>   TRUE
## 11              <NA> short          NA       0        <NA> <NA>   TRUE
## 12          g3845.t1 short          NA       0        <NA>  Col  FALSE
## 13          g3845.t1 short          NA       0        <NA> <NA>  FALSE
## 14 g3815.t1;g3816.t1 short rnd, tandem      25    g5200.t1  Col  FALSE
## 15          g3816.t1 short          NA       0        <NA>  Col  FALSE
## 16          g3816.t1 short          NA       0        <NA> <NA>  FALSE
```

```
subsetByOverlaps(swap(gbs$Oki_Osa), granges(swap(Hox4_4e4))) |> sort(i=T)  |> data.frame()
```

```
##    seqnames   start     end width strand   Arm          rep repOvlp
## 1      Chr2 1116356 1116510   155      + short           NA       0
## 2      Chr2 1117617 1117737   121      + short           NA       0
## 3      Chr2 1117896 1118034   139      - short           NA       0
## 4      Chr2 1119050 1119261   212      - short           NA       0
## 5      Chr2 1122014 1122116   103      + short           NA       0
## 6      Chr2 1122663 1122777   115      - short           NA       0
## 7      Chr2 1125551 1125975   425      - short           NA       0
## 8      Chr2 1126128 1127291  1164      - short          rnd      18
## 9      Chr2 1127536 1127867   332      - short           NA       0
## 10     Chr2 1128441 1128739   299      - short           NA       0
## 11     Chr2 1128741 1129039   299      - short           NA       0
## 12     Chr2 1129482 1129785   304      - short          rnd       6
## 13     Chr2 1130131 1130308   178      - short           NA       0
## 14     Chr2 1143345 1146561  3217      - short           NA       0
## 15     Chr2 1146661 1146869   209      - short           NA       0
## 16     Chr2 1147032 1147428   397      + short           NA       0
## 17     Chr2 1147686 1148058   373      + short LowCompl....      37
## 18     Chr2 1148434 1148642   209      - short           NA       0
## 19     Chr2 1154904 1155305   402      - short           NA       0
## 20     Chr2 1156232 1157087   856      + short           NA       0
## 21     Chr2 1157327 1158757  1431      + short       tandem      58
## 22     Chr2 1162301 1162866   566      - short           NA       0
## 23     Chr2 1165237 1166110   874      + short       tandem      62
## 24     Chr2 1167024 1168181  1158      + short           NA       0
## 25     Chr2 1170520 1170816   297      - short          rnd      82
## 26     Chr2 1172127 1172242   116      + short           NA       0
## 27     Chr2 1172509 1172726   218      + short           NA       0
## 28     Chr2 1175781 1175935   155      - short           NA       0
## 29     Chr2 1175980 1176136   157      - short           NA       0
## 30     Chr2 1176714 1176965   252      - short           NA       0
## 31     Chr2 1177240 1177619   380      + short           NA       0
## 32     Chr2 1179193 1179404   212      - short           NA       0
## 33     Chr2 1179725 1179917   193      + short           NA       0
## 34     Chr2 1180191 1180422   232      + short           NA       0
## 35     Chr2 1180978 1181238   261      - short LowCompl....      52
## 36     Chr2 1181615 1181861   247      - short           NA       0
## 37     Chr2 1183092 1183265   174      - short           NA       0
## 38     Chr2 1183512 1183682   171      + short           NA       0
## 39     Chr2 1185336 1185510   175      - short           NA       0
## 40     Chr2 1186670 1186786   117      - short           NA       0
## 41     Chr2 1187265 1188647  1383      - short           NA       0
## 42     Chr2 1189530 1189790   261      + short           NA       0
## 43     Chr2 1190125 1190380   256      - short           NA       0
## 44     Chr2 1192014 1192315   302      - short           NA       0
## 45     Chr2 1192688 1193211   524      + short           NA       0
## 46     Chr2 1194037 1194926   890      - short           NA       0
## 47     Chr2 1195129 1195830   702      - short           NA       0
## 48     Chr2 1195952 1196071   120      - short           NA       0
##          transcripts query.seqnames query.start query.end query.width
## 1               <NA>           chr2     1023370   1023532         163
## 2               <NA>           chr2     1019589   1019734         146
## 3               <NA>           chr2     1019029   1019173         145
## 4               <NA>           chr2     1017389   1017657         269
## 5               <NA>           chr2     1010105   1010207         103
## 6               <NA>           chr2     1007291   1007406         116
## 7  g3343.t1;g3343.t2           chr2     4684457   4684873         417
## 8  g3343.t1;g3343.t2           chr2     4682468   4683740        1273
## 9  g3343.t1;g3343.t2           chr2     4682067   4682396         330
## 10 g3343.t1;g3343.t2           chr2     4681217   4681508         292
## 11 g3343.t1;g3343.t2           chr2     4680646   4680944         299
## 12 g3343.t1;g3343.t2           chr2     4680306   4680615         310
## 13 g3343.t1;g3343.t2           chr2     4680017   4680185         169
## 14 g3344.t1;g3344.t2           chr2     2332229   2335564        3336
## 15              <NA>           chr2     2331312   2331545         234
## 16              <NA>           chr2     2331643   2332043         401
## 17              <NA>           chr2     2330703   2331067         365
## 18              <NA>           chr2     2330196   2330404         209
## 19          g3345.t1           chr1     5048358   5048789         432
## 20          g3346.t1           chr2     4685956   4686922         967
## 21          g3346.t1           chr2     4687083   4688536        1454
## 22          g3348.t1           chr1     1196277   1196833         557
## 23          g3350.t1           chr2     2375085   2375971         887
## 24          g3351.t1           chr2     2376702   2377879        1178
## 25              <NA>           chr2     2303024   2303357         334
## 26              <NA>           chr2     2301006   2301121         116
## 27              <NA>           chr2     2297961   2298224         264
## 28          g3353.t1           chr2     2297391   2297553         163
## 29          g3353.t1           chr2     2296032   2296209         178
## 30          g3353.t1           chr2     2296898   2297147         250
## 31          g3353.t1           chr2     2294607   2295014         408
## 32          g3353.t1           chr2     2293313   2293516         204
## 33          g3353.t1           chr2     2292910   2293103         194
## 34          g3353.t1           chr2     2291596   2291823         228
## 35          g3353.t1           chr2     2290662   2290931         270
## 36          g3353.t1           chr2     2289870   2290126         257
## 37          g3353.t1           chr2     2287920   2288086         167
## 38          g3353.t1           chr2     2288998   2289174         177
## 39          g3353.t1           chr2     2285456   2285627         172
## 40          g3353.t1           chr2     2285188   2285303         116
## 41          g3353.t1           chr2     2282961   2284548        1588
## 42              <NA>           chr2     2281760   2282066         307
## 43          g3354.t1           chr2     2279412   2279670         259
## 44          g3354.t1           chr2     2280385   2280692         308
## 45          g3354.t1           chr1    13642675  13643204         530
## 46          g3355.t1           chr2     2275739   2276911        1173
## 47          g3355.t1           chr2     2274650   2275361         712
## 48              <NA>           chr2     2272732   2272851         120
##    query.strand
## 1             *
## 2             *
## 3             *
## 4             *
## 5             *
## 6             *
## 7             *
## 8             *
## 9             *
## 10            *
## 11            *
## 12            *
## 13            *
## 14            *
## 15            *
## 16            *
## 17            *
## 18            *
## 19            *
## 20            *
## 21            *
## 22            *
## 23            *
## 24            *
## 25            *
## 26            *
## 27            *
## 28            *
## 29            *
## 30            *
## 31            *
## 32            *
## 33            *
## 34            *
## 35            *
## 36            *
## 37            *
## 38            *
## 39            *
## 40            *
## 41            *
## 42            *
## 43            *
## 44            *
## 45            *
## 46            *
## 47            *
## 48            *
```

### Hox 1

HOG `N19.HOG0000193`.

```
sapply(orthoPairs[1:15], \(gb) {
  x <- gb |> plyranges::filter(HOG == "N19.HOG0000193")
  if (length(x) == 0) return(NULL)
  x})
```

```
## $Oki_Osa
## GBreaks object with 1 range and 9 metadata columns:
##            seqnames            ranges strand |     tx_id     tx_name
##               <Rle>         <IRanges>  <Rle> | <integer> <character>
##   g8043.t1     chr2 14331701-14339427      * |      9021    g8043.t1
##            dNdS_GUIDANCE2 dNdS_HmmCleaner dNdS_PRANK                  query
##                 <numeric>       <numeric>  <numeric>              <GRanges>
##   g8043.t1             NA              NA    0.06616 Chr2:11876406-11881984
##                       HOG          OG      Arm
##               <character> <character> <factor>
##   g8043.t1 N19.HOG0000193   OG0000004     long
##   -------
##   seqinfo: 19 sequences from OKI2018.I69 genome
## 
## $Oki_Bar
## GBreaks object with 1 range and 9 metadata columns:
##            seqnames            ranges strand |     tx_id     tx_name
##               <Rle>         <IRanges>  <Rle> | <integer> <character>
##   g8043.t1     chr2 14331701-14339427      * |      9021    g8043.t1
##            dNdS_GUIDANCE2 dNdS_HmmCleaner dNdS_PRANK                query
##                 <numeric>       <numeric>  <numeric>            <GRanges>
##   g8043.t1             NA              NA    0.06616 Chr2:6198535-6201003
##                       HOG          OG      Arm
##               <character> <character> <factor>
##   g8043.t1 N19.HOG0000193   OG0000004     long
##   -------
##   seqinfo: 19 sequences from OKI2018.I69 genome
## 
## $Oki_Kum
## GBreaks object with 1 range and 9 metadata columns:
##            seqnames            ranges strand |     tx_id     tx_name
##               <Rle>         <IRanges>  <Rle> | <integer> <character>
##   g8043.t1     chr2 14331701-14339427      * |      9021    g8043.t1
##            dNdS_GUIDANCE2 dNdS_HmmCleaner dNdS_PRANK
##                 <numeric>       <numeric>  <numeric>
##   g8043.t1             NA              NA    0.06616
##                                  query            HOG          OG      Arm
##                              <GRanges>    <character> <character> <factor>
##   g8043.t1 contig_81_1:1615872-1623410 N19.HOG0000193   OG0000004     long
##   -------
##   seqinfo: 19 sequences from OKI2018.I69 genome
## 
## $Oki_Aom
## GBreaks object with 1 range and 9 metadata columns:
##            seqnames            ranges strand |     tx_id     tx_name
##               <Rle>         <IRanges>  <Rle> | <integer> <character>
##   g8043.t1     chr2 14331701-14339427      * |      9021    g8043.t1
##            dNdS_GUIDANCE2 dNdS_HmmCleaner dNdS_PRANK                     query
##                 <numeric>       <numeric>  <numeric>                 <GRanges>
##   g8043.t1             NA              NA    0.06616 contig_22_1:289322-292719
##                       HOG          OG      Arm
##               <character> <character> <factor>
##   g8043.t1 N19.HOG0000193   OG0000004     long
##   -------
##   seqinfo: 19 sequences from OKI2018.I69 genome
## 
## $Oki_Nor
## GBreaks object with 1 range and 9 metadata columns:
##            seqnames            ranges strand |     tx_id     tx_name
##               <Rle>         <IRanges>  <Rle> | <integer> <character>
##   g8043.t1     chr2 14331701-14339427      * |      9021    g8043.t1
##            dNdS_GUIDANCE2 dNdS_HmmCleaner dNdS_PRANK                    query
##                 <numeric>       <numeric>  <numeric>                <GRanges>
##   g8043.t1             NA              NA    0.06616 scaffold_9:221719-225168
##                       HOG          OG      Arm
##               <character> <character> <factor>
##   g8043.t1 N19.HOG0000193   OG0000004     long
##   -------
##   seqinfo: 19 sequences from OKI2018.I69 genome
## 
## $Osa_Bar
## GBreaks object with 1 range and 9 metadata columns:
##            seqnames            ranges strand |     tx_id     tx_name
##               <Rle>         <IRanges>  <Rle> | <integer> <character>
##   g6520.t1     Chr2 11876406-11881984      * |      7447    g6520.t1
##            dNdS_GUIDANCE2 dNdS_HmmCleaner dNdS_PRANK                query
##                 <numeric>       <numeric>  <numeric>            <GRanges>
##   g6520.t1             NA              NA    0.06616 Chr2:6198535-6201003
##                       HOG          OG      Arm
##               <character> <character> <factor>
##   g6520.t1 N19.HOG0000193   OG0000004     long
##   -------
##   seqinfo: 483 sequences from OSKA2016v1.9 genome
## 
## $Osa_Oki
## GBreaks object with 1 range and 9 metadata columns:
##            seqnames            ranges strand |     tx_id     tx_name
##               <Rle>         <IRanges>  <Rle> | <integer> <character>
##   g6520.t1     Chr2 11876406-11881984      * |      7447    g6520.t1
##            dNdS_GUIDANCE2 dNdS_HmmCleaner dNdS_PRANK                  query
##                 <numeric>       <numeric>  <numeric>              <GRanges>
##   g6520.t1             NA              NA    0.06616 chr2:14331701-14339427
##                       HOG          OG      Arm
##               <character> <character> <factor>
##   g6520.t1 N19.HOG0000193   OG0000004     long
##   -------
##   seqinfo: 483 sequences from OSKA2016v1.9 genome
## 
## $Osa_Kum
## GBreaks object with 1 range and 9 metadata columns:
##            seqnames            ranges strand |     tx_id     tx_name
##               <Rle>         <IRanges>  <Rle> | <integer> <character>
##   g6520.t1     Chr2 11876406-11881984      * |      7447    g6520.t1
##            dNdS_GUIDANCE2 dNdS_HmmCleaner dNdS_PRANK
##                 <numeric>       <numeric>  <numeric>
##   g6520.t1             NA              NA    0.06616
##                                  query            HOG          OG      Arm
##                              <GRanges>    <character> <character> <factor>
##   g6520.t1 contig_81_1:1615872-1623410 N19.HOG0000193   OG0000004     long
##   -------
##   seqinfo: 483 sequences from OSKA2016v1.9 genome
## 
## $Osa_Aom
## GBreaks object with 1 range and 9 metadata columns:
##            seqnames            ranges strand |     tx_id     tx_name
##               <Rle>         <IRanges>  <Rle> | <integer> <character>
##   g6520.t1     Chr2 11876406-11881984      * |      7447    g6520.t1
##            dNdS_GUIDANCE2 dNdS_HmmCleaner dNdS_PRANK                     query
##                 <numeric>       <numeric>  <numeric>                 <GRanges>
##   g6520.t1             NA              NA    0.06616 contig_22_1:289322-292719
##                       HOG          OG      Arm
##               <character> <character> <factor>
##   g6520.t1 N19.HOG0000193   OG0000004     long
##   -------
##   seqinfo: 483 sequences from OSKA2016v1.9 genome
## 
## $Osa_Nor
## GBreaks object with 1 range and 9 metadata columns:
##            seqnames            ranges strand |     tx_id     tx_name
##               <Rle>         <IRanges>  <Rle> | <integer> <character>
##   g6520.t1     Chr2 11876406-11881984      * |      7447    g6520.t1
##            dNdS_GUIDANCE2 dNdS_HmmCleaner dNdS_PRANK                    query
##                 <numeric>       <numeric>  <numeric>                <GRanges>
##   g6520.t1             NA              NA    0.06616 scaffold_9:221719-225168
##                       HOG          OG      Arm
##               <character> <character> <factor>
##   g6520.t1 N19.HOG0000193   OG0000004     long
##   -------
##   seqinfo: 483 sequences from OSKA2016v1.9 genome
## 
## $Bar_Osa
## GBreaks object with 1 range and 9 metadata columns:
##            seqnames          ranges strand |     tx_id     tx_name
##               <Rle>       <IRanges>  <Rle> | <integer> <character>
##   g4409.t1     Chr2 6198535-6201003      * |      3884    g4409.t1
##            dNdS_GUIDANCE2 dNdS_HmmCleaner dNdS_PRANK                  query
##                 <numeric>       <numeric>  <numeric>              <GRanges>
##   g4409.t1             NA              NA    0.06616 Chr2:11876406-11881984
##                       HOG          OG      Arm
##               <character> <character> <factor>
##   g4409.t1 N19.HOG0000193   OG0000004     long
##   -------
##   seqinfo: 68 sequences from Bar2.p4 genome
## 
## $Bar_Oki
## GBreaks object with 1 range and 9 metadata columns:
##            seqnames          ranges strand |     tx_id     tx_name
##               <Rle>       <IRanges>  <Rle> | <integer> <character>
##   g4409.t1     Chr2 6198535-6201003      * |      3884    g4409.t1
##            dNdS_GUIDANCE2 dNdS_HmmCleaner dNdS_PRANK                  query
##                 <numeric>       <numeric>  <numeric>              <GRanges>
##   g4409.t1             NA              NA    0.06616 chr2:14331701-14339427
##                       HOG          OG      Arm
##               <character> <character> <factor>
##   g4409.t1 N19.HOG0000193   OG0000004     long
##   -------
##   seqinfo: 68 sequences from Bar2.p4 genome
## 
## $Bar_Kum
## GBreaks object with 1 range and 9 metadata columns:
##            seqnames          ranges strand |     tx_id     tx_name
##               <Rle>       <IRanges>  <Rle> | <integer> <character>
##   g4409.t1     Chr2 6198535-6201003      * |      3884    g4409.t1
##            dNdS_GUIDANCE2 dNdS_HmmCleaner dNdS_PRANK
##                 <numeric>       <numeric>  <numeric>
##   g4409.t1             NA              NA    0.06616
##                                  query            HOG          OG      Arm
##                              <GRanges>    <character> <character> <factor>
##   g4409.t1 contig_81_1:1615872-1623410 N19.HOG0000193   OG0000004     long
##   -------
##   seqinfo: 68 sequences from Bar2.p4 genome
## 
## $Bar_Aom
## GBreaks object with 1 range and 9 metadata columns:
##            seqnames          ranges strand |     tx_id     tx_name
##               <Rle>       <IRanges>  <Rle> | <integer> <character>
##   g4409.t1     Chr2 6198535-6201003      * |      3884    g4409.t1
##            dNdS_GUIDANCE2 dNdS_HmmCleaner dNdS_PRANK                     query
##                 <numeric>       <numeric>  <numeric>                 <GRanges>
##   g4409.t1             NA              NA    0.06616 contig_22_1:289322-292719
##                       HOG          OG      Arm
##               <character> <character> <factor>
##   g4409.t1 N19.HOG0000193   OG0000004     long
##   -------
##   seqinfo: 68 sequences from Bar2.p4 genome
## 
## $Bar_Nor
## GBreaks object with 1 range and 9 metadata columns:
##            seqnames          ranges strand |     tx_id     tx_name
##               <Rle>       <IRanges>  <Rle> | <integer> <character>
##   g4409.t1     Chr2 6198535-6201003      * |      3884    g4409.t1
##            dNdS_GUIDANCE2 dNdS_HmmCleaner dNdS_PRANK                    query
##                 <numeric>       <numeric>  <numeric>                <GRanges>
##   g4409.t1             NA              NA    0.06616 scaffold_9:221719-225168
##                       HOG          OG      Arm
##               <character> <character> <factor>
##   g4409.t1 N19.HOG0000193   OG0000004     long
##   -------
##   seqinfo: 68 sequences from Bar2.p4 genome
```

```
Hox1 <- orthoPairs$Oki_Osa |> plyranges::filter(HOG == "N19.HOG0000193")
Hox1_1e4 <- GBreaks(target = granges(Hox1) + 1e4, query = Hox1$query + 1e4)
Hox1_4e4 <- GBreaks(target = granges(Hox1) + 4e4, query = Hox1$query + 4e4)
Hox1_1e5 <- GBreaks(target = granges(Hox1) + 1e5, query = Hox1$query + 1e5)

subsetByOverlaps(coa$Oki_Osa, Hox1_1e4) |> plotApairOfChrs("chr2", xlim = gb2xlim(Hox1_1e4))
```

```
subsetByOverlaps(coa$Oki_Osa, Hox1_4e4) |> plotApairOfChrs("chr2", xlim = gb2xlim(Hox1_4e4))
```

```
subsetByOverlaps(coa$Oki_Osa, Hox1_1e5) |> plotApairOfChrs("chr2", xlim = gb2xlim(Hox1_1e5))
```

```
## Genes
Hox1_4e4.genes <- transcripts$OKI2018.I69 |> subsetByOverlaps(Hox1_4e4 |> range())
mids <- Hox1_4e4.genes |> gr2dna_seg() |> dplyr::mutate(mid = (start + end) / 2) |> dplyr::pull(mid)
text <- Hox1_4e4.genes$tx_name |> as.character()
annot <- genoPlotR::annotation(x1=start(Hox1_4e4.genes), x2=end(Hox1_4e4.genes), text=text, rot=30)
gbs$Oki_Osa |> plotApairOfChrs("chr2", xlim = gb2xlim(Hox1_4e4), annotations = annot)
```

Click to see details on where the non-plotted alignments go.

```
subsetByOverlaps(gbs$Oki_Osa, granges(Hox1_4e4)) |> sort(i=T)  |> data.frame()
```

```
##    seqnames    start      end width strand score query.seqnames query.start
## 1      chr2 14291485 14301205  9721      + 14583           Chr2    11840898
## 2      chr2 14301496 14301975   480      +  1238           Chr2    11850325
## 3      chr2 14302003 14303041  1039      +  1772           Chr2    11851025
## 4      chr2 14303396 14308700  5305      + 12924           Chr2    11852392
## 5      chr2 14308706 14309700   995      -  1745            XSR     6558097
## 6      chr2 14309745 14316019  6275      + 12337           Chr2    11858653
## 7      chr2 14317283 14318281   999      +  1524           Chr2    11865528
## 8      chr2 14318553 14318967   415      +   546           Chr2    11866673
## 9      chr2 14319031 14323935  4905      +  9635           Chr2    11867661
## 10     chr2 14324459 14325534  1076      +  2647           Chr2    11872354
## 11     chr2 14326826 14327212   387      +   836           Chr2    11873646
## 12     chr2 14328382 14329239   858      +  1599           Chr2    11874085
## 13     chr2 14329493 14329772   280      +   595           Chr2    11875043
## 14     chr2 14330549 14330942   394      +   944           Chr2    11875452
## 15     chr2 14331321 14332339  1019      +  2206           Chr2    11876062
## 16     chr2 14332623 14333178   556      +  1436           Chr2    11877195
## 17     chr2 14333407 14335057  1651      +  3720           Chr2    11877808
## 18     chr2 14335316 14335647   332      +   960           Chr2    11879702
## 19     chr2 14337548 14340877  3330      +  4593           Chr2    11880320
## 20     chr2 14342948 14343636   689      +  2119           Chr2    11883383
## 21     chr2 14343877 14344887  1011      +  1286           Chr2    11884223
## 22     chr2 14345125 14346591  1467      +  2249           Chr2    11885434
## 23     chr2 14347027 14350252  3226      +  5426           Chr2    11887227
## 24     chr2 14350415 14351050   636      +   569           Chr2    11891522
## 25     chr2 14358965 14360158  1194      +  2046           Chr2    10290400
## 26     chr2 14366037 14367427  1391      +  2092           Chr2    10291647
## 27     chr2 14367524 14367889   366      -   637           Chr2    10446531
## 28     chr2 14367996 14369921  1926      -  3010           Chr2    10443655
## 29     chr2 14370767 14371196   430      -   615           Chr2    10442957
## 30     chr2 14371255 14371688   434      -   669           Chr2    10441657
##    query.end query.width query.strand query.Arm query.rep query.repOvlp
## 1   11850128        9231            *      long    tandem            26
## 2   11850830         506            *      long        NA             0
## 3   11852070        1046            *      long       rnd             6
## 4   11857551        5160            *      long        NA             0
## 5    6559035         939            *       XSR        NA             0
## 6   11864754        6102            *      long        NA             0
## 7   11866483         956            *      long        NA             0
## 8   11867070         398            *      long        NA             0
## 9   11872352        4692            *      long        NA             0
## 10  11873433        1080            *      long        NA             0
## 11  11874035         390            *      long        NA             0
## 12  11875033         949            *      long        NA             0
## 13  11875304         262            *      long        NA             0
## 14  11875839         388            *      long        NA             0
## 15  11876969         908            *      long        NA             0
## 16  11877761         567            *      long        NA             0
## 17  11879404        1597            *      long        NA             0
## 18  11880036         335            *      long        NA             0
## 19  11883221        2902            *      long        NA             0
## 20  11884079         697            *      long        NA             0
## 21  11885210         988            *      long        NA             0
## 22  11886873        1440            *      long        NA             0
## 23  11890368        3142            *      long    tandem            34
## 24  11892136         615            *      long        NA             0
## 25  10291593        1194            *      long        NA             0
## 26  10293038        1392            *      long    tandem            23
## 27  10446893         363            *      long        NA             0
## 28  10445529        1875            *      long        NA             0
## 29  10443440         484            *      long        NA             0
## 30  10442052         396            *      long        NA             0
##             query.transcripts  Arm          rep repOvlp
## 1  g6508.t1;g6509.t1;g6510.t1 long       tandem      42
## 2                    g6511.t1 long           NA       0
## 3                    g6511.t1 long           NA       0
## 4  g6512.t1;g6513.t1;g6514.t1 long LowCompl....      48
## 5                   g13445.t1 long           NA       0
## 6           g6516.t1;g6516.t2 long           NA       0
## 7                    g6517.t1 long           NA       0
## 8                    g6517.t1 long           NA       0
## 9  g6517.t1;g6518.t1;g6519.t1 long           NA       0
## 10                   g6519.t1 long           NA       0
## 11                       <NA> long           NA       0
## 12                       <NA> long          rnd       0
## 13                       <NA> long       tandem      25
## 14                       <NA> long           NA       0
## 15                   g6520.t1 long           NA       0
## 16                   g6520.t1 long           NA       0
## 17                   g6520.t1 long           NA       0
## 18                   g6520.t1 long           NA       0
## 19                   g6520.t1 long      unknown     101
## 20                   g6521.t2 long           NA       0
## 21 g6521.t2;g6521.t1;g6522.t1 long           NA       0
## 22                   g6522.t1 long           NA       0
## 23          g6523.t1;g6523.t2 long           NA       0
## 24                       <NA> long           NA       0
## 25                       <NA> long           NA       0
## 26                       <NA> long           NA       0
## 27          g6048.t1;g6048.t2 long           NA       0
## 28 g6047.t1;g6048.t1;g6048.t2 long       tandem       0
## 29                   g6046.t1 long           NA       0
## 30                       <NA> long           NA       0
##                                     transcripts flag nonCoa
## 1  g8029.t1;g8030.t1;g8031.t1;g8032.t1;g8033.t1  Col  FALSE
## 2                                      g8034.t1  Col  FALSE
## 3                                      g8034.t1  Col  FALSE
## 4                             g8034.t1;g8035.t1  Tra  FALSE
## 5                                          <NA> <NA>   TRUE
## 6                                      g8037.t1  Col  FALSE
## 7                                          <NA>  Col  FALSE
## 8                                          <NA>  Col  FALSE
## 9                             g8039.t1;g8040.t1  Col  FALSE
## 10                                         <NA>  Col  FALSE
## 11                                         <NA>  Col  FALSE
## 12                                         <NA>  Col  FALSE
## 13                                         <NA>  Col  FALSE
## 14                                         <NA>  Col  FALSE
## 15                                         <NA>  Col  FALSE
## 16                                         <NA>  Col  FALSE
## 17                                         <NA>  Col  FALSE
## 18                                         <NA>  Col  FALSE
## 19                                         <NA>  Col  FALSE
## 20                                     g8044.t1  Col  FALSE
## 21                                     g8045.t1  Col  FALSE
## 22                                     g8045.t1  Col  FALSE
## 23                            g8046.t1;g8046.t2  Col  FALSE
## 24                                         <NA> <NA>  FALSE
## 25                                     g8048.t1  Col  FALSE
## 26                                     g8051.t1 <NA>  FALSE
## 27                                     g8052.t1  Col  FALSE
## 28                                     g8052.t1  Col  FALSE
## 29                                         <NA>  Col  FALSE
## 30                                         <NA> <NA>  FALSE
```

```
subsetByOverlaps(swap(gbs$Oki_Osa), granges(swap(Hox1_4e4))) |> sort(i=T)  |> data.frame()
```

```
##    seqnames    start      end width strand  Arm    rep repOvlp
## 1      Chr2 11836695 11836910   216      + long     NA       0
## 2      Chr2 11837950 11838286   337      + long     NA       0
## 3      Chr2 11838557 11839782  1226      + long     NA       0
## 4      Chr2 11839855 11840506   652      + long     NA       0
## 5      Chr2 11840898 11850128  9231      + long tandem      26
## 6      Chr2 11850325 11850830   506      + long     NA       0
## 7      Chr2 11851025 11852070  1046      + long    rnd       6
## 8      Chr2 11852392 11857551  5160      + long     NA       0
## 9      Chr2 11858653 11864754  6102      + long     NA       0
## 10     Chr2 11865528 11866483   956      + long     NA       0
## 11     Chr2 11866673 11867070   398      + long     NA       0
## 12     Chr2 11867661 11872352  4692      + long     NA       0
## 13     Chr2 11872354 11873433  1080      + long     NA       0
## 14     Chr2 11873646 11874035   390      + long     NA       0
## 15     Chr2 11874085 11875033   949      + long     NA       0
## 16     Chr2 11875043 11875304   262      + long     NA       0
## 17     Chr2 11875452 11875839   388      + long     NA       0
## 18     Chr2 11876062 11876969   908      + long     NA       0
## 19     Chr2 11877195 11877761   567      + long     NA       0
## 20     Chr2 11877808 11879404  1597      + long     NA       0
## 21     Chr2 11879702 11880036   335      + long     NA       0
## 22     Chr2 11880320 11883221  2902      + long     NA       0
## 23     Chr2 11883383 11884079   697      + long     NA       0
## 24     Chr2 11884223 11885210   988      + long     NA       0
## 25     Chr2 11885434 11886873  1440      + long     NA       0
## 26     Chr2 11887227 11890368  3142      + long tandem      34
## 27     Chr2 11891522 11892136   615      + long     NA       0
## 28     Chr2 11892246 11895803  3558      - long     NA       0
## 29     Chr2 11898001 11904292  6292      - long tandem      17
## 30     Chr2 11904831 11905119   289      - long     NA       0
## 31     Chr2 11905364 11908894  3531      - long tandem      35
## 32     Chr2 11908930 11909902   973      - long     NA       0
## 33     Chr2 11910312 11911039   728      - long     NA       0
## 34     Chr2 11911499 11915152  3654      - long     NA       0
## 35     Chr2 11915518 11916015   498      - long     NA       0
## 36     Chr2 11916043 11916133    91      - long     NA       0
## 37     Chr2 11916531 11916876   346      - long tandem      50
## 38     Chr2 11917362 11917452    91      - long     NA       0
## 39     Chr2 11918407 11921078  2672      - long     NA       0
## 40     Chr2 11921113 11924324  3212      - long     NA       0
##                                     transcripts query.seqnames query.start
## 1                                          <NA>           chr2    14285932
## 2                                          <NA>           chr2    14287358
## 3                                      g6508.t1           chr2    14288200
## 4                                      g6508.t1           chr2    14289936
## 5                    g6508.t1;g6509.t1;g6510.t1           chr2    14291485
## 6                                      g6511.t1           chr2    14301496
## 7                                      g6511.t1           chr2    14302003
## 8                    g6512.t1;g6513.t1;g6514.t1           chr2    14303396
## 9                             g6516.t1;g6516.t2           chr2    14309745
## 10                                     g6517.t1           chr2    14317283
## 11                                     g6517.t1           chr2    14318553
## 12                   g6517.t1;g6518.t1;g6519.t1           chr2    14319031
## 13                                     g6519.t1           chr2    14324459
## 14                                         <NA>           chr2    14326826
## 15                                         <NA>           chr2    14328382
## 16                                         <NA>           chr2    14329493
## 17                                         <NA>           chr2    14330549
## 18                                     g6520.t1           chr2    14331321
## 19                                     g6520.t1           chr2    14332623
## 20                                     g6520.t1           chr2    14333407
## 21                                     g6520.t1           chr2    14335316
## 22                                     g6520.t1           chr2    14337548
## 23                                     g6521.t2           chr2    14342948
## 24                   g6521.t2;g6521.t1;g6522.t1           chr2    14343877
## 25                                     g6522.t1           chr2    14345125
## 26                            g6523.t1;g6523.t2           chr2    14347027
## 27                                         <NA>           chr2    14350415
## 28                   g6524.t1;g6524.t2;g6524.t3           chr1     4306180
## 29                            g6528.t1;g6529.t1           chr2    12400690
## 30                                         <NA>           chr2    12399778
## 31 g6531.t1;g6532.t1;g6533.t1;g6534.t1;g6534.t2           chr2    12396262
## 32                            g6534.t1;g6534.t2           chr2    12395020
## 33                            g6534.t1;g6534.t2           chr2    12392902
## 34                   g6534.t1;g6534.t2;g6535.t1           chr2    12388523
## 35                                         <NA>           chr2    12388067
## 36                                         <NA>           chr2    12387612
## 37                                         <NA>           chr2    12386629
## 38                                         <NA>           chr2    12385932
## 39                                     g6536.t1           chr2    12382223
## 40          g6536.t1;g6537.t1;g6537.t2;g6538.t1           chr2    12378062
##    query.end query.width query.strand
## 1   14286139         208            *
## 2   14287714         357            *
## 3   14289564        1365            *
## 4   14290622         687            *
## 5   14301205        9721            *
## 6   14301975         480            *
## 7   14303041        1039            *
## 8   14308700        5305            *
## 9   14316019        6275            *
## 10  14318281         999            *
## 11  14318967         415            *
## 12  14323935        4905            *
## 13  14325534        1076            *
## 14  14327212         387            *
## 15  14329239         858            *
## 16  14329772         280            *
## 17  14330942         394            *
## 18  14332339        1019            *
## 19  14333178         556            *
## 20  14335057        1651            *
## 21  14335647         332            *
## 22  14340877        3330            *
## 23  14343636         689            *
## 24  14344887        1011            *
## 25  14346591        1467            *
## 26  14350252        3226            *
## 27  14351050         636            *
## 28   4309907        3728            *
## 29  12407280        6591            *
## 30  12400072         295            *
## 31  12399665        3404            *
## 32  12396031        1012            *
## 33  12393798         897            *
## 34  12392467        3945            *
## 35  12388501         435            *
## 36  12387706          95            *
## 37  12386995         367            *
## 38  12386031         100            *
## 39  12384947        2725            *
## 40  12381443        3382            *
```

## Ancestral states

Prototype 1

In the concept case below, B has moved in the North Atlantic
clade.

- Oki: A-B-C
- Osa: A-B-C
- Bar: A-C, and B can be found somewhere else

As we work on alignment pairs, this translates to:

- Oki\_Osa: A-B-C
- Oki\_Bar: A, B, C

To find such cases I search for Oki-Bar orthoblock breakpoints that
fall within an Oki-Osa orthoblock.

```
reIndex <- \(gb) {names(gb) <- seq_along(gb) ; gb}
midBreaks <- sapply(orthoBlocks_one2oneInOiks, get_bps, direction = "mid") |> SimpleList()

ROIs <- SimpleList()
findROIs <- function(g1, g2, g3) {
  pair1_2 <- paste(g1, sep = '_', g2)
  pair1_3 <- paste(g1, sep = '_', g3)
  gb <- orthoBlocks_one2oneInOiks[[pair1_2]] |>
    reIndex() |>
    subsetByOverlaps(midBreaks[[pair1_3]]) |>
    subset(geneNumber > 2)
  # Drop columns to ease browsing on single lines.
  NULL -> gb$dNdS_GUIDANCE2 -> gb$dNdS_HmmCleaner -> gb$dNdS_PRANK
  gb
}
ROIs$Oki_Osa_Bar <- findROIs("Oki", "Osa", "Bar")
ROIs$Oki_Bar_Osa <- findROIs("Oki", "Bar", "Osa")
ROIs$Oki_Aom_Nor <- findROIs("Oki", "Aom", "Nor")

findGene <- function(gb, gene) {
  if(is.null(gb$geneNames)) {
    ids <- gb$tx_name
    index <- gb$tx_name %in% gene
  } else {
    ids <- gb$geneNames
    index <- any(ids %in% gene)
  }
  gb[index]
}

# Genes of Interest

(GoI1 <- head(ROIs$Oki_Osa_Bar$geneNames, 1) |> unlist())
```

```
## [1] "g1206.t1" "g1207.t1" "g1211.t1"
```

```
GoI2 <- c("g1205.t1", "g1208.t1", "g1209.t1", "g1210.t1") # To provide context

# Exploring the region

orthoBlocks$Oki_Osa |> findGene(c(GoI1, GoI2)) # g1206.t1 g1207.t1 g1208.t1 g1211.t1 are in chr1:4573346-4588029 | Chr1:402525-416983
```

```
## GBreaks object with 1 range and 9 metadata columns:
##       seqnames          ranges strand |              query     score
##          <Rle>       <IRanges>  <Rle> |          <GRanges> <integer>
##   [1]     chr1 4573346-4588029      * | Chr1:402525-416983     14684
##                            geneNames
##                      <CharacterList>
##   [1] g1206.t1,g1207.t1,g1208.t1,...
##                                                   HOGs dNdS_GUIDANCE2
##                                        <CharacterList>  <NumericList>
##   [1] N19.HOG0003732,N19.HOG0010718,N19.HOG0015251,...   NA,NA,NA,...
##       dNdS_HmmCleaner                  dNdS_PRANK geneNumber      Arm
##         <NumericList>               <NumericList>  <integer> <factor>
##   [1]    NA,NA,NA,... 0.06413,0.05353,     NA,...          4    short
##   -------
##   seqinfo: 19 sequences from OKI2018.I69 genome
```

```
orthoPairs $Oki_Osa |> findGene(c(GoI1, GoI2)) |> as.data.frame() # Individual coordinates of the genes
```

```
##          seqnames   start     end width strand tx_id  tx_name dNdS_GUIDANCE2
## g1206.t1     chr1 4573346 4575265  1920      *   631 g1206.t1             NA
## g1207.t1     chr1 4575321 4577337  2017      *  2777 g1207.t1             NA
## g1208.t1     chr1 4577995 4578612   618      *   632 g1208.t1             NA
## g1211.t1     chr1 4581484 4588029  6546      *  2778 g1211.t1             NA
##          dNdS_HmmCleaner dNdS_PRANK query.seqnames query.start query.end
## g1206.t1              NA    0.06413           Chr1      414718    416983
## g1207.t1              NA    0.05353           Chr1      410741    413759
## g1208.t1              NA         NA           Chr1      409351    409953
## g1211.t1              NA    0.03702           Chr1      402525    406012
##          query.width query.strand query.tx_id query.tx_name
## g1206.t1        2266            *        1722        g96.t1
## g1207.t1        3019            *          52        g95.t1
## g1208.t1         603            *        1721        g94.t1
## g1211.t1        3488            *          50        g92.t1
##          query.dNdS_GUIDANCE2 query.dNdS_HmmCleaner query.dNdS_PRANK
## g1206.t1                   NA                    NA          0.06413
## g1207.t1                   NA                    NA          0.05353
## g1208.t1                   NA                    NA               NA
## g1211.t1                   NA                    NA          0.03702
##                     HOG        OG   Arm
## g1206.t1 N19.HOG0003732 OG0000664 short
## g1207.t1 N19.HOG0010718 OG0006871 short
## g1208.t1 N19.HOG0015251 OG0016820 short
## g1211.t1 N19.HOG0008357 OG0003884 short
```

```
orthoBlocks$Oki_Bar |> findGene(c(GoI1, GoI2)) # g1205.t1 g1206.t1 g1207.t1 g1208.t1 g1211.t1 are in chr1 4571907-4588029 | Chr1:582810-2680665
```

```
## GBreaks object with 3 ranges and 9 metadata columns:
##       seqnames          ranges strand |                query     score
##          <Rle>       <IRanges>  <Rle> |            <GRanges> <integer>
##   [1]     chr1 4571907-4573262      * |   Chr1:582810-583163      1356
##   [2]     chr1 4573346-4577337      * | Chr1:2669327-2674502      3992
##   [3]     chr1 4577995-4588029      * | Chr1:2675030-2680665     10035
##               geneNames                          HOGs dNdS_GUIDANCE2
##         <CharacterList>               <CharacterList>  <NumericList>
##   [1]          g1205.t1                N19.HOG0002360             NA
##   [2] g1206.t1,g1207.t1 N19.HOG0003732,N19.HOG0010718          NA,NA
##   [3] g1208.t1,g1211.t1 N19.HOG0015251,N19.HOG0008357          NA,NA
##       dNdS_HmmCleaner      dNdS_PRANK geneNumber      Arm
##         <NumericList>   <NumericList>  <integer> <factor>
##   [1]              NA              NA          1    short
##   [2]           NA,NA 0.06413,0.05353          2    short
##   [3]           NA,NA      NA,0.03702          2    short
##   -------
##   seqinfo: 19 sequences from OKI2018.I69 genome
```

```
orthoPairs $Oki_Bar |> findGene(c(GoI1, GoI2)) |> as.data.frame() # Individual coordinates of the genes
```

```
##          seqnames   start     end width strand tx_id  tx_name dNdS_GUIDANCE2
## g1205.t1     chr1 4571907 4573262  1356      *   630 g1205.t1             NA
## g1206.t1     chr1 4573346 4575265  1920      *   631 g1206.t1             NA
## g1207.t1     chr1 4575321 4577337  2017      *  2777 g1207.t1             NA
## g1208.t1     chr1 4577995 4578612   618      *   632 g1208.t1             NA
## g1211.t1     chr1 4581484 4588029  6546      *  2778 g1211.t1             NA
##          dNdS_HmmCleaner dNdS_PRANK query.seqnames query.start query.end
## g1205.t1              NA         NA           Chr1      582810    583163
## g1206.t1              NA    0.06413           Chr1     2672887   2674502
## g1207.t1              NA    0.05353           Chr1     2669327   2672825
## g1208.t1              NA         NA           Chr1     2675030   2675328
## g1211.t1              NA    0.03702           Chr1     2678743   2680665
##          query.width query.strand query.tx_id query.tx_name
## g1205.t1         354            *        1566       g122.t1
## g1206.t1        1616            *        1842       g651.t1
## g1207.t1        3499            *         356       g650.t1
## g1208.t1         299            *         357       g652.t1
## g1211.t1        1923            *        1844       g654.t1
##          query.dNdS_GUIDANCE2 query.dNdS_HmmCleaner query.dNdS_PRANK
## g1205.t1                   NA                    NA               NA
## g1206.t1                   NA                    NA          0.06413
## g1207.t1                   NA                    NA          0.05353
## g1208.t1                   NA                    NA               NA
## g1211.t1                   NA                    NA          0.03702
##                     HOG        OG   Arm
## g1205.t1 N19.HOG0002360 OG0000249 short
## g1206.t1 N19.HOG0003732 OG0000664 short
## g1207.t1 N19.HOG0010718 OG0006871 short
## g1208.t1 N19.HOG0015251 OG0016820 short
## g1211.t1 N19.HOG0008357 OG0003884 short
```

```
orthoBlocks$Bar_Oki |> subsetByOverlaps(GRanges("Chr1:2669327-2680665")) |> swap()  # Let's forget about g1205.t1
```

```
## GBreaks object with 2 ranges and 5 metadata columns:
##       seqnames          ranges strand |         geneNames dNdS_GUIDANCE2
##          <Rle>       <IRanges>  <Rle> |   <CharacterList>  <NumericList>
##   [1]     chr1 4573346-4577337      * | g1207.t1,g1206.t1          NA,NA
##   [2]     chr1 4577995-4588029      * | g1208.t1,g1211.t1          NA,NA
##       dNdS_HmmCleaner      dNdS_PRANK                query
##         <NumericList>   <NumericList>            <GRanges>
##   [1]           NA,NA 0.05353,0.06413 Chr1:2669327-2674502
##   [2]           NA,NA      NA,0.03702 Chr1:2675030-2680665
##   -------
##   seqinfo: 19 sequences from OKI2018.I69 genome
```

```
orthoBlocks$Bar_Oki |> subsetByOverlaps(GRanges("Chr1:2669327-2680665")) |> swap() |> cleanGaps() |> width()
```

```
## [1] 657
```

```
Oki         Osa       Bar  
g1206.t1    g96.t1    g651.t1
g1207.t1    g95.t1    g650.t1
g1208.t1    g94.t1    g652.t1
g1211.t1    g92.t1    g654.t1
```

```
### Between "g1207.t1" and "g1208.t1", there is a repeat region, where the Bar genome broke, but that is still in the Osa genome (bri)

unal$Oki_Osa |> subsetByOverlaps(GRanges("chr1", (4577337 + 4577995)/2)) # between g1207.t1 and g1208.t1 on Oki
```

```
## GRanges object with 1 range and 4 metadata columns:
##       seqnames          ranges strand |      Arm             rep transcripts
##          <Rle>       <IRanges>  <Rle> | <factor> <CharacterList>       <Rle>
##   [1]     chr1 4577462-4577685      * |    short         unknown        <NA>
##          nonCoa
##       <logical>
##   [1]     FALSE
##   -------
##   seqinfo: 19 sequences from OKI2018.I69 genome
```

```
unal$Osa_Oki |> subsetByOverlaps(GRanges("Chr1", ( 409953 + 410741 )/2)) # between g95.t1 and g94.t1 on Osa
```

```
## GRanges object with 1 range and 4 metadata columns:
##       seqnames        ranges strand |      Arm             rep transcripts
##          <Rle>     <IRanges>  <Rle> | <factor> <CharacterList>       <Rle>
##   [1]     Chr1 410306-410620      * |    short     unknown,rnd        <NA>
##          nonCoa
##       <logical>
##   [1]     FALSE
##   -------
##   seqinfo: 483 sequences from OSKA2016v1.9 genome
```

```
# Or just:

(BP1_Oki_Osa <- bri$Oki_Osa |> subsetByOverlaps(GRanges("chr1", (4577337 + 4577995)/2))) # between g1207.t1 and g1208.t1 on Oki
```

```
## GBreaks object with 1 range and 5 metadata columns:
##       seqnames          ranges strand |              query      Arm
##          <Rle>       <IRanges>  <Rle> |          <GRanges> <factor>
##   [1]     chr1 4577462-4577685      * | Chr1:410306-410620    short
##                   rep   repOvlp transcripts
##       <CharacterList> <integer>       <Rle>
##   [1]         unknown       110        <NA>
##   -------
##   seqinfo: 19 sequences from OKI2018.I69 genome
```

```
# Now on Bar:
gbs$Bar_Oki |> reIndex()|> subsetByOverlaps(GRanges("Chr1", (2674502 + 2675030)/2)) # between g651.t1 and  g652.t1 on Bar
```

```
## GBreaks object with 1 range and 8 metadata columns:
##        seqnames          ranges strand |     score                query
##           <Rle>       <IRanges>  <Rle> | <numeric>            <GRanges>
##   2026     Chr1 2674688-2675041      + |       515 chr1:4577686-4578054
##             Arm             rep   repOvlp transcripts        flag    nonCoa
##        <factor> <CharacterList> <integer>       <Rle> <character> <logical>
##   2026    short            <NA>         0     g652.t1         Col     FALSE
##   -------
##   seqinfo: 68 sequences from Bar2.p4 genome
```

```
coa$Bar_Oki |> reIndex()|> subsetByOverlaps(GRanges("Chr1", (2674502 + 2675030)/2)) # between g651.t1 and  g652.t1 on Bar
```

```
## GBreaks object with 1 range and 8 metadata columns:
##       seqnames          ranges strand |                query     score      Arm
##          <Rle>       <IRanges>  <Rle> |            <GRanges> <integer> <factor>
##   893     Chr1 2674688-2680680      + | chr1:4577686-4588063      5993    short
##                   rep   repOvlp transcripts        flag    nonCoa
##       <CharacterList> <integer>       <Rle> <character> <logical>
##   893     unknown,rnd      1060     g652.t1        <NA>     FALSE
##   -------
##   seqinfo: 68 sequences from Bar2.p4 genome
```

```
(BP1_Bar <- unal$Bar_Oki |> subsetByOverlaps(GRanges("Chr1", (2674538 + 2674688)/2))) # between g651.t1 and  g652.t1 on Bar according to gbs or coa
```

```
## GRanges object with 1 range and 4 metadata columns:
##       seqnames          ranges strand |      Arm             rep transcripts
##          <Rle>       <IRanges>  <Rle> | <factor> <CharacterList>       <Rle>
##   [1]     Chr1 2674539-2674687      * |    short            <NA>        <NA>
##          nonCoa
##       <logical>
##   [1]      TRUE
##   -------
##   seqinfo: 68 sequences from Bar2.p4 genome
```

```
# Nothing noticeable on either strand
pairwiseAlignment(type ="local", BP1_Oki_Osa |> plyranges::mutate(strand = '-'))
```

```
## Local PairwiseAlignmentsSingleSubject (1 of 1)
## pattern: [164] TATTTTATT
## subject: [182] TATTTTATT
## score: 17.8358
```

```
pairwiseAlignment(type ="local", BP1_Oki_Osa |> plyranges::mutate(strand = '+'))
```

```
## Local PairwiseAlignmentsSingleSubject (1 of 1)
## pattern: [37] AAGATGGTCCAT
## subject: [23] AAGATTGTCCAT
## score: 15.90003
```

```
pairwiseAlignment(type ="local", BP1_Oki_Osa $query, BP1_Bar)
```

```
## Local PairwiseAlignmentsSingleSubject (1 of 1)
## pattern: [188] ATTTTTTC
## subject:  [31] ATTTTTTC
## score: 15.85405
```

```
pairwiseAlignment(type ="global", BP1_Oki_Osa $query, BP1_Bar  |> plyranges::mutate(strand = '-'))
```

```
## Global PairwiseAlignmentsSingleSubject (1 of 1)
## pattern: AGTTTGCGGGTAAAGTAACCAAAAGATTGTCCAT...ACCTAAATATTAGAGCCTTGAGATTTTCAGTGAC
## subject: AGTTAGTG----AAGAAACAATAA-ACTGTGAAT...ACCG-----------------GATTTTG------
## score: -867.5856
```

```
oP_one2oneInOiks_bridges$Oki_Osa |> subsetByOverlaps(GRanges("chr1", (4577337 + 4577995)/2))
```

```
## GBreaks object with 1 range and 1 metadata column:
##       seqnames          ranges strand |              query
##          <Rle>       <IRanges>  <Rle> |          <GRanges>
##   [1]     chr1 4577338-4581483      * | Chr1:413760-414717
##   -------
##   seqinfo: 19 sequences from OKI2018.I69 genome
```

I can not search for orthologs that are marked “Col” in Oki\_Osa and
“Tra” in Osa\_Bar, because the transposition flagging does not work on
strandless ranges.

```
a <- orthoBlocks$Oki_Osa$HOGs |> Filter(f = \(x) length(x) > 1)
b <- orthoBlocks$Oki_Bar$HOGs |> Filter(f = \(x) length(x) > 1)

makePairs.vector <- function(v) {
  L <- GenomicBreaks::zipWithNext(x) |> as("List")
  L <- L[1:(length(L) -1)]
  l <- lapply(L, \(x) paste(first(x), second(x)))
  unlist(l)
}
```

## Session information

```
sessionInfo()
```

```
## R version 4.3.1 (2023-06-16)
## Platform: x86_64-pc-linux-gnu (64-bit)
## Running under: Debian GNU/Linux 12 (bookworm)
## 
## Matrix products: default
## BLAS:   /usr/lib/x86_64-linux-gnu/blas/libblas.so.3.11.0 
## LAPACK: /usr/lib/x86_64-linux-gnu/lapack/liblapack.so.3.11.0
## 
## locale:
##  [1] LC_CTYPE=en_US.UTF-8       LC_NUMERIC=C              
##  [3] LC_TIME=en_US.UTF-8        LC_COLLATE=en_US.UTF-8    
##  [5] LC_MONETARY=en_US.UTF-8    LC_MESSAGES=en_US.UTF-8   
##  [7] LC_PAPER=en_US.UTF-8       LC_NAME=C                 
##  [9] LC_ADDRESS=C               LC_TELEPHONE=C            
## [11] LC_MEASUREMENT=en_US.UTF-8 LC_IDENTIFICATION=C       
## 
## time zone: Etc/UTC
## tzcode source: system (glibc)
## 
## attached base packages:
## [1] stats4    stats     graphics  grDevices utils     datasets  methods  
## [8] base     
## 
## other attached packages:
##  [1] BSgenome.Oidioi.genoscope.OdB3_1.0.0   
##  [2] BSgenome.Oidioi.OIST.AOM.5.5f_1.0.1    
##  [3] BSgenome.Oidioi.OIST.KUM.M3.7f_1.0.1   
##  [4] BSgenome.Oidioi.OIST.Bar2.p4_1.0.1     
##  [5] BSgenome.Oidioi.OIST.OSKA2016v1.9_1.0.0
##  [6] BSgenome.Oidioi.OIST.OKI2018.I69_1.0.1 
##  [7] patchwork_1.1.2                        
##  [8] OikScrambling_5.1.0                    
##  [9] ggplot2_3.4.4                          
## [10] GenomicBreaks_0.14.3                   
## [11] BSgenome_1.68.0                        
## [12] rtracklayer_1.60.1                     
## [13] Biostrings_2.68.1                      
## [14] XVector_0.40.0                         
## [15] GenomicRanges_1.52.1                   
## [16] GenomeInfoDb_1.36.4                    
## [17] IRanges_2.34.1                         
## [18] S4Vectors_0.38.2                       
## [19] BiocGenerics_0.46.0                    
## 
## loaded via a namespace (and not attached):
##   [1] splines_4.3.1               BiocIO_1.10.0              
##   [3] filelock_1.0.2              bitops_1.0-7               
##   [5] tibble_3.2.1                R.oo_1.25.0                
##   [7] XML_3.99-0.15               rpart_4.1.19               
##   [9] lifecycle_1.0.3             rprojroot_2.0.4            
##  [11] lattice_0.20-45             MASS_7.3-58.2              
##  [13] backports_1.4.1             magrittr_2.0.3             
##  [15] Hmisc_5.1-1                 sass_0.4.7                 
##  [17] rmarkdown_2.25              jquerylib_0.1.4            
##  [19] yaml_2.3.7                  plotrix_3.8-3              
##  [21] DBI_1.1.3                   CNEr_1.36.0                
##  [23] minqa_1.2.6                 RColorBrewer_1.1-3         
##  [25] ade4_1.7-22                 abind_1.4-5                
##  [27] zlibbioc_1.46.0             purrr_1.0.2                
##  [29] R.utils_2.12.2              RCurl_1.98-1.13            
##  [31] nnet_7.3-18                 pracma_2.4.2               
##  [33] rappdirs_0.3.3              GenomeInfoDbData_1.2.10    
##  [35] gdata_3.0.0                 annotate_1.78.0            
##  [37] pkgdown_2.0.7               codetools_0.2-19           
##  [39] DelayedArray_0.26.7         xml2_1.3.5                 
##  [41] tidyselect_1.2.0            shape_1.4.6                
##  [43] farver_2.1.1                lme4_1.1-35.1              
##  [45] BiocFileCache_2.8.0         matrixStats_1.0.0          
##  [47] base64enc_0.1-3             GenomicAlignments_1.36.0   
##  [49] jsonlite_1.8.7              mitml_0.4-5                
##  [51] Formula_1.2-5               survival_3.5-3             
##  [53] iterators_1.0.14            systemfonts_1.0.5          
##  [55] foreach_1.5.2               progress_1.2.2             
##  [57] tools_4.3.1                 ragg_1.2.5                 
##  [59] Rcpp_1.0.11                 glue_1.6.2                 
##  [61] gridExtra_2.3               pan_1.9                    
##  [63] xfun_0.41                   MatrixGenerics_1.12.3      
##  [65] EBImage_4.42.0              dplyr_1.1.3                
##  [67] withr_2.5.2                 fastmap_1.1.1              
##  [69] boot_1.3-28.1               fansi_1.0.5                
##  [71] digest_0.6.33               R6_2.5.1                   
##  [73] mice_3.16.0                 textshaping_0.3.7          
##  [75] colorspace_2.1-0            GO.db_3.17.0               
##  [77] gtools_3.9.4                poweRlaw_0.70.6            
##  [79] biomaRt_2.56.1              jpeg_0.1-10                
##  [81] RSQLite_2.3.3               weights_1.0.4              
##  [83] R.methodsS3_1.8.2           utf8_1.2.4                 
##  [85] tidyr_1.3.0                 generics_0.1.3             
##  [87] data.table_1.14.8           prettyunits_1.2.0          
##  [89] httr_1.4.7                  htmlwidgets_1.6.2          
##  [91] S4Arrays_1.0.6              pkgconfig_2.0.3            
##  [93] gtable_0.3.4                blob_1.2.4                 
##  [95] htmltools_0.5.7             fftwtools_0.9-11           
##  [97] plyranges_1.20.0            scales_1.2.1               
##  [99] Biobase_2.60.0              png_0.1-8                  
## [101] knitr_1.45                  heatmaps_1.24.0            
## [103] rstudioapi_0.15.0           tzdb_0.4.0                 
## [105] reshape2_1.4.4              rjson_0.2.21               
## [107] curl_5.1.0                  checkmate_2.3.0            
## [109] nlme_3.1-162                nloptr_2.0.3               
## [111] cachem_1.0.8                stringr_1.5.0              
## [113] KernSmooth_2.23-20          parallel_4.3.1             
## [115] genoPlotR_0.8.11            foreign_0.8-84             
## [117] AnnotationDbi_1.62.2        restfulr_0.0.15            
## [119] desc_1.4.2                  pillar_1.9.0               
## [121] grid_4.3.1                  vctrs_0.6.4                
## [123] jomo_2.7-6                  dbplyr_2.3.3               
## [125] xtable_1.8-4                cluster_2.1.4              
## [127] htmlTable_2.4.2             evaluate_0.23              
## [129] GenomicFeatures_1.52.1      readr_2.1.4                
## [131] cli_3.6.1                   locfit_1.5-9.8             
## [133] compiler_4.3.1              Rsamtools_2.16.0           
## [135] rlang_1.1.2                 crayon_1.5.2               
## [137] labeling_0.4.3              plyr_1.8.9                 
## [139] fs_1.6.3                    stringi_1.7.12             
## [141] BiocParallel_1.34.2         munsell_0.5.0              
## [143] tiff_0.1-11                 glmnet_4.1-8               
## [145] Matrix_1.5-3                hms_1.1.3                  
## [147] bit64_4.0.5                 KEGGREST_1.40.1            
## [149] highr_0.10                  SummarizedExperiment_1.30.2
## [151] broom_1.0.5                 memoise_2.0.1              
## [153] bslib_0.5.1                 bit_4.0.5
```

## Contents

Developed by Aleksandra Bliznina, Michael Mansfield, Charles Plessy.

Site built with pkgdown 2.0.7.
