# Supplementary material for: Extreme genome scrambling in marine planktonic Oikopleura dioica cryptic species
Source: Genome Res. 2024 Mar;34(3):426–40. doi: 10.1101/gr.278295.123 (PMC11067885; doi:10.1101/gr.278295.123)
Supplement: Supplement 4 [file Supplemental_Data.zip › Supplemental_Data.tar.xz!/Supplemental_Data/articles/ParallelPlots.html]

Whole-chromosome parallel plots • OikScrambling
 


  


 


Toggle navigation


OikScrambling
5.1.0

- Get started
- Data load
- Supplemental material
  - Figure 1A DNA line plots
  - Figure 1B chromosome parallel plots
  - Table 1 scrambling index
  - Figure 5 genome-wide feature patterns
  - Figure 7 DNA line plots
  - Figure 7 breakpoint number barplots
- Articles
  - Scaffolding of the AOM-5 genome
  - Scaffolding of the OdB3 genome
  - Conserved non-coding elements
  - Chromosome-scale feature density plots
  - Modifying coalescent cutoffs
  - Regions interrupting colinearity
  - Distance Measures
  - Genomic features of the breakpoints
  - Genomic features of the breakpoints (heatmaps)
  - Inversions
  - Scaffolding of the KUM-M3 genome
  - Load genomic break data
  - Operon conservation analysis
  - Orthogroup syntenies
  - Alignment line plots, whole genomes
  - Region of interest: PAC3
  - Motif search and analysis
  - Whole-chromosome parallel plots
  - Width of the aligned and mapped regions.
  - Regions of interest
  - Repeat regions
  - Scaling breakpoints
  - Segment movement distances
  - Alignment line plots, 10×10 Mbp windows
  - Transcription factor binding sites
  - Tandem site duplications
  - Translocations
- Changelog
- Reference

# Whole-chromosome parallel plots

#### Charles Plessy

#### 06 November, 2023

Source: `vignettes/ParallelPlots.Rmd`

`ParallelPlots.Rmd`

```
knitr::opts_chunk$set(cache = TRUE, cache.lazy = FALSE)
knitr::opts_knit$set(verbose = TRUE)
```

## Introduction

Pairwise alignments between whole chromosomes are plotted in parallel
in order to reflect synteny and the difference of scrambling between
short and long arms.

Figure 1 panel B was generated in this vignette.

## Load R pacakges and data

### Load pacakges

```
library('OikScrambling') |> suppressPackageStartupMessages()
genomes <- OikScrambling:::loadAllGenomes()
```

```
## Warning in runHook(".onLoad", env, package.lib, package): input string
## 'Génoscope' cannot be translated from 'ANSI_X3.4-1968' to UTF-8, but is valid
## UTF-8

## Warning in runHook(".onLoad", env, package.lib, package): input string
## 'Génoscope' cannot be translated from 'ANSI_X3.4-1968' to UTF-8, but is valid
## UTF-8
```

```
load("BreakPoints.Rdata")
```

### Phylogenetic cladogram

We assume that the *North Pacific* and the *Atlantic*
species are more related to each other than to the *Okinawan*
species.

```
requireNamespace("ade4")
treeLeaf <- function(name, length=NULL) {
  if(!is.null(length)) length <- paste0(':', length)
  paste0(name, length)
}

treeNode <- function(branch1, branch2, length = NULL) {
  if(!is.null(length)) length <- paste0(':', length)
  paste0('(', branch1, ',', branch2, ')', length)
}

addRoot <- function(branch)  paste0(branch, ";")

tree <-
  addRoot(
    treeNode(
      treeNode( length = 2,
        treeLeaf("Okinawa", 1),
        treeLeaf("Kume", 1)
      ),
      treeNode( length = 1,
        treeNode( length = 1,
          treeLeaf("Osaka", 1),
          treeLeaf("Aomori", 1)
        ),
        treeNode( length =1,
          treeLeaf("Norway", 1),
          treeLeaf("Barcelona", 1)
        )
      )
    )
  )

plot(ade4::newick2phylog(tree))
```

## Plot chr2 in six species

We chose chromosome 2 because it has *Hox4*.

### Parallel plot

```
KumChr2 <- scaffoldByFlipAndMerge(gbs$Oki_Kum |> swap(), scafs$Kum_Oki, drop = TRUE) |> swap() |> coalesce_contigs()
Aom_Osa <- scaffoldByFlipAndMerge(gbs$Osa_Aom |> swap(), scafs$Aom_Osa, drop = TRUE) |> swap() |> coalesce_contigs()
Aom_Oki <- scaffoldByFlipAndMerge(gbs$Oki_Aom |> swap(), scafs$Aom_Osa, drop = TRUE) |> swap() |> coalesce_contigs()
Bar_Nor <- gbs$Bar_Nor |> swap() |>
  splitSeqLevel("scaffold_3", 700924) |>
  splitSeqLevel("scaffold_8", 1110680) |>
  scaffoldByFlipAndMerge(scafs$Nor_Bar) |>
  coalesce_contigs() |> swap(sort = TRUE)

dsList6 <- list(
    Kume = KumChr2 |> swap() |> plyranges::filter(seqnames == 'Chr2', seqnames(query) == 'chr2') |> gr2dna_seg(),
    Okinawa = c(
      KumChr2           |> plyranges::filter(seqnames == 'chr2', seqnames(query) == 'Chr2') |> granges(),
      Aom_Oki           |> plyranges::filter(seqnames == 'Chr2', seqnames(query) == 'chr2') |> granges()
      ) |> reduce() |> gr2dna_seg(),
    Aomori = c(
      Aom_Oki |> swap() |> plyranges::filter(seqnames == 'Chr2', seqnames(query) == 'chr2') |> granges(),
      Aom_Osa |> swap() |> plyranges::filter(seqnames == 'Chr2', seqnames(query) == 'Chr2') |> granges()
      ) |> reduce() |> gr2dna_seg(),
    Osaka = c(
      Aom_Osa           |> plyranges::filter(seqnames == 'Chr2', seqnames(query) == 'Chr2') |> granges(),
      coa$Osa_Bar       |> plyranges::filter(seqnames == 'Chr2', seqnames(query) == 'Chr2') |> granges()
      ) |> reduce() |> gr2dna_seg(),
    Barcelona = c(
      coa$Bar_Osa       |> plyranges::filter(seqnames == 'Chr2', seqnames(query) == 'Chr2') |> granges(),
      Bar_Nor           |> plyranges::filter(seqnames == 'Chr2', seqnames(query) == 'Chr2') |> granges()
      ) |> reduce() |> gr2dna_seg(),
    Bergen = Bar_Nor |> swap() |> plyranges::filter(seqnames == 'Chr2', seqnames(query) == 'Chr2') |> gr2dna_seg()
)

compList6 <- list(
    Oki_Kum = KumChr2 |> swap() |> plyranges::filter(seqnames == 'Chr2', seqnames(query) == 'chr2') |> gb2comp(),
    Aom_Oki = Aom_Oki           |> plyranges::filter(seqnames == 'chr2', seqnames(query) == 'Chr2') |> gb2comp(),
    Osa_Aom = Aom_Osa |> swap() |> plyranges::filter(seqnames == 'Chr2', seqnames(query) == 'Chr2') |> gb2comp(),
    Osa_Bar = coa$Osa_Bar       |> plyranges::filter(seqnames == 'Chr2', seqnames(query) == 'Chr2') |> gb2comp(),
    Bar_Nor = Bar_Nor           |> plyranges::filter(seqnames == 'Chr2', seqnames(query) == 'Chr2') |> gb2comp()
)

dsList6$Kume$col <- dsList6$Kume$fill <- dsList6$Okinawa$col <- dsList6$Okinawa$fill <-
  dsList6$Aomori$col <- dsList6$Aomori$fill <-
  dsList6$Osaka$col <- dsList6$Osaka$fill <- dsList6$Barcelona$col <- dsList6$Barcelona$fill <-
  dsList6$Bergen$col <- dsList6$Bergen$fill <- "#A6D854"

compList6$Oki_Kum$color <- compList6$Aom_Oki$color <- compList6$Osa_Aom$color <-
  compList6$Osa_Bar$color <- compList6$Bar_Nor$color <- "#D4EACC"

compList6$Oki_Kum$col <- ifelse(compList6$Oki_Kum$col == "darksalmon", "#D4EACC", "#00A08E")
compList6$Aom_Oki$col <- ifelse(compList6$Aom_Oki$col == "darksalmon", "#D4EACC", "#00A08E")
compList6$Osa_Aom$col <- ifelse(compList6$Osa_Aom$col == "darksalmon", "#D4EACC", "#00A08E")
compList6$Osa_Bar$col <- ifelse(compList6$Osa_Bar$col == "darksalmon", "#D4EACC", "#00A08E")
compList6$Bar_Nor$col <- ifelse(compList6$Bar_Nor$col == "darksalmon", "#D4EACC", "#00A08E")

genoPlotR::plot_gene_map(dsList6, compList6, dna_seg_scale = FALSE,
                         tree = ade4::newick2phylog("((Kume:1,Okinawa:1):2,((Aomori:1,Osaka:1):1,(Barcelona:1,Bergen:1):1):1);"),
                         main = "Chromosome 2 in six Oikopleura genome assemblies")
```

## Plot chr2 in four species

We chose chromosome 2 because it has *Hox4*.

### Parallel plot

```
dsList4 <- list(
    KUM = KumChr2 |> swap() |> plyranges::filter(seqnames == 'Chr2', seqnames(query) == 'chr2') |> gr2dna_seg(),
    OKI = KumChr2           |> plyranges::filter(seqnames == 'chr2', seqnames(query) == 'Chr2') |> gr2dna_seg(),
    OSA = coa$Osa_Bar |> plyranges::filter(seqnames == 'Chr2', seqnames(query) == 'Chr2') |> gr2dna_seg(),
    BAR = coa$Bar_Osa |> plyranges::filter(seqnames == 'Chr2', seqnames(query) == 'Chr2') |> gr2dna_seg()
)

compList4 <- list(
    Oki_Kum = KumChr2 |> swap() |> plyranges::filter(seqnames == 'Chr2', seqnames(query) == 'chr2') |> gb2comp(),
    Oki_Osa = coa$Oki_Osa |> plyranges::filter(seqnames == 'chr2', seqnames(query) == 'Chr2') |> gb2comp(),
    Osa_Bar = coa$Osa_Bar |> plyranges::filter(seqnames == 'Chr2', seqnames(query) == 'Chr2') |> gb2comp()
  )

dsList4$KUM$col <- dsList4$KUM$fill <- dsList4$OKI$col <- dsList4$OKI$fill <-
  dsList4$OSA$col <- dsList4$OSA$fill <- dsList4$BAR$col <- dsList4$BAR$fill <-
  "#A6D854"

compList4$Oki_Kum$color <- compList4$Oki_Osa$color <- compList4$Osa_Bar$color <- "#D4EACC"

compList4$Oki_Kum$col <- ifelse(compList4$Oki_Kum$col == "darksalmon", "#D4EACC", "#00A08E")
compList4$Osa_Bar$col <- ifelse(compList4$Osa_Bar$col == "darksalmon", "#D4EACC", "#00A08E")
compList4$Oki_Osa$col <- ifelse(compList4$Oki_Osa$col == "darksalmon", "#D4EACC", "#00A08E")


genoPlotR::plot_gene_map(dsList4, compList4, dna_seg_scale = FALSE,
                         tree = ade4::newick2phylog(addRoot(treeNode(treeNode("OKI", "KUM"), treeNode("OSA", "BAR")))),
                         main = "Chromosome 2 in four Oikopleura genome assemblies")
```

## Plot chr2 in five species

We chose chromosome 2 because it has *Hox4*.

### Parallel plot

```
Aom_Osa <- scaffoldByFlipAndMerge(gbs$Osa_Aom |> swap(), scafs$Aom_Osa, drop = TRUE) |> swap() |> coalesce_contigs()
Aom_Oki <- scaffoldByFlipAndMerge(gbs$Oki_Aom |> swap(), scafs$Aom_Osa, drop = TRUE) |> swap() |> coalesce_contigs()

dsList5 <- list(
    KUM = KumChr2 |> swap() |> plyranges::filter(seqnames == 'Chr2', seqnames(query) == 'chr2') |> gr2dna_seg(),
    OKI = KumChr2           |> plyranges::filter(seqnames == 'chr2', seqnames(query) == 'Chr2') |> gr2dna_seg(),
    AOM = Aom_Osa |> swap() |> plyranges::filter(seqnames == 'Chr2', seqnames(query) == 'Chr2') |> gr2dna_seg(),
    OSA = coa$Osa_Bar |> plyranges::filter(seqnames == 'Chr2', seqnames(query) == 'Chr2') |> gr2dna_seg(),
    BAR = coa$Bar_Osa |> plyranges::filter(seqnames == 'Chr2', seqnames(query) == 'Chr2') |> gr2dna_seg()
)

compList5 <- list(
    Oki_Kum = KumChr2 |> swap() |> plyranges::filter(seqnames == 'Chr2', seqnames(query) == 'chr2') |> gb2comp(),
    Aom_Oki = Aom_Oki |> swap() |> plyranges::filter(seqnames == 'Chr2', seqnames(query) == 'chr2') |> gb2comp(),
    Osa_Aom = Aom_Osa |> plyranges::filter(seqnames == 'Chr2', seqnames(query) == 'Chr2') |> gb2comp(),
    Osa_Bar = coa$Osa_Bar |> plyranges::filter(seqnames == 'Chr2', seqnames(query) == 'Chr2') |> gb2comp()
  )

dsList5$KUM$col <- dsList5$KUM$fill <- dsList5$OKI$col <- dsList5$OKI$fill <-
  dsList5$AOM$col <- dsList5$AOM$fill <-
  dsList5$OSA$col <- dsList5$OSA$fill <- dsList5$BAR$col <- dsList5$BAR$fill <-
  "#A6D854"

compList5$Oki_Kum$color <- compList5$Aom_Oki$color <- compList5$Osa_Aom$color <-  compList5$Osa_Bar$color <- "#D4EACC"

compList5$Oki_Kum$col <- ifelse(compList5$Oki_Kum$col == "darksalmon", "#D4EACC", "#00A08E")
compList5$Aom_Oki$col <- ifelse(compList5$Aom_Oki$col == "darksalmon", "#D4EACC", "#00A08E")
compList5$Osa_Aom$col <- ifelse(compList5$Osa_Aom$col == "darksalmon", "#D4EACC", "#00A08E")
compList5$Osa_Bar$col <- ifelse(compList5$Osa_Bar$col == "darksalmon", "#D4EACC", "#00A08E")


genoPlotR::plot_gene_map(dsList5, compList5, dna_seg_scale = FALSE,
                         tree = ade4::newick2phylog(addRoot(treeNode(treeNode("OKI", "KUM"), treeNode(treeNode("OSA", "AOM"), "BAR")))),
                         main = "Chromosome 2 in five Oikopleura genome assemblies")
```

### Strand randomisation indexes

These numbers were added to the plot in Figure 1B.

```
chr2Long <-  \(gb) plyranges::filter(gb, seqnames %in% c("chr2", "Chr2"), seqnames(query) %in% c("chr2", "Chr2"), Arm == "long",  query$Arm == "long")
chr2short <- \(gb) plyranges::filter(gb, seqnames %in% c("chr2", "Chr2"), seqnames(query) %in% c("chr2", "Chr2"), Arm == "short", query$Arm == "short")
chr2both <- \(gb) plyranges::filter(gb, seqnames %in% c("chr2", "Chr2"), seqnames(query) %in% c("chr2", "Chr2"))

KumChr2 <- GenomicBreaks::flagLongShort(KumChr2, longShort$OKI2018.I69)
KumChr2$query$Arm <- Rle("long")
KumChr2$query$Arm[
  end(KumChr2$query) <= (cumsum(seqlengths(coa$Oki_Kum$query)[scafs$Kum_Oki$Chr2$contig]) -1) ["contig_16_1"] &
    seqnames(KumChr2) == "chr2" &
    seqnames(KumChr2$query) == "Chr2"
  ] <- "short"

Aom_Osa <- GenomicBreaks::flagLongShort(Aom_Osa, longShort$OSKA2016v1.9)
Aom_Osa$query <- GenomicBreaks::flagLongShort(
  Aom_Osa$query,
  GRanges(c("Chr2:1-5523357", "Chr2:5523357-12906126"), Type = c("short", "long")))

Aom_Oki <- GenomicBreaks::flagLongShort(Aom_Oki, longShort$OKI2018.I69)
Aom_Oki$query <- GenomicBreaks::flagLongShort(
  Aom_Oki$query,
  GRanges(c("Chr2:1-5523357", "Chr2:5523357-12906126"), Type = c("short", "long")))


Bar_Nor <- GenomicBreaks::flagLongShort(Bar_Nor, longShort$Bar2.p4)
Bar_Nor$query <- GenomicBreaks::flagLongShort(
  Bar_Nor$query,
  GRanges(c("Chr2:1-4525974", "Chr2:4525975-12906790"), Type = c("short", "long")))

SRI_Both_Strands <- \(gb) mean(c(strand_randomisation_index(gb)
                                ,strand_randomisation_index(swap(gb))))

(SRIs <- c(
  Kum_Chr2_long  = KumChr2 |> chr2Long()  |> SRI_Both_Strands(),
  Kum_Chr2_short = KumChr2 |> chr2short() |> SRI_Both_Strands(),
  Kum_Chr2_both  = KumChr2 |> chr2both()  |> SRI_Both_Strands(),
  
  Oki_Aom_long   = Aom_Oki |> chr2Long()  |> SRI_Both_Strands(),
  Oki_Aom_short  = Aom_Oki |> chr2short() |> SRI_Both_Strands(),
  Oki_Aom_both   = Aom_Oki |> chr2both()  |> SRI_Both_Strands(),
  
  Osa_Aom_long   = Aom_Osa |> chr2Long()  |> SRI_Both_Strands(),
  Osa_Aom_short  = Aom_Osa |> chr2short() |> SRI_Both_Strands(),
  Osa_Aom_both   = Aom_Osa |> chr2both()  |> SRI_Both_Strands(),

  Oki_Osa_long   = coa$Oki_Osa |> chr2Long() |> SRI_Both_Strands(),
  Osa_Oki_long   = coa$Osa_Oki |> chr2Long() |> SRI_Both_Strands(),

  Oki_Osa_short  = coa$Oki_Osa |> chr2short() |> SRI_Both_Strands(),
  Osa_Oki_short  = coa$Osa_Oki |> chr2short() |> SRI_Both_Strands(),

  Oki_Osa_both   = coa$Oki_Osa |> chr2both() |> SRI_Both_Strands(),
  Osa_Oki_both   = coa$Osa_Oki |> chr2both() |> SRI_Both_Strands(),

  Osa_Bar_long   = coa$Osa_Bar |> chr2Long() |> SRI_Both_Strands(),
  Bar_Osa_long   = coa$Bar_Osa |> chr2Long() |> SRI_Both_Strands(),

  Osa_Bar_short  = coa$Osa_Bar |> chr2short() |> SRI_Both_Strands(),
  Bar_Osa_short  = coa$Bar_Osa |> chr2short() |> SRI_Both_Strands(),

  Osa_Bar_both   = coa$Osa_Bar |> chr2both() |> SRI_Both_Strands(),
  Bar_Osa_both   = coa$Bar_Osa |> chr2both() |> SRI_Both_Strands(),
  
  Bar_Nor_short  = Bar_Nor     |> chr2short() |> SRI_Both_Strands(),
  Bar_Nor_long   = Bar_Nor     |> chr2Long()  |> SRI_Both_Strands()
))
```

```
##  Kum_Chr2_long Kum_Chr2_short  Kum_Chr2_both   Oki_Aom_long  Oki_Aom_short 
##    0.832774687    0.762407551    0.807347070    0.007713088    0.014947894 
##   Oki_Aom_both   Osa_Aom_long  Osa_Aom_short   Osa_Aom_both   Oki_Osa_long 
##    0.020490460    0.762052760    0.169998327    0.509978155    0.013253234 
##   Osa_Oki_long  Oki_Osa_short  Osa_Oki_short   Oki_Osa_both   Osa_Oki_both 
##    0.017958605    0.020211761    0.019965884    0.016624847    0.014163168 
##   Osa_Bar_long   Bar_Osa_long  Osa_Bar_short  Bar_Osa_short   Osa_Bar_both 
##    0.158432788    0.159058114    0.018673220    0.016156226    0.108829940 
##   Bar_Osa_both  Bar_Nor_short   Bar_Nor_long 
##    0.105527771    0.980679790    0.967169963
```

```
roundMean <- \(x) round(mean(SRIs[x]), 2)
data.frame(
  short = c(
    Kum_Oki = roundMean(c("Kum_Chr2_short")),
    Oki_Aom = roundMean(c("Oki_Aom_short")),
    Aom_Osa = roundMean(c("Osa_Aom_short")),
    Osa_Bar = roundMean(c("Osa_Bar_short", "Bar_Osa_short")),
    Bar_Nor = roundMean(c("Bar_Nor_short"))
  ),
  long = c(
    Kum_Oki = roundMean(c("Kum_Chr2_long")),
    Oki_Aom = roundMean(c("Oki_Aom_long")),
    Aom_Osa = roundMean(c("Osa_Aom_long")),
    Osa_Bar = roundMean(c("Osa_Bar_long",  "Bar_Osa_long")),
    Bar_Nor = roundMean(c("Bar_Nor_long"))
  )
)
```

```
##         short long
## Kum_Oki  0.76 0.83
## Oki_Aom  0.01 0.01
## Aom_Osa  0.17 0.76
## Osa_Bar  0.02 0.16
## Bar_Nor  0.98 0.97
```

## Plot pairs of chromosome 1s.

```
plotApairOfChrs(coa$Oki_Bar, "chr1", main = "Oki vs Bar")
```

```
plotApairOfChrs(coa$Oki_Osa, "chr1", main = "Oki vs Osa")
```

```
plotApairOfChrs(coa$Osa_Oki, "Chr1", main = "Osa vs Oki")
```

```
plotApairOfChrs(coa$Osa_Bar, "Chr1", main = "Osa vs Bar")
```

```
plotApairOfChrs(coa2$Osa_Bar, "Chr1", main = "Osa vs Bar (double-collapsed")
```

## Scrambling of a 1-megabase region.

```
x <- subsetByOverlaps(coa2$Oki_Osa, granges(coa2$Oki_Bar)) |> subset(width > 1e5)
y <- subsetByOverlaps(coa2$Oki_Bar, granges(coa2$Oki_Osa)) |> subset(width > 1e5)
findOverlapPairs(x,y)
```

```
## Pairs object with 4 pairs and 0 metadata columns:
##                          first                   second
##                      <GBreaks>                <GBreaks>
##   [1] chr2:10664980-10829335:+ chr2:10664980-10828981:+
##   [2] chr2:13895789-14003399:- chr2:13895789-14003408:-
##   [3]  PAR:15193293-15358116:-  PAR:15193294-15358116:-
##   [4]  XSR:10090052-10210973:-  XSR:10090052-10210972:-
```

```
subsetByOverlaps(coa2$Oki_Osa, gaps(coa2$Oki_Bar)) |> subset(width > 1e5)
```

```
## GBreaks object with 16 ranges and 6 metadata columns:
##        seqnames            ranges strand |                  query      Arm
##           <Rle>         <IRanges>  <Rle> |              <GRanges> <factor>
##    [1]     chr2   5825747-5966560      + |   Chr2:5465216-5578318     long
##    [2]     chr2 10361302-10481638      + | Chr2:11648925-11718071     long
##    [3]     chr2 10664980-10829335      + |   Chr2:5679693-5828385     long
##    [4]     chr2 13895789-14003399      - | Chr2:11294781-11373503     long
##    [5]     chr2 14726918-14844516      + |   Chr2:8440297-8537610     long
##    ...      ...               ...    ... .                    ...      ...
##   [12]      XSR   7236669-7346658      + |    XSR:4093395-4195157      XSR
##   [13]      XSR   7543498-7653200      + |    XSR:3606737-3707501      XSR
##   [14]      XSR   8709632-8820503      + |    XSR:7297396-7413239      XSR
##   [15]      XSR   8929901-9075791      - |    XSR:7506741-7641724      XSR
##   [16]      XSR 10090052-10210973      - |  XSR:10484001-10612402      XSR
##                                  rep   repOvlp            transcripts
##                      <CharacterList> <integer>                  <Rle>
##    [1]        tandem,unknown,rnd,...      5240 g5554.t1;g5556.t1;g5..
##    [2]        tandem,unknown,rnd,...     11135 g6889.t1;g6890.t1;g6..
##    [3] rnd,unknown,LowComplexity,...      5389 g7009.t1;g7010.t1;g7..
##    [4]        rnd,unknown,tandem,...      6725 g7918.t1;g7922.t1;g7..
##    [5]        tandem,rnd,unknown,...      9346 g8164.t1;g8165.t1;g8..
##    ...                           ...       ...                    ...
##   [12]        tandem,rnd,unknown,...      2161 g15405.t1;g15406.t1;..
##   [13]        tandem,rnd,unknown,...      5241 g15508.t1;g15509.t1;..
##   [14]      rnd,tandem,LowComplexity      1409 g15864.t1;g15865.t1;..
##   [15] unknown,rnd,LowComplexity,...      3812 g15934.t1;g15935.t1;..
##   [16]  LowComplexity,rnd,tandem,...      1737 g16292.t1;g16293.t1;..
##               flag
##        <character>
##    [1]        <NA>
##    [2]        <NA>
##    [3]        <NA>
##    [4]        <NA>
##    [5]        <NA>
##    ...         ...
##   [12]         Tra
##   [13]        <NA>
##   [14]        <NA>
##   [15]        <NA>
##   [16]        <NA>
##   -------
##   seqinfo: 19 sequences from OKI2018.I69 genome
```

```
ROI <- GRanges("chr2:10000000-11000000")

a <- coa$Oki_Osa |> subsetByOverlaps(ROI) |> plyranges::filter(seqnames == 'chr2', seqnames(query) == 'Chr2')
b <- coa$Oki_Bar |> subsetByOverlaps(ROI) |> plyranges::filter(seqnames == 'chr2', seqnames(query) == 'Chr2')
c <- coa$Bar_Osa |> subsetByOverlaps(range(b$query))                    |> plyranges::filter(seqnames == 'Chr2', seqnames(query) == 'Chr2')

dsList <- list(
  OSA = swap(a) |> gr2dna_seg(),
  OKI = a |> gr2dna_seg(),
  BAR = swap(b) |> gr2dna_seg(),
  Osa = swap(c) |> gr2dna_seg()
)

compList <- list(
  a |> swap() |> gb2comp(),
  b |> gb2comp(),
  c |> gb2comp()
)

genoPlotR::plot_gene_map(dsList, compList, dna_seg_scale=TRUE)
```

## *Ciona* species

```
plotApairOfChrs(coa2$Ply_Ros, "BNJZ01000001.1", main = "Ciona intestinalis Plymouth vs Roscoff")
```

```
plotApairOfChrs(coa2$Ply_Rob, "BNJZ01000001.1", main = "Ciona intestinalis Plymouth vs Ciona robusta")
```

Unfortunately, *C. savignyi*’s genome assembly is too
fragmented. Here is a plot with the largest fragment.

```
plotApairOfChrs(coa2$Ply_Sav, "BNJZ01000001.1", main = "Ciona intestinalis Plymouth vs Ciona savigny")
```

## Session information

```
sessionInfo()
```

```
## R version 4.3.1 (2023-06-16)
## Platform: x86_64-pc-linux-gnu (64-bit)
## Running under: Debian GNU/Linux 12 (bookworm)
## 
## Matrix products: default
## BLAS:   /usr/lib/x86_64-linux-gnu/blas/libblas.so.3.11.0 
## LAPACK: /usr/lib/x86_64-linux-gnu/lapack/liblapack.so.3.11.0
## 
## locale:
##  [1] LC_CTYPE=C.UTF-8       LC_NUMERIC=C           LC_TIME=C.UTF-8       
##  [4] LC_COLLATE=C.UTF-8     LC_MONETARY=C.UTF-8    LC_MESSAGES=C.UTF-8   
##  [7] LC_PAPER=C.UTF-8       LC_NAME=C              LC_ADDRESS=C          
## [10] LC_TELEPHONE=C         LC_MEASUREMENT=C.UTF-8 LC_IDENTIFICATION=C   
## 
## time zone: Etc/UTC
## tzcode source: system (glibc)
## 
## attached base packages:
## [1] stats4    stats     graphics  grDevices utils     datasets  methods  
## [8] base     
## 
## other attached packages:
##  [1] BSgenome.Oidioi.genoscope.OdB3_1.0.0   
##  [2] BSgenome.Oidioi.OIST.AOM.5.5f_1.0.1    
##  [3] BSgenome.Oidioi.OIST.KUM.M3.7f_1.0.1   
##  [4] BSgenome.Oidioi.OIST.Bar2.p4_1.0.1     
##  [5] BSgenome.Oidioi.OIST.OSKA2016v1.9_1.0.0
##  [6] BSgenome.Oidioi.OIST.OKI2018.I69_1.0.1 
##  [7] OikScrambling_5.0.0                    
##  [8] ggplot2_3.4.3                          
##  [9] GenomicBreaks_0.14.2                   
## [10] BSgenome_1.68.0                        
## [11] rtracklayer_1.60.0                     
## [12] Biostrings_2.68.1                      
## [13] XVector_0.40.0                         
## [14] GenomicRanges_1.52.0                   
## [15] GenomeInfoDb_1.36.1                    
## [16] IRanges_2.34.1                         
## [17] S4Vectors_0.38.1                       
## [18] BiocGenerics_0.46.0                    
## 
## loaded via a namespace (and not attached):
##   [1] splines_4.3.1               BiocIO_1.10.0              
##   [3] bitops_1.0-7                tibble_3.2.1               
##   [5] R.oo_1.25.0                 XML_3.99-0.14              
##   [7] rpart_4.1.19                lifecycle_1.0.3            
##   [9] rprojroot_2.0.3             lattice_0.20-45            
##  [11] MASS_7.3-58.2               backports_1.4.1            
##  [13] magrittr_2.0.3              Hmisc_5.1-0                
##  [15] sass_0.4.7                  rmarkdown_2.23             
##  [17] jquerylib_0.1.4             yaml_2.3.7                 
##  [19] plotrix_3.8-2               DBI_1.1.3                  
##  [21] CNEr_1.36.0                 minqa_1.2.5                
##  [23] RColorBrewer_1.1-3          ade4_1.7-22                
##  [25] abind_1.4-5                 zlibbioc_1.46.0            
##  [27] purrr_1.0.2                 R.utils_2.12.2             
##  [29] RCurl_1.98-1.12             nnet_7.3-18                
##  [31] pracma_2.4.2                GenomeInfoDbData_1.2.10    
##  [33] gdata_2.19.0                annotate_1.78.0            
##  [35] pkgdown_2.0.7               codetools_0.2-19           
##  [37] DelayedArray_0.26.7         tidyselect_1.2.0           
##  [39] shape_1.4.6                 lme4_1.1-34                
##  [41] matrixStats_1.0.0           base64enc_0.1-3            
##  [43] GenomicAlignments_1.36.0    jsonlite_1.8.7             
##  [45] mitml_0.4-5                 Formula_1.2-5              
##  [47] survival_3.5-3              iterators_1.0.14           
##  [49] systemfonts_1.0.5           foreach_1.5.2              
##  [51] tools_4.3.1                 ragg_1.2.5                 
##  [53] Rcpp_1.0.11                 glue_1.6.2                 
##  [55] gridExtra_2.3               pan_1.8                    
##  [57] xfun_0.40                   MatrixGenerics_1.12.2      
##  [59] EBImage_4.42.0              dplyr_1.1.3                
##  [61] withr_2.5.1                 fastmap_1.1.1              
##  [63] boot_1.3-28.1               fansi_1.0.5                
##  [65] digest_0.6.33               R6_2.5.1                   
##  [67] mice_3.16.0                 textshaping_0.3.7          
##  [69] colorspace_2.1-0            GO.db_3.17.0               
##  [71] gtools_3.9.4                poweRlaw_0.70.6            
##  [73] jpeg_0.1-10                 RSQLite_2.3.1              
##  [75] weights_1.0.4               R.methodsS3_1.8.2          
##  [77] utf8_1.2.3                  tidyr_1.3.0                
##  [79] generics_0.1.3              data.table_1.14.8          
##  [81] httr_1.4.7                  htmlwidgets_1.6.2          
##  [83] S4Arrays_1.0.5              pkgconfig_2.0.3            
##  [85] gtable_0.3.4                blob_1.2.4                 
##  [87] htmltools_0.5.6.1           fftwtools_0.9-11           
##  [89] plyranges_1.20.0            scales_1.2.1               
##  [91] Biobase_2.60.0              png_0.1-8                  
##  [93] knitr_1.44                  heatmaps_1.24.0            
##  [95] rstudioapi_0.15.0           tzdb_0.4.0                 
##  [97] reshape2_1.4.4              rjson_0.2.21               
##  [99] checkmate_2.2.0             nlme_3.1-162               
## [101] nloptr_2.0.3                cachem_1.0.8               
## [103] stringr_1.5.0               KernSmooth_2.23-20         
## [105] parallel_4.3.1              genoPlotR_0.8.11           
## [107] foreign_0.8-84              AnnotationDbi_1.62.2       
## [109] restfulr_0.0.15             desc_1.4.2                 
## [111] pillar_1.9.0                grid_4.3.1                 
## [113] vctrs_0.6.3                 jomo_2.7-6                 
## [115] xtable_1.8-4                cluster_2.1.4              
## [117] htmlTable_2.4.1             evaluate_0.22              
## [119] readr_2.1.4                 cli_3.6.1                  
## [121] locfit_1.5-9.8              compiler_4.3.1             
## [123] Rsamtools_2.16.0            rlang_1.1.1                
## [125] crayon_1.5.2                plyr_1.8.8                 
## [127] fs_1.6.3                    stringi_1.7.12             
## [129] BiocParallel_1.34.2         munsell_0.5.0              
## [131] tiff_0.1-11                 glmnet_4.1-7               
## [133] Matrix_1.5-3                hms_1.1.3                  
## [135] bit64_4.0.5                 KEGGREST_1.40.0            
## [137] SummarizedExperiment_1.30.2 broom_1.0.5                
## [139] memoise_2.0.1               bslib_0.5.1                
## [141] bit_4.0.5
```

## Contents

Developed by Aleksandra Bliznina, Michael Mansfield, Charles Plessy.

Site built with pkgdown 2.0.7.
