# Supplementary material for: Extreme genome scrambling in marine planktonic Oikopleura dioica cryptic species
Source: Genome Res. 2024 Mar;34(3):426–40. doi: 10.1101/gr.278295.123 (PMC11067885; doi:10.1101/gr.278295.123)
Supplement: Supplement 4 [file Supplemental_Data.zip › Supplemental_Data.tar.xz!/Supplemental_Data/articles/OrthogroupSyntenies_files/figure-html/synteny_block_histograms_2-1.pdf]

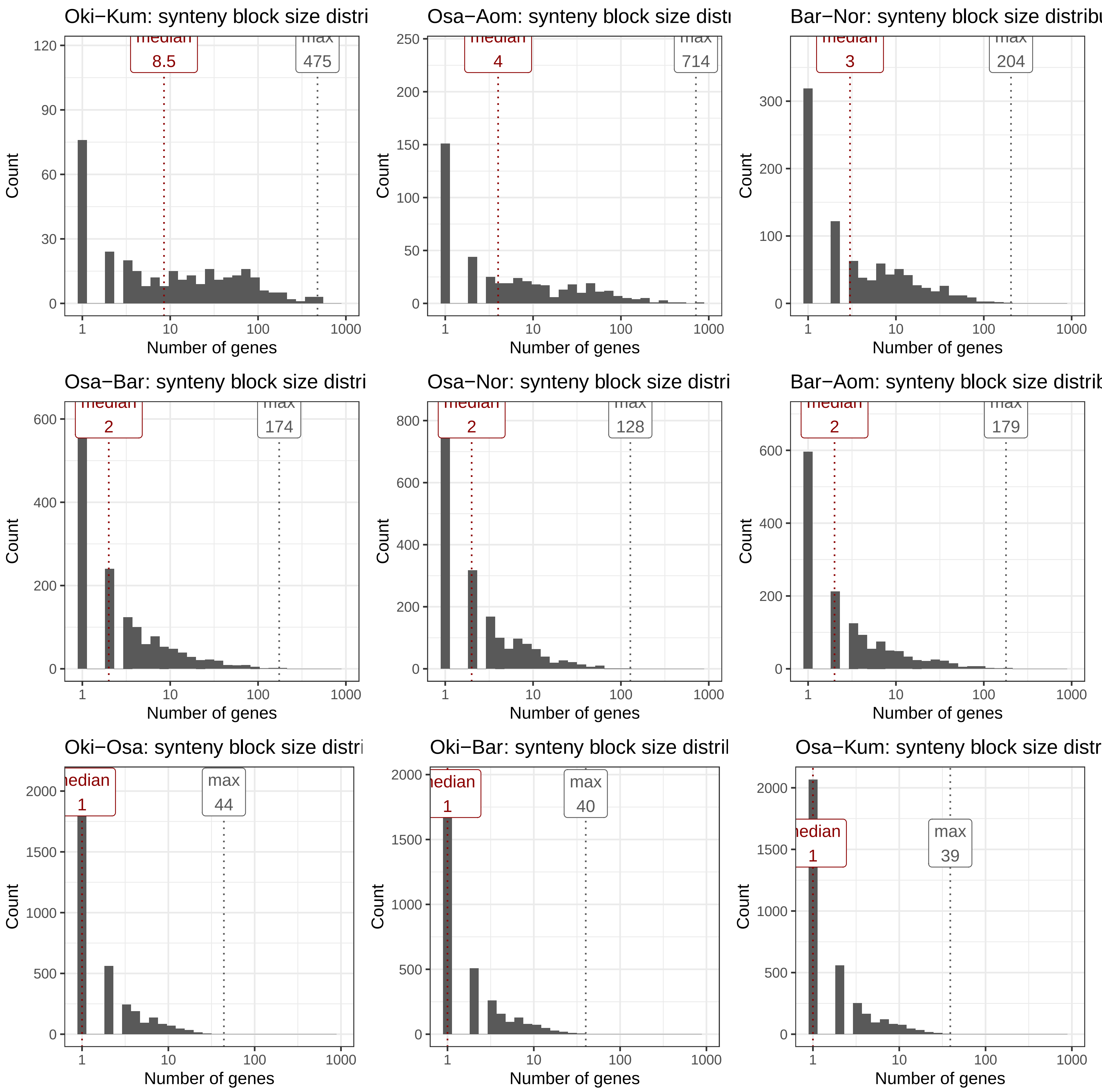

Oki−Kum: synteny block size distri
median
8.5
max
475
120
90
60
30
0
Count
1
10
100
1000
Number of genes
Osa−Aom: synteny block size distı
median
4
max
714
250
200
150
100
50
0
Count
1
10
100
1000
Number of genes
Bar−Nor: synteny block size distribu
median
3
max
204
300
200
100
0
Count
1
10
100
1000
Number of genes
Osa−Bar: synteny block size distri
median
2
max
174
600
400
200
0
Count
1
10
100
1000
Number of genes
Osa−Nor: synteny block size distri
median
2
max
128
800
600
400
200
0
Count
1
10
100
1000
Number of genes
Bar−Aom: synteny block size distrib
median
2
max
179
600
400
200
0
Count
1
10
100
1000
Number of genes
Oki−Osa: synteny block size distri
nedian
1
max
44
2000
1500
1000
500
0
Count
1
10
100
1000
Number of genes
Oki−Bar: synteny block size distril
nedian
1
max
40
2000
1500
1000
500
0
Count
1
10
100
1000
Number of genes
Osa−Kum: synteny block size distr
nedian
1
max
39
2000
1500
1000
500
0
Count
1
10
100
1000
Number of genes
