# Supplementary material for: Extreme genome scrambling in marine planktonic Oikopleura dioica cryptic species
Source: Genome Res. 2024 Mar;34(3):426–40. doi: 10.1101/gr.278295.123 (PMC11067885; doi:10.1101/gr.278295.123)
Supplement: Supplement 4 [file Supplemental_Data.zip › Supplemental_Data.tar.xz!/Supplemental_Data/articles/OrthogroupSyntenies_files/figure-html/synteny_block_histograms_3-2.pdf]

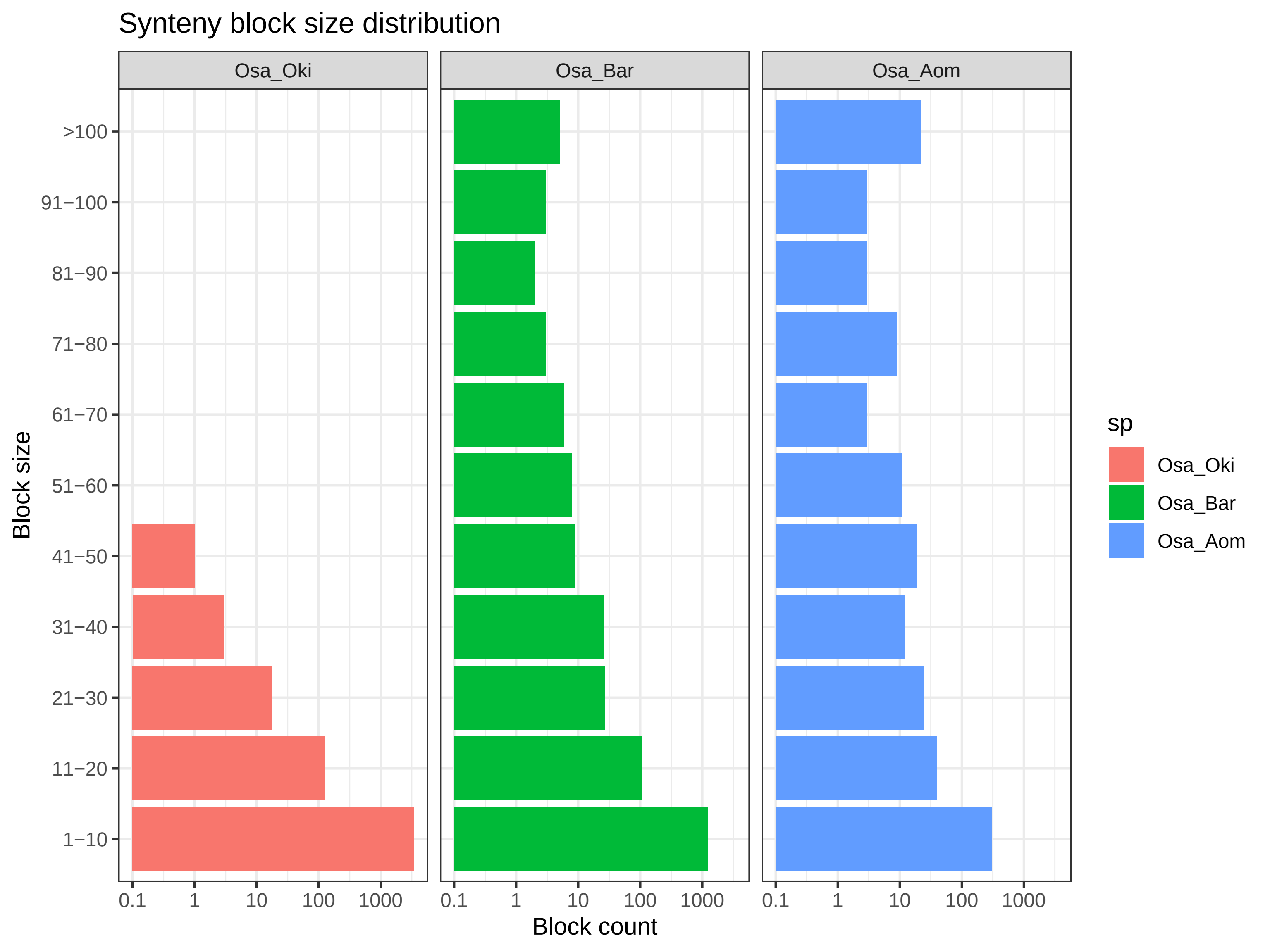

Synteny block size distribution
Osa_Oki
Osa_Bar
Osa_Aom
>100
91−100
81−90
71−80
61−70
51−60
41−50
31−40
21−30
11−20
1−10
Block size
0.1
1
10
100
1000
Block count
sp
Osa_Oki
Osa_Bar
Osa_Aom
