# Supplementary material for: Extreme genome scrambling in marine planktonic Oikopleura dioica cryptic species
Source: Genome Res. 2024 Mar;34(3):426–40. doi: 10.1101/gr.278295.123 (PMC11067885; doi:10.1101/gr.278295.123)
Supplement: Supplement 4 [file Supplemental_Data.zip › Supplemental_Data.tar.xz!/Supplemental_Data/articles/OrthogroupSyntenies_files/figure-html/synteny_block_histograms_3-7.pdf]

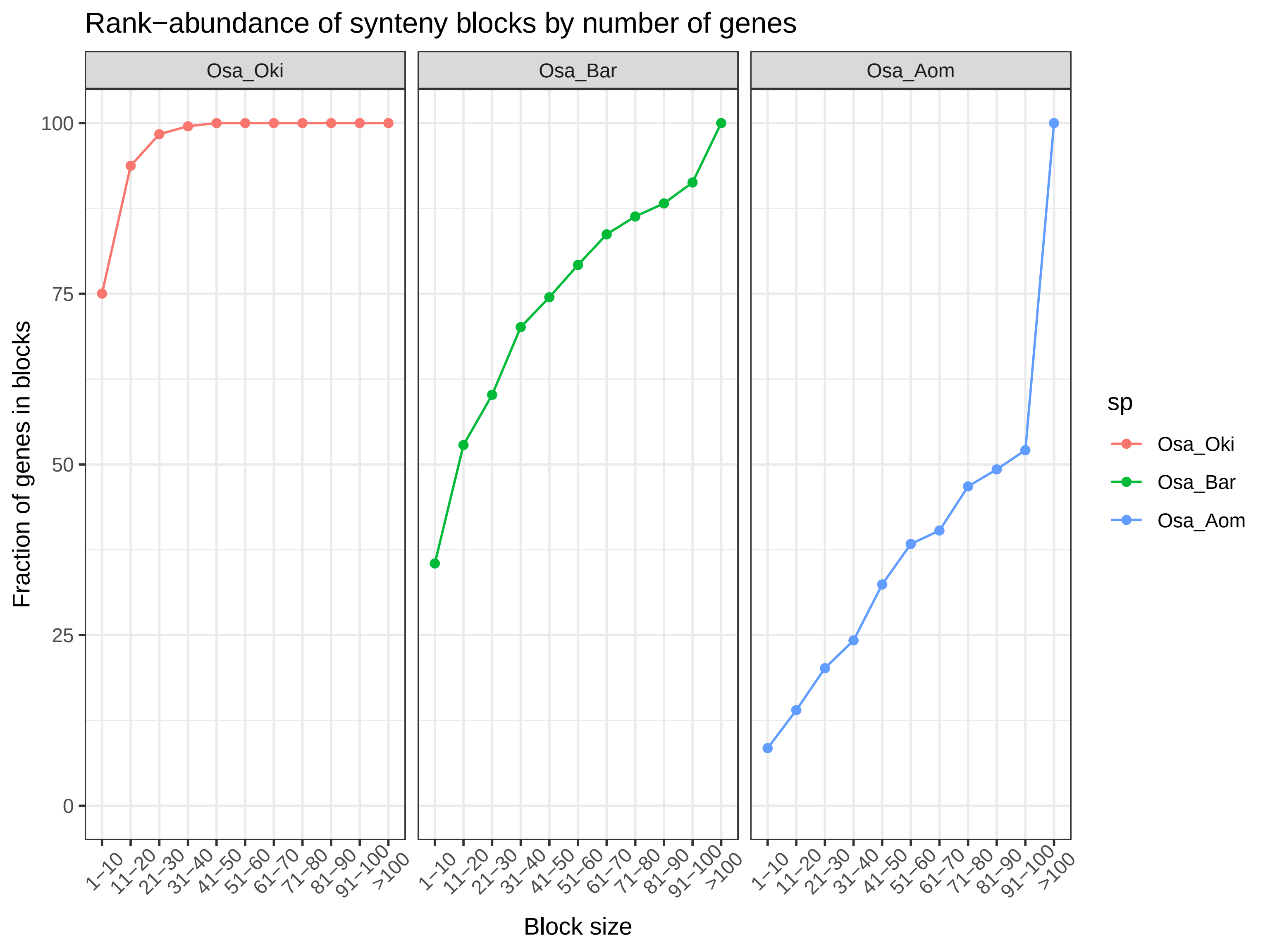

Rank−abundance of synteny blocks by number of genes
Osa_Oki
Osa_Bar
Osa_Aom
Fraction of genes in blocks
100
75
50
25
0
1−10
11−20
21−30
31−40
41−50
51−60
61−70
71−80
81−90
91−100
>100
Block size
sp
Osa_Oki
Osa_Bar
Osa_Aom
