# Supplementary material for: Extreme genome scrambling in marine planktonic Oikopleura dioica cryptic species
Source: Genome Res. 2024 Mar;34(3):426–40. doi: 10.1101/gr.278295.123 (PMC11067885; doi:10.1101/gr.278295.123)
Supplement: Supplement 4 [file Supplemental_Data.zip › Supplemental_Data.tar.xz!/Supplemental_Data/articles/OrthogroupSyntenies_files/figure-html/Hox1-1.pdf]

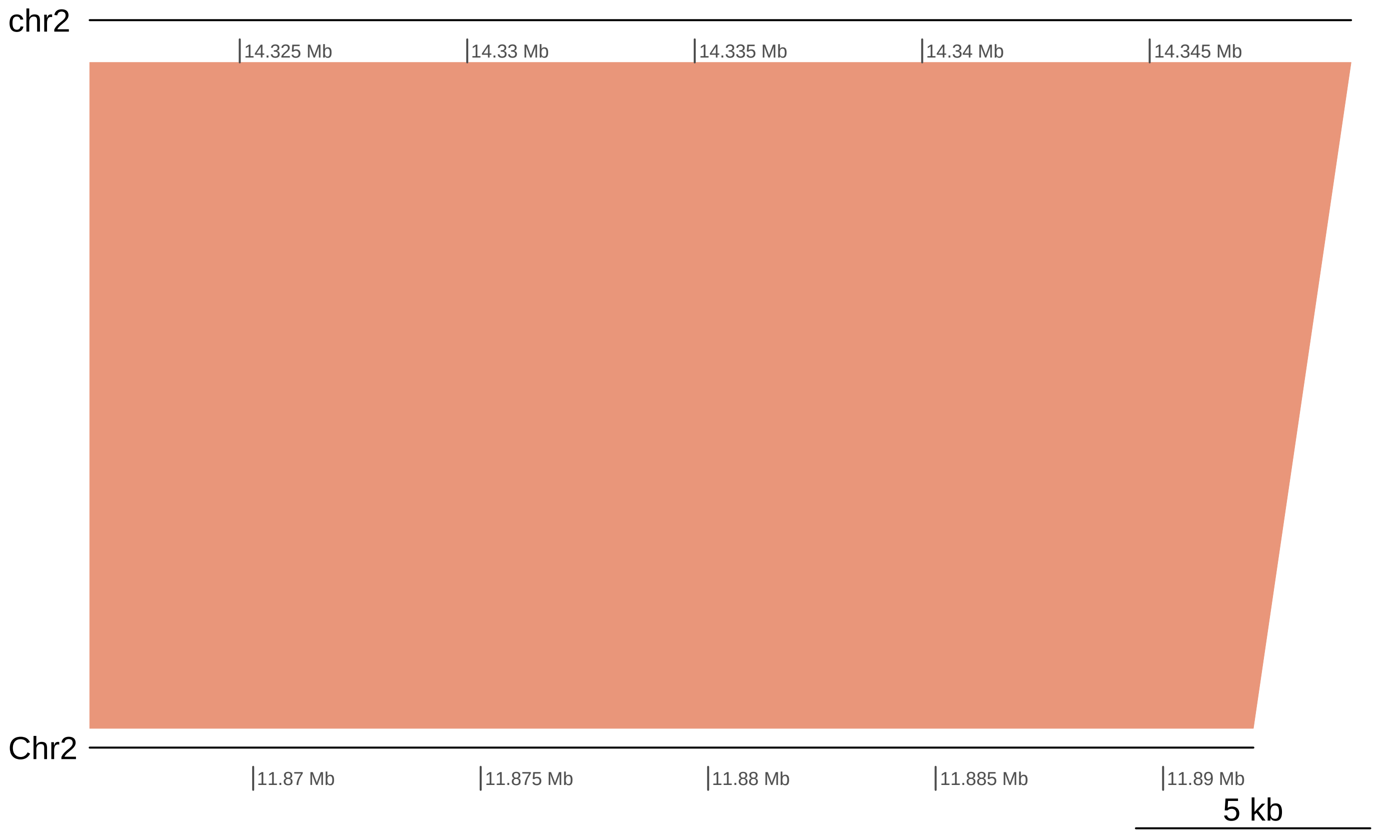
chr2
14.325 Mb
14.33 Mb
14.335 Mb
14.34 Mb
14.345 Mb
Chr2
11.87 Mb
11.875 Mb
11.88 Mb
11.885 Mb
11.89 Mb
5 kb
