# Supplementary material for: Extreme genome scrambling in marine planktonic Oikopleura dioica cryptic species
Source: Genome Res. 2024 Mar;34(3):426–40. doi: 10.1101/gr.278295.123 (PMC11067885; doi:10.1101/gr.278295.123)
Supplement: Supplement 4 [file Supplemental_Data.zip › Supplemental_Data.tar.xz!/Supplemental_Data/articles/OrthogroupSyntenies_files/figure-html/Hox4-4.pdf]

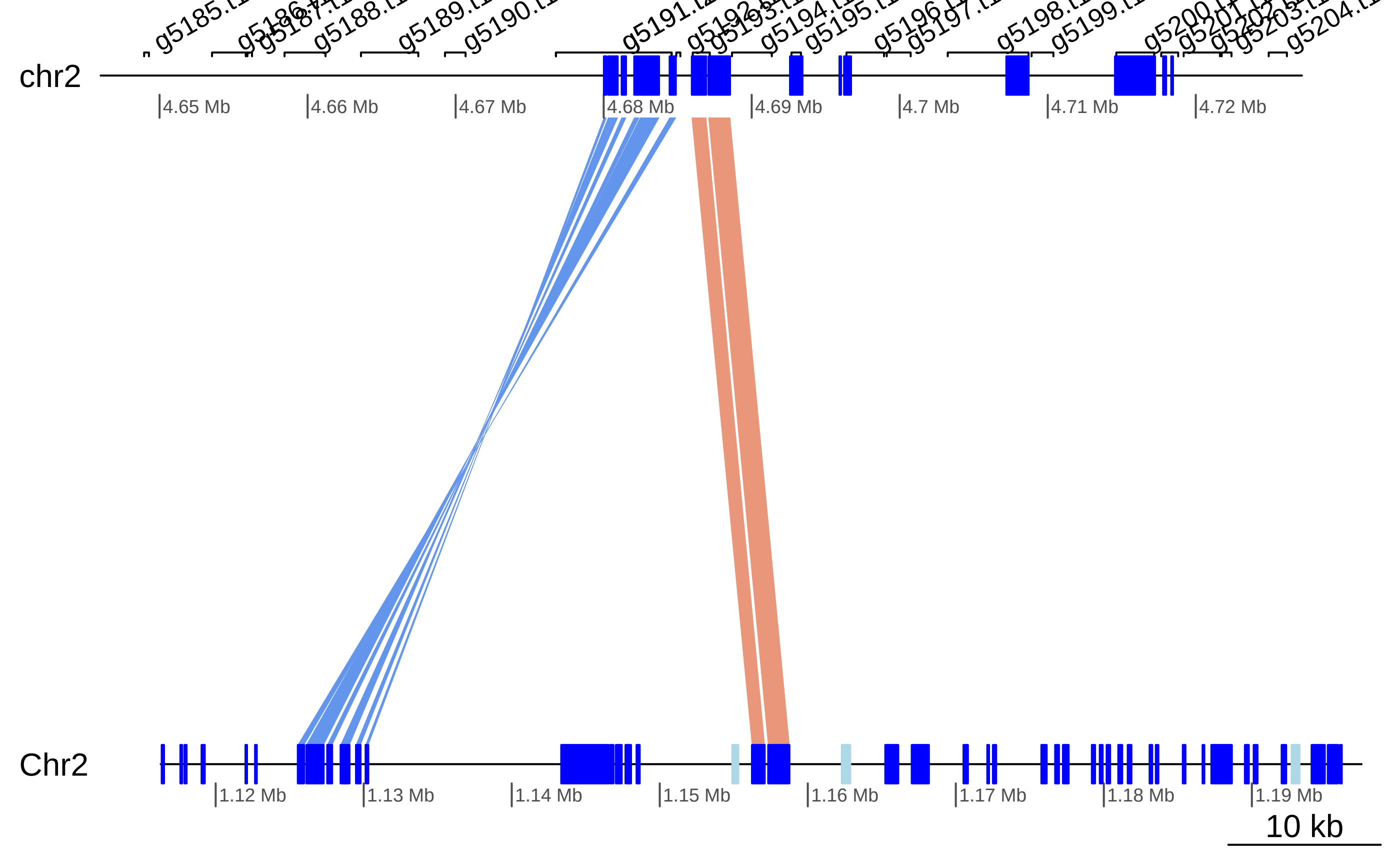
chr2
g5185.t
g5188.t
g5189.t
g5190.t
g5191.t
g5194.t
g5195.t
g5198.t
g5199.t
g5204.t
4.65 Mb
4.66 Mb
4.67 Mb
4.68 Mb
4.69 Mb
4.7 Mb
4.71 Mb
4.72 Mb
Chr2
1.12 Mb
1.13 Mb
1.14 Mb
1.15 Mb
1.16 Mb
1.17 Mb
1.18 Mb
1.19 Mb
10 kb
