# Supplementary material for: Extreme genome scrambling in marine planktonic Oikopleura dioica cryptic species
Source: Genome Res. 2024 Mar;34(3):426–40. doi: 10.1101/gr.278295.123 (PMC11067885; doi:10.1101/gr.278295.123)
Supplement: Supplement 4 [file Supplemental_Data.zip › Supplemental_Data.tar.xz!/Supplemental_Data/articles/OrthogroupSyntenies_files/figure-html/Hox4-1.pdf]

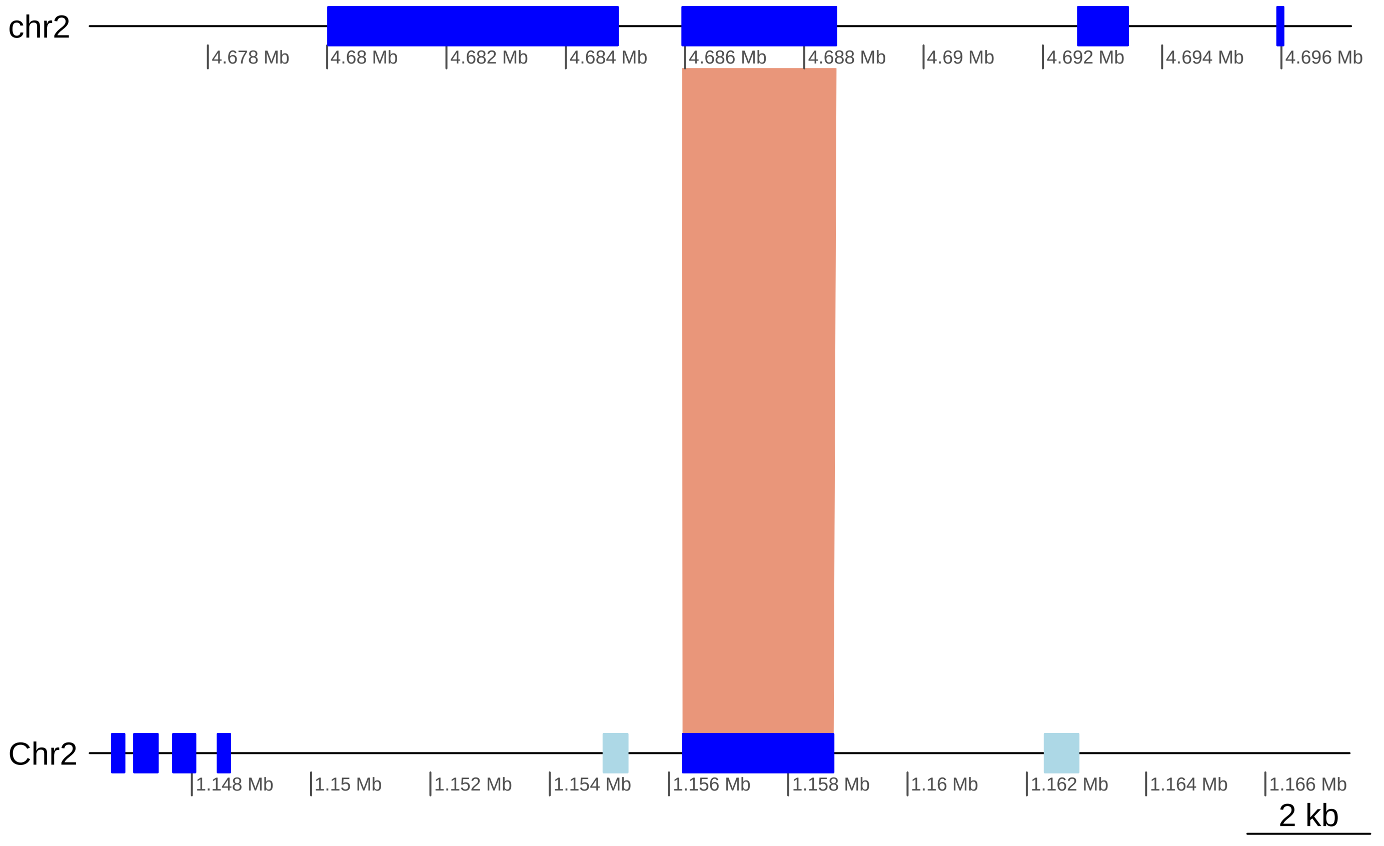

chr2
4.678 Mb
4.68 Mb
4.682 Mb
4.684 Mb
4.686 Mb
4.688 Mb
4.69 Mb
4.692 Mb
4.694 Mb
4.696 Mb
Chr2
1.148 Mb
1.15 Mb
1.152 Mb
1.154 Mb
1.156 Mb
1.158 Mb
1.16 Mb
1.162 Mb
1.164 Mb
1.166 Mb
2 kb
