# Supplementary material for: Extreme genome scrambling in marine planktonic Oikopleura dioica cryptic species
Source: Genome Res. 2024 Mar;34(3):426–40. doi: 10.1101/gr.278295.123 (PMC11067885; doi:10.1101/gr.278295.123)
Supplement: Supplement 4 [file Supplemental_Data.zip › Supplemental_Data.tar.xz!/Supplemental_Data/articles/OrthogroupSyntenies_files/figure-html/synteny_block_histograms-1.pdf]

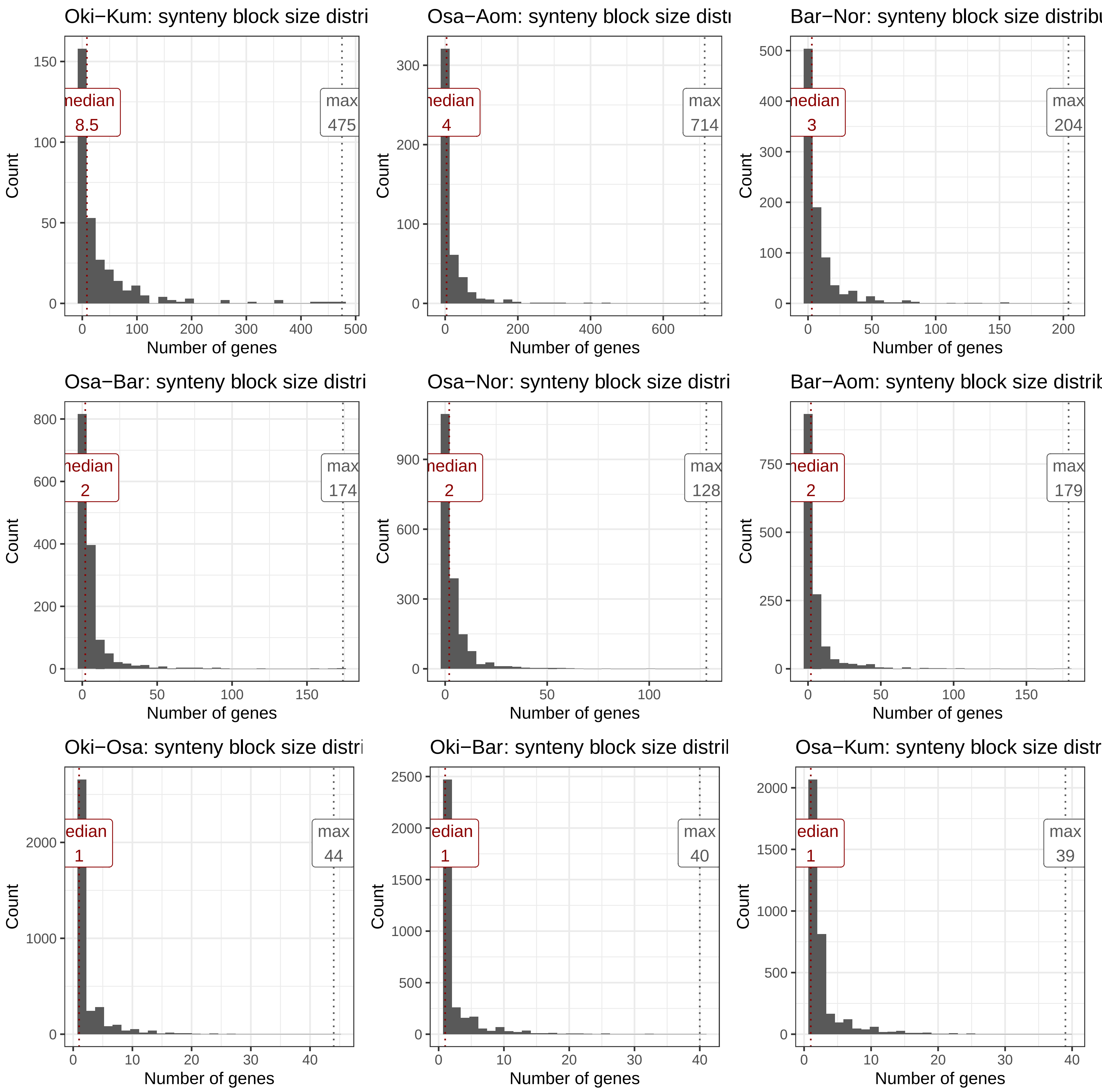
Oki−Kum: synteny block size distri
nedian
8.5
max
475
Count
Number of genes
Osa−Aom: synteny block size distı
nedian
4
max
714
Count
Number of genes
Bar−Nor: synteny block size distribu
nedian
3
max
204
Count
Number of genes
Osa−Bar: synteny block size distri
nedian
2
max
174
Count
Number of genes
Osa−Nor: synteny block size distri
nedian
2
max
128
Count
Number of genes
Bar−Aom: synteny block size distrib
nedian
2
max
179
Count
Number of genes
Oki−Osa: synteny block size distri
edian
1
max
44
Count
Number of genes
Oki−Bar: synteny block size distril
edian
1
max
40
Count
Number of genes
Osa−Kum: synteny block size distr
edian
1
max
39
Count
Number of genes
