# Supplementary material for: Extreme genome scrambling in marine planktonic Oikopleura dioica cryptic species
Source: Genome Res. 2024 Mar;34(3):426–40. doi: 10.1101/gr.278295.123 (PMC11067885; doi:10.1101/gr.278295.123)
Supplement: Supplement 4 [file Supplemental_Data.zip › Supplemental_Data.tar.xz!/Supplemental_Data/articles/OrthogroupSyntenies_files/figure-html/orthopair_plots-1.pdf]

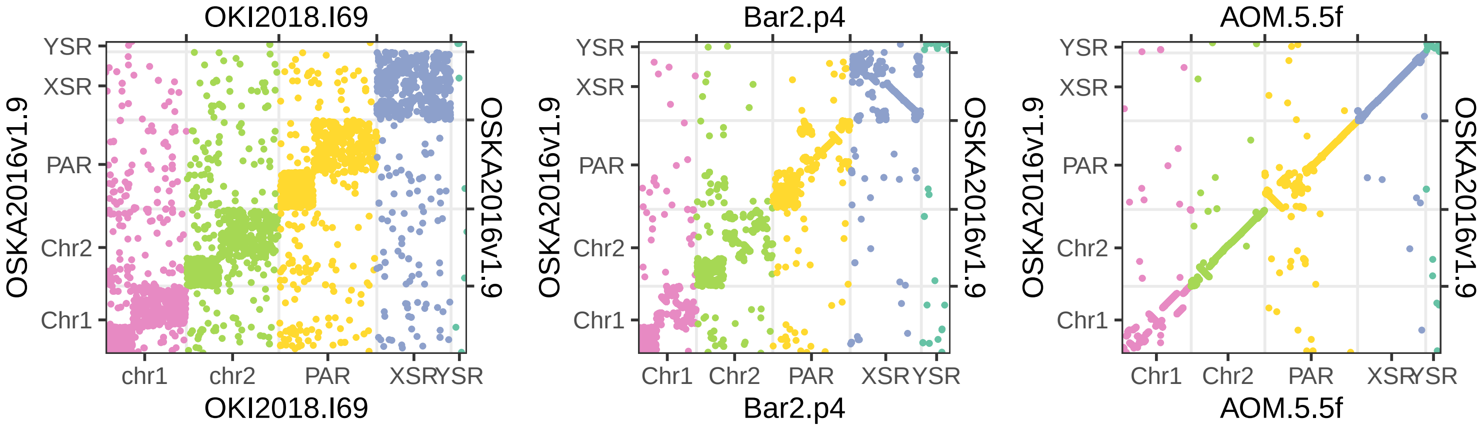
OKI2018.I69
YSR
XSR
PAR
Chr2
Chr1
OSKA2016v1.9
chr1
chr2
PAR
XSR
YSR
OKI2018.I69
OSKA2016v1.9
Bar2.p4
YSR
XSR
PAR
Chr2
Chr1
OSKA2016v1.9
Chr1
Chr2
PAR
XSR
YSR
Bar2.p4
OSKA2016v1.9
AOM.5.5f
YSR
XSR
PAR
Chr2
Chr1
OSKA2016v1.9
Chr1
Chr2
PAR
XSR
YSR
AOM.5.5f
OSKA2016v1.9
