# Supplementary material for: Extreme genome scrambling in marine planktonic Oikopleura dioica cryptic species
Source: Genome Res. 2024 Mar;34(3):426–40. doi: 10.1101/gr.278295.123 (PMC11067885; doi:10.1101/gr.278295.123)
Supplement: Supplement 4 [file Supplemental_Data.zip › Supplemental_Data.tar.xz!/Supplemental_Data/articles/OrthogroupSyntenies_files/figure-html/orthopair_plots_suppl-1.pdf]

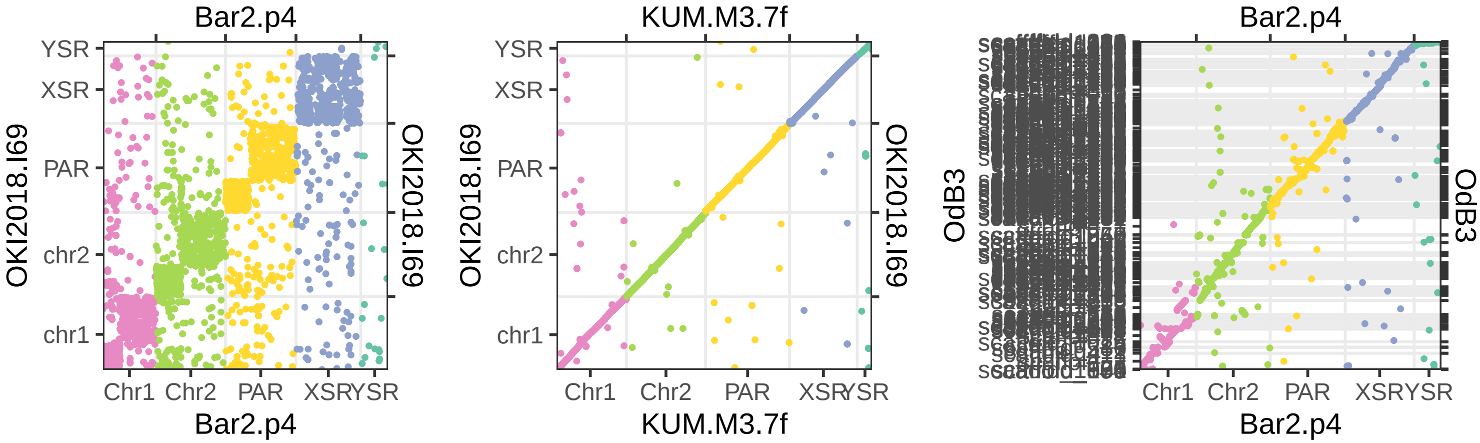

Bar2.p4
YSR
XSR
PAR
chr2
chr1
OKI2018.I69
Chr1
Chr2
PAR
XSR
YSR
Bar2.p4
KUM.M3.7f
YSR
XSR
PAR
chr2
chr1
OKI2018.I69
Chr1
Chr2
PAR
XSR
YSR
KUM.M3.7f
Bar2.p4
OdB3
Chr1
Chr2
PAR
XSR
YSR
Bar2.p4
