# Supplementary material for: Extreme genome scrambling in marine planktonic Oikopleura dioica cryptic species
Source: Genome Res. 2024 Mar;34(3):426–40. doi: 10.1101/gr.278295.123 (PMC11067885; doi:10.1101/gr.278295.123)
Supplement: Supplement 4 [file Supplemental_Data.zip › Supplemental_Data.tar.xz!/Supplemental_Data/articles/SquareWindowPlots.html]

Alignment line plots, 10×10 Mbp windows • OikScrambling
 


  


 


Toggle navigation


OikScrambling
5.1.0

- Get started
- Data load
- Supplemental material
  - Figure 1A DNA line plots
  - Figure 1B chromosome parallel plots
  - Table 1 scrambling index
  - Figure 5 genome-wide feature patterns
  - Figure 7 DNA line plots
  - Figure 7 breakpoint number barplots
- Articles
  - Scaffolding of the AOM-5 genome
  - Scaffolding of the OdB3 genome
  - Conserved non-coding elements
  - Chromosome-scale feature density plots
  - Modifying coalescent cutoffs
  - Regions interrupting colinearity
  - Distance Measures
  - Genomic features of the breakpoints
  - Genomic features of the breakpoints (heatmaps)
  - Inversions
  - Scaffolding of the KUM-M3 genome
  - Load genomic break data
  - Operon conservation analysis
  - Orthogroup syntenies
  - Alignment line plots, whole genomes
  - Region of interest: PAC3
  - Motif search and analysis
  - Whole-chromosome parallel plots
  - Width of the aligned and mapped regions.
  - Regions of interest
  - Repeat regions
  - Scaling breakpoints
  - Segment movement distances
  - Alignment line plots, 10×10 Mbp windows
  - Transcription factor binding sites
  - Tandem site duplications
  - Translocations
- Changelog
- Reference

# Alignment line plots, 10×10 Mbp windows

#### Charles Plessy

#### 06 November, 2023

Source: `vignettes/SquareWindowPlots.Rmd`

`SquareWindowPlots.Rmd`

```
knitr::opts_knit$set(cache = TRUE)
```

## Introduction

This vignette produces alignment line plots of 10 × 10 Mbp windows in
pairs of genomes to illustrate differences in the extent of scrambling
in *Oikopleura* compared to equivalent pairs in other genera. The
size of the windows is fixed to allow comparison at the same scale. To
avoid arbitrary choices, the windows are placed at the centre of the
largest pair of homologous chromosomes (or arms, in case of
*Drosophila*).

**Figure 7 alignment panels were generated in this
vignette.** They are presented on white background.

Extra alignments on grey background show how the non-chromosomal
assemblies were scaffolded by hand in the region selected for the window
plot.

## Setup

### Load R pacakges and data

```
library('OikScrambling') |> suppressPackageStartupMessages()
genomes <- OikScrambling:::loadAllGenomes()
```

```
## Warning in runHook(".onLoad", env, package.lib, package): input string
## 'Génoscope' cannot be translated from 'ANSI_X3.4-1968' to UTF-8, but is valid
## UTF-8

## Warning in runHook(".onLoad", env, package.lib, package): input string
## 'Génoscope' cannot be translated from 'ANSI_X3.4-1968' to UTF-8, but is valid
## UTF-8
```

```
load("BreakPoints.Rdata")
```

### Define custom function

The `mkOxPlotWindow` function searches for the longest
*sequence features* (chromosomes, scaffolds, …) in each genome
and selects a 10 × 10 window centered at their midpoints.

```
mkOxPlotWindow <- function(gb, win = 5e6, size = 2, centre = c('midpoint', 'maxwidth'), ...) {
  centre <- match.arg(centre)
  seqlevels(gb) <- seqlevelsInUse(gb)
  gb <- forceSeqLengths(gb)
  longest_target_seq <- seqlengths(gb) |> sort() |> tail(1) |> names()
  best_query_match   <- gb[seqnames(gb) == longest_target_seq]$query |> seqnames() |> table() |> sort() |> tail(1) |> names()
  if (centre == 'midpoint') {
    target_centre <- round(seqlengths(gb)[longest_target_seq]     / 2)
    query_centre  <- round(seqlengths(gb$query)[best_query_match] / 2)
  } else {
    gb_coa <- coalesce_contigs(gb)
    maxwidth <- which.max(width(gb_coa))
    target_centre <- gb_coa[maxwidth]       |> resize(1, fix = "center") |> start()
    query_centre  <- gb_coa$query[maxwidth] |> resize(1, fix = "center") |> start()
  }
  gb <- gb |> plyranges::filter(
    seqnames == longest_target_seq,
    seqnames(query) == best_query_match,
    start > target_centre - win,
    end < target_centre + win,
    start(query) > query_centre - win,
    end(query) < query_centre + win
  )
  # score(gb) <- stringDist_GBreaks(gb) / width(gb)
  makeOxfordPlots(gb, col = "strand", size = size, ...) +
    theme_bw() +
    theme(legend.position="none")
}
```

## Oikopleura

### Okinawa vs Osaka

66.12972 % (matches / alignment widths)

```
mkOxPlotWindow(gbs$Oki_Osa)
```

### Okinawa vs Barcelona

66.99999 %

```
mkOxPlotWindow(gbs$Oki_Bar)
```

### Osaka vs Barcelona

```
mkOxPlotWindow(gbs$Osa_Bar)
```

### Osaka vs Okinawa

```
mkOxPlotWindow(gbs$Osa_Oki)
```

### Osaka vs Aomori

94.45642 % matches / alignment length

The Aomori genome needs some custom scaffolding to make a contiguous
10 × 10 region.

```
Osa_Aom_PAR <- gbs$Osa_Aom |> plyranges::filter(seqnames == "PAR")
seqlevels(Osa_Aom_PAR)       <- seqlevelsInUse(Osa_Aom_PAR)
seqlevels(Osa_Aom_PAR$query) <- seqlevelsInUse(Osa_Aom_PAR$query)

makeOxfordPlots(Osa_Aom_PAR)
```

```
# Let's remove the contigs that have their main match elsewhere.
QTcoverage <- function(gb) {
  stopifnot (length(seqlevels(gb)) == 1) # Not ready for full objects
  stopifnot (!any(is.na(seqlengths(gb$query))))
  grl <- split(gb, seqnames(gb$query))
  lapply(grl, \(gb) sum(width(gb$query))) |> unlist() / seqlengths(gb$query)
}
Osa_Aom_PAR[seqnames(Osa_Aom_PAR$query) %in% seqlevels(Osa_Aom_PAR$query)[QTcoverage(Osa_Aom_PAR) < 0.5]] <- NULL
seqlevels(Osa_Aom_PAR$query) <- seqlevelsInUse(Osa_Aom_PAR$query)
makeOxfordPlots(Osa_Aom_PAR)
```

```
# Let's remove the smallest ones that barely display on one pixel
seqlengths(Osa_Aom_PAR$query)
```

```
## contig_12_1 contig_15_1 contig_34_1 contig_38_1 contig_49_1 
##     7097394     6419763     1209792      203373     1316631
```

```
Osa_Aom_PAR[seqnames(Osa_Aom_PAR$query) %in% seqlevels(Osa_Aom_PAR$query)[seqlengths(Osa_Aom_PAR$query) < 2e4]] <- NULL
seqlevels(Osa_Aom_PAR$query) <- seqlevelsInUse(Osa_Aom_PAR$query)
makeOxfordPlots(Osa_Aom_PAR, col = "strand")
```

```
# And now let's flip by hand the ones that need
grl <- split(Osa_Aom_PAR, seqnames(Osa_Aom_PAR$query))
grl[["contig_38_1"]] <- reverse(grl[["contig_38_1"]], query = TRUE)
grl[["contig_49_1"]] <- reverse(grl[["contig_49_1"]], query = TRUE)
Osa_Aom_PAR <- unlist(grl)
makeOxfordPlots(Osa_Aom_PAR, col = "strand")
```

```
# Finally, let's reorder and merge remaining seqlevels
seqlevels(Osa_Aom_PAR$query) <- seqlevels(Osa_Aom_PAR$query)[orderQuerySeqLevels(Osa_Aom_PAR)]
Osa_Aom_PAR$query <- mergeSeqLevels(Osa_Aom_PAR$query, seqlevels(Osa_Aom_PAR$query), "PAR")
```

```
mkOxPlotWindow(Osa_Aom_PAR)
```

### Okinawa vs Kume

94.23956 % matches / alignment length

Same for the Kume genome…

```
Oki_Kum_PAR <- gbs$Oki_Kum |> plyranges::filter(seqnames == "PAR")
seqlevels(Oki_Kum_PAR)       <- seqlevelsInUse(Oki_Kum_PAR)
seqlevels(Oki_Kum_PAR$query) <- seqlevelsInUse(Oki_Kum_PAR$query)

makeOxfordPlots(Oki_Kum_PAR)
```

```
# Let's remove the contigs that have their main match elsewhere.
QTcoverage <- function(gb) {
  stopifnot (length(seqlevels(gb)) == 1) # Not ready for full objects
  stopifnot (!any(is.na(seqlengths(gb$query))))
  grl <- split(gb, seqnames(gb$query))
  lapply(grl, \(gb) sum(width(gb$query))) |> unlist() / seqlengths(gb$query)
}
Oki_Kum_PAR[seqnames(Oki_Kum_PAR$query) %in% seqlevels(Oki_Kum_PAR$query)[QTcoverage(Oki_Kum_PAR) < 0.5]] <- NULL
seqlevels(Oki_Kum_PAR$query) <- seqlevelsInUse(Oki_Kum_PAR$query)
makeOxfordPlots(Oki_Kum_PAR)
```

```
# Let's remove the smallest ones that barely display on one pixel
seqlengths(Oki_Kum_PAR$query)
```

```
## contig_12_1 contig_13_1 contig_18_1 contig_28_1 contig_38_1 contig_41_1 
##     2357322      793615         832     6415228       61242     2611992 
## contig_43_1 contig_61_1 contig_72_1 contig_82_1 contig_85_1 contig_89_1 
##      841236       17591       84743     2322619      428051      241503 
## contig_91_1 
##      126775
```

```
Oki_Kum_PAR[seqnames(Oki_Kum_PAR$query) %in% seqlevels(Oki_Kum_PAR$query)[seqlengths(Oki_Kum_PAR$query) < 2e4]] <- NULL
seqlevels(Oki_Kum_PAR$query) <- seqlevelsInUse(Oki_Kum_PAR$query)
makeOxfordPlots(Oki_Kum_PAR)
```

```
# And now let's flip by hand the ones that need
grl <- split(Oki_Kum_PAR, seqnames(Oki_Kum_PAR$query))
grl[["contig_12_1"]] <- reverse(grl[["contig_12_1"]], query = TRUE)
grl[["contig_43_1"]] <- reverse(grl[["contig_43_1"]], query = TRUE)
Oki_Kum_PAR <- unlist(grl)
makeOxfordPlots(Oki_Kum_PAR)
```

```
# Finally, let's reorder and merge remaining seqlevels
seqlevels(Oki_Kum_PAR$query) <- seqlevels(Oki_Kum_PAR$query)[orderQuerySeqLevels(Oki_Kum_PAR)]
Oki_Kum_PAR$query <- mergeSeqLevels(Oki_Kum_PAR$query, seqlevels(Oki_Kum_PAR$query), "PAR")
```

```
mkOxPlotWindow(Oki_Kum_PAR)
```

### Barcelona vs Norway

No plot, but 95.91132 % matches / alignment length

## Ciona

### *Ciona intestinalis* (Plymouth vs Roscoff)

```
mkOxPlotWindow(gbs$Ply_Ros) + ggtitle("Ciona intestinalis Plymouth vs Roscoff")
```

### *Ciona intestinalis* (Plymouth) vs *Ciona robusta*

```
mkOxPlotWindow(gbs$Ply_Rob) + ggtitle("Ciona intestinalis Plymouth vs Ciona robusta")
```

### *Ciona intestinalis* (Plymouth) vs *Ciona savignyi*

Here again we need some custom scaffolding.

```
Ply_Sav_chr1 <- gbs$Ply_Sav |> plyranges::filter(seqnames == "BNJZ01000001.1")
seqlevels(Ply_Sav_chr1)       <- seqlevelsInUse(Ply_Sav_chr1)
seqlevels(Ply_Sav_chr1$query) <- seqlevelsInUse(Ply_Sav_chr1$query)
Ply_Sav_chr1$query <- forceSeqLengths(Ply_Sav_chr1$query)

makeOxfordPlots(Ply_Sav_chr1)
```

```
# Let's remove the contigs that have their main match elsewhere.
QTcoverage <- function(gb) {
  stopifnot (length(seqlevels(gb)) == 1) # Not ready for full objects
  stopifnot (!any(is.na(seqlengths(gb$query))))
  grl <- split(gb, seqnames(gb$query))
  lapply(grl, \(gb) sum(width(gb$query))) |> unlist() / seqlengths(gb$query)
}
Ply_Sav_chr1[seqnames(Ply_Sav_chr1$query) %in% seqlevels(Ply_Sav_chr1$query)[QTcoverage(Ply_Sav_chr1) < 0.05]] <- NULL
seqlevels(Ply_Sav_chr1$query) <- seqlevelsInUse(Ply_Sav_chr1$query)
makeOxfordPlots(Ply_Sav_chr1)
```

```
# Let's remove the smallest ones that barely display on one pixel
seqlengths(Ply_Sav_chr1$query)
```

```
##   R1063    R113   R1167   R1383     R14   R1490   R1596    R162    R173   R1779 
##   24606  277504   10138    7422 1702960    3223    8495  276690  455223   32296 
##   R1942   R2368   R2407   R2517    R268     R27    R281   R2872     R30     R31 
##   10471    6428    9904    5720  550239 2366314  114725    8506 3493700  766944 
##    R347    R352    R417    R489    R491      R5     R51     R62     R64    R650 
##  180004   46220   61968  180871   28116 2728683 1639093  608627  540266   23432 
##    R767     R98 
##   42085  434095
```

```
Ply_Sav_chr1[seqnames(Ply_Sav_chr1$query) %in% seqlevels(Ply_Sav_chr1$query)[seqlengths(Ply_Sav_chr1$query) < 2e4]] <- NULL
seqlevels(Ply_Sav_chr1$query) <- seqlevelsInUse(Ply_Sav_chr1$query)
makeOxfordPlots(Ply_Sav_chr1)
```

```
# Finally, let's reorder and merge remaining seqlevels
seqlevels(Ply_Sav_chr1$query) <- seqlevels(Ply_Sav_chr1$query)[orderQuerySeqLevels(Ply_Sav_chr1)]
Ply_Sav_chr1$query <- mergeSeqLevels(Ply_Sav_chr1$query, seqlevels(Ply_Sav_chr1$query), "Merged matching contigs")
makeOxfordPlots(Ply_Sav_chr1)
```

```
mkOxPlotWindow(Ply_Sav_chr1) + ggtitle("Ciona intestinalis Plymouth vs Ciona savignyi")
```

## *Drosophila*

*Note that in some genomes, each chromosome arm is represented in
a separate sequence*.

### *D. melanogaster* vs *D. mauritania*

```
# Force strand flipping for aesthetic purpose
mkOxPlotWindow(gbs$Dme_Dma |> forceSeqLengths() |> reverse()) + ggtitle("Drosophila melanogaster vs Drosophila mauritania")
```

### *D. melanogaster* vs *D. yakuba*

```
mkOxPlotWindow(gbs$Dme_Dya) + ggtitle("Drosophila melanogaster vs Drosophila yakuba")
```

### *D. melanogaster* vs *D. subpulchrella*

On arm 3R there is a large unaligned region that causes the plot to
not be squared. I think that the problem may be related to the presence
of large centromere regions in the best assemblies.

```
mkOxPlotWindow(gbs$Dme_Dsu) + ggtitle("Drosophila melanogaster vs Drosophila subpulchrella")
```

Blacklist 3R as the algorithm does not find an nicely square window
on it.

```
mkOxPlotWindow(gbs$Dme_Dsu |> plyranges::filter(seqnames != "3R")) + ggtitle("Drosophila melanogaster vs Drosophila subpulchrella")
```

### *D. melanogaster* vs *D. buskii*

```
mkOxPlotWindow(gbs$Dme_Dbu) + ggtitle("Drosophila melanogaster vs Drosophila buskii")
```

## Mammals

### Human versus mouse

Chromosome 18 was chosen because it is small and it has the same name
in both species. Region was centered on the alignment with the longest
width, as otherwise the window is out of target.

Still, most of the region is not aligned. The syntenic regions only
look contiguous because the dots need to be thick to be easy to see.

```
hgmm <- load_genomic_breaks(system.file("extdata/hg38_mm10/hg38_chr18__mm10_chr18.gff3", package = "BreakpointsData"))
mkOxPlotWindow(hgmm, centre = 'maxwidth') + ggtitle("Homo sapiens vs Mus musculus")
```

```
## Warning in min(x): no non-missing arguments to min; returning Inf
```

```
## Warning in max(x): no non-missing arguments to max; returning -Inf
```

```
## Warning in min(x): no non-missing arguments to min; returning Inf
```

```
## Warning in max(x): no non-missing arguments to max; returning -Inf
```

### Muntjaks

```
Mmun_Mree <- load_genomic_breaks(system.file("extdata/Muntjaks/Rrev_Rmun.07.postmasked.gff.gz", package = "BreakpointsData"), type = "match")
# Given the enormous differences of karyotype, the M. reevesi chromosomes need to be trimmed for # 
# the window centering alorithm to function.
Mmun_Mree.trim <- Mmun_Mree |> plyranges::filter(seqnames == "CM018478.1", seqnames(query) == "CM018501.1", end(query) < 273507479)
mkOxPlotWindow(Mmun_Mree.trim, centre = 'maxwidth') + ggtitle("Muntiacus muntjak vs M. reevesi (95% aligned, 98% identical).")
```

```
## Warning in min(x): no non-missing arguments to min; returning Inf
```

```
## Warning in max(x): no non-missing arguments to max; returning -Inf
```

```
## Warning in min(x): no non-missing arguments to min; returning Inf
```

```
## Warning in max(x): no non-missing arguments to max; returning -Inf
```

## Nematodes

*Caenorhabditis briggsae* and *C. elegans* are
hermaphrodites, so let’s avoid using them as target genomes.

But *C. briggsae* is closest to *C. nigoni* so we need
it once as a query genome.

### *C. nigoni* vs *C. briggsae*

*C. nigoni* genome assembly `GCA_027920645.1`
length: 128,508,886. It has no unplaced contigs nor mitochondrial
genome.

*C. briggsae*’s genome assmebly `GCA_022453885.1`
length is 106,953,292, with mitochondrial genome as I forgot to remove
it.

```
mkOxPlotWindow(gbs$Cni_Cbr, centre = 'maxwidth') + ggtitle("Caenorhabditis nigoni vs C. briggsae (61% aligned, 85% identity)")
```

```
mkOxPlotWindow(gbs$Cbr_Cni, centre = 'maxwidth') + ggtitle("Caenorhabditis briggsae vs C. nigoni (69% aligned, 85% identity)")
```

### *C. nigoni* or *briggsae* vs *C. remanei*

Unplaced contigs and mitochondrial genome were removed from *C.
remanei*’s assembly `GCA_001643735.4`.

```
mkOxPlotWindow(gbs$Cni_Cre, centre = 'maxwidth') + ggtitle("Caenorhabditis nigoni vs C. remanei (25% aligned, 73% identity)")
```

```
## Warning in min(x): no non-missing arguments to min; returning Inf
```

```
## Warning in max(x): no non-missing arguments to max; returning -Inf
```

```
mkOxPlotWindow(gbs$Cbr_Cre, centre = 'maxwidth') + ggtitle("Caenorhabditis briggsae vs C. remanei (30% aligned, 72% identity)")
```

```
## Warning in min(x): no non-missing arguments to min; returning Inf
```

```
## Warning in max(x): no non-missing arguments to max; returning -Inf
```

### *C. nigoni* vs *C. inopinata*

```
mkOxPlotWindow(gbs$Cni_Cin, centre = 'maxwidth') + ggtitle("Caenorhabditis nigoni vs C. inopinata (16% aligned, 72% identity)")
```

```
## Warning in min(x): no non-missing arguments to min; returning Inf
```

```
## Warning in max(x): no non-missing arguments to max; returning -Inf
```

### \_C. briggsae vs *C. elegans*

```
mkOxPlotWindow(gbs$Cbr_Cel, centre = 'maxwidth') + ggtitle("Caenorhabditis briggsae vs C. elegans (23% aligned, 72% identity)")
```

```
## Warning in min(x): no non-missing arguments to min; returning Inf
```

```
## Warning in max(x): no non-missing arguments to max; returning -Inf
```

```
## Warning in min(x): no non-missing arguments to min; returning Inf
```

```
## Warning in max(x): no non-missing arguments to max; returning -Inf
```

## Contents

Developed by Aleksandra Bliznina, Michael Mansfield, Charles Plessy.

Site built with pkgdown 2.0.7.
