# Supplementary material for: Extreme genome scrambling in marine planktonic Oikopleura dioica cryptic species
Source: Genome Res. 2024 Mar;34(3):426–40. doi: 10.1101/gr.278295.123 (PMC11067885; doi:10.1101/gr.278295.123)
Supplement: Supplement 4 [file Supplemental_Data.zip › Supplemental_Data.tar.xz!/Supplemental_Data/articles/GenomicFeatures_files/figure-html/genomic_features_byType-1.pdf]

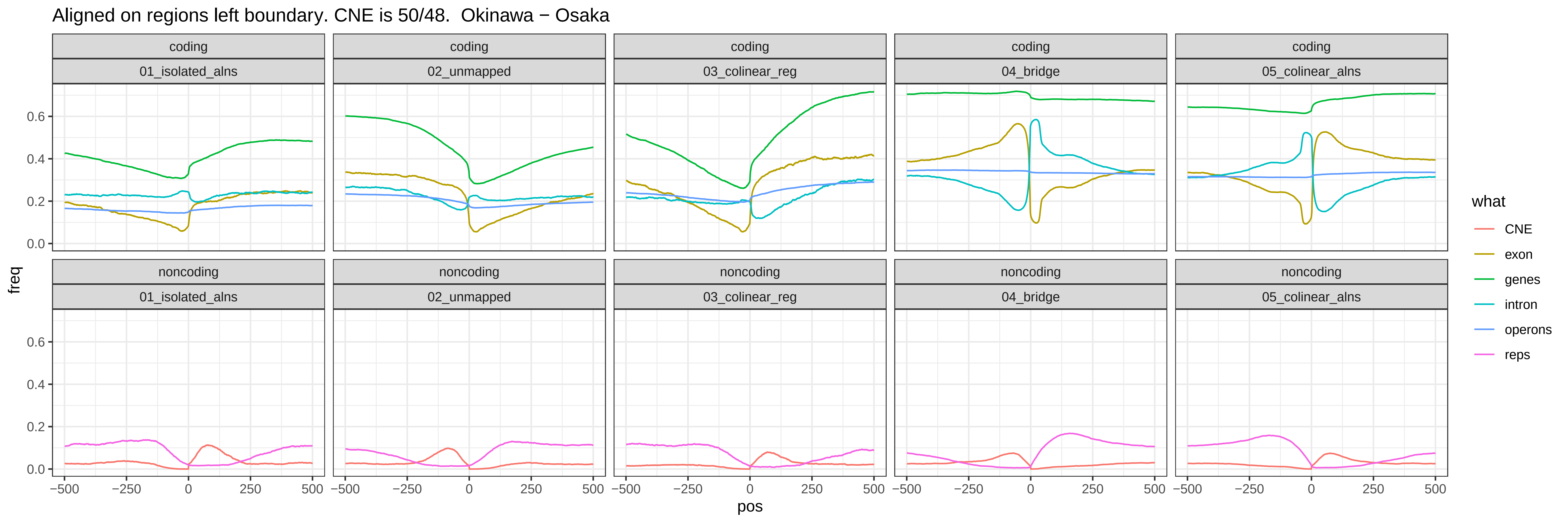

Aligned on regions left boundary. CNE is 50/48. Okinawa − Osaka
coding
01_isolated_alns
02_unmapped
03_colinear_reg
04_bridge
05_colinear_alns
noncoding
freq
pos
0.0
0.2
0.4
0.6
−500
−250
0
250
500
what
CNE
exon
genes
intron
operons
reps
