# Supplementary material for: Extreme genome scrambling in marine planktonic Oikopleura dioica cryptic species
Source: Genome Res. 2024 Mar;34(3):426–40. doi: 10.1101/gr.278295.123 (PMC11067885; doi:10.1101/gr.278295.123)
Supplement: Supplement 4 [file Supplemental_Data.zip › Supplemental_Data.tar.xz!/Supplemental_Data/articles/GenomicFeatures_files/figure-html/operon_scrambling-1.pdf]

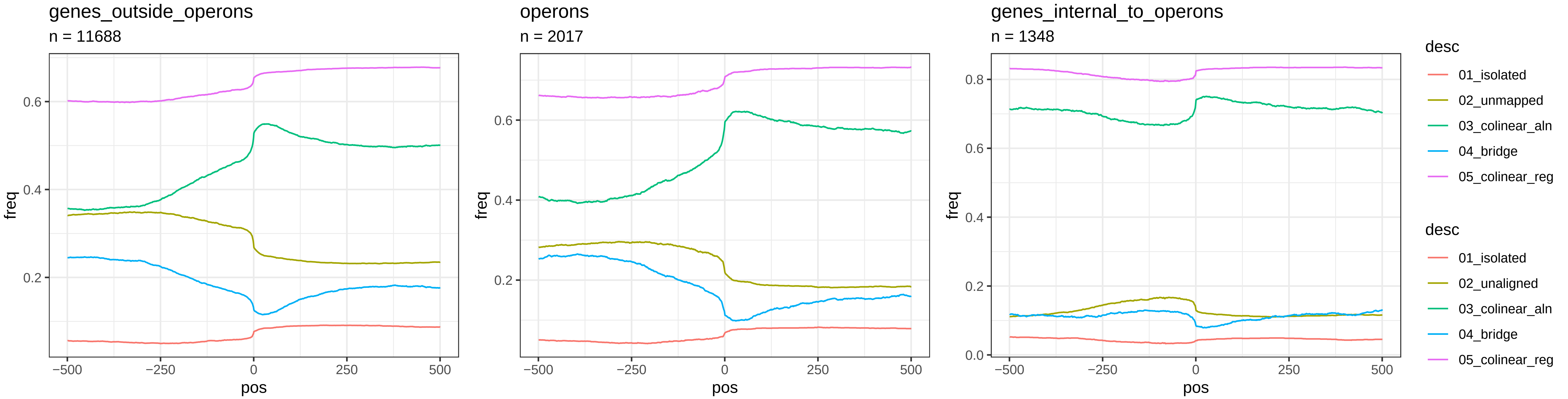

genes_outside_operons
n = 11688
operons
n = 2017
genes_internal_to_operons
n = 1348
freq
pos
0.6
0.4
0.2
0.8
0.0
−500
−250
0
250
500
desc
01_isolated
02_unmapped
03_colinear_aln
04_bridge
05_colinear_reg
desc
01_isolated
02_unaligned
03_colinear_aln
04_bridge
05_colinear_reg
