# Supplementary material for: Extreme genome scrambling in marine planktonic Oikopleura dioica cryptic species
Source: Genome Res. 2024 Mar;34(3):426–40. doi: 10.1101/gr.278295.123 (PMC11067885; doi:10.1101/gr.278295.123)
Supplement: Supplement 4 [file Supplemental_Data.zip › Supplemental_Data.tar.xz!/Supplemental_Data/articles/GenomicFeatures_files/figure-html/genomic_features_byType_Oki-1.pdf]

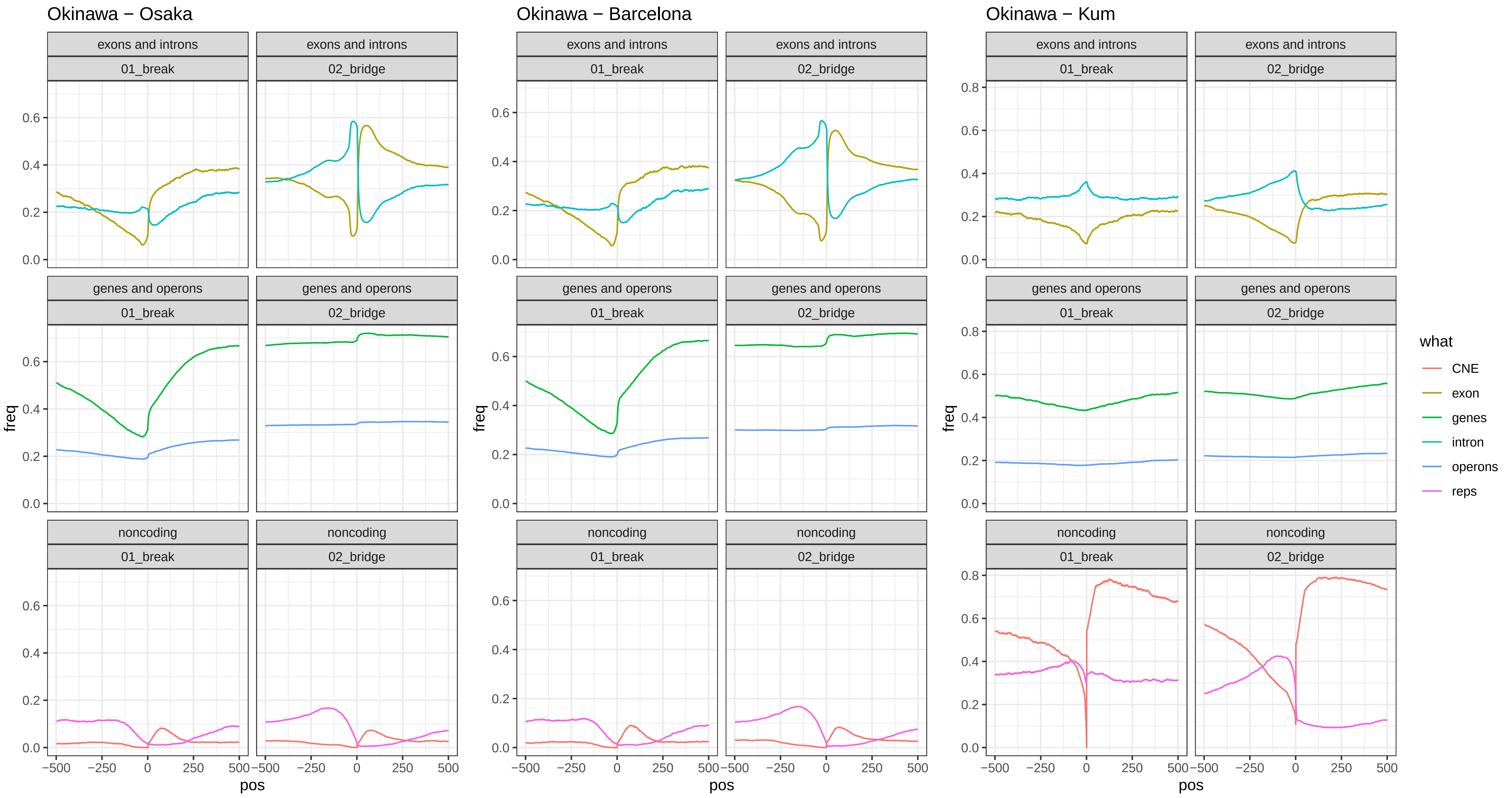

Okinawa − Osaka
Okinawa − Barcelona
Okinawa − Kum
exons and introns
genes and operons
noncoding
01_break
02_bridge
freq
pos
what
CNE
exon
genes
intron
operons
reps
