# Supplementary material for: Extreme genome scrambling in marine planktonic Oikopleura dioica cryptic species
Source: Genome Res. 2024 Mar;34(3):426–40. doi: 10.1101/gr.278295.123 (PMC11067885; doi:10.1101/gr.278295.123)
Supplement: Supplement 4 [file Supplemental_Data.zip › Supplemental_Data.tar.xz!/Supplemental_Data/articles/GenomicFeatures.html]

Genomic features of the breakpoints • OikScrambling
 


  


 


Toggle navigation


OikScrambling
5.1.0

- Get started
- Data load
- Supplemental material
  - Figure 1B and S1 DNA line plots
  - Figure 1C chromosome parallel plots
  - Figure 1D scrambling index
  - Figure 2A and S1 orthogroup dot plots and synteny block histograms
  - Figure 3C breakpoint feature density plots
  - Figure 4B and 4C operon barplots
  - Figure 5 genome-wide feature patterns
  - Figure 7A–D DNA line plots
  - Figure 7E breakpoint number barplots
- Articles
  - Scaffolding of the AOM-5 genome
  - Scaffolding of the OdB3 genome
  - Conserved non-coding elements
  - Chromosome-scale feature density plots
  - Modifying coalescent cutoffs
  - Regions interrupting colinearity
  - Distance Measures
  - Genomic features of the breakpoints
  - Genomic features of the breakpoints (heatmaps)
  - Inversions
  - Scaffolding of the KUM-M3 genome
  - Load genomic break data
  - Operon conservation analysis
  - Operon detection and counting
  - Orthogroup syntenies
  - Alignment line plots, whole genomes
  - Region of interest: PAC3
  - Motif search and analysis
  - Whole-chromosome parallel plots
  - Width of the aligned and mapped regions.
  - Regions of interest
  - Repeat regions
  - Scaling breakpoints
  - Segment movement distances
  - Alignment line plots, 10×10 Mbp windows
  - Transcription factor binding sites
  - Tandem site duplications
  - Translocations
- Changelog
- Reference

# Genomic features of the breakpoints

#### Charlotte West

#### Charles Plessy

#### 20 November, 2023

Source: `vignettes/GenomicFeatures.Rmd`

`GenomicFeatures.Rmd`

## Introduction

Breakpoint feature analyses.

**Figure 3 panel C is produced by this vignette.**

It also computes operons as we need their boundaries in the figure
panel.

\*\* Figure 4 panel C is produced by this vignette\*\*

The vignette also contains extra material that is not entirely
relevant to the manuscript, but that can not be moved elsewhere because
it needs the orthogroup `GBreaks` object generated at the
beginning at the top.

### Load packages and data

See `?OikScrambling:::loadAllGenomes`,
`?OikScrambling:::loadAllTranscriptsGR`, and
`vignette("LoadGenomicBreaks", package = "OikScrambling")`
for how the different objects are prepared.

```
library('OikScrambling')   |> suppressPackageStartupMessages()
library('GenomicFeatures') |> suppressPackageStartupMessages()
library('heatmaps')        |> suppressPackageStartupMessages()
library('patchwork')       |> suppressPackageStartupMessages()
genomes     <- OikScrambling:::loadAllGenomes(compat = F)
```

```
## Warning in runHook(".onLoad", env, package.lib, package): input string
## 'Génoscope' cannot be translated from 'ANSI_X3.4-1968' to UTF-8, but is valid
## UTF-8

## Warning in runHook(".onLoad", env, package.lib, package): input string
## 'Génoscope' cannot be translated from 'ANSI_X3.4-1968' to UTF-8, but is valid
## UTF-8
```

```
# Cannot use Knitr cache as long as "annot" objects are used.
annots      <- OikScrambling:::loadAllAnnotations()   |> suppressWarnings()
reps        <- OikScrambling:::loadAllRepeats(compat = F)
genes       <- sapply(annots, function(a) sort(genes(a))) |> SimpleList()
load("BreakPoints.Rdata")
requireNamespace("rGADEM")    |> suppressPackageStartupMessages()
requireNamespace("ggseqlogo") |> suppressPackageStartupMessages()
ces <- readRDS("CEs.Rds")
tfbs <- readRDS("pwmMatchesOki_12_95.Rds")
```

### Compute operons

See the
`vignette("OperonDetection", package = "OikScrambling")` for
details.

```
operons <- lapply(annots, \(a) {a |> genes() |> OikScrambling:::calcOperons(window = 500) }) |> SimpleList() |> suppressWarnings()
operons$Nor <- OikScrambling:::calcOperons(window = 500, transcripts(annots$Nor) |> reduce()) |> suppressWarnings()
sapply(operons, length)
```

```
##  Oki  Osa  Bar  Kum  Aom  Nor  Ply  Ros 
## 3124 2741 2379 2974 2648 2996 1088 1472
```

```
sapply(operons, \(o) {table(o$n)})
```

```
## $Oki
## 
##    2    3    4    5    6    7    8    9   10   11   14 
## 1943  647  259  134   69   33   19   10    6    3    1 
## 
## $Osa
## 
##    2    3    4    5    6    7    8    9   10   11   12   13 
## 1714  557  248   98   63   32   13   10    1    3    1    1 
## 
## $Bar
## 
##    2    3    4    5    6    7    8    9   10   11   12   13   14   17 
## 1450  475  221  106   57   30   23    9    3    1    1    1    1    1 
## 
## $Kum
## 
##    2    3    4    5    6    7    8    9   10   11   12   13   14   17 
## 1855  648  233  114   56   28   18    4    7    6    1    1    2    1 
## 
## $Aom
## 
##    2    3    4    5    6    7    8    9   10   11   12   14 
## 1689  492  239  107   52   36   14   10    3    4    1    1 
## 
## $Nor
## 
##    2    3    4    5    6    7    8    9   10   11   12   13   14   15   16   17 
## 1724  562  264  138   93   65   38   40   16   21   11   10    3    4    3    1 
##   20   22 
##    1    2 
## 
## $Ply
## 
##   2   3   4   5   6   7   8   9  13  14  15 
## 937 114  22   6   2   1   1   1   1   1   2 
## 
## $Ros
## 
##    2    3    4    5    6    7    9   10   11   21   26   29 
## 1262  163   33    5    1    1    1    1    1    1    1    2
```

## Gene feature coverage around aligned and unaligned genomic regions.

### Accessory functions

```
prepareFreqPlot <- function(gb, annot = NULL, rep = NULL, cne = NULL, tfbs = NULL, operons = NULL, win = 1000, direction = "left", withGenes=c("yes", "no", "only")) {
  withGenes <- match.arg(withGenes)
  l <- list()
  # Note that get_bps(direction="mid") is probably not what you think.
  if(direction == "mid") {
    gb <- resize(gb, 1, "center")
    direction <- "left"
  }
  if (!is.null(annot)) {
    if (withGenes %in% c("yes", "only")) {
      l$genes  <- feature_coverage(gb,     genes(annot),         win = win, lab = "genes",  direction = direction)
    }
    if (withGenes %in% c("yes", "no")) {
      l$intron <- feature_coverage(gb,     intronicParts(annot), win = win, lab = "intron", direction = direction)
      l$exon   <- feature_coverage(gb,     exonicParts(annot),   win = win, lab = "exon",   direction = direction)
      #reorder
      newOrder <- order(rowSums(l$intron@image))
      l$intron@image <- l$intron@image[newOrder,]
      l$exon@image   <- l$exon@image[newOrder,]
    }
  }
  if (!is.null(operons))
    l$operons<-feature_coverage(gb,    operons,              win = win, lab = "operons",direction = direction)
  if (!is.null(rep))
    l$reps   <- feature_coverage(gb,   rep,                  win = win, lab = "repeats",direction = direction)
  if (!is.null(cne))
    l$CNE   <- feature_coverage(gb,    cne,                  win = win, lab = "CNE",    direction = direction)
  if (!is.null(tfbs))
    l$tfbs  <- feature_coverage(gb,    tfbs,                 win = win, lab = "TFBS",   direction = direction)
  l
}

#' Prepare a long tibble for frequency plotting
#' 
#' Loop over a list of `Heatmap` objects to produce a long tibble that can be
#' used with ggplot to plot frequency of each feature included in the list.
#'
#' @param x A list of `Heatmap` objects
#' @param desc An additional description (character string)
#'
#' @return A long `tibble` that is the vertical concatenation of all tibbles
#' produced for each element of the list `x`.  Its `what` column is made from
#' the names of the heatmaps in the list, and its `desc` column is made from the
#' `desc` arguments.  The other columns (`val`, `freq` and `pos`) originate from
#' the `Heatmap_to_freq_tibble` function call.
prepareGGFreqPlot <- function(x, desc = NULL) {
  l <- lapply(names(x), \(name){
    tib <- Heatmap_to_freq_tibble(x[[name]], desc = desc)
    tib$what <- name
    tib
  })
   do.call(rbind, l)
}

#' Heatmap to long tibble for ggplot
#' 
#' Coerce a Heatmap object into a \dQuote{long} tibble with one value per
#' position, either as raw counts or as a frequency.
#' 
#' The tibble can then be passed to ggplot.  The `image` matrix of the `Heatmap`
#' object has one column per nucleotide position and it is assumed that its
#' central column is for position zero.
#'
#' @param h a `Heatmap` object
#'
#' @return a `tibble` with `val`, `freq` and `pos` columns
Heatmap_to_freq_tibble <- function(h, desc = NULL) {
  val  <- colSums(h@image)
  tibble::tibble( val  = val
                , freq = val / nrow(h@image)
                , pos  = 1:ncol(h@image) - round(ncol(h@image)) / 2
                , desc = desc)
}
```

### Centering on left-aligned boundaries.

Breakpoints are between mapped regions. If the unmapped region is
broad we have little information where the breakpoint originally was. We
set arbitrarly the breakpoints to the left-side (end-side) coordinate of
genomic regions.

Mapped regions can consist of multiple colinear aligned regions, or
be a single uncoalesced aligned region. We will see that they have
different properties.

Our annotations is mostly made of coding exons, so the features
*exons* and *cds* have very similar profiles. Therefore we
keep only *exons* in the rest of the analysis.

Positive coordinates (and zero) start in selected regions and
negative coordinates start outside these regions. As we move further
from 0, we can not guarantee that the statement is still true because
some regions have a width narrower than the plot window. This is why the
values tend to equilibrate away from the boundaries.

Aligned regions are enriched in exons and depleted in introns.

Close to the breakpoint of uncoalesced mapped regions, exon
annotations are depleted, which makes sense, as exons are usually
mapable. Within the mapped regions, frequency of introns and exons are
comparable. In contrary, within coalesced mapped regions, the exon
annotations are over-represented.

```
#' Pre-compute feature frequencies in fixed windows
#'
#' @param target 3-letter name of one Oik genome
#' @param query 3-letter name of another Oik genome
#' @param perpareFreqPlot a `perpareFreqPlot` function
#' @param reps `SimpleList` of `GRanges` of repeat regions
#' @param CE `SimpleList` of `GRanges` with conserved non-coding elements
#' @param tfbs `SimpleList` of `GRanges` of putative transcription factor binding sites
#' @param operons `SimpleList` of `GRanges` of operon regions.
#' @param direction Where to center the windows (`left`, `right` or `mid`)
#'
#' @return A `SimpleList` of Heatmap objects
computePlotData <- function(target, query, annots=NULL, reps=NULL, CE=NULL, tfbs=NULL, operons=NULL, direction="left") {
  pair <- paste(target, query, sep = "_")
  current_GB_aln_nonCoa     <- gbs[[pair]][   gbs[[pair]]$nonCoa]
  current_GB_aln_coa        <- gbs[[pair]][ ! gbs[[pair]]$nonCoa]
  current_GB_syn_block      <- coa[[pair]][ ! coa[[pair]]$nonCoa]
  # We do not use unal, because it overlaps with bri
  current_GB_unmap          <- unmap[[pair]]
  current_GB_bri        <- bri[[pair]]
  
  targetGenome <- unique(genome(gbs[[pair]]))
  
  prepareFreqPlot_default <- \(x) prepareFreqPlot(x, annot = annots[[target]], rep = reps[[targetGenome]], cne = CE[[pair]], tfbs = tfbs, operons = operons[[target]], direction = direction)
  
  hmList <- SimpleList()
  hmList$aln_nonCoa <- prepareFreqPlot_default(current_GB_aln_nonCoa)
  hmList$aln_coa    <- prepareFreqPlot_default(current_GB_aln_coa)
  hmList$syn_block  <- prepareFreqPlot_default(current_GB_syn_block)
  hmList$unmap      <- prepareFreqPlot_default(current_GB_unmap)
  hmList$bri        <- prepareFreqPlot_default(current_GB_bri)
  hmList
}

hmList_to_plot_noncoding <- computePlotData("Oki", "Osa", reps = reps, CE = ces$ce__50__48)
hmList_to_plot_coding    <- computePlotData("Oki", "Osa", annots = annots, operons = operons)

list_of_heatmaps_to_long_tibble <- function(hmList_to_plot, desc = NULL) {
  tib <-rbind(
    hmList_to_plot$aln_nonCoa  |> prepareGGFreqPlot("01_isolated_alns"),
    hmList_to_plot$unmap       |> prepareGGFreqPlot("02_unmapped"),
    hmList_to_plot$syn_block   |> prepareGGFreqPlot("03_colinear_reg"),
    hmList_to_plot$bri         |> prepareGGFreqPlot("04_bridge"),
    hmList_to_plot$aln_coa     |> prepareGGFreqPlot("05_colinear_alns")
  )
  tib$desc2 <- desc
  tib
}

rbind(
  list_of_heatmaps_to_long_tibble(hmList_to_plot_noncoding, "noncoding"),
  list_of_heatmaps_to_long_tibble(hmList_to_plot_coding, "coding")
) |> ggplot() +
  aes(pos, freq, col = what) +
  geom_line() +
  facet_wrap(~desc2 + desc, nrow = 2) +
  theme_bw() +
  ggtitle("Aligned on regions left boundary. CNE is 50/48.  Okinawa - Osaka")
```

Most of the narrow-width aligned regions did not coalesce. We use
this as a classifier instead of an arbitrary width cutoff. See
`vignette("RegionWidths", package = "OikScrambling")` for
details.

## Figure 3C panel

,With only 2 panels, for unmapped–aligned and bridge–aligned, we
display almost all the information present in the 5-panel version
above.

```
computePlotData_simplified <- function(target, query, annots=NULL, reps=NULL, CE=NULL, tfbs=NULL, operons=NULL, direction="left", withGenes = c("yes", "no", "only")) {
  withGenes <- match.arg(withGenes)
  pair <- paste(target, query, sep = "_")
  current_GB_brk <- unmap[[pair]] |> subsetByOverlaps(coa[[pair]][!coa[[pair]]$nonCoa] + 1) # The unmapped region flanking colinear regions
  current_GB_bri <- bri[[pair]]
  
  targetGenome <- unique(genome(gbs[[pair]]))
  
  prepareFreqPlot_default <- \(x) prepareFreqPlot(x, annot = annots[[target]], rep = reps[[targetGenome]], cne = CE[[pair]], tfbs = tfbs, operons = operons[[target]], direction = direction, withGenes = withGenes)
  
  hmList <- SimpleList()
  hmList$brk <- prepareFreqPlot_default(current_GB_brk)
  hmList$bri <- prepareFreqPlot_default(current_GB_bri)
  hmList
}

list_of_heatmaps_to_long_tibble <- function(hmList_to_plot, desc = NULL) {
  tib <-rbind(
    hmList_to_plot$brk         |> prepareGGFreqPlot("01_break"),
    hmList_to_plot$bri         |> prepareGGFreqPlot("02_bridge")
  )
  tib$desc2 <- desc
  tib
}

twoBySixFigure <- function(noncod, genesop, exonintron)
  rbind(
  list_of_heatmaps_to_long_tibble(noncod,    "noncoding"),
  list_of_heatmaps_to_long_tibble(genesop, "genes and operons"),
  list_of_heatmaps_to_long_tibble(exonintron,   "exons and introns")
) |> ggplot() +
  aes(pos, freq, col = what) +
  geom_line() +
  facet_wrap(~desc2 + desc, nrow = 3) +
  theme_bw()
```

### Okinawa–Osaka

```
pOkiOsa <- twoBySixFigure(
  computePlotData_simplified("Oki", "Osa", reps = reps, CE = ces$ce__50__48, direction = "right"),
  computePlotData_simplified("Oki", "Osa", annots = annots, operons = operons, direction = "right", withGenes = "only"),
  computePlotData_simplified("Oki", "Osa", annots = annots, direction = "right", withGenes = "no")
) +
  ggtitle("Okinawa - Osaka")

pOkiOsa + ggtitle("Aligned on regions left boundary. CNE is 50/48.  Okinawa - Osaka")
```

### Okinawa–Barcelona

```
pOkiBar <- twoBySixFigure(
  computePlotData_simplified("Oki", "Bar", reps = reps, CE = ces$ce__50__48, direction = "right"),
  computePlotData_simplified("Oki", "Bar", annots = annots, operons = operons, direction = "right", withGenes = "only"),
  computePlotData_simplified("Oki", "Bar", annots = annots, direction = "right", withGenes = "no")
) +
  ggtitle("Okinawa - Barcelona")
```

### Okinawa–Kume

```
pOkiKum <- twoBySixFigure(
  computePlotData_simplified("Oki", "Kum", reps = reps, CE = ces$ce__50__48, direction = "right"),
  computePlotData_simplified("Oki", "Kum", annots = annots, operons = operons, direction = "right", withGenes = "only"),
  computePlotData_simplified("Oki", "Kum", annots = annots, direction = "right", withGenes = "no")
) +
  ggtitle("Okinawa - Kum")
```

```
## Warning in valid.GenomicRanges.seqinfo(x, suggest.trim = TRUE): GRanges object contains 1 out-of-bound range located on sequence PAR.
##   Note that ranges located on a sequence whose length is unknown (NA) or
##   on a circular sequence are not considered out-of-bound (use
##   seqlengths() and isCircular() to get the lengths and circularity flags
##   of the underlying sequences). You can use trim() to trim these ranges.
##   See ?`trim,GenomicRanges-method` for more information.

## Warning in valid.GenomicRanges.seqinfo(x, suggest.trim = TRUE): GRanges object contains 1 out-of-bound range located on sequence PAR.
##   Note that ranges located on a sequence whose length is unknown (NA) or
##   on a circular sequence are not considered out-of-bound (use
##   seqlengths() and isCircular() to get the lengths and circularity flags
##   of the underlying sequences). You can use trim() to trim these ranges.
##   See ?`trim,GenomicRanges-method` for more information.

## Warning in valid.GenomicRanges.seqinfo(x, suggest.trim = TRUE): GRanges object contains 1 out-of-bound range located on sequence PAR.
##   Note that ranges located on a sequence whose length is unknown (NA) or
##   on a circular sequence are not considered out-of-bound (use
##   seqlengths() and isCircular() to get the lengths and circularity flags
##   of the underlying sequences). You can use trim() to trim these ranges.
##   See ?`trim,GenomicRanges-method` for more information.
```

### Okinawa plots

```
pOkiOsa + pOkiBar + pOkiKum + plot_layout(guides = 'collect')
```

### Osaka–Okinawa

```
pOsaOki <- twoBySixFigure(
  computePlotData_simplified("Osa", "Oki", reps = reps, CE = ces$ce__50__48, direction = "right"),
  computePlotData_simplified("Osa", "Oki", annots = annots, operons = operons, direction = "right", withGenes = "only"),
  computePlotData_simplified("Osa", "Oki", annots = annots, direction = "right", withGenes = "no")
) +
  ggtitle("Osaka - Okinawa")
```

### Osaka–Barcelona

```
pOsaBar <- twoBySixFigure(
  computePlotData_simplified("Osa", "Bar", reps = reps, CE = ces$ce__50__48, direction = "right"),
  computePlotData_simplified("Osa", "Bar", annots = annots, operons = operons, direction = "right", withGenes = "only"),
  computePlotData_simplified("Osa", "Bar", annots = annots, direction = "right", withGenes = "no")
) +
  ggtitle("Osaka - Barcelona")
```

```
## Warning in valid.GenomicRanges.seqinfo(x, suggest.trim = TRUE): GRanges object contains 1 out-of-bound range located on sequence Chr1.
##   Note that ranges located on a sequence whose length is unknown (NA) or
##   on a circular sequence are not considered out-of-bound (use
##   seqlengths() and isCircular() to get the lengths and circularity flags
##   of the underlying sequences). You can use trim() to trim these ranges.
##   See ?`trim,GenomicRanges-method` for more information.

## Warning in valid.GenomicRanges.seqinfo(x, suggest.trim = TRUE): GRanges object contains 1 out-of-bound range located on sequence Chr1.
##   Note that ranges located on a sequence whose length is unknown (NA) or
##   on a circular sequence are not considered out-of-bound (use
##   seqlengths() and isCircular() to get the lengths and circularity flags
##   of the underlying sequences). You can use trim() to trim these ranges.
##   See ?`trim,GenomicRanges-method` for more information.

## Warning in valid.GenomicRanges.seqinfo(x, suggest.trim = TRUE): GRanges object contains 1 out-of-bound range located on sequence Chr1.
##   Note that ranges located on a sequence whose length is unknown (NA) or
##   on a circular sequence are not considered out-of-bound (use
##   seqlengths() and isCircular() to get the lengths and circularity flags
##   of the underlying sequences). You can use trim() to trim these ranges.
##   See ?`trim,GenomicRanges-method` for more information.
```

### Osaka–Aomori

```
pOsaAom <- twoBySixFigure(
  computePlotData_simplified("Osa", "Aom", reps = reps, CE = ces$ce__50__48, direction = "right"),
  computePlotData_simplified("Osa", "Aom", annots = annots, operons = operons, direction = "right", withGenes = "only"),
  computePlotData_simplified("Osa", "Aom", annots = annots, direction = "right", withGenes = "no")
) +
  ggtitle("Osaka - Aomori")
```

```
## Warning in valid.GenomicRanges.seqinfo(x, suggest.trim = TRUE): GRanges object contains 2 out-of-bound ranges located on sequences Chr1
##   and Chr2. Note that ranges located on a sequence whose length is
##   unknown (NA) or on a circular sequence are not considered out-of-bound
##   (use seqlengths() and isCircular() to get the lengths and circularity
##   flags of the underlying sequences). You can use trim() to trim these
##   ranges. See ?`trim,GenomicRanges-method` for more information.

## Warning in valid.GenomicRanges.seqinfo(x, suggest.trim = TRUE): GRanges object contains 2 out-of-bound ranges located on sequences Chr1
##   and Chr2. Note that ranges located on a sequence whose length is
##   unknown (NA) or on a circular sequence are not considered out-of-bound
##   (use seqlengths() and isCircular() to get the lengths and circularity
##   flags of the underlying sequences). You can use trim() to trim these
##   ranges. See ?`trim,GenomicRanges-method` for more information.

## Warning in valid.GenomicRanges.seqinfo(x, suggest.trim = TRUE): GRanges object contains 2 out-of-bound ranges located on sequences Chr1
##   and Chr2. Note that ranges located on a sequence whose length is
##   unknown (NA) or on a circular sequence are not considered out-of-bound
##   (use seqlengths() and isCircular() to get the lengths and circularity
##   flags of the underlying sequences). You can use trim() to trim these
##   ranges. See ?`trim,GenomicRanges-method` for more information.
```

### Osaka plots

```
pOsaOki + pOsaBar + pOsaAom + plot_layout(guides = 'collect')
```

## Extra genomic feature coverage plots.

### Bridge regions classified by width.

Short bridge regions contain small introns. Long bridge regions
contain repeats, which may be in introns.

```
computePlotData_for_bridge <- function(target, query, annots=NULL, reps=NULL, CE=NULL, tfbs=NULL, operons=NULL, direction="left") {
  pair <- paste(target, query, sep = "_")

  current_GB_bri       <- bri[[pair]]
  current_GB_bri_small <- current_GB_bri[width(current_GB_bri) <  150]
  current_GB_bri_long  <- current_GB_bri[width(current_GB_bri) >= 150]

  targetGenome <- unique(genome(gbs[[pair]]))
  
  prepareFreqPlot_default <- \(x) prepareFreqPlot(x, annot = annots[[target]], rep = reps[[targetGenome]], cne = CE[[pair]], tfbs = tfbs, operons = operons[[target]], direction = direction)
  
  hmList <- SimpleList()
  hmList$bri       <- prepareFreqPlot_default(current_GB_bri)
  hmList$bri_small <- prepareFreqPlot_default(current_GB_bri_small)
  hmList$bri_long  <- prepareFreqPlot_default(current_GB_bri_long)
  hmList
}

hmList_to_plot_noncoding <- computePlotData_for_bridge("Oki", "Osa", reps = reps, CE = ces$ce__50__48)
hmList_to_plot_coding    <- computePlotData_for_bridge("Oki", "Osa", annots = annots, operons = operons)

list_of_heatmaps_to_long_tibble <- function(hmList_to_plot, desc = NULL) {
  tib <-rbind(
    hmList_to_plot$bri         |> prepareGGFreqPlot("01_bridge"),
    hmList_to_plot$bri_small   |> prepareGGFreqPlot("02_bridge_small"),
    hmList_to_plot$bri_long    |> prepareGGFreqPlot("02_bridge_long")
  )
  tib$desc2 <- desc
  tib
}

rbind(
  list_of_heatmaps_to_long_tibble(hmList_to_plot_noncoding, "noncoding"),
  list_of_heatmaps_to_long_tibble(hmList_to_plot_coding, "coding")
) |> ggplot() +
  aes(pos, freq, col = what) +
  geom_line() +
  facet_wrap(~desc2 + desc, nrow = 2) +
  theme_bw() +
  ggtitle("Aligned on regions left boundary. CNE is 50/48.  Okinawa - Osaka")
```

### Aligning on center instead of border.

```
hmList_to_plot.centered <- computePlotData("Oki", "Osa", annots = annots, CE = ces$ce__50__48, tfbs =  tfbs, operons = operons, direction = "mid")

(p_boundary <- rbind(
  hmList_to_plot.centered$aln_nonCoa  |> prepareGGFreqPlot("01_isolated_alns"),
  hmList_to_plot.centered$unmap       |> prepareGGFreqPlot("02_unmapped"),
  hmList_to_plot.centered$syn_block   |> prepareGGFreqPlot("03_colinear_reg"),
  hmList_to_plot.centered$bri         |> prepareGGFreqPlot("04_bridge"),
  hmList_to_plot.centered$aln_coa     |> prepareGGFreqPlot("05_colinear_alns")

)|> ggplot() +
  aes(pos, freq, col = what) +
  geom_line() +
  facet_wrap(~desc, nrow = 1) +
  theme_bw() +
  ggtitle("Aligned on center of regions. CNE is 50/48.  Okinawa - Osaka"))
```

### Splice junctions

```
rbind(
  feature_coverage(gbs$Oki_Osa[gbs$Oki_Osa$nonCoa], intronicParts(annots$Oki) |> resize(1), window = 1000, lab = "splicejunctions", dir = "left") |>
    Heatmap_to_freq_tibble("01_isolated"),
  feature_coverage(unal$Oki_Osa[unal$Oki_Osa$nonCoa], intronicParts(annots$Oki) |> resize(1), window = 1000, lab = "splicejunctions", dir = "left") |>
    Heatmap_to_freq_tibble("02_unaligned"),
  feature_coverage(coa$Oki_Osa[!coa$Oki_Osa$nonCoa], intronicParts(annots$Oki) |> resize(1), window = 1000, lab = "splicejunctions", dir = "left") |>
    Heatmap_to_freq_tibble("03_colinear_reg"),
  feature_coverage(bri$Oki_Osa, intronicParts(annots$Oki) |> resize(1), window = 1000, lab = "splicejunctions", dir = "left") |>
    Heatmap_to_freq_tibble("04_bridge") 
) |> ggplot() + aes(pos, freq, col = desc) + geom_line()
```

### PWM

The PWM motif, which is rare in comparison to the other features,
follows a profile that bears resemblance to the repeat profile.

```
gadems.invLeftGap <- readRDS("gadems.invLeftGap.Rda")
pwm <- gadems.invLeftGap$Oki_Osa[[1]]
pwm@consensus
```

```
## [1] "rArAAGCCGCdwAGCsGCw"
```

```
# pwmHits <- matchPWM(pwm = sapply(pwm@alignList, \(x) x@seq) |> as("DNAStringSet") |> PWM(), genomes$Oki) # Gives different scores...
pwmHits <- matchPWM(pwm = rGADEM::getPWM(pwm), genomes$OKI2018.I69) |> suppressWarnings()
# Suppressing warnings like:
# Warning messages:
# 1: In .Call2("XString_match_PWM", pwm, subject, min.score, count.only,  :
#   'subject' contains letters not in [ACGT] ==> assigned weight 0 to them
# 
# plotHeatmapMeta(list(feature_coverage(current_GB_wide,   pwmHits, win = 1e3, lab = "wide",    direction = "left")))
# plotHeatmapMeta(list(feature_coverage(current_GB_narrow, pwmHits, win = 1e3, lab = "narrow",  direction = "left")))
```

### Gene feature coverage around PWM hits

Let’s look at coverage around PWM hits of the AAGCsGCwwmkCGrCTTyn
motif

```
current_GB <- pwmHits

hmList_PWM_OKI <- list()
hmList_PWM_OKI$genes     <- feature_coverage(current_GB, genes(annots$Oki),                         win = 2000, lab = "Oki genes", direction = "left")
hmList_PWM_OKI$promoters <- feature_coverage(current_GB, promoters(annots$Oki),                     win = 2000, lab = "Oki promoters", direction = "left")
```

```
## Warning in valid.GenomicRanges.seqinfo(x, suggest.trim = TRUE): GRanges object contains 4 out-of-bound ranges located on sequences
##   chr1, chrUn_1, and chrUn_10. Note that ranges located on a sequence
##   whose length is unknown (NA) or on a circular sequence are not
##   considered out-of-bound (use seqlengths() and isCircular() to get the
##   lengths and circularity flags of the underlying sequences). You can use
##   trim() to trim these ranges. See ?`trim,GenomicRanges-method` for more
##   information.
```

```
hmList_PWM_OKI$exons     <- feature_coverage(current_GB, exonicParts(annots$Oki),                   win = 2000, lab = "Oki exons", direction = "left")
hmList_PWM_OKI$introns   <- feature_coverage(current_GB, intronicParts(annots$Oki),                 win = 2000, lab = "Oki introns", direction = "left")
hmList_PWM_OKI$cds       <- feature_coverage(current_GB, cds(annots$Oki),                           win = 2000, lab = "Oki cds", direction = "left")

plotHeatmapMeta(hmList_PWM_OKI[c("genes", "exons", "cds")]) # Show they look alike.  We keep "exons"
```

```
plotHeatmapMeta(hmList_PWM_OKI[c("promoters", "exons", "introns")])
```

They are enriched in introns…

```
# 
# # Sandbox to make sanity checks.
# 
# a <- coa$Oki_Osa
# b <- unlist(fiveUTRsByTranscript(annots$Oki))
# 
# hm_a <- feature_coverage(a, b, win = 2000, lab = "All ranges", direction = "left")
# hm_b <- feature_coverage(subsetByOverlaps(a,  b), b, win = 2000, lab = "Subsetted ranges", direction = "left")
# 
# plotHeatmapMeta(list(hm_a))
# plotHeatmapMeta(list(hm_b))
```

### Breakpoint coverage around PWM hits

TODO: triplecheck `feature_coverage`, it is old…

```
current_GB <- pwmHits

hmList_PWM_OKI$gbOsa     <- feature_coverage(current_GB, gbs$Oki_Osa,  win = 5000, lab = "Oki-Osa", direction = "left")
hmList_PWM_OKI$gbBar     <- feature_coverage(current_GB, gbs$Oki_Bar,  win = 5000, lab = "Oki-Bar", direction = "left")
hmList_PWM_OKI$gbKum     <- feature_coverage(current_GB, gbs$Oki_Kum,  win = 5000, lab = "Oki-Kum", direction = "left")

plotHeatmapMeta(hmList_PWM_OKI[c("gbOsa", "gbBar", "gbKum")])
```

```
hmList_PWM_OKI$coOsa     <- feature_coverage(current_GB, coa$Oki_Osa,  win = 5000, lab = "Oki-Osa", direction = "left")
hmList_PWM_OKI$coBar     <- feature_coverage(current_GB, coa$Oki_Bar,  win = 5000, lab = "Oki-Bar", direction = "left")
hmList_PWM_OKI$coKum     <- feature_coverage(current_GB, coa$Oki_Kum,  win = 5000, lab = "Oki-Kum", direction = "left")

plotHeatmapMeta(hmList_PWM_OKI[c("coOsa", "coBar", "coKum")])
```

### Okinawa genome coverage

As previously discussed, low coverage over an alignment stop could
lower the likelihood of it being considered as a breakpoint. We have
per-base coverage depth information for the Okinawan genome
(`Oki_cov_pb`). However, the coverage of this particular
assembly is quite good. In fact, we can investigate the coverge over
alignment stops from the information obtained using
`master_bp_analysis`.

```
# fin_Oki <- fin_gr_O_Oki[[2]]
# min_cov <- min(min(fin_Oki$left_cov_pb), min(fin_Oki$right_cov_pb)) # minimum coverage over an alignment stop
# min_cov
# length(fin_Oki[fin_Oki$left_cov_pb == min_cov]) + length(fin_Oki[fin_Oki$right_cov_pb == min_cov]) # how many of the minimum coverage is observed
# length(fin_Oki[fin_Oki$left_cov_pb <= 50]) + length(fin_Oki[fin_Oki$right_cov_pb <= 50]) # how many alignment stops have a coverage of less than or euqal to 50
```

We may choose to kick out the one alignment stop for which there is
no coverage. However, only 15 out of 34572 alignment stops have a
coverage of less than or equal to 50, meaning that it would be hard to
exclude more than just a few alignment stops using coverage information
for breakpoint analysis.

## Operons and genes

Do alignments often cross gene or operon boundaries ?

### Operons boundaries

Operon boundaries tend to coincide with alignment boundaries and the
largest shift in frequency is for colinear alignments. Baseline
frequency of colinear regions is higher than 0.6 on both side of operon
boundaries.

```
feature_coverage_on_operons <- {
  function(feat, desc)
    feature_coverage(operons$Oki, feat, window = 1000, lab = "", dir = "left") |> Heatmap_to_freq_tibble(desc)
}

pairname <- "Oki_Bar"
pairname <- "Oki_Osa"

p_op_scrambling <- rbind(
  feature_coverage_on_operons(gbs[[pairname]][gbs[[pairname]]$nonCoa], "01_isolated"),
  feature_coverage_on_operons(unal[[pairname]][unal[[pairname]]$nonCoa], "02_unaligned"),
  feature_coverage_on_operons(gbs[[pairname]][!gbs[[pairname]]$nonCoa], "03_colinear_aln"),
  #feature_coverage_on_operons(subsetByOverlaps(gbs$Oki_Osa[!gbs$Oki_Osa$nonCoa], granges(coa$Oki_Osa[!coa$Oki_Osa$nonCoa] - 1),type = "within"), "03b_colinear_aln_intern"),
  #feature_coverage_on_operons(subsetByOverlaps(gbs$Oki_Osa[!gbs$Oki_Osa$nonCoa], unal$Oki_Osa[unal$Oki_Osa$nonCoa] + 1), "03c_colinear_aln_extern"),

  feature_coverage_on_operons(bri[[pairname]], "04_bridge"),
  feature_coverage_on_operons(coa[[pairname]][!coa[[pairname]]$nonCoa], "05_colinear_reg")
) |> ggplot() + aes(pos, freq, col = desc) + geom_line() + ggtitle("operons", subtitle = "n = 2017") + theme_bw()
```

### Gene boundaries inside operons

```
# Shrinking operons of 1 base and requesting full overlap
# in order to exclude the genes at the boundaries.
genes_inside_operons  <- subsetByOverlaps(genes(annots$Oki),           operons$Oki -1, type = "within")
genes_outside_operons <- subsetByOverlaps(genes(annots$Oki), cleanGaps(operons$Oki),   type = "within")

feature_coverage_on_genes <- {
  function(feat, desc)
    feature_coverage(genes_inside_operons, feat, window = 1000, lab = "", dir = "left") |> Heatmap_to_freq_tibble(desc)
}

p_gene_int_scrambling <- rbind(
  feature_coverage_on_genes(gbs$Oki_Osa[gbs$Oki_Osa$nonCoa], "01_isolated"),
  feature_coverage_on_genes(unal$Oki_Osa[unal$Oki_Osa$nonCoa], "02_unmapped"),
  feature_coverage_on_genes(gbs$Oki_Osa[!gbs$Oki_Osa$nonCoa], "03_colinear_aln"),
  feature_coverage_on_genes(bri$Oki_Osa, "04_bridge"),
  feature_coverage_on_genes(coa$Oki_Osa[!coa$Oki_Osa$nonCoa], "05_colinear_reg")
) |> ggplot() + aes(pos, freq, col = desc) + geom_line() + ggtitle("genes_internal_to_operons", subtitle = "n = 1348") + theme_bw()

feature_coverage_on_genes_outside <- {
  function(feat, desc)
    feature_coverage(genes_outside_operons, feat, window = 1000, lab = "", dir = "left") |> Heatmap_to_freq_tibble(desc)
}

p_gene_ext_scrambling <- rbind(
  feature_coverage_on_genes_outside(gbs$Oki_Osa[gbs$Oki_Osa$nonCoa], "01_isolated"),
  feature_coverage_on_genes_outside(unal$Oki_Osa[unal$Oki_Osa$nonCoa], "02_unmapped"),
  feature_coverage_on_genes_outside(gbs$Oki_Osa[!gbs$Oki_Osa$nonCoa], "03_colinear_aln"),
  feature_coverage_on_genes_outside(bri$Oki_Osa, "04_bridge"),
  feature_coverage_on_genes_outside(coa$Oki_Osa[!coa$Oki_Osa$nonCoa], "05_colinear_reg")
) |> ggplot() + aes(pos, freq, col = desc) + geom_line() + ggtitle("genes_outside_operons", subtitle = "n = 11688") + theme_bw()
```

### Summary plot

```
p_gene_ext_scrambling + p_op_scrambling + p_gene_int_scrambling + plot_layout(nrow = 1, guides = 'collect')
```

### Operon conservation

```
# Distribution of operon lengths
operons$Oki$n |> table()
```

```
## 
##    2    3    4    5    6    7    8    9   10   11   14 
## 1943  647  259  134   69   33   19   10    6    3    1
```

```
# An operon is scrambled if it overlaps with an unaligned noncoalesced (= unmapped) region
scrambled_operons     <- subsetByOverlaps(operons$Oki, unal$Oki_Osa[unal$Oki_Osa$nonCoa])
not_scrambled_operons <- operons$Oki[! operons$Oki %in% scrambled_operons ]

# Distribution of operon lengths (scrambled)
scrambled_operons$n     |> table()
```

```
## 
##   2   3   4   5   6   7   8   9  10  11 
## 905 298 106  44  31   9   7   6   4   3
```

```
not_scrambled_operons$n |> table()
```

```
## 
##    2    3    4    5    6    7    8    9   10   14 
## 1038  349  153   90   38   24   12    4    2    1
```

```
# Example of long scrambled operons
scrambled_operons[scrambled_operons$n > 5]
```

```
## GRanges object with 60 ranges and 2 metadata columns:
##        seqnames            ranges strand |         n                  gene_id
##           <Rle>         <IRanges>  <Rle> | <integer>          <CharacterList>
##    [1]     chr1   5715727-5730794      + |         7    g1521,g1522,g1523,...
##    [2]     chr1   6147428-6157837      + |         6    g1613,g1614,g1615,...
##    [3]     chr1   9809914-9836205      + |        10    g2620,g2621,g2622,...
##    [4]     chr1 10022216-10035952      + |         7    g2668,g2669,g2670,...
##    [5]     chr1 10547772-10569235      - |         9    g2832,g2833,g2834,...
##    ...      ...               ...    ... .       ...                      ...
##   [56]      XSR   6611822-6622862      + |         7 g15207,g15208,g15209,...
##   [57]      XSR   6843015-6861410      + |         8 g15282,g15283,g15284,...
##   [58]      XSR   7875989-7897781      - |         7 g15606,g15607,g15608,...
##   [59]      XSR 10693937-10705412      + |         6 g16499,g16500,g16501,...
##   [60]      XSR 12736159-12757464      - |         8 g17016,g17017,g17018,...
##   -------
##   seqinfo: 19 sequences from OKI2018.I69 genome
```

In comparison: genes

```
all_genes <- genes(annots$Oki)
scrambled_genes <- (subsetByOverlaps(all_genes, unal$Oki_Osa[unal$Oki_Osa$nonCoa])) |> sort(i=F) |> flagLongShort(longShort$OKI2018.I69)
not_scrambled_genes <- all_genes[! all_genes %in% scrambled_genes]                  |> sort(i=F) |> flagLongShort(longShort$OKI2018.I69)

length(scrambled_genes)
```

```
## [1] 5294
```

```
length(all_genes)
```

```
## [1] 17291
```

```
scr_g_tbl <- table(paste(seqnames(    scrambled_genes), scrambled_genes$Arm))
nsc_g_tbl <- table(paste(seqnames(not_scrambled_genes), not_scrambled_genes$Arm))

Oki_Osa_scr_ratio_long_arm <- c(
  scr_g_tbl[["chr1 long"]] / ( scr_g_tbl[["chr1 long"]] + nsc_g_tbl[["chr1 long"]]),
  scr_g_tbl[["chr2 long"]] / ( scr_g_tbl[["chr2 long"]] + nsc_g_tbl[["chr2 long"]]),
  scr_g_tbl[["PAR long"]]  / ( scr_g_tbl[["PAR long"]]  + nsc_g_tbl[["PAR long"]])
)
mean(Oki_Osa_scr_ratio_long_arm)
```

```
## [1] 0.2314521
```

```
Oki_Osa_scr_ratio_short_arm <- c(
  scr_g_tbl[["chr1 short"]] / ( scr_g_tbl[["chr1 short"]] + nsc_g_tbl[["chr1 short"]]),
  scr_g_tbl[["chr2 short"]] / ( scr_g_tbl[["chr2 short"]] + nsc_g_tbl[["chr2 short"]]),
  scr_g_tbl[["PAR short"]]  / ( scr_g_tbl[["PAR short"]]  + nsc_g_tbl[["PAR short"]])
)
mean(Oki_Osa_scr_ratio_short_arm)
```

```
## [1] 0.5374672
```

```
t.test(Oki_Osa_scr_ratio_long_arm, Oki_Osa_scr_ratio_short_arm, paired = T)
```

```
## 
##  Paired t-test
## 
## data:  Oki_Osa_scr_ratio_long_arm and Oki_Osa_scr_ratio_short_arm
## t = -9.5775, df = 2, p-value = 0.01073
## alternative hypothesis: true mean difference is not equal to 0
## 95 percent confidence interval:
##  -0.4434904 -0.1685397
## sample estimates:
## mean difference 
##      -0.3060151
```

Replicate on an independent pair of genomes.

```
all_genes <- genes(annots$Bar)
scrambled_genes <- (subsetByOverlaps(all_genes, unal$Bar_Kum[unal$Bar_Kum$nonCoa])) |> sort(i=F) |> flagLongShort(longShort$Bar2.p4)
not_scrambled_genes <- all_genes[! all_genes %in% scrambled_genes]                  |> sort(i=F) |> flagLongShort(longShort$Bar2.p4)

length(scrambled_genes)
```

```
## [1] 4002
```

```
length(all_genes)
```

```
## [1] 14272
```

```
scr_g_tbl <- table(paste(seqnames(    scrambled_genes), scrambled_genes$Arm))
nsc_g_tbl <- table(paste(seqnames(not_scrambled_genes), not_scrambled_genes$Arm))

Bar_Kum_scr_ratio_long_arm <- c(
  scr_g_tbl[["Chr1 long"]] / ( scr_g_tbl[["Chr1 long"]] + nsc_g_tbl[["Chr1 long"]]),
  scr_g_tbl[["Chr2 long"]] / ( scr_g_tbl[["Chr2 long"]] + nsc_g_tbl[["Chr2 long"]]),
  scr_g_tbl[["PAR long"]]  / ( scr_g_tbl[["PAR long"]]  + nsc_g_tbl[["PAR long"]])
)
Bar_Kum_scr_ratio_short_arm <- c(
  scr_g_tbl[["Chr1 short"]] / ( scr_g_tbl[["Chr1 short"]] + nsc_g_tbl[["Chr1 short"]]),
  scr_g_tbl[["Chr2 short"]] / ( scr_g_tbl[["Chr2 short"]] + nsc_g_tbl[["Chr2 short"]]),
  scr_g_tbl[["PAR short"]]  / ( scr_g_tbl[["PAR short"]]  + nsc_g_tbl[["PAR short"]])
)

t.test(Bar_Kum_scr_ratio_long_arm, Bar_Kum_scr_ratio_short_arm, paired = TRUE)
```

```
## 
##  Paired t-test
## 
## data:  Bar_Kum_scr_ratio_long_arm and Bar_Kum_scr_ratio_short_arm
## t = -16.994, df = 2, p-value = 0.003445
## alternative hypothesis: true mean difference is not equal to 0
## 95 percent confidence interval:
##  -0.3825454 -0.2279699
## sample estimates:
## mean difference 
##      -0.3052576
```

```
mean(c(Oki_Osa_scr_ratio_long_arm, Bar_Kum_scr_ratio_long_arm))
```

```
## [1] 0.2055334
```

```
mean(c(Oki_Osa_scr_ratio_short_arm, Bar_Kum_scr_ratio_short_arm))
```

```
## [1] 0.5111697
```

```
t.test(
  c(Oki_Osa_scr_ratio_long_arm, Bar_Kum_scr_ratio_long_arm),
  c(Oki_Osa_scr_ratio_short_arm, Bar_Kum_scr_ratio_short_arm),
  paired = TRUE
)
```

```
## 
##  Paired t-test
## 
## data:  c(Oki_Osa_scr_ratio_long_arm, Bar_Kum_scr_ratio_long_arm) and c(Oki_Osa_scr_ratio_short_arm, Bar_Kum_scr_ratio_short_arm)
## t = -18.644, df = 5, p-value = 8.172e-06
## alternative hypothesis: true mean difference is not equal to 0
## 95 percent confidence interval:
##  -0.3477765 -0.2634962
## sample estimates:
## mean difference 
##      -0.3056363
```

How about exons ?

```
all_exons <- exonicParts(annots$Oki)
scrambled_exons <- (subsetByOverlaps(all_exons, unal$Oki_Osa[unal$Oki_Osa$nonCoa])) |> sort(i=F) |> flagLongShort(longShort$OKI2018.I69)
not_scrambled_exons <- all_exons[! all_exons %in% scrambled_exons]                  |> sort(i=F) |> flagLongShort(longShort$OKI2018.I69)

length(scrambled_exons)
```

```
## [1] 16787
```

```
length(all_exons)
```

```
## [1] 106811
```

```
scr_g_tbl <- table(paste(seqnames(    scrambled_exons), scrambled_exons$Arm))
nsc_g_tbl <- table(paste(seqnames(not_scrambled_exons), not_scrambled_exons$Arm))

Oki_Osa_scr_ratio_long_arm <- c(
  scr_g_tbl[["chr1 long"]] / ( scr_g_tbl[["chr1 long"]] + nsc_g_tbl[["chr1 long"]]),
  scr_g_tbl[["chr2 long"]] / ( scr_g_tbl[["chr2 long"]] + nsc_g_tbl[["chr2 long"]]),
  scr_g_tbl[["PAR long"]]  / ( scr_g_tbl[["PAR long"]]  + nsc_g_tbl[["PAR long"]])
)
mean(Oki_Osa_scr_ratio_long_arm)
```

```
## [1] 0.1172023
```

```
Oki_Osa_scr_ratio_short_arm <- c(
  scr_g_tbl[["chr1 short"]] / ( scr_g_tbl[["chr1 short"]] + nsc_g_tbl[["chr1 short"]]),
  scr_g_tbl[["chr2 short"]] / ( scr_g_tbl[["chr2 short"]] + nsc_g_tbl[["chr2 short"]]),
  scr_g_tbl[["PAR short"]]  / ( scr_g_tbl[["PAR short"]]  + nsc_g_tbl[["PAR short"]])
)
mean(Oki_Osa_scr_ratio_short_arm)
```

```
## [1] 0.3003286
```

```
t.test(Oki_Osa_scr_ratio_long_arm, Oki_Osa_scr_ratio_short_arm, paired = T)
```

```
## 
##  Paired t-test
## 
## data:  Oki_Osa_scr_ratio_long_arm and Oki_Osa_scr_ratio_short_arm
## t = -5.9254, df = 2, p-value = 0.02732
## alternative hypothesis: true mean difference is not equal to 0
## 95 percent confidence interval:
##  -0.31610038 -0.05015222
## sample estimates:
## mean difference 
##      -0.1831263
```

## Session information

```
sessionInfo()
```

```
## R version 4.3.1 (2023-06-16)
## Platform: x86_64-pc-linux-gnu (64-bit)
## Running under: Debian GNU/Linux 12 (bookworm)
## 
## Matrix products: default
## BLAS:   /usr/lib/x86_64-linux-gnu/blas/libblas.so.3.11.0 
## LAPACK: /usr/lib/x86_64-linux-gnu/lapack/liblapack.so.3.11.0
## 
## locale:
##  [1] LC_CTYPE=en_US.UTF-8       LC_NUMERIC=C              
##  [3] LC_TIME=en_US.UTF-8        LC_COLLATE=en_US.UTF-8    
##  [5] LC_MONETARY=en_US.UTF-8    LC_MESSAGES=en_US.UTF-8   
##  [7] LC_PAPER=en_US.UTF-8       LC_NAME=C                 
##  [9] LC_ADDRESS=C               LC_TELEPHONE=C            
## [11] LC_MEASUREMENT=en_US.UTF-8 LC_IDENTIFICATION=C       
## 
## time zone: Etc/UTC
## tzcode source: system (glibc)
## 
## attached base packages:
## [1] stats4    stats     graphics  grDevices utils     datasets  methods  
## [8] base     
## 
## other attached packages:
##  [1] BSgenome.Oidioi.genoscope.OdB3_1.0.0   
##  [2] BSgenome.Oidioi.OIST.AOM.5.5f_1.0.1    
##  [3] BSgenome.Oidioi.OIST.KUM.M3.7f_1.0.1   
##  [4] BSgenome.Oidioi.OIST.Bar2.p4_1.0.1     
##  [5] BSgenome.Oidioi.OIST.OSKA2016v1.9_1.0.0
##  [6] BSgenome.Oidioi.OIST.OKI2018.I69_1.0.1 
##  [7] patchwork_1.1.2                        
##  [8] heatmaps_1.24.0                        
##  [9] GenomicFeatures_1.52.1                 
## [10] AnnotationDbi_1.62.2                   
## [11] Biobase_2.60.0                         
## [12] OikScrambling_5.1.0                    
## [13] ggplot2_3.4.4                          
## [14] GenomicBreaks_0.14.3                   
## [15] BSgenome_1.68.0                        
## [16] rtracklayer_1.60.1                     
## [17] Biostrings_2.68.1                      
## [18] XVector_0.40.0                         
## [19] GenomicRanges_1.52.1                   
## [20] GenomeInfoDb_1.36.4                    
## [21] IRanges_2.34.1                         
## [22] S4Vectors_0.38.2                       
## [23] BiocGenerics_0.46.0                    
## 
## loaded via a namespace (and not attached):
##   [1] splines_4.3.1               BiocIO_1.10.0              
##   [3] filelock_1.0.2              bitops_1.0-7               
##   [5] tibble_3.2.1                R.oo_1.25.0                
##   [7] XML_3.99-0.15               rpart_4.1.19               
##   [9] rGADEM_2.48.0               lifecycle_1.0.3            
##  [11] rprojroot_2.0.4             lattice_0.20-45            
##  [13] MASS_7.3-58.2               backports_1.4.1            
##  [15] magrittr_2.0.3              Hmisc_5.1-1                
##  [17] sass_0.4.7                  rmarkdown_2.25             
##  [19] jquerylib_0.1.4             yaml_2.3.7                 
##  [21] plotrix_3.8-3               DBI_1.1.3                  
##  [23] CNEr_1.36.0                 minqa_1.2.6                
##  [25] RColorBrewer_1.1-3          ade4_1.7-22                
##  [27] abind_1.4-5                 zlibbioc_1.46.0            
##  [29] purrr_1.0.2                 R.utils_2.12.2             
##  [31] RCurl_1.98-1.13             nnet_7.3-18                
##  [33] pracma_2.4.2                rappdirs_0.3.3             
##  [35] GenomeInfoDbData_1.2.10     seqLogo_1.66.0             
##  [37] gdata_3.0.0                 annotate_1.78.0            
##  [39] pkgdown_2.0.7               codetools_0.2-19           
##  [41] DelayedArray_0.26.7         xml2_1.3.5                 
##  [43] tidyselect_1.2.0            shape_1.4.6                
##  [45] ggseqlogo_0.1               farver_2.1.1               
##  [47] lme4_1.1-35.1               BiocFileCache_2.8.0        
##  [49] matrixStats_1.0.0           base64enc_0.1-3            
##  [51] GenomicAlignments_1.36.0    jsonlite_1.8.7             
##  [53] mitml_0.4-5                 Formula_1.2-5              
##  [55] survival_3.5-3              iterators_1.0.14           
##  [57] systemfonts_1.0.5           foreach_1.5.2              
##  [59] progress_1.2.2              tools_4.3.1                
##  [61] ragg_1.2.5                  Rcpp_1.0.11                
##  [63] glue_1.6.2                  gridExtra_2.3              
##  [65] pan_1.9                     xfun_0.41                  
##  [67] MatrixGenerics_1.12.3       EBImage_4.42.0             
##  [69] dplyr_1.1.3                 withr_2.5.2                
##  [71] fastmap_1.1.1               boot_1.3-28.1              
##  [73] fansi_1.0.5                 digest_0.6.33              
##  [75] R6_2.5.1                    mice_3.16.0                
##  [77] textshaping_0.3.7           colorspace_2.1-0           
##  [79] GO.db_3.17.0                gtools_3.9.4               
##  [81] poweRlaw_0.70.6             jpeg_0.1-10                
##  [83] biomaRt_2.56.1              RSQLite_2.3.3              
##  [85] weights_1.0.4               R.methodsS3_1.8.2          
##  [87] utf8_1.2.4                  tidyr_1.3.0                
##  [89] generics_0.1.3              data.table_1.14.8          
##  [91] prettyunits_1.2.0           httr_1.4.7                 
##  [93] htmlwidgets_1.6.2           S4Arrays_1.0.6             
##  [95] pkgconfig_2.0.3             gtable_0.3.4               
##  [97] blob_1.2.4                  htmltools_0.5.7            
##  [99] fftwtools_0.9-11            scales_1.2.1               
## [101] png_0.1-8                   knitr_1.45                 
## [103] rstudioapi_0.15.0           tzdb_0.4.0                 
## [105] reshape2_1.4.4              rjson_0.2.21               
## [107] curl_5.1.0                  checkmate_2.3.0            
## [109] nlme_3.1-162                nloptr_2.0.3               
## [111] cachem_1.0.8                stringr_1.5.0              
## [113] KernSmooth_2.23-20          parallel_4.3.1             
## [115] genoPlotR_0.8.11            foreign_0.8-84             
## [117] restfulr_0.0.15             desc_1.4.2                 
## [119] pillar_1.9.0                grid_4.3.1                 
## [121] vctrs_0.6.4                 jomo_2.7-6                 
## [123] dbplyr_2.3.3                xtable_1.8-4               
## [125] cluster_2.1.4               htmlTable_2.4.2            
## [127] evaluate_0.23               readr_2.1.4                
## [129] cli_3.6.1                   locfit_1.5-9.8             
## [131] compiler_4.3.1              Rsamtools_2.16.0           
## [133] rlang_1.1.2                 crayon_1.5.2               
## [135] labeling_0.4.3              plyr_1.8.9                 
## [137] fs_1.6.3                    stringi_1.7.12             
## [139] BiocParallel_1.34.2         munsell_0.5.0              
## [141] tiff_0.1-11                 glmnet_4.1-8               
## [143] Matrix_1.5-3                hms_1.1.3                  
## [145] bit64_4.0.5                 KEGGREST_1.40.1            
## [147] highr_0.10                  SummarizedExperiment_1.30.2
## [149] broom_1.0.5                 memoise_2.0.1              
## [151] bslib_0.5.1                 bit_4.0.5
```

## Contents

Developed by Aleksandra Bliznina, Michael Mansfield, Charles Plessy.

Site built with pkgdown 2.0.7.
